# Rh(I)-Catalyzed Regio- and Enantioselective Ring Opening of Vinyl Cyclopropanes

**DOI:** 10.1021/jacs.4c09490

**Published:** 2024-08-20

**Authors:** Stephen J. Webster, László B. Balázs, F. Wieland Goetzke, Violeta Stojalnikova, Ke Liu, Kirsten E. Christensen, Harold W. Mackenzie, Stephen P. Fletcher

**Affiliations:** Department of Chemistry, University of Oxford, 12 Mansfield Road, Oxford OX1 3TA, U.K.

## Abstract

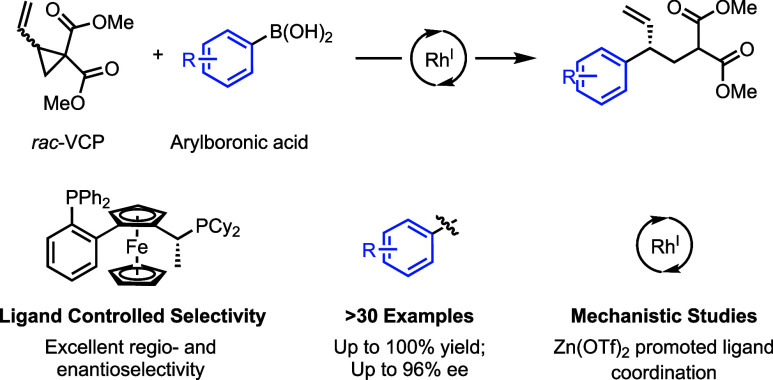

We describe a Rh(I) catalyzed asymmetric ring opening of *racemic* vinyl cyclopropanes using aryl boronic acids as C-nucleophiles. When ferrocene-based chiral bisphosphines are used as ligands, the products are obtained with regioselectivities typically 99:1 r.r. and ee’s generally between 88 and 96%. A wide range of aryl boronic acids can be used, and the products can be converted into a variety of targets. Preliminary mechanistic studies indicate that Zn(OTf)_2_ plays a significant role in the reaction by promoting rhodium-ligand complex formation and accelerating the reaction. We expect this method and these mechanistic insights to be useful in the development of new asymmetric methods.

## Introduction

**Scheme 1:**
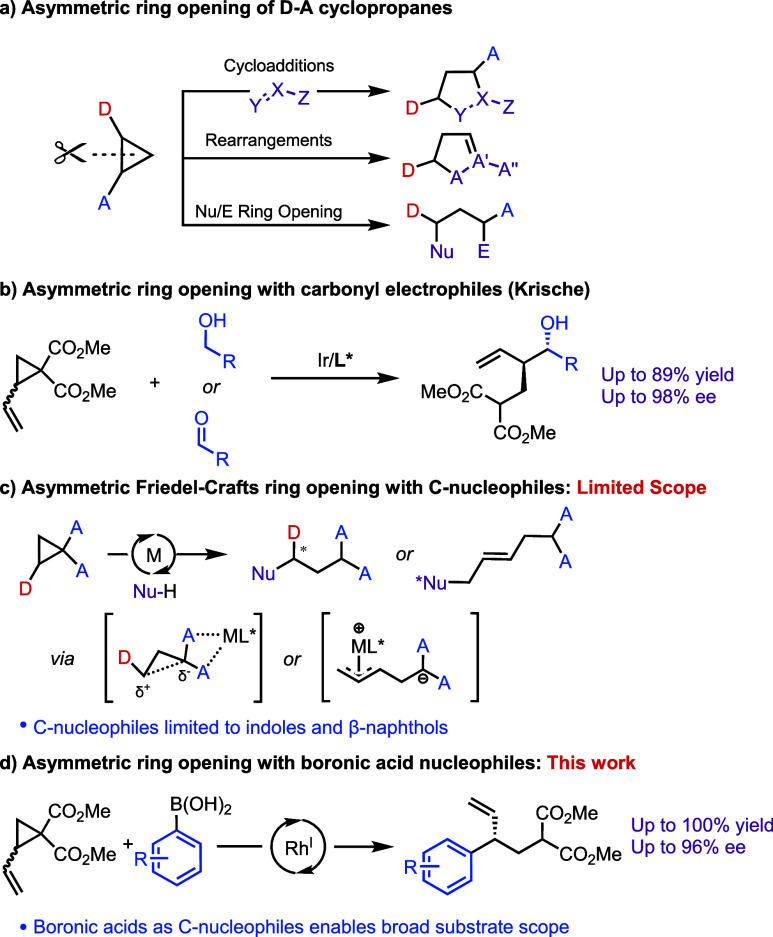
Asymmetric Ring Opening of D–A Cyclopropanes

The cyclopropane motif has attracted the attention of synthetic chemists for decades due to the perceived reactivity of the C–C bonds.^1^ Despite the significant ring strain the 3-membered-ring carries (27 kcal mol^–1^), the C–C bond of the cyclopropane is surprisingly kinetically inert.^2^ To overcome this barrier, chemists have activated the C–C bond through vicinal electron-donating and electron-withdrawing groups.^3^ These donor–acceptor (D–A) cyclopropanes are versatile building blocks in organic synthesis, and can be considered as 1,3-dipolar zwitterionic synthons for a variety of reactions. Reactions of D–A cyclopropanes can be classified into three distinct categories: (1) Annulations to yield carbo- or heterocycles; (2) Rearrangements resulting in ring expansion; and (3) Direct ring openings with electrophiles or nucleophiles (Scheme 1a).^4^

Catalytic asymmetric annulations with D–A cyclopropanes have been well studied and extensively reviewed.^5,6^ However, catalytic asymmetric ring opening reactions of D–A cyclopropanes with electrophiles and nucleophiles are less developed. This sharp contrast in the number of literature reports may be due to the challenges associated with this class of reactions, where both the regioselectivity (branched/linear) and enantioselectivity of the reactions need to be controlled.^7^ Krische and co-workers described direct opening of D–A cyclopropanes with carbonyl electrophiles through the generation of nucleophilic π-allyl-iridium intermediates (Scheme 1b).^8^ Despite sporadic reports of electrophilic opening, this remains the only enantioselective electrophilic opening of D–A cyclopropanes.^9,10^

Nucleophilic opening of D–A cyclopropanes provides a straightforward way to form chiral branched products containing multiple functional groups. It would be particularly attractive to develop this method to enantioselectively form C–C bonds, which is desirable in the synthesis of natural products and pharmaceuticals. Currently, asymmetric ring opening of D–A cyclopropanes with carbon nucleophiles are dominated by chiral Lewis acid chemistry, where the electrophilicity of the cyclopropane is increased by coordination, making it susceptible to Friedel–Crafts nucleophilic opening (Scheme 1c).^4^ In 2013, Johnson and co-workers developed an asymmetric Friedel–Crafts alkylation of indoles with D–A cyclopropanes using chiral Lewis acids.^11^ Chiral Lewis acids in asymmetric ring opening of D–A cyclopropanes have since been used with aminocyclopropanes^12^ and *meso*-cyclopropanes.^13^

Alternatively, a transition metal catalyst can be used to ring cleave D–A cyclopropanes that have a vinyl group as the donor moiety (Scheme 1c). To this effect, in 2018 Trost reported a Pd catalyzed opening of D–A cyclopropanes with indole nucleophiles to give indolenine products in good yield and ee.^14^ Similar transformations have since been reported,^7^ but to the best of our knowledge, all these methods are Friedel–Crafts based and require electron rich (hetero)aryl nucleophiles.

Based on our recent work on Rh catalysis,^15−22^ we wondered whether we could ring open vinyl cyclopropanes using a Rh(I) catalyst with aryl boronic acids (Scheme 1d). This would generate allyl-Rh-complexes, and form a C–C bond upon reductive elimination. The use of aryl boronic acids as nucleophiles is desirable as they are often commercially available and the method would not be limited to electron rich species. Here, we present a regio- and enantioselective rhodium-catalyzed asymmetric ring opening of *racemic* vinyl cyclopropanes with boronic acid nucleophiles.

## Results and Discussion

We began our study with *racemic* vinyl cyclopropane **1**, which has been used in a variety of ring openings.^23−25^ Extensive optimization studies led to [Rh(cod)(OH)]_2_ and **L1** as a catalyst complex, with Zn(OTf)_2_ as an additive, Cs_2_CO_3_ as the base and tetrahydropyran (THP) as the solvent, giving the branched product (**3a**) as a single regioisomer (99:1 r.r.) in high yield and enantioselectivity (Table 1, entry 1). The use of ferrocene ligands proved to be essential for regioselectivity, with other ligands providing less control.

**Table 1:**
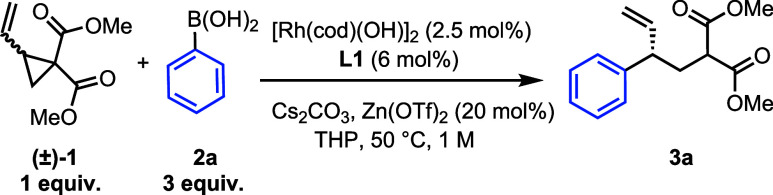

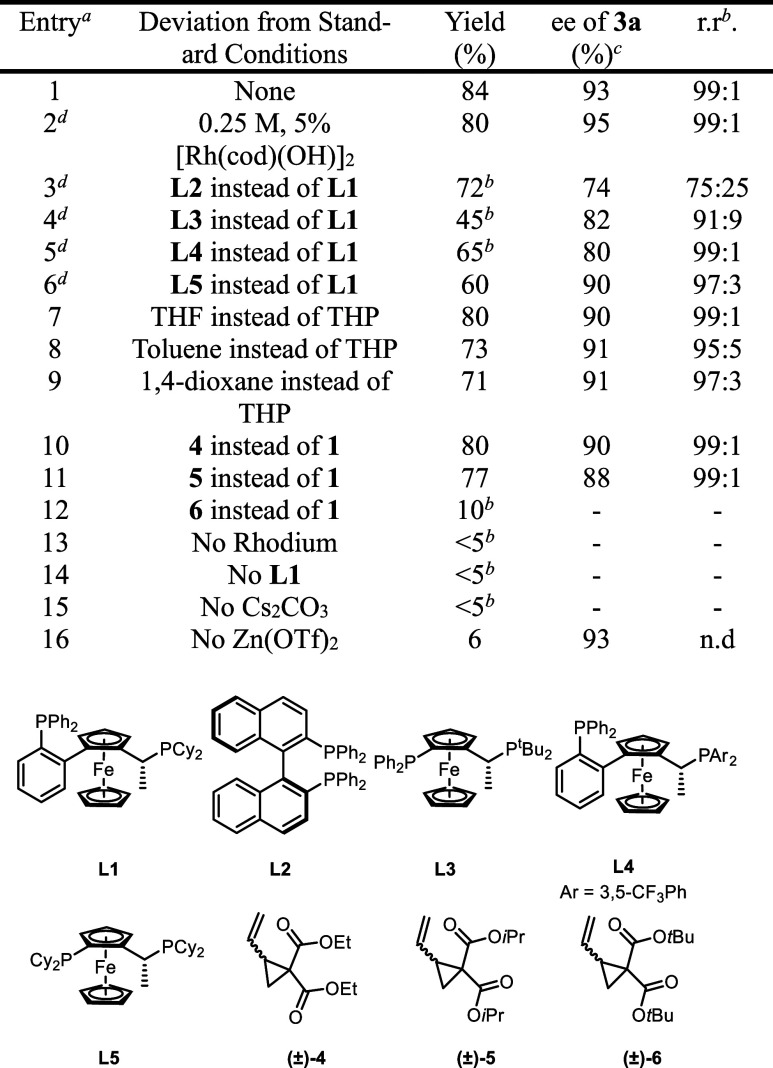
Selected Optimization Experiments for Asymmetric Ring Opening of Vinyl Cyclopropanes

a Reaction conditions: **1** (0.5 mmol, 1 equiv), **2a** (1.5 mmol, 3 equiv), [Rh(cod)(OH)]_2_ (0.0125 mmol, 2.5 mol %), ligand (0.03 mmol, 6 mol %), base (0.5 mmol, 1 equiv), Zn(OTf)_2_ (0.1 mmol, 0.2 equiv.), solvent (0.5 mL), 50 °C, 24 h.

b Yield and r.r. (regioselectivity ratio) of **3a** determined by ^1^H NMR spectroscopy; CH_2_Br_2_ used as internal standard.

c The ee values were determined by supercritical fluid chromatography (SFC) analysis on a chiral nonracemic stationary phase.

d Reaction conditions: **1** (0.4 mmol, 1 equiv), **2a** (1.2 mmol, 3 equiv), [Rh(cod)(OH)]_2_ (0.02 mmol, 5 mol %), ligand (0.048 mmol, 12 mol %), Cs_2_CO_3_ (0.4 mmol, 1 equiv), Zn(OTf)_2_ (0.08 mmol, 0.2 equiv), solvent (1.6 mL, 0.25 M), 60 °C, 16–24 h.

C2-symmetric bisphosphine ligands are useful for Rh-catalyzed asymmetric reactions, however neither **L2** (Table 1, entry 3) nor any other tested C2-symmetric bisphosphines afforded **3a** in high yield and ee. Ferrocene-based ligands provided better stereocontrol in general, although the yield and regioselectivity with these ligands varied (Table 1, entries 4–6). Other solvents saw a slight decrease in yield/ee (Table 1, entries 7–9). Related diester starting materials (Et and ^*i*^Pr esters) can also be used, although the *^t^*Bu ester did not give good results under the standard reaction conditions (entries 10–12). Rhodium, base and Zn(OTf)_2_ (*vide infra*) were shown to be essential for reactivity (entries 13–16).

**Scheme 2:**
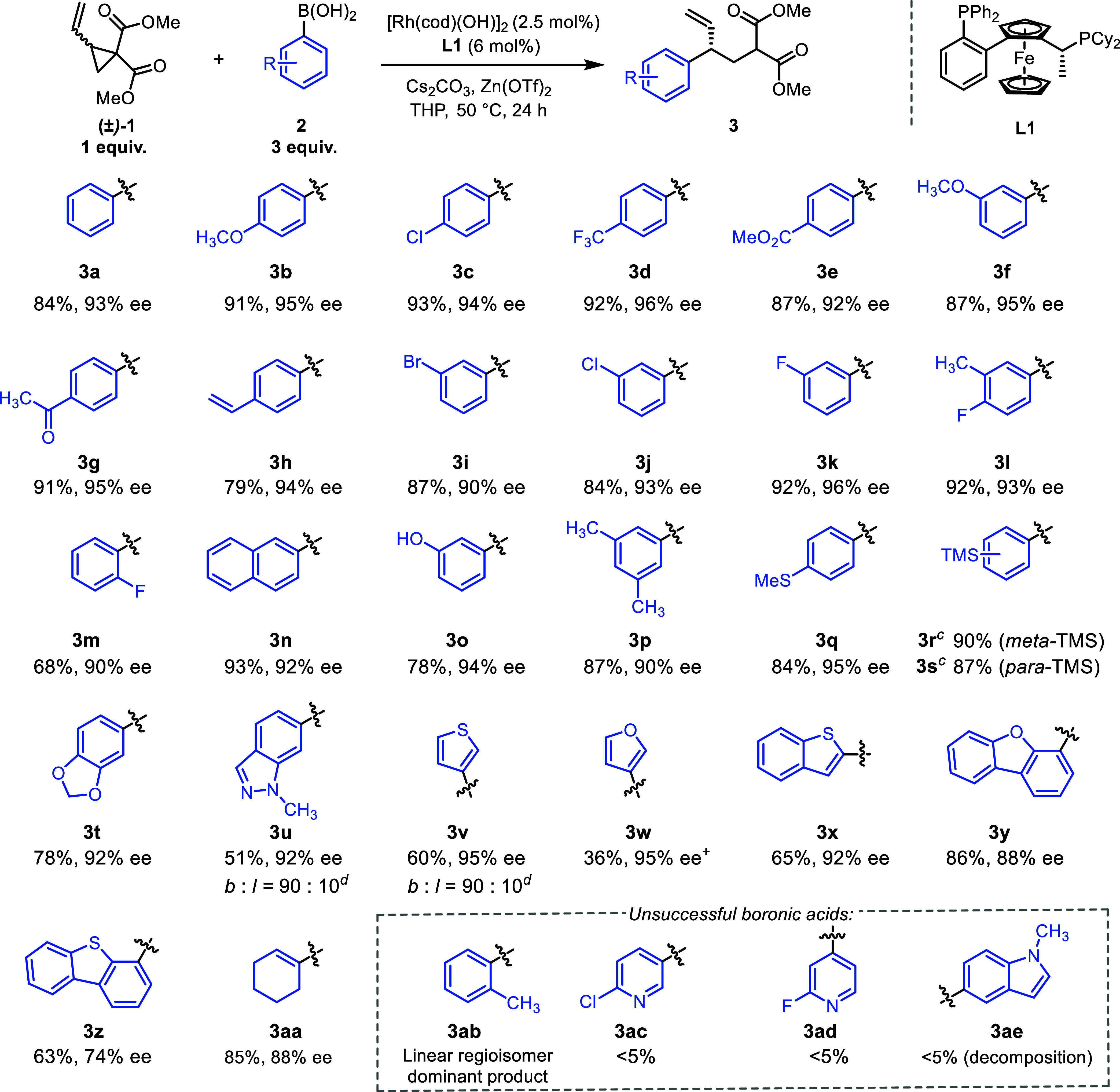
Asymmetric Ring Opening of (±)-1^,^ Experiments performed on 0.5 mmol scale. Enantiomeric ratios were determined by SFC on a chiral nonracemic stationary phase. Enantiomeric ratios unable to be determined. Branched: linear regioselectivity determined by ^1^H NMR spectroscopic analysis of the crude reaction mixture. ^+^Product was slightly (∼7%) impure. Absolute configurations were assigned by analogy to (*S*)-**11** as determined by X-ray crystallography, which was derivatized from **3a**.

Using these conditions we explored the aryl boronic acid scope (Scheme 2) and were pleased to observe excellent yields and enantioselectivities when using a range of boronic acids, with branched regioselectivity exclusively observed (unless otherwise stated). Electron donating (**3b**) and withdrawing groups (**3d**) are tolerated, giving excellent yield and enantioselectivity.

A variety of functional groups on the aryl ring could be tolerated in the reaction, including halogens (**3c**, **3i** and **3j**) and esters (**3e**). More challenging boronic acids containing functional groups such as acetyl (**3g**), vinyl (**3h**) and phenol groups (**3o**) were compatible with our reaction conditions, and both *para* and *meta* substituted boronic acids underwent the transformation smoothly. *Ortho*-substituted methyl phenylboronic acid preferentially formed the linear regioisomer (**3ab**), but less sterically hindered *ortho-*substituents (e.g., **3m**, **3y**) worked well. However, increasing the size of the *ortho* substituent led to a decrease in enantioselectivity (**3z**). A selection of electron rich heterocycles also underwent the transformation in good to modest yield including indazole (**3u**), thiophenes (**3v**), furans (**3w**) and benzothiophenes (**3x**). However, pyridines (**3ac** and **3ad**) and some indoles (**3ae**), were unsuccessful. Alkenyl boronic acids are known to be challenging coupling partners in Suzuki–Miyaura couplings because of rapid protodeborylation,^26^ however vinyl boronic acid **3aa** was able to undergo reaction with **1** in good yield and ee (85 and 88% respectively).

**Scheme 3:**
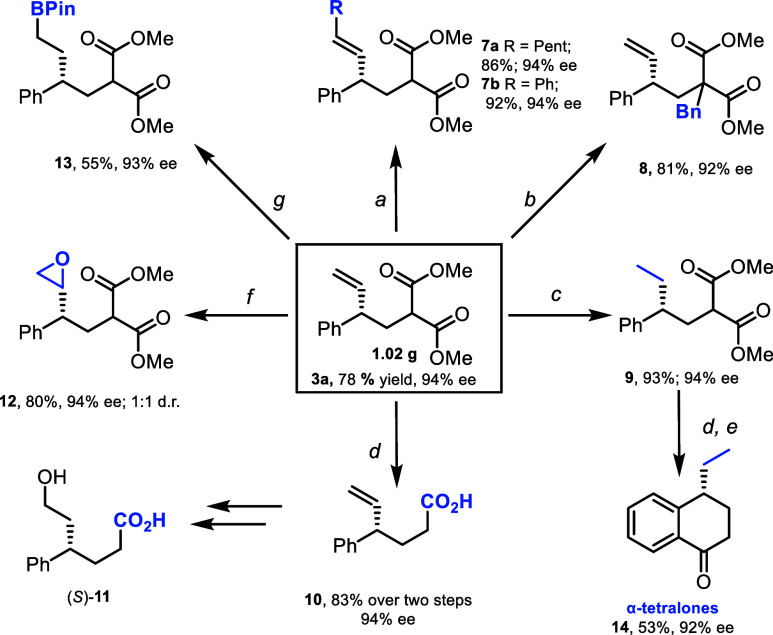
Product Derivatization Alkene, Grubbs II, DCM, 45 °C; BnCl, NaH, THF, r.t.; H_2_, [RhCl(PPh_3_)_3_], DCM, r.t.; NaOH, THF, r.t. *then* CDI, NaOH, THF; TFA, TFAA, 0 °C; m-CPBA, DCM, r.t.; HBPin, [RhCl(PPh_3_)_3_], THF, r.t.

Our protocol is robust and easily scalable, with 1.02 g of **3a** synthesized in high yield and ee (78 and 94% respectively). Next, the downstream reactivity of **3a** (Scheme 3) was investigated. **3a** contains multiple functional handles which enable a diverse range of subsequent modifications. The alkene can undergo olefin metathesis to give substituted products **7a** and **7b**. The malonate can react with electrophiles to give products such as **8**. The malonate ester groups can also be hydrolyzed and subsequently decarboxylated to give carboxylic acid **10**. **10** was functionalized through a hydroboration–oxidation sequence to give alcohol **11**, the absolute configuration of which was determined by X-ray crystallography (see SI, p S81–85). The alkene can be turned into other useful functional groups, such as epoxide **12** and BPin **13**, ready for further derivatization. We looked to employ our protocol in the synthesis of tetralones, which are found in numerous natural products and biologically active compounds.^27^**3a** can be converted to α-tetralone **14** smoothly through a decarboxylative–cyclization sequence in good yield and high ee. Our method provides a conceptually different approach to these targets and a means to prepare analogues by starting with different boronic acids, with the alkene also serving as a versatile synthetic handle. All derivatization products were formed in high yield and without erosion of ee.

**Scheme 4:**
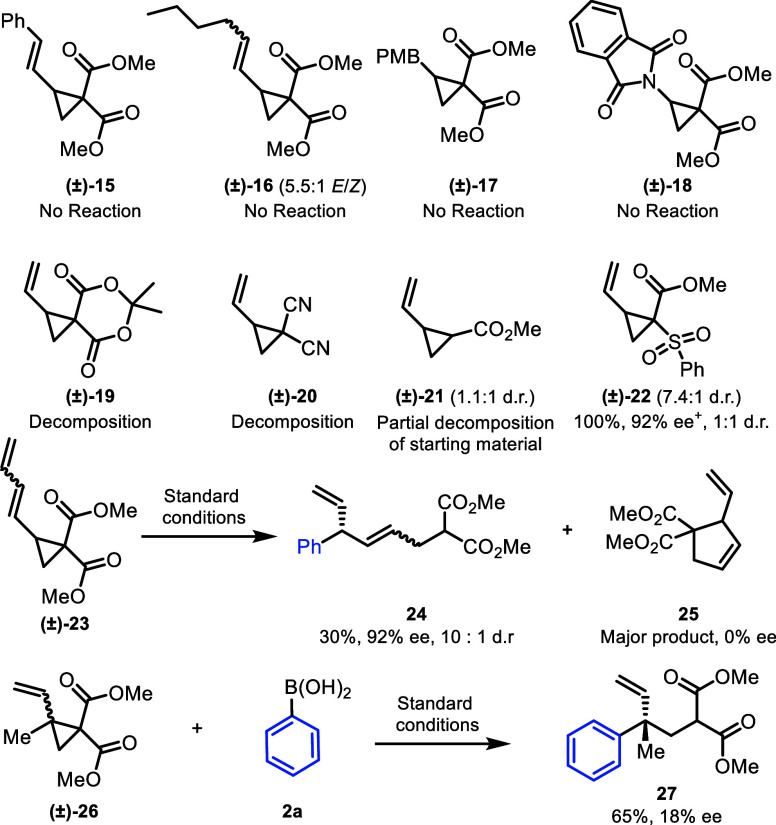
Diverse Reactivity with D–A Cyclopropanes^,^^,^ Experiments performed on 0.5 mmol scale. Enantiomeric ratios were determined by SFC using a chiral nonracemic stationary phase. *E*/*Z* and d.r. ratios determined by ^1^H NMR spectroscopic analysis of the crude reaction mixture. ^+^ee of one diastereomer

Cyclopropanes with other donor and acceptor groups were investigated (Scheme 4). Substitution at the terminal position of the alkene with aryl (**15**) and alkyl (**16**) groups resulted in no reactivity, with only starting material recovered under the standard reaction conditions (although the products that would be obtained from **15** and **16** can be formed from **3a** using metathesis, see Scheme 3). Furthermore, cyclopropanes known to undergo Friedel–Crafts alkylation such as **17** containing a *para*-methoxybenzene and **18** containing a phthalimide also failed to react.

We observed decomposition of starting material when the malonate esters were replaced with Meldrum’s acid (**19**) and malononitrile (**20**) groups. When using a derivative with one ester (**21**), no desired product was obtained, but one diastereomer of the starting material was found to decompose preferentially, so that a ∼3:1 ratio of diastereomers of starting material was recovered from an initial ∼1.1:1 mixture. However, replacing one of the ester groups with a sulfone (**22**) gave the arylated product in quantitative yield as a 1:1 mixture of diastereomers in 92% ee.

Intriguingly, we found using a terminal diene (**23**) as the donor allows reaction, but product **24** from addition to this diene is a different regioisomer than observed above, so that a chiral ε-product (Scheme 4) is observed with 92% ee. **24** was isolated in low yield, as **23** is known to rearrange.^28^ Also, we found that we could make quaternary centers by addition to cyclopropane **26**, with this quaternary center product **27** formed with complete regioselectively, although the yield (65% yield) and enantioselectivity (18% ee) still need to be improved. Attempts to optimize the formation of **24** and **27** are underway in our laboratory.

**Scheme 5:**
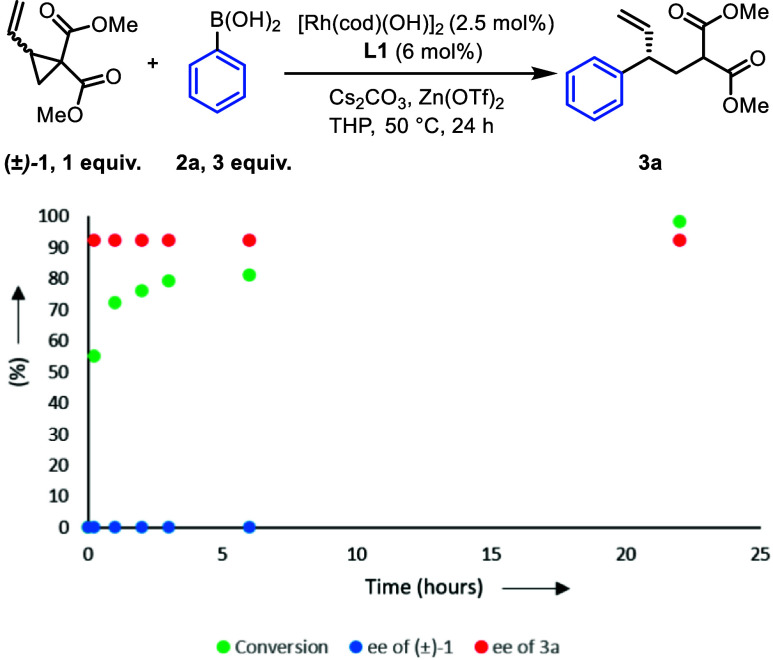
Kinetics of Ring-opening in Time

We were curious as to how the mechanism of this reaction might compare to Rh-catalyzed asymmetric additions with allyl halides,^29^ and so we monitored the enantiomeric excess of **1** and **3a** in time using standard reaction conditions (Scheme 5). While the ee of product **3a** was constant, we were surprised to see the ee of **1** was also constant, with **1** remaining racemic throughout the reaction. This suggests that either both enantiomers of **1** undergo oxidative addition at comparable rates, or the starting material racemizes during the reaction, possibly through reversible ring-opening/closing.

**Scheme 6:**
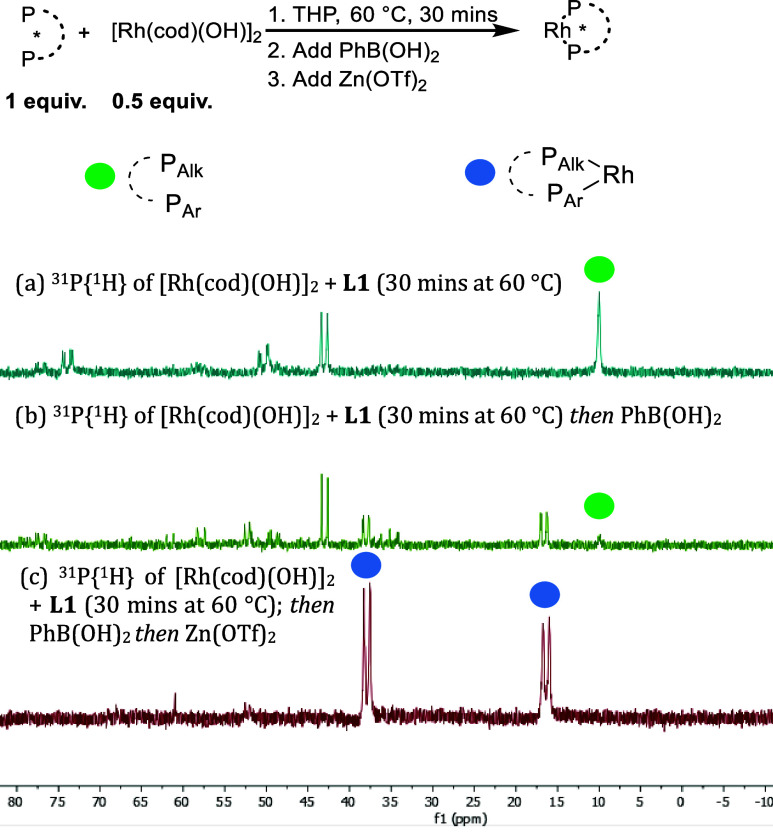
Effect of Zn(OTf)_2_ on Catalyst Mixture Catalyst components are soluble under the conditions used.

We have found compelling evidence to suggest Zn(OTf)_2_ facilitates ligand binding to rhodium. It is worth noting that none of our prior studies used **L1** or other non-C2 symmetrical bisphosphine ligands.^15−22^^31^P{^1^H} NMR spectroscopy studies on a mixture of [Rh(cod)(OH)]_2_ and **L1** showed formation of bidentate rhodium-ligand species was incomplete and slow, with a large amount of uncoordinated **L1** alongside a mixture of unknown species after 30 min at 60 °C (Scheme 6a), and even after 3 h only small amounts of bidentate rhodium species were present (see SI, p S60–62). Addition of PhB(OH)_2_ to the mixture failed to facilitate smooth ligand coordination (Scheme 6b). However, addition of Zn(OTf)_2_ dramatically simplified the NMR spectra to give virtually a single bidentate ligand-rhodium species (Scheme 6c).

**Scheme 7:**
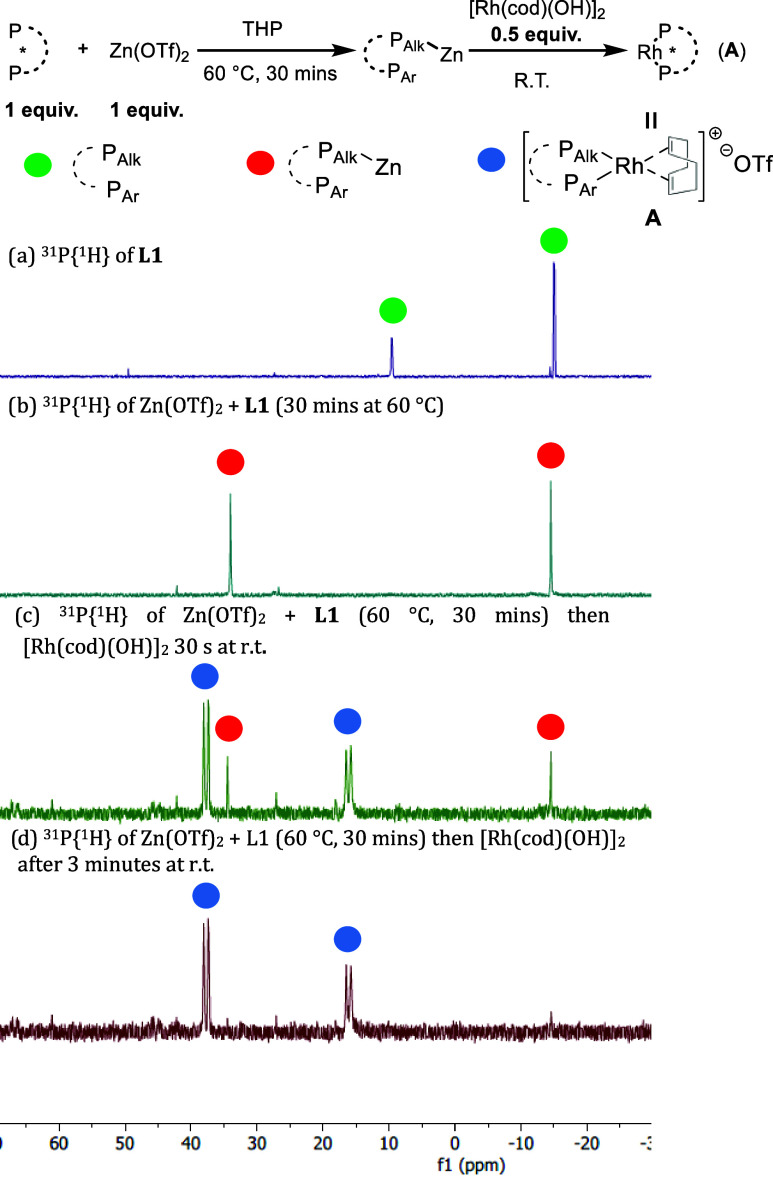
Zn(OTf)_2_ Promoted Coordination Catalyst components are soluble under the conditions used.

Further investigation saw clean formation of a bidentate Rh-ligand complex achieved through monocoordination of **L1** to zinc (Scheme 7b), followed by rapid zinc to rhodium exchange at room temperature (Scheme 7c). Within 3 min, full conversion to a bidentate rhodium species was observed (blue dots in Scheme 7d).

We fully characterized this Rh-ligand complex (**A**) formed from [Rh(cod)(OH)]_2_, Zn(OTf)_2_ and **L1** in *d*_8_-THF by NMR spectroscopy, and were able to identity it as shown in Scheme 7. In **A**, **L1** is coordinated to Rh in a bidentate manner, with a rapidly exchanging 1,5-cyclooctadiene also bound to Rh, and a triflate counterion (see SI, p S67–69 for full details).

Previously, we removed Zn(OTf)_2_ from the standard reaction conditions which resulted in low conversion, with product **3a** isolated in only 6% yield, and 93% ee (Table 1, entry 16 and Table 2, entry 2), along with unreacted starting material. We were curious as to how Zn(OTf)_2_ aided complex formation and so tested alternative additives with triflate and zinc components. We found using La(OTf)_3_ (70%, 92% ee, entry 3) and ZnBr_2_ (68%, 90% ee, entry 4) both gave comparable results and (see SI, p S65) promote formation of bidentate rhodium species.

**Table 2:** Comparison of Reaction Components

entry	deviation from standard conditions	yield **3a** (%)	ee of **3a** (%)
1	none	84	93
2	without Zn(OTf)_2_	6	93
3	La(OTf)_3_ instead of Zn(OTf)_2_	70	92
4	ZnBr_2_ instead of Zn(OTf)_2_	68	90
5	using [Rh(C_2_H_4_)_2_Cl]_2_ instead of [Rh(cod)(OH)_2_]	86	92
6	using [Rh(C_2_H_4_)_2_Cl]_2_ instead of [Rh(cod)(OH)]_2_ and without Zn(OTf)_2_	43	94

We also tested to see whether the beneficial effect of Zn(OTf)_2_ is observed with other rhodium precatalysts. When using **L1**, Zn(OTf)_2_ improved complex formation with [Rh(cod)(OMe)]_2_ but not with [Rh(coe)_2_Cl]_2_ or [Rh(C_2_H_4_)_2_Cl]_2_ (see SI, p S70–72).

**Scheme 8:**
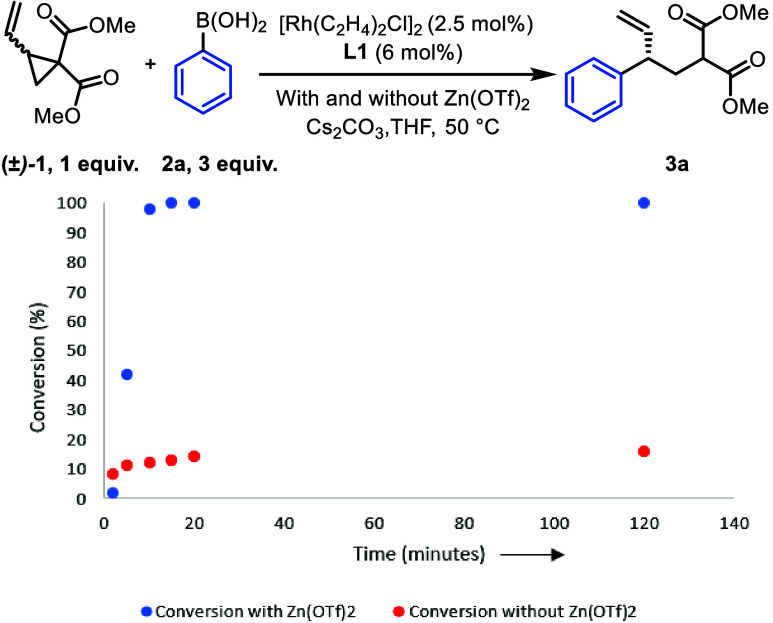
Rate Dependence on Zn(OTf)_2_

Coordination of **L1** with [Rh(C_2_H_4_)_2_Cl]_2_ proceeded smoothly in the absence of a Lewis acid salt, allowing us to test the idea that Zn(OTf)_2_ may enhance the reaction in ways other than simply facilitating the formation of an active Rh-species.^30,31^ We found [Rh(C_2_H_4_)_2_Cl]_2_ gave different results (Table 2, entries 5 and 6) with (86%, 92% ee) and without (43%, 94% ee) the addition of Zn(OTf)_2_. Reaction kinetics using [Rh(C_2_H_4_)_2_Cl]_2_ with and without Zn(OTf)_2_ (Scheme 8) show the reaction rate depends on the presence of Zn(OTf)_2_. The reaction with Zn(OTf)_2_ is initially slow, but after an induction period of a few minutes, the reaction rapidly goes to completion, with full conversion in ∼15 min. Without Zn(OTf)_2_ the reaction is initially fast, but stalls after about 10% conversion, so the reaction slows significantly.

As a comparison, we chose to examine a similar experiment using (*S*)-BINAP as the ligand in combination with [Rh(cod)(OH)]_2_ (see SI, p S75–76). In the BINAP experiment, we again observed a rate dependence on the presence of Zn(OTf)_2_ (cf. Scheme 8). With Zn(OTf)_2_ the reaction again was initially slow (6% conversion after ∼5 min), but the rate then increased so that full conversion was achieved after 2 h (2:1 regioselectivity, 70% ee). Again, the reaction without Zn(OTf)_2_ was initially fast (∼17% conversion after 5 min) with the reaction then slowing significantly so that after 2 h the conversion was only ∼50% (∼70% ee).

These results suggest that a system using [Rh(C_2_H_4_)_2_Cl]_2_ and Zn(OTf)_2_ should allow us to reduce the catalyst loading. We found that when using 50 mol % Zn(OTf)_2_, we could use 0.25 mol % of [Rh(C_2_H_4_)_2_Cl]_2_ and 0.6 mol % of **L1** to obtain **3a** in 80% yield and 94% ee (see SI, p S80).

We have often observed that formation of bidentate Rh-complexes can be surprisingly low yielding and poorly selective,^29^ but have only optimized complex formation when performing reactions on larger scales where using much lower catalyst loading is essential.^32^ The demonstration that Zn(OTf)_2_ and other Lewis acid salts can rapidly promote formation of otherwise kinetically unfavorable metal-complexes may be of use to chemists developing new catalytic reactions, and suggests that it should be considered as a standard additive to explore while screening new reaction conditions, particularly where the metal–ligand interactions are not well understood.

**Scheme 9:**
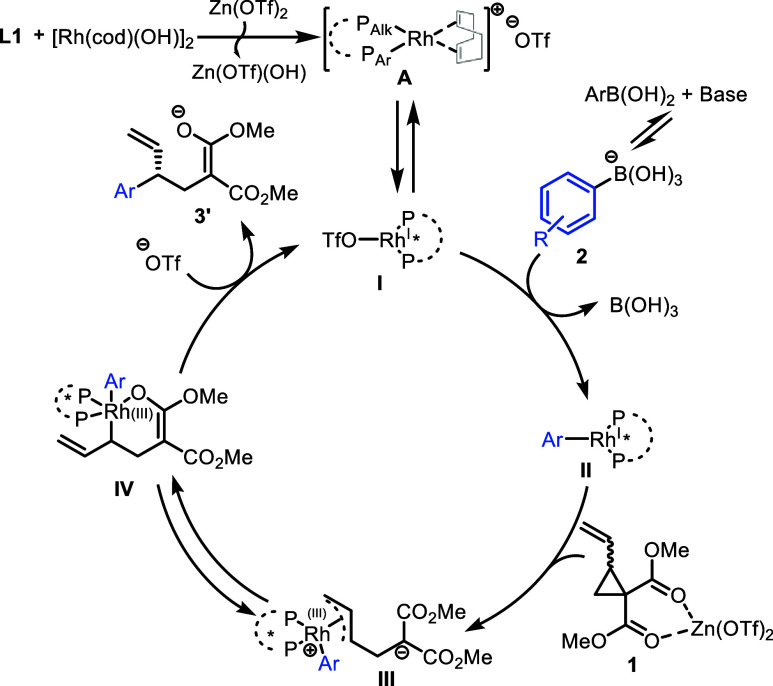
Proposed Mechanism

We propose the following preliminary mechanism based on our observations (Scheme 9). Zn(OTf)_2_ promoted coordination of **L1** to [Rh(cod)(OH)]_2_ allows for the formation of complex **I**. Following base-assisted transmetalation with aryl boronic acid **2** to give **II**, oxidative addition gives Rh(III) π-allyl intermediate **III**. The branched regioselectivity of the reaction may be aided by coordination of the malonate oxygen to give 6-membered intermediate **IV**,^33^ which upon reductive elimination generates product **3**′ and regenerates active catalyst **I**.

In summary, we have developed a Rh-catalyzed regio- and enantioselective ring opening of vinyl cyclopropanes with boronic acid nucleophiles. The use of nonsymmetrical ferrocene based bisphosphine ligands was necessary in order to get satisfactory control of selectivity. Preliminary mechanistic studies suggest the Zn(OTf)_2_ additive has a significant role in the reaction, facilitating both formation of the rhodium-ligand complex and promoting the actual reaction. The products have a range of functional groups which can be derivatized, which is highlighted in the synthesis of α-tetralone **14**, and alcohol **11**, which was used to determine the absolute configuration through X-ray crystallography. We envisage this approach can be expanded in a number of ways to be useful in synthesis. Our results also suggest that Zn(OTf)_2_ should be considered as an additive when new transition-metal-catalyzed reactions are developed by screening mixtures of ligands and metals.

## Supporting Information Available

Experimental procedures, compound synthesis and characterization data, and supporting discussion (PDF)

## Abbreviations

D-A: donor–acceptor cyclopropane
VCP: vinyl cyclopropane
