# Supplementary material for: Rh(I)-Catalyzed Regio- and Enantioselective Ring Opening of Vinyl Cyclopropanes
Source: J Am Chem Soc. 2024 Aug 20;146(35):24708–15. doi: 10.1021/jacs.4c09490 (PMC11378301; doi:10.1021/jacs.4c09490)
Supplement: Supplementary file 1 — ja4c09490_si_001.pdf [file ja4c09490_si_001.pdf]

Supporting Information

Rh(I)-Catalyzed Regio- and Enantioselective Ring Opening of Vinyl Cyclopropanes

Stephen J. Webster, László B. Balázs, F. Wieland Goetzke, Violeta Stojalnikova, Ke Liu, Kirsten E. Christensen, Harold W. Mackenzie and Stephen P. Fletcher*

Chemistry Research Laboratory, University of Oxford, 12 Mansfield Road, Oxford OX1 3TA, United Kingdom

1. Table of Contents

2. General Methods 3
3. Rh-Catalyzed Ring Openings 5
4. Procedures for Product Derivatization 42
5. Procedures for the Synthesis of Starting Materials 53
6. Reactivity of Donor-Acceptor Cyclopropanes 59
7. Mechanistic Studies 60
   7.1 Determining the structure of the Rh-ligand complex 67
   7.2 Comparison of L1 complexation with other rhodium pre-catalysts 70
   7.3 Kinetic studies in the presence and absence of $Zn(OTf)_2$ 73
   7.4 Procedures from Mechanistic Studies 77
8. X-ray crystallographic analysis 81
9. NMR Spectra 86
10. SFC Traces 129
11. References 160

2. General Methods

All reactions were carried out under argon atmosphere and anhydrous environment in flame-dried glassware and anhydrous solvent, with continuous magnetic stirring unless otherwise stated. Heating was performed using DrySyn heating blocks.

Nuclear Magnetic Resonance (NMR) spectroscopy was measured at room temperature. ^{1}H NMR, ^{13}C NMR experiments were carried out using Bruker AVIII HD 400 (400/101 MHz) spectrometers. Chemical shifts (δ) are reported in parts per million (ppm) relative to the residual solvent peak with corresponding coupling constants (J) in Hertz (Hz) and multiplicities (s: singlet, d: doublet, t: triplet, q: quartet, m: multiplet and combinations of these and app.: apparent multiplicities). Rhodium-ligand complex NMR experiments were carried out at 298K on a Bruker AVIIIHD spectrometer equipped with a 11.7 T magnet (^{1}H: 500 MHz, ^{13}C: 126 MHz, ^{19}F: 471 MHz, ^{31}P: 203 MHz) and a room temperature TBO probe.

Optical rotations ($[\alpha]_{25}^{D}$) were recorded using a Schmidt Haensch Unipol L2000 polarimeter in a cell with a path length of 1 dm (using the sodium D line, 589 nm). Concentrations (c) are reported in g/100 mL. Temperatures are reported in °C.

Infrared (IR, neat or thin film): spectroscopy was carried out on a Bruker Tensor 27 FT–IR spectrometer with an internal calibration range of 4000–600 cm^{-1}. The samples are reported as absorption maxima in cm^{-1}.

Chiral SFC (supercritical fluid chromatography) separations were conducted on a Waters Acquity UPC2 system using Waters Empower software. Chiralpak® columns (150x3 mm, particle size 3 μm) were used as specified in the text. Solvents used were of HPLC grade (Fisher Scientific, Sigma Aldrich or Rathburn).

High Resolution Mass spectra were carried out by internal service at the University of Oxford. (1) Electron spray ionisation (ESI+) was recorded on a Fisons Platform II. (2) Electron ionization

(EI)/Chemical ionisation (CI): Analyses were performed on an Agilent 7200 quadrupole time of flight (Q-ToF) instrument equipped with a direct insertion probe supplied by Scientific instrument Manufacturer (SIM) GmbH. (3) Atmospheric pressure chemical ionisation ($APCI^+$): Analyses were performed using a Thermo Exactive mass spectrometer equipped with Waters Acquity liquid chromatography system.

Commercially available reagents and ligands were purchased from Sigma Aldrich, Alfa Aesar, Acros Organics, Fluorochem and Strem Chemicals and unless otherwise stated were used without further purification. $[Rh(cod)OH]_2$ and $[Rh(C_2H_4)_2Cl]_2$ was bought from Sigma Aldrich; Walphos-SL-W003-1 was purchased from Strem Chemicals or provided by Solvias.

Dry solvents were collected freshly from an mBraun SPS–800 solvent purification system after having passed through anhydrous alumina columns, or purchased from Sigma Aldrich. Deuterated solvents were purchased from Sigma Aldrich.

3. Rh-Catalyzed Ring Openings

General Procedure A:

$[Rh(cod)OH]_2$ (5.7 mg, 0.0125 mmol, 0.025 eq) and Walphos-SL-W003-1 (20.1 mg, 0.03 mmol, 0.06 eq) were added to a flame dried 5 mL round bottom flask, sealed with a rubber septum under an argon atmosphere and dissolved in THP (0.3 mL). This solution was stirred at 60 °C (the solution turned dark brown). After 30 min, this catalyst solution was added to a flame dried 5 mL round bottom flask containing boronic acid (1.5 mmol, 3.0 eq), dimethyl 2-vinylcyclopropane-1,1-dicarboxylate **1** (92.1, 0.5 mmol, 1.0 eq), Cs_2CO_3 (162.9 mg, 0.5 mmol, 1 eq) and $Zn(OTf)_2$ (36.4 mg, 0.1 mmol, 0.20 eq) via syringe, and the flask rinsed with THP (0.2 mL) and added. The resulting dark brown reaction mixture was stirred at 50 °C for 16-24 h.

The reaction mixture was cooled to room temperature and Et_2O added. The reaction mixture was filtered through a plug of silica and washed with Et_2O (x3) and the filtrate collected and concentrated *in vacuo*. The crude product was purified by using silica gel chromatography (eluted with the indicated solvent mixture) to afford the desired products.

Racemates:

$[Rh(cod)OH]_2$ (9.1 mg, 0.020 mmol, 0.05 eq) and *rac*-BINAP (29.9 mg, 0.048 mmol, 0.12 eq), were added to a flame dried 5 mL round bottom flask, sealed with a rubber septum under an argon atmosphere, dissolved in THF (0.70 mL) and stirred at 60 °C. After 30 min, this solution was added to a solution of boronic acid (1.20 mmol, 3.0 eq), dimethyl 2-vinylcyclopropane-1,1-dicarboxylate **1** (73.7 mg, 0.40 mmol, 1.0 eq), Cs_2CO_3 (130 mg, 0.4 mmol, 1 eq) and $Zn(OTf)_2$ (29.1 mg, 0.08 mmol, 0.20 eq) in THF (0.7 mL) via syringe, and the flask rinsed with THF (0.2 mL) and added and the resulting mixture was stirred at 60 °C overnight.

The reaction mixture was cooled to room temperature and Et_2O added. The reaction mixture was filtered through a plug of silica and washed with Et_2O (x3) and the filtrate collected and concentrated *in vacuo*. The crude product was purified by using silica gel chromatography (eluted with the indicated solvent mixture) to afford the desired products.

Upscale:

Larger-scale experiment was performed on 5 mmol scale in direct analogy to the **General Procedure A** in a 25 ml round-bottom flask.

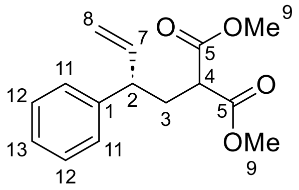

(+)-Dimethyl (*S*)-2-(2-phenylbut-3-en-1-yl)malonate 3a

The corresponding compound was prepared following **General Procedure A** using vinyl cyclopropane **1** and phenylboronic acid **2a**. Purification by silica gel chromatography (hexane/EtOAc = 100/0 to 80/20) afforded the product **3a** as a colourless oil (110 mg, 84%). SFC analysis showed an enantiomeric excess of 93%.

^{1}H NMR (400 MHz, $CDCl_3$): δ 7.35 – 7.27 (m, 2H; ; 2x C(12)–H), 7.25 – 7.16 (m, 3H; 2x C(11)–H, C(13)-H), 5.99 – 5.86 (m, 1H; C(7)-H), 5.10 (d, *J* = 1.1 Hz, 1H; C(8)-H), 5.07 (dt, *J* = 6.7, 1.3 Hz, 1H; C(8')-H), 3.74 (s, 3H; C(9)H3), 3.69 (s, 3H; C(9')H3), 3.35 (t, *J* = 7.4 Hz, 1H; C(4)-H), 3.29 (q, *J* = 7.8 Hz, 1H; C(2)-H), 2.41 – 2.25 (m, 2H; 2x C(3)-H).

^{13}C NMR (101 MHz, $CDCl_3$) δ 169.9 (C=O), 169.8 (C=O), 142.7 (C(1)), 140.6 (C(7)), 128.8 (C(12)), 127.7 (C(11)), 126.9 (C(13)), 115.5 (C(8)), 52.6 (C(9)), 49.9 (C(4)), 47.7 (C(2)), 34.3 (C(3)).

IR ($CHCl_3$ film): 2954, 2918, 2849, 1752, 1736, 1494 cm^{-1}.

HRMS (ESI): m/z calcd for $C_{15}H_{18}O_4Na^+$ $[M + Na]^+$ 285.1097 found 285.1096.

SFC: Chiralpak® IG, 1500 psi, 30 °C; flow: 1.5 mL/min; 1% to 30% MeOH over 5 min, then from 30% to 50% MeOH in 0.5 min, then hold 50% MeOH for 1.5 min; 96.4:3.6 er (major enantiomer t_R = 1.71 min, minor enantiomer t_R = 1.56 min).

$[\alpha]^{25}_D$ = +12.6 (c = 1.0, $CHCl_3$).

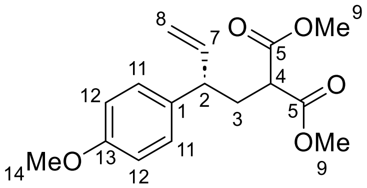

(+)-Dimethyl (S)-2-(2-(4-methoxyphenyl)but-3-en-1-yl)malonate 3b

The corresponding compound was prepared following **General Procedure A** using vinyl cyclopropane **1** and 4-methoxyphenylboronic acid **2b**. Purification by silica gel chromatography (hexane/EtOAc = 100/0 to 75/25) afforded the product **3b** as a colourless oil (133 mg, 91%). SFC analysis showed an enantiomeric excess of 95%.

^{1}H NMR (400 MHz, $CDCl_3$) δ 7.14 – 7.05 (m, 2H; 2x C(11)–H), 6.93 – 6.81 (m, 2H; 2x C(12)-H), 5.89 (ddd, *J* = 16.8, 10.5, 7.6 Hz, 1H; C(7)-H), 5.09 – 5.06 (m, 1H; C(8)-H), 5.04 (dt, *J* = 8.8, 1.3 Hz, 1H; C(8')-H), 3.79 (s, 3H; C(14)H3), 3.74 (s, 3H; C(9)H3), 3.69 (s, 3H; C(9')H3), 3.33 (t, *J* = 7.4 Hz, 1H; C(4)-H), 3.24 (q, *J* = 7.7 Hz, 1H; C(2)-H), 2.38 – 2.20 (m, 2H; 2x C(3)-H).

^{13}C NMR (101 MHz, $CDCl_3$) δ 170.0 (C=O), 169.9 (C=O), 158.5 (C(13)), 141.0 (C(7), 134.7 (C(1)), 128.7 (C(11)), 115.1 (C(8)), 114.2 (C(12)), 55.4 (C(14)), 52.6 (C(9)), 49.9 (C(4)), 46.8 (C(2)), 34.4 (C(3)).

IR ($CHCl_3$ film): 2981, 2889, 2838, 1752 1735, 1633, 1611, 1583, 1512 cm^{-1}

HRMS (ESI): m/z calcd for $C_{16}H_{20}O_5Na^+$ $[M + Na]^+$ 315.1203 found 315.1202.

SFC: Chiralpak® IG, 1500 psi, 30 °C; flow: 1.5 mL/min; 1% to 30% MeOH over 5 min, then from 30% to 50% MeOH in 0.5 min, then hold 50% MeOH for 1.5 min; 97.4:2.6 er (major enantiomer t_R = 2.49 min, minor enantiomer t_R = 2.18 min).

$[\alpha]^{25}_D$ = +10.2 (c = 1.0, $CHCl_3$).

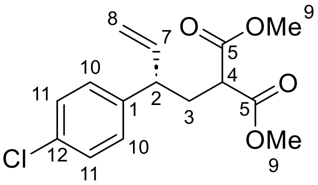

(+)-Dimethyl (*S*)-2-(2-(4-chlorophenyl)but-3-en-1-yl)malonate 3c

The corresponding compound was prepared following **General Procedure A** using vinyl cyclopropane **1** and 4-Cl-phenylboronic acid **2c**. Purification by silica gel chromatography (hexane/EtOAc = 100/0 to 75/25) afforded the product **3c** as a colourless oil (139 mg, 93%). SFC analysis showed an enantiomeric excess of 94% .

^{1}H NMR (400 MHz, $CDCl_3$) δ 7.32 – 7.23 (m, 2H; 2x C(10)-H), 7.16 – 7.07 (m, 2H; 2x C(11)-H), 5.93 – 5.80 (m, 1H; C(7)-H), 5.14 – 5.02 (m, 2H; 2x C(8)-H), 3.73 (s, 3H C(9)H3), 3.69 (s, 3H; C(9')H3), 3.36 – 3.22 (m, 2H C(4)-H and C(2)-H), 2.38 – 2.20 (m, 2H; 2x C(3)-H).

^{13}C NMR (101 MHz, $CDCl_3$) δ 169.8 (C=O), 169.7 (C=O), 141.1 (C(1)), 140.1 (C(7)), 132.6 (C(12)) , 129.1 (C(11)), 128.9 (C(10)), 116.0 (C(8)), 52.72 (C(9)), 52.70 (C(9')), 49.8 (C(4)), 47.0 (C(2)), 34.2 (C(3)).

IR ($CHCl_3$ film): 3082, 2974, 2923, 1735, 1637, 1492 cm^{-1}.

HRMS (ESI): m/z calcd for $C_{15}H_{17}O_4ClNa^+$ $[M + Na]^+$ 319.0708 found 319.0703.

SFC: Chiralpak® IG, 1500 psi, 30 °C; flow: 1.5 mL/min; 1% to 30% MeOH over 5 min, then from 30% to 50% MeOH in 0.5 min, then hold 50% MeOH for 1.5 min; 96.8:3.2 er (major enantiomer t_R = 2.12 min, minor enantiomer t_R = 1.87 min).

$[\alpha]^{25}_D$ = +8.6 (c = 1.0, $CHCl_3$).

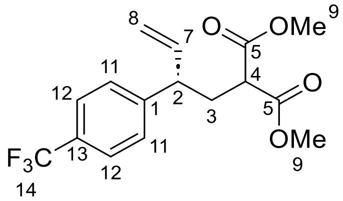

(+)-Dimethyl (*S*)-2-(2-(4-(trifluoromethyl)phenyl)but-3-en-1-yl)malonate 3d

The corresponding compound was prepared following **General Procedure A** using vinyl cyclopropane **1** and 4-CF_3-phenylboronic acid **2d**. Purification by silica gel chromatography (hexane/EtOAc = 100/0 to 80/20) afforded the product **3d** as a colourless oil (152 mg, 92%). SFC analysis showed an enantiomeric excess of 96%.

^{1}H NMR (400 MHz, $CDCl_3$) δ 7.60 – 7.54 (m, 2H; 2x C(12)–H), 7.31 (d, *J* = 8.0 Hz, 2H; 2x C(11)–H), 5.89 (ddd, *J* = 17.1, 10.3, 7.7 Hz, 1H; C(7)-H), 5.18 – 5.05 (m, 2H; 2x C(8)-H), 3.74 (s, 3H; C(9)H3), 3.69 (s, 3H; C(9')H3), 3.42 – 3.29 (m, 2H; C(2)-H and C(4)-H), 2.42 – 2.25 (m, 2H; 2x C(3)-H).

^{13}C NMR (101 MHz, $CDCl_3$) δ 169.7 (C=O), 169.6 (C=O), 146.8 (C(1)), 139.6 (C(7)), 129.5 (q, *J* = 32.6 Hz; C(13)), 128.2 – 128.0 (m; C(11)), 125.7 (q, *J* = 3.8 Hz' C(12)), 124.3 (q, *J* = 271.8; C(14)), 116.5 (C(8)), 52.74 (C(9)) , 52.70 (C(9')), 49.8 C(4)), 47.5 (C(2)), 34.1 (C(3)).

^{19}F NMR (376 MHz, $CDCl_3$) δ -62.48.

IR ($CHCl_3$ film): 2981, 2889, 1753, 1736, 1618 cm^{-1}.

HRMS (ESI): m/z calcd for $C_{16}H_{17}F_3O_4Na^+$ $[M + Na]^+$ 353.0971 found 353.0968.

SFC: Chiralpak® IG, 1500 psi, 30 °C; flow: 1.5 mL/min; 1% to 30% MeOH over 5 min, then from 30% to 50% MeOH in 0.5 min, then hold 50% MeOH for 1.5 min; 97.7:2.3 er (major enantiomer t_R = 1.36 min, minor enantiomer t_R = 1.21 min).

$[\alpha]^{25}_D$ = +11.5 (c = 1.0, $CHCl_3$).

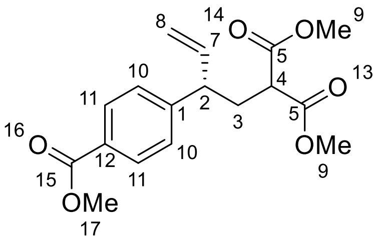

(+)-Dimethyl (*S*)-2-(2-(4-(methoxycarbonyl)phenyl)but-3-en-1-yl)malonate 3e

The corresponding compound was prepared following **General Procedure A** using vinyl cyclopropane **1** and (4-(methoxycarbonyl)phenyl)boronic acid **2e**. Purification by silica gel chromatography (hexane/EtOAc = 95/5 to 70/30) afforded the product **3e** as a colourless oil (139 mg, 87%). SFC analysis showed an enantiomeric excess of 92%.

^{1}H NMR (400 MHz, $CDCl_3$) δ 8.02 – 7.94 (m, 2H; 2x C(11)-H), 7.30 – 7.22 (m, 2H; 2x C(10)-H), 5.89 (ddd, *J* = 17.0, 10.3, 7.8 Hz, 1H; C(7)-H), 5.16 – 5.04 (m, 2H; 2x C(8)-H), 3.90 (s, 3H; C(17)H3), 3.73 (s, 3H; C(9)H3), 3.69 (s, 3H; C(9')H3), 3.41 – 3.28 (m, 2H; C(2)-H and C(4)-H), 2.41 – 2.25 (m, 2H; 2x C(3)-H).

^{13}C NMR (101 MHz, $CDCl_3$) δ 169.7 (C=O), 169.6 (C=O), 167.0 (C=O), 148.0 (C(1)), 139.7 (C(7)), 130.1 (C(11)), 128.8 (C(12)), 127.8 (C(10)), 116.4 (C(8)), 52.73 (C(9)), 52.71 (C(9')), 52.2 (C(17)), 49.8 (C(4)), 47.7 (C(2)), 34.1 (C(3)).

IR ($CHCl_3$ film): 3023, 3015, 2964, 1752, 1723, 1509, 1436 cm^{-1}.

HRMS (ESI): m/z calcd for $C_{17}H_{20}O_6Na^+$ $[M + Na]^+$ 343.1152 found 343.1153.

SFC: Chiralpak® IG, 1500 psi, 30 °C; flow: 1.5 mL/min; 1% to 30% MeOH over 5 min, then from 30% to 50% MeOH in 0.5 min, then hold 50% MeOH for 1.5 min; 96.0:4.0 er (major enantiomer t_R = 4.53 min, minor enantiomer t_R = 3.64 min).

$[\alpha]^{25}_D$ = +16.7 (c = 1.0, $CHCl_3$).

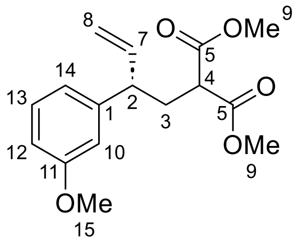

(+)-Dimethyl (*S*)-2-(2-(3-methoxyphenyl)but-3-en-1-yl)malonate 3f

The corresponding compound was prepared following **General Procedure A** using vinyl cyclopropane **1** and (3-(methoxycarbonyl)phenyl)boronic acid **2f**. Purification by silica gel chromatography (hexane/EtOAc = 100/0 to 75/25) afforded the product **3f** as a colourless oil (128 mg, 87%). SFC analysis showed an enantiomeric excess of 95%.

^{1}H NMR (400 MHz, $CDCl_3$) δ 7.23 (t, *J* = 7.8 Hz, 1H; C(13)-H), 6.81 – 6.70 (m, 3H; 3 x CAr-H), 5.97 – 5.84 (m, 1H; C(7)-H), 5.10 (dt, *J* = 3.9, 1.3 Hz, 1H; C(8)-H), 5.07 (dt, *J* = 3.4, 1.3 Hz, 1H; C(8')-H), 3.79 (s, 3H; C(15)H3), 3.74 (s, 3H; C(9)H3), 3.69 (s, 3H; C(9)H3), 3.35 (t, *J* = 7.4 Hz, 1H; C(4)-H), 3.26 (q, *J* = 7.8 Hz, 1H; C(2)-H), 2.39 – 2.23 (m, 2H; 2x C(3)-H).

^{13}C NMR (101 MHz, $CDCl_3$) δ 169.9 (C=O), 169.8 (C=O), 159.95 (C(11)), 144.3 (C(1)), 140.5 (C(7)) , 129.8 (C(13)), 120.0 (C(14)), 115.6 (C(8)), 113.6 (C(12)), 112.1 (C(10)), 55.3 (C(15)), 52.6 (C(9)), 49.9 (C(4)), 47.7 (C(2)), 34.2 (C(3)).

IR ($CHCl_3$ film): 3007, 2954, 1751, 1735, 1600, 1585, 1436 cm^{-1}.

HRMS (ESI): m/z calcd for $C_{16}H_{20}O_5Na^+$ $[M + Na]^+$ 315.1203 found 315.1204.

SFC: Chiralpak® IG, 1500 psi, 30 °C; flow: 1.5 mL/min; 1% to 30% MeOH over 5 min, then from 30% to 50% MeOH in 0.5 min, then hold 50% MeOH for 1.5 min; 97.6:2.4 er (major enantiomer t_R = 2.20 min, minor enantiomer t_R = 2.05 min).

$[\alpha]^{25}_D$ = +9.1 (c = 1.0, $CHCl_3$).

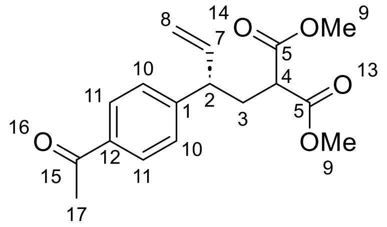

(+)-Dimethyl (*S*)-2-(2-(4-acetylphenyl)but-3-en-1-yl)malonate 3g

The corresponding compound was prepared following **General Procedure A** using vinyl cyclopropane **1** and (4-acetylphenyl)boronic acid **2g**. Purification by silica gel chromatography (hexane/EtOAc = 95/5 to 70/30) afforded the product **3g** as a colourless oil (139 mg, 91%). SFC analysis showed an enantiomeric excess of 95%.

^{1}H NMR (400 MHz, $CDCl_3$) δ 7.95 – 7.87 (m, 2H; 2x C(11)-H), 7.33 – 7.27 (m, 2H; 2x C(10)-H), 5.89 (ddd, *J* = 17.1, 10.3, 7.8 Hz, 1H; C(7)-H), 5.17 – 5.05 (m, 2H; 2x C(8)-H), 3.74 (s, 3H; C(9)H3), 3.70 (s, 3H; C(9')H3), 3.34 (dt, *J* = 11.4, 7.6 Hz, 2H; C(2)-H and C(4)-H), 2.58 (s, 3H; C(17)H3), 2.42 – 2.25 (m, 2H; 2x C(3)-H).

^{13}C NMR (101 MHz, $CDCl_3$) δ 197.8 (C=O), 169.7 (C=O), 169.6 (C=O), 148.3 (C(1)), 139.7 (C(7)), 136.0 ((C12)), 129.0 (C(11)), 128.0 (C(10)), 116.4 (C(8)), 52.75 (C(9)), 52.74 (C(9')), 49.8 (C(4)), 47.7 (C(2)), 34.1 (C(3)), 26.7 (C(17)).

IR ($CHCl_3$ film): 3004, 2922, 1751, 1734, 1683, 1639, 1606, 1571, 1435 cm^{-1}.

HRMS (ESI): m/z calcd for $C_{16}H_{20}O_5Na^+$ [M + Na]+ 327.1203 found 327.1201.

SFC: Chiralpak® IG, 1500 psi, 30 °C; flow: 1.5 mL/min; 1% to 30% MeOH over 5 min, then from 30% to 50% MeOH in 0.5 min, then hold 50% MeOH for 1.5 min; 97.6:2.4 er (major enantiomer t_R = 3.93 min, minor enantiomer t_R = 3.43 min).

$[\alpha]^{25}_D$ = +18.4 (c = 1.0, $CHCl_3$).

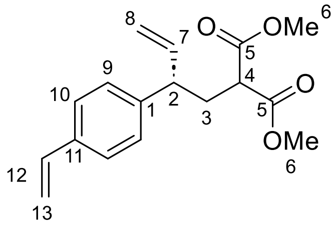

(+)-Dimethyl (*S*)-2-(2-(4-vinylphenyl)but-3-en-1-yl)malonate 3h

The corresponding compound was prepared following **General Procedure A** using vinyl cyclopropane **1** and (4-vinylphenyl)boronic acid **2h**. Purification by silica gel chromatography (hexane/EtOAc = 100/0 to 80/20) afforded the product **3h** as a colourless oil (114 mg, 79%). SFC analysis showed an enantiomeric excess of 94%.

^{1}H NMR (400 MHz, $CDCl_3$) δ 7.40 – 7.31 (m, 2H; 2 x C(10)-H), 7.18 – 7.09 (m, 2H; 2 x C(9)-H), 6.69 (dd, *J* = 17.6, 10.9 Hz, 1H; C(12)-H), 5.97 – 5.84 (m, 1H; C(7)-H), 5.72 (dd, *J* = 17.6, 1.0 Hz, 1H; C(13)-H), 5.22 (dd, *J* = 10.9, 0.9 Hz, 1H; C(13')-H), 5.10 (d, *J* = 1.1 Hz, 1H; C(8)-H), 5.06 (dt, *J* = 7.7, 1.3 Hz, 1H; C(8')-H), 3.74 (s, 3H; C(6)H3), 3.68 (s, 3H; C(6')H3), 3.34 (t, *J* = 7.4 Hz, 1H; C(4)-H), 3.28 (q, *J* = 7.8 Hz, 1H; C(2)-H), 2.40 – 2.24 (m, 2H; C(3)-H).

^{13}C NMR (101 MHz, $CDCl_3$) δ 169.9 (C=O), 169.8 (C=O), 142.3 (C(1)), 140.5 (C(7)), 136.6 (C(12)), 136.3 (C(11)), 127.9 (C(9)), 126.7 (C(10)), 115.6 (C(8)), 113.7 (C(13)), 52.7 (C(6)), 49.9 (C(4)), 47.4 (C(2)), 34.2 (C(3)).

IR ($CHCl_3$ film): 3022, 2918, 1754, 1737, 1691, 1483 cm^{-1}.

HRMS (ESI): m/z calcd for $C_{17}H_{20}O_4Na^+$ [M + Na]+ 311.1254 found 311.1255.

SFC: Chiralpak® IG, 1500 psi, 30 °C; flow: 1.5 mL/min; 1% to 30% MeOH over 5 min, then from 30% to 50% MeOH in 0.5 min, then hold 50% MeOH for 1.5 min; 97.2:2.8 er (major enantiomer t_R = 2.40 min, minor enantiomer t_R = 2.16 min).

$[\alpha]^{25}_D$ = +13.6 (c = 1.0, $CHCl_3$).

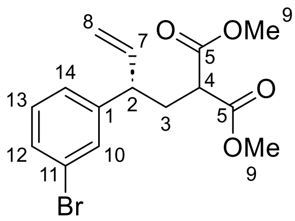

(+)-Dimethyl (*S*)-2-(2-(3-bromophenyl)but-3-en-1-yl)malonate 3i

The corresponding compound was prepared following **General Procedure A** using vinyl cyclopropane **1** and (3-bromophenyl)boronic acid **2i**. Purification by silica gel chromatography (hexane/EtOAc = 100/0 to 75/25) afforded the product **3i** as a pale yellow oil (148 mg, 87%). SFC analysis showed an enantiomeric excess of 90%.

^{1}H NMR (400 MHz, $CDCl_3$) δ 7.40 – 7.30 (m, 2H; 2 x CAr-H), 7.18 (td, *J* = 7.7, 0.5 Hz, 1H; C(13)-H), 7.14 – 7.09 (m, 1H; C(14)-H), 5.86 (ddd, *J* = 17.1, 10.3, 7.8 Hz, 1H; C(7)-H), 5.16 – 5.04 (m, 2H; 2x C(8)-H), 3.74 (s, 3H; C(9)H3), 3.70 (s, 3H; C(9')H3), 3.34 (t, *J* = 7.4 Hz, 1H; C(4)-H), 3.26 (q, *J* = 7.8 Hz, 1H; C(2)-H), 2.38 – 2.22 (m, 2H; 2x C(3)-H).

^{13}C NMR (101 MHz, $CDCl_3$) δ 169.8 (C=O), 169.6 (C=O), 145.1 (C(1)), 139.8 (C(7)), 130.8 (C(12)), 130.4 (C(13)), 130.0 (C(10)), 126.4 (C(14)), 122.9 (C(11)), 116.3 (C(8)), 52.74 (C(9)), 52.72 (C(9')), 49.8 (C(4)), 47.4 (C(2)), 34.1 (C(3)).

IR ($CHCl_3$ film): 3021, 2953, 1751, 1735, 1638, 1593, 1568, 1475 cm^{-1}.

HRMS (ESI): m/z calcd for $C_{15}H_{17}BrO_4Na^+$ $[M + Na]^+$ 363.0202, 365.0182 found 363.0201, 365.0179.

SFC: Chiralpak® IG, 1500 psi, 30 °C; flow: 1.5 mL/min; 1% to 30% MeOH over 5 min, then from 30% to 50% MeOH in 0.5 min, then hold 50% MeOH for 1.5 min; 95.1:4.9 er (major enantiomer t_R = 1.88 min, minor enantiomer t_R = 1.77 min).

$[\alpha]^{25}_D$ = +9.0 (c = 1.0, $CHCl_3$).

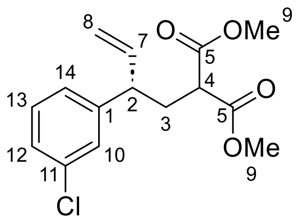

(+)-Dimethyl (*S*)-2-(2-(3-chlorophenyl)but-3-en-1-yl)malonate 3j

The corresponding compound was prepared following **General Procedure A** using vinyl cyclopropane **1** and (3-chlorophenyl)boronic acid **2j**. Purification by silica gel chromatography (hexane/EtOAc = 100/0 to 75/25) afforded the product **3j** as a pale yellow oil (125 mg, 84%). SFC analysis showed an enantiomeric excess of 93%.

^{1}H NMR (400 MHz, $CDCl_3$) δ 7.28 – 7.13 (m, 3H; 3 x CAr-H), 7.07 (dt, *J* = 7.4, 1.6 Hz, 1H; C(13)-H), 5.87 (ddd, *J* = 17.0, 10.3, 7.8 Hz, 1H; C(7)-H), 5.14 – 5.05 (m, 2H; 2x C(8)-H), 3.74 (s, 3H; C(9)H3), 3.70 (s, 3H; C(9')H3), 3.34 (t, *J* = 7.4 Hz, 1H; C(4)-H), 3.27 (q, *J* = 7.8 Hz, 1H; C(2)-H), 2.38 – 2.22 (m, 2H; 2x C(3)-H).

^{13}C NMR (101 MHz, $CDCl_3$) δ 169.8 (C=O), 169.6 (C=O), 144.8 (C(1)), 139.8 (C(7)), 134.6 (C(11)), 130.1 (C(13)), 127.9 (C(12)), 127.1 (C(10)), 125.9 (C(14)), 116.3 (C(8)), 52.73 (C(9)), 52.71 (C(9')), 49.8 (C(4)), 47.4 (C(2)), 34.1 (C(3)).

IR ($CHCl_3$ film): 2973, 2953, 1752, 1735, 1573, 1530, 1478, 1435 cm^{-1}.

HRMS (ESI): m/z calcd for $C_{15}H_{17}ClO_4$ $[M + Na]^+$ 319.0708 found 319.0707.

SFC: Chiralpak® IG, 1500 psi, 30 °C; flow: 1.5 mL/min; 1% to 30% MeOH over 5 min, then from 30% to 50% MeOH in 0.5 min, then hold 50% MeOH for 1.5 min; 96.5:3.5 er (major enantiomer t_R = 1.69 min, minor enantiomer t_R = 1.58 min).

$[\alpha]^{25}_D$ = +10.5 (c = 1.0, $CHCl_3$).

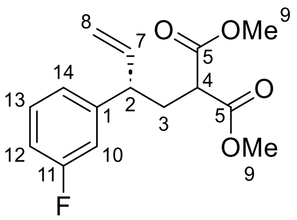

(+)-Dimethyl (*S*)-2-(2-(3-fluorophenyl)but-3-en-1-yl)malonate 3k

The corresponding compound was prepared following **General Procedure A** using vinyl cyclopropane **1** and (3-fluorophenyl)boronic acid **2k**. Purification by silica gel chromatography (hexane/EtOAc = 100/0 to 80/20) afforded the product **3k** as a colourless oil (129 mg, 92%). SFC analysis showed an enantiomeric excess of 96%.

^{1}H NMR (400 MHz, $CDCl_3$) δ 7.32 – 7.22 (m, 1H; C(13)-H), 6.97 (dt, *J* = 7.6, 1.4 Hz, 1H; C(14)-H), 6.94 – 6.87 (m, 2H; 2x CAr-H), 5.88 (ddd, *J* = 17.0, 10.3, 7.7 Hz, 1H; C(7)-H), 5.15 – 5.04 (m, 2H; 2x C(8)-H), 3.74 (s, 3H; C(9)H3), 3.70 (s, 3H; C(9')H3), 3.38 – 3.24 (m, 2H; C(2)-H and C(4)-H), 2.38 – 2.22 (m, 2H; 2x C(3)-H).

^{13}C NMR (101 MHz, $CDCl_3$) δ 169.8 (C=O), 169.7 (C=O), 163.2 (d, *J* = 246.0 Hz; C(11)), 145.3 (d, *J* = 6.8 Hz; C(1)), 139.9 (C(7)), 130.3 (d, *J* = 8.3 Hz; C(13)), 123.4 (d, *J* = 2.8 Hz; C(14)), 116.1 (C(8)), 114.6 (d, *J* = 21.4 Hz; C(12)), 113.8 (d, *J* = 21.0 Hz; C(10)), 52.72 (C(9)), 52.71 (C(9')), 49.8 (C(4), 47.4 (d, *J* = 1.8 Hz; C(2)), 34.1 (C(3)).

^{19}F NMR (376 MHz, $CDCl_3$) δ -112.89 (td, *J* = 9.4, 6.0 Hz).

IR ($CHCl_3$ film): 3027, 2954, 1752, 1735, 1639, 1613, 1589, 1488, 1437 cm^{-1}.

HRMS (ESI): m/z calcd for $C_{15}H_{17}FO_4$ $[M + Na]^+$ 303.1003 found 303.1000.

SFC: Chiralpak® IG, 1500 psi, 30 °C; flow: 1.5 mL/min; 1% to 30% MeOH over 5 min, then from 30% to 50% MeOH in 0.5 min, then hold 50% MeOH for 1.5 min; 97.7:2.3 er (major enantiomer t_R = 1.56 min, minor enantiomer t_R = 1.42 min).

$[\alpha]^{25}_D$ = +12.9 (c = 1.0, $CHCl_3$).

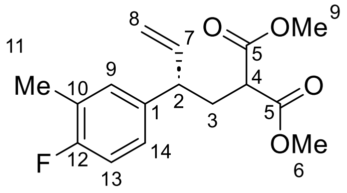

(+)-Dimethyl (*S*)-2-(2-(4-fluoro-3-methylphenyl)but-3-en-1-yl)malonate 3l

The corresponding compound was prepared following **General Procedure A** using vinyl cyclopropane **1** and (4-fluoro-3-methylphenyl)boronic acid **2l**. Purification by silica gel chromatography (hexane/EtOAc = 100/0 to 80/20) afforded the product **3l** as a pale yellow oil (135 mg, 92%). SFC analysis showed an enantiomeric excess of 93%.

^{1}H NMR (400 MHz, $CDCl_3$) δ 7.02 – 6.88 (m, 3H; 3 x CAr-H), 5.87 (ddd, *J* = 17.0, 10.3, 7.7 Hz, 1H; C(7)-H), 5.12 – 5.01 (m, 2H; 2 x C(8)), 3.74 (s, 3H; C(6)H3), 3.70 (s, 3H; C(6')H3), 3.33 (t, *J* = 7.4 Hz, 1H; C(4)-H), 3.28 – 3.17 (m, 1H; C(2)-H), 2.36 – 2.19 (m, 5H; 2 x C(3)-H and C(11)H3).

^{13}C NMR (101 MHz, $CDCl_3$) δ 169.90 (C=O), 169.8 (C=O), 160.3 (d, *J* = 243.6 Hz; C(12)), 140.6 (C(7)), 138.1 (d, *J* = 3.6 Hz; C(1)), 130.7 (d, *J* = 5.0 Hz; C(9)), 126.3 (d, *J* = 7.9 Hz; C(14)), 125.0 (d, *J* = 17.4 Hz; C(10)), 115.5 (C(8)), 115.2 (d, *J* = 22.3 Hz; C(13)), 52.7 (C(6)), 49.8 (C(4)), 46.9 (C(2)), 34.4 (C(3)), 14.7 (d, *J* = 3.6 Hz; C(11)).

^{19}F NMR (376 MHz, $CDCl_3$) δ -120.57 – -120.74 (m).

IR ($CHCl_3$ film): 3036, 2919, 1753, 1736, 1503, 1462 cm^{-1}.

HRMS (ESI): m/z calcd for $C_{16}H_{19}FO_4Na^+$ $[M + Na]^+$ 317.1160 found 317.1155.

SFC: Chiralpak® IG, 1500 psi, 30 °C; flow: 1.5 mL/min; 1% to 30% MeOH over 5 min, then from 30% to 50% MeOH in 0.5 min, then hold 50% MeOH for 1.5 min; 96.5:3.5 er (major enantiomer t_R = 1.30 min, minor enantiomer t_R = 1.25 min).

$[\alpha]^{25}_D$ = +9.3 (c = 1.0, $CHCl_3$).

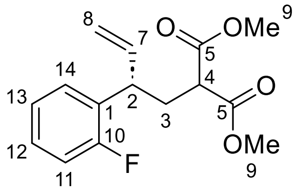

(+)-Dimethyl (*S*)-2-(2-(2-fluorophenyl)but-3-en-1-yl)malonate 3m

The corresponding compound was prepared following **General Procedure A** using vinyl cyclopropane **1** and (2-fluorophenyl)boronic acid **2m**. Purification by silica gel chromatography (hexane/EtOAc = 95/5 to 80/20) afforded the product **3m** as a pale yellow oil (95 mg, 68%). SFC analysis showed an enantiomeric excess of 90%.

^{1}H NMR (400 MHz, $CDCl_3$) δ 7.23 – 7.15 (m, 2H; 2 x CAr-H), 7.14 – 7.07 (m, 1H; C(13)-H), 7.06 – 6.97 (m, 1H; C(11)-H), 6.00 – 5.87 (m, 1H; C(7)-H), 5.12 (dt, *J* = 2.3, 0.8 Hz, 1H; C(8)-H), 5.10 – 5.05 (m, 1H; C(8')-H), 3.73 (s, 3H; C(9)H3), 3.68 (s, 3H; C(9)H3), 3.63 (q, *J* = 7.9 Hz, 1H; C(2)-H), 3.34 (t, *J* = 7.4 Hz, 1H; C(4)-H), 2.46 – 2.24 (m, 2H; C(3)-H).

^{13}C NMR (101 MHz, $CDCl_3$) δ 169.76 (C=O), 169.71 (C=O), 160.8 (d, *J* = 245.9 Hz; C(10)), 139.1 C(7)), 129.4 (d, *J* = 14.4 Hz; C(1)), 128.9 (d, *J* = 4.7 Hz; C(12)), 128.4 (d, *J* = 8.4 Hz; C(14)), 124.5 (d, *J* = 3.6 Hz; C(13)), 116.2 (C(8)), 115.8 (d, *J* = 22.6 Hz; C(11)), 52.7 (C(9)), 49.9 (C(4)), 41.0 (d, *J* = 1.9 Hz; (C(2)), 33.3 (d, *J* = 1.5 Hz; C(3)).

^{19}F NMR (377 MHz, $CDCl_3$) δ -117.89 (dt, *J* = 11.9, 6.2 Hz).

IR ($CHCl_3$ film): 3018, 2955, 2919, 1754, 1737, 1530, 1513, 1492 cm^{-1}.

HRMS (ESI): m/z calcd for $C_{15}H_{17}FO_4Na^+$ $[M + Na]^+$ 281.1184 found 281.1182.

SFC: Chiralpak® IG, 1500 psi, 30 °C; flow: 1.5 mL/min; 1% to 30% MeOH over 5 min, then from 30% to 50% MeOH in 0.5 min, then hold 50% MeOH for 1.5 min; 94.9:5.1 er (major enantiomer t_R = 1.54 min, minor enantiomer t_R = 1.40 min).

$[\alpha]^{25}_D$ = +16.3 (c = 1.0, $CHCl_3$).

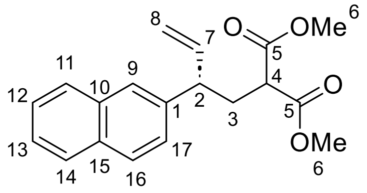

(+)-Dimethyl (*S*)-2-(2-(naphthalen-2-yl)but-3-en-1-yl)malonate 3n

The corresponding compound was prepared following **General Procedure A** using vinyl cyclopropane **1** and naphthalen-2-ylboronic acid **2n**. Purification by silica gel chromatography (hexane/EtOAc = 100/0 to 80/20) afforded the product **3n** as a pale yellow oil (146 mg, 93%). SFC analysis showed an enantiomeric excess of 92%.

^{1}H NMR (400 MHz, $CDCl_3$) δ 7.85 – 7.75 (m, 3H; 3 x CAr-H), 7.63 (d, *J* = 1.7 Hz, 1H; 1 x CAr-H), 7.51 – 7.40 (m, 2H; 2 x CAr-H), 7.33 (dd, *J* = 8.5, 1.8 Hz, 1H; 1 x CAr-H), 6.01 (ddd, *J* = 17.5, 10.0, 7.6 Hz, 1H; C(7)-H), 5.15 (dt, *J* = 3.7, 1.4 Hz, 1H; C(8)-H), 5.12 (dt, *J* = 3.8, 1.3 Hz, 1H; C(8')-H), 3.75 (s, 3H; C(6)H3), 3.66 (s, 3H; C(6')H3), 3.47 (q, *J* = 7.7 Hz, 1H; C(2)-H), 3.38 (t, *J* = 7.4 Hz, 1H; C(4)-H), 2.43 (t, *J* = 7.5 Hz, 2H; 2 x C(3)-H).

^{13}C NMR (101 MHz, $CDCl_3$) δ 170.0 (C=O), 169.9 (C=O), 140.6 (C(7)), 140.0 (C(1)), 133.7 (C(10)), 132.6 (C(15)), 128.5 (C(Ar)), 127.8 (C(Ar)), 127.7 (C(Ar)), 126.3 (C(Ar)), 126.2 (C(Ar)), 126.1 (C(Ar)), 125.8 (C(Ar)), 115.8 (C(8)), 52.7 C(6)), 52.6 (C(6')), 49.9 C(4)), 47.7 (C(2)), 34.2 (C(3)).

IR ($CHCl_3$ film): 2950, 2919, 2849,1751, 1734, 1508, 1435 cm^{-1}.

HRMS (ESI): m/z calcd for $C_{19}H_{20}O_4Na^+$ $[M + Na]^+$ 335.1254 found 335.1252.

SFC: Chiralpak® IG, 1500 psi, 30 °C; flow: 1.5 mL/min; 1% to 30% MeOH over 5 min, then from 30% to 50% MeOH in 0.5 min, then hold 50% MeOH for 1.5 min; 96.3:3.7 er (major enantiomer t_R = 2.83 min, minor enantiomer t_R = 2.64 min).

$[\alpha]^{25}_D$ = +15.3 (c = 1.0, $CHCl_3$).

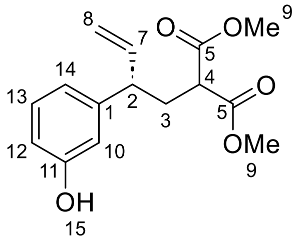

(+)-Dimethyl (*S*)-2-(2-(3-hydroxyphenyl)but-3-en-1-yl)malonate 3o

The corresponding compound was prepared following **General Procedure A** using vinyl cyclopropane **1** and (3-hydroxyphenyl)boronic acid **2o**. Purification by silica gel chromatography (hexane/EtOAc = 95/5 to 70/30) afforded the product **3o** as a colourless oil (109 mg, 78%). SFC analysis showed an enantiomeric excess of 94%.

^{1}H NMR (400 MHz, $CDCl_3$) δ 7.17 (t, *J* = 7.8 Hz, 1H; C(12)-H), 6.75 (dt, *J* = 7.6, 1.2 Hz, 1H; C(14)-H), 6.73 – 6.65 (m, 2H; 2 x CAr-H), 5.95 – 5.82 (m, 1H; C(7)-H), 5.10 (d, *J* = 1.1 Hz, 1H; C(8)-H), 5.06 (dt, *J* = 6.7, 1.3 Hz, 1H; C(8')-H), 5.01 (br. s, 1H; O-H), 3.74 (s, 3H C(9)H3), 3.70 (s, 3H C(9')H3), 3.36 (t, *J* = 7.4 Hz, 1H; C(4)-H), 3.23 (q, *J* = 7.8 Hz, 1H; C(2)-H), 2.38 – 2.22 (m, 2H; 2x C(3)-H).

^{13}C NMR (101 MHz, $CDCl_3$) δ 170.0 (C=O), 169.9 (C=O), 156.0 (C(11)), 144.6 (C(1)), 140.4 (C(7)), 130.0 (C(13)), 120.2 (C(14), 115.7 (C(12)), 114.6 (C(8)), 113.9 (C(10)), 52.7 (C(9)), 49.9 (C(4)), 47.5 (C(2)), 34.2 (C(3)).

IR ($CHCl_3$ film): 3435, 3003, 2955, 1734, 1637, 1600, 1590, 1487, 1454, 1437 cm^{-1}.

HRMS (ESI): m/z calcd for $C_{15}H_{18}O_5Na^+$ $[M + Na]^+$ 301.1046 found 301.1046.

SFC: Chiralpak® IG, 1500 psi, 30 °C; flow: 1.5 mL/min; 1% to 30% MeOH over 5 min, then from 30% to 50% MeOH in 0.5 min, then hold 50% MeOH for 1.5 min; 96.8:3.2 er (major enantiomer t_R = 3.27 min, minor enantiomer t_R = 3.09 min).

$[\alpha]^{25}_D$ = +8.8 (c = 1.0, $CHCl_3$).

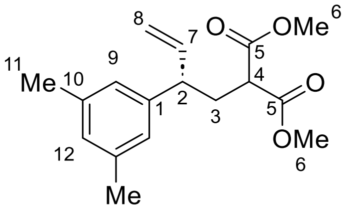

(+)-Dimethyl (*S*)-2-(2-(3,5-dimethylphenyl)but-3-en-1-yl)malonate 3p

The corresponding compound was prepared following **General Procedure A** using vinyl cyclopropane **1** and (3,5-dimethylphenyl)boronic acid **2p**. Purification by silica gel chromatography (hexane/EtOAc = 100/0 to 80/20) afforded the product **3p** as a yellow oil (126 mg, 87%). SFC analysis showed an enantiomeric excess of 90%.

^{1}H NMR (400 MHz, $CDCl_3$) δ 6.85 (tt, *J* = 1.5, 0.8 Hz, 1H; C(12)-H, 6.79 (dt, *J* = 1.8, 0.8 Hz, 2H; C(9)-H), 5.97 – 5.83 (m, 1H; C(7)-H), 5.12 – 5.07 (m, 1H; C(8)-H), 5.06 (d, *J* = 1.0 Hz, 1H; C(8')-H), 3.74 (s, 3H; C(6)H_3), 3.70 (s, 3H; C(6')H_3), 3.36 (t, *J* = 7.4 Hz, 1H; C(4)-H), 3.20 (q, *J* = 7.8 Hz, 1H; C(2)-H), 2.36 – 2.23 (m, 8H; 2 x C(3)-H and C(11)H_3).

^{13}C NMR (101 MHz, $CDCl_3$) δ 170.0 (C=O), 169.9 (C=O), 142.7 (C(1)), 140.8 (C(7)), 138.3 (C(10)), 128.5 (C(12)), 125.4 (C(9)), 115.4 (C(8)), 52.6 (C(6)), 50.0 (C(4)), 47.7 (C(2)), 34.3 (C(3)), 21.4 (C(11)).

IR ($CHCl_3$ film): 3018, 2954, 2919, 2850, 1753, 1736, 1637, 1604, 1436 cm^{-1}.

HRMS (ESI): m/z calcd for $C_{17}H_{22}O_4Na^+$ $[M + Na]^+$ 313.1410 found 313.1407.

SFC: Chiralpak® IC, 1500 psi, 30 °C; flow: 1.5 mL/min; 1% to 30% MeOH over 5 min, then from 30% to 50% MeOH in 0.5 min, then hold 50% MeOH for 1.5 min; 94.8:5.2 er (major enantiomer t_R = 1.50 min, minor enantiomer t_R = 1.55 min).

$[\alpha]^{25}_D$ = +9.9 (c = 1.0, $CHCl_3$).

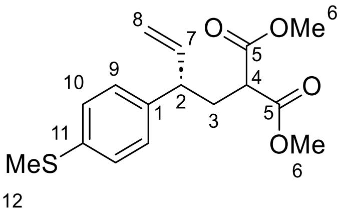

(+)-Dimethyl (*S*)-2-(2-(4-(methylthio)phenyl)but-3-en-1-yl)malonate 3q

The corresponding compound was prepared following **General Procedure A** using vinyl cyclopropane **1** and (4-(methylthio)phenyl)boronic acid **2q**. Purification by silica gel chromatography (hexane/EtOAc = 100/0 to 80/20) afforded the product **3q** as a pale yellow oil (130 mg, 84%). SFC analysis showed an enantiomeric excess of 95%.

^{1}H NMR (400 MHz, $CDCl_3$) δ 7.25 – 7.17 (m, 2H; 2 x C(9)-H, 7.14 – 7.06 (m, 2H; 2 x C(10)-H), 5.88 (ddd, *J* = 16.9, 10.4, 7.6 Hz, 1H; C(7)-H), 5.09 (dt, *J* = 2.7, 1.2 Hz, 1H; C(8)-H), 5.05 (dt, *J* = 10.5, 1.3 Hz, 1H; C(8')-H), 3.73 (s, 3H; C(6)H_3), 3.69 (s, 3H; C(6')H_3), 3.33 (t, *J* = 7.4 Hz, 1H; C(4)-H), 3.25 (q, *J* = 7.7 Hz, 1H; C(2)-H), 2.46 (s, 3H; C(12)H_3), 2.38 – 2.21 (m, 2H; 2 x C(3)-H).

^{13}C NMR (101 MHz, $CDCl_3$) δ 169.9 (C=O), 169.8 (C=O), 140.5 (C(7)), 139.6 (C(1)), 136.7 (C(11)), 128.3 (C(10)), 127.3 (C(9)), 115.6 (C(8)), 52.7 (C(6)), 49.8 (C(4)), 47.1 (C(2)), 34.2 (C(3)), 16.2 (C(12)).

IR ($CHCl_3$ film): 2982, 2954, 2920, 1752, 1735, 1638, 1599, 1495, 1436 cm^{-1}.

HRMS (ESI): m/z calcd for $C_{16}H_{20}O_4SNa^+$ $[M + Na]^+$ 331.0975 found 331.0977.

SFC: Chiralpak® IG, 1500 psi, 30 °C; flow: 1.5 mL/min; 1% to 30% MeOH over 5 min, then from 30% to 50% MeOH in 0.5 min, then hold 50% MeOH for 1.5 min; 97.4:2.6 er (major enantiomer t_R = 3.27 min, minor enantiomer t_R = 2.92 min).

$[\alpha]^{25}_D$ = +12.7 (c = 1.0, $CHCl_3$).

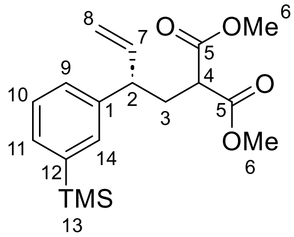

(+)-Dimethyl (*S*)-2-(2-(3-(trimethylsilyl)phenyl)but-3-en-1-yl)malonate 3r

The corresponding compound was prepared following **General Procedure A** using vinyl cyclopropane **1** and (3-(trimethylsilyl)phenyl)boronic acid **2r**. Purification by silica gel chromatography (hexane/EtOAc = 100/0 to 80/20) afforded the product **3r** as a pale yellow oil (151 mg, 90%). Enantiomers inseperable by SFC.

^{1}H NMR (400 MHz, $CDCl_3$) δ 7.38 (dt, *J* = 7.4, 1.2 Hz, 1H; C_{Ar}-H), 7.33 – 7.27 (m, 2H; 2 x C_{Ar}-H), 7.18 (dt, *J* = 7.8, 1.7 Hz, 1H; C_{Ar}-H), 6.00 – 5.87 (m, 1H; C(7)-H), 5.11 (dt, *J* = 5.9, 1.4 Hz, 1H; C(8)-H), 5.08 (d, *J* = 1.1 Hz, 1H; C(8')-H), 3.74 (s, 3H; C(6)H_3), 3.69 (s, 3H; C(6')H_3), 3.35 (t, *J* = 7.4 Hz, 1H; C(4)-H), 3.29 (q, *J* = 7.8 Hz, 1H; C(2)-H), 2.42 – 2.25 (m, 2H; 2 x C(3)-H), 0.26 (s, 9H; 3 x C(13)H_3).

^{13}C NMR (101 MHz, $CDCl_3$) δ 170.0 (C=O), 169.9 (C=O), 141.8 (C(1)), 141.1 (C(7)), 140.7 (C(12)), 132.8 (C_{Ar}), 131.9 (C_{Ar}), 128.2 (C_{Ar}), 128.0 (C_{Ar}), 115.5 (C(8)), 52.6 (C(6)), 49.9 (C(5)), 47.9 (C(2)), 34.4 (C(3)), -0.97 (C(13)).

IR ($CHCl_3$ film): 2955, 1754, 1737, 1637, 1436 cm^{-1}.

HRMS (ESI): m/z calcd for $C_{18}H_{26}O_4SiNa^+$ $[M + Na]^+$ 357.1493 found 357.1490.

$[\alpha]^{25}_D$ = +10.6 (c = 1.0, $CHCl_3$).

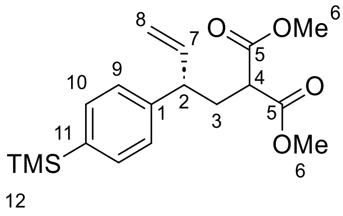

(+)-Dimethyl (S)-2-(2-(4-(trimethylsilyl)phenyl)but-3-en-1-yl)malonate 3s

The corresponding compound was prepared following **General Procedure A** using vinyl cyclopropane **1** and (4-(trimethylsilyl)phenyl)boronic acid **2s** stirred at 60 °C. Purification by silica gel chromatography (hexane/EtOAc = 100/0 to 80/20) afforded the product **3s** as a colourless oil (147 mg, 87%). Enantiomers inseperable by SFC analysis.

^{1}H NMR (400 MHz, $CDCl_3$) δ 7.51 – 7.41 (m, 2H; 2 x C(10)-H), 7.21 – 7.14 (m, 2H; 2 x C(9)-H), 5.98 – 5.85 (m, 1H; C(7)-H), 5.10 (dt, *J* = 6.0, 1.3 Hz, 1H; C(8)-H), 5.07 (d, *J* = 1.0 Hz, 1H; C(8')-H), 3.74 (s, 3H; C(6)H_3), 3.69 (s, 3H; C(6')H_3), 3.36 (t, *J* = 7.4 Hz, 1H; C(4)-H), 3.27 (q, *J* = 7.8 Hz, 1H; C(2)-H), 2.33 (td, *J* = 7.6, 1.7 Hz, 2H; 2 x C(3)-H), 0.25 (s, 9H; 3 x C(12)-H_3).

^{13}C NMR (101 MHz, $CDCl_3$) δ 169.9 (C=O), 169.8 (C=O), 143.3 (C(1)), 140.6 (C(7)), 138.8 (C(11), 133.9 (C(10)), 127.1 (C(9)), 115.6 (C(8)), 52.7 (C(6)), 52.6 (C(6')), 49.9 (C(4)), 47.7 (C(2)), 34.2 (C(3)), -0.97 (C(12)).

IR ($CHCl_3$ film): 3068, 3012, 2954, 2898, 2849, 1754, 1737, 1638, 1599, 1436 cm^{-1}.

HRMS (ESI): m/z calcd for $C_{18}H_{26}O_4SiNa^+$ $[M + Na]^+$ 357.1493 found 357.1491.

$[\alpha]^{25}_{D}$ = +11.6 (c = 1.0, $CHCl_3$).

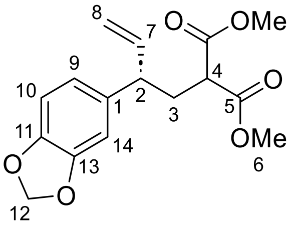

(+)-Dimethyl (*S*)-2-(2-(benzo[d][1,3]dioxol-5-yl)but-3-en-1-yl)malonate 3t

The corresponding compound was prepared following **General Procedure A** using vinyl cyclopropane **1** and benzo[d][1,3]dioxol-5-ylboronic acid **2t**. Purification by silica gel chromatography (hexane/EtOAc = 100/0 to 80/20) afforded the product **3t** as a colourless oil (120 mg, 78%). SFC analysis showed an enantiomeric excess of 92%.

^{1}H NMR (400 MHz, $CDCl_3$) δ 6.74 (d, *J* = 7.9 Hz, 1H; C(10)-H), 6.67 (d, *J* = 1.8 Hz, 1H; C(14)-H), 6.62 (dd, *J* = 8.0, 1.7 Hz, 1H; C9)-H), 5.93 (s, 2H; C(12)-H), 5.87 (ddd, *J* = 16.8, 10.6, 7.6 Hz, 1H; C(7)-H), 5.08 (t, *J* = 1.0 Hz, 1H; C(8)-H), 5.05 (dt, *J* = 8.6, 1.3 Hz, 1H; C(8')-H), 3.74 (s, 3H; C(9)H3), 3.70 (s, 3H; C(9')H3), 3.33 (dd, *J* = 7.9, 6.9 Hz, 1H; C(4)-H), 3.21 (q, *J* = 7.7 Hz, 1H; C(2)-H), 2.36 – 2.17 (m, 2H; C(3)-H).

^{13}C NMR (101 MHz, $CDCl_3$) δ 169.91 (C=O), 169.85 (C=O), 148.0 (C(13)), 146.4 (C(11)), 140.7 (C(7)), 136.5 (C(1)), 120.8 (C(9)), 115.3 (C(8)), 108.5 (C(10)), 108.0 (C(14)), 101.1 (C(12)), 52.7 (C(6)), 49.8 (C(4)), 47.2 (C(2)), 34.4 (C(3)).

IR ($CHCl_3$ film): 3029, 2936, 2919, 2850, 1753, 1735, 1530, 1503, 1487 cm^{-1}.

HRMS (ESI): m/z calcd for $C_{16}H_{18}O_6Na^+$ $[M + Na]^+$ 329.0996 found 329.0995.

SFC: Chiralpak® IC, 1500 psi, 30 °C; flow: 1.5 mL/min; 1% to 30% MeOH over 5 min, then from 30% to 50% MeOH in 0.5 min, then hold 50% MeOH for 1.5 min; 96.0:4.0 er (major enantiomer t_R = 1.83 min, minor enantiomer t_R = 1.89 min).

$[\alpha]^{25}_D$ = +3.2 (c = 1.0, $CHCl_3$).

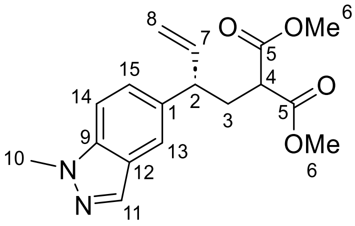

(+)-Dimethyl (*S*)-2-(2-(1-methyl-1H-indazol-5-yl)but-3-en-1-yl)malonate 3u

$[Rh(cod)OH]_2$ (5.7 mg, 0.0125 mmol, 0.025 eq) and Walphos-SL-W003-1 (20.1 mg, 0.03 mmol, 0.06 eq) were added to a flame dried 5 mL round bottom flask, sealed with a rubber septum under an argon atmosphere and dissolved in THP (0.3 mL). This solution was stirred at 60 °C (the solution turned dark brown). After 30 min, this catalyst solution was added to a flame dried 5 mL round bottom flask containing (1-methyl-1H-indazol-5-yl)boronic acid (264.0 mg, 1.5 mmol, 3.0 eq), dimethyl 2-vinylcyclopropane-1,1-dicarboxylate **1** (92.1, 0.5 mmol, 1.0 eq), Cs_2CO_3 (162.9 mg, 0.5 mmol, 1 eq) and $Zn(OTf)_2$ (36.4 mg, 0.1 mmol, 0.20 eq) via syringe, and the flask rinsed with THP (0.2 mL) and added. The resulting dark brown reaction mixture was stirred at 70 °C overnight.

The reaction mixture was cooled to room temperature and Et_2O added. The reaction mixture was filtered through a plug of silica and washed with Et_2O (x3) and the filtrate collected and concentrated *in vacuo*. The crude product was purified by using silica gel chromatography (hexane/EtOAc = 95/5 to 50/50) to afford the product **3u** as a yellow oil (81 mg, 51%). SFC analysis showed an enantiomeric excess of 92%.

^{1}H NMR (400 MHz, $CDCl_3$) δ 7.92 (d, *J* = 1.0 Hz, 1H; C_{Ar}-H), 7.35 (dt, *J* = 8.7, 1.0 Hz, 1H; C_{Ar}-H), 7.23 (dd, *J* = 8.7, 1.6 Hz, 1H; C_{Ar}-H), 6.03 – 5.90 (m, 1H; C(7)-H), 5.13 – 5.10 (m, 1H; C(8)-H), 5.08 (dt, *J* = 8.0, 1.3 Hz, 1H; C(8')-H), 4.05 (s, 3H; C(10)H_3), 3.74 (s, 3H; C(6)H_3), 3.66 (s, 3H; C(6')H_3), 3.41 (q, *J* = 7.7 Hz, 1H; C(2)-H), 3.34 (t, *J* = 7.4 Hz, 1H; C(4)-H), 2.46 – 2.29 (m, 2H; 2 x C(3)-H).

^{13}C NMR (101 MHz, $CDCl_3$) δ 169.94 (C=O), 169.87 (C=O), 141.0 (C(7)), 139.3 (C(9)), 134.8 (C(1)), 132.6 (C(11)), 126.6 (C(15)), 124.4 (C(12)), 119.5 (C(13)), 115.4 (C(8)), 109.4 (C(14)), 52.7 (C(6)), 52.6 (C(6')), 49.9 (C(4)), 47.4 (C(2)), 35.7 (C(10)), 34.5 (C(3)).

IR ($CHCl_3$ film): 2954, 2917, 2849, 1750, 1734, 1637, 1509, 1435 cm^{-1}.

HRMS (ESI): m/z calcd for $C_{17}H_{20}N_2O_4Na^+$ $[M + Na]^+$ 339.1315 found 339.1313.

SFC: Chiralpak® IG, 1500 psi, 30 °C; flow: 1.5 mL/min; 1% to 30% MeOH over 5 min, then from 30% to 50% MeOH in 0.5 min, then hold 50% MeOH for 1.5 min; 96.0:4.0 er (major enantiomer t_R = 4.16 min, minor enantiomer t_R = 3.66 min).

$[\alpha]^{25}_D$ = +4.7 (c = 1.0, $CHCl_3$).

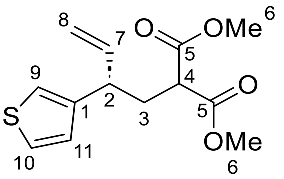

(+)-Dimethyl (*S*)-2-(2-(thiophen-3-yl)but-3-en-1-yl)malonate 3v

The corresponding compound was prepared following **General Procedure A** using vinyl cyclopropane **1** and thiophen-3-ylboronic acid **2v**. Purification by silica gel chromatography (hexane/EtOAc = 100/0 to 75/25) afforded the product **3v** as a colourless oil (70 mg, 60%). SFC analysis showed an enantiomeric excess of 95%.

^{1}H NMR (400 MHz, $CDCl_3$) δ 7.28 (dd, *J* = 5.0, 3.0 Hz, 1H; C(10)-H), 7.00 (ddd, *J* = 2.9, 1.3, 0.7 Hz, 1H; C(9)-H), 6.95 (dd, *J* = 5.0, 1.3 Hz, 1H; C(11)-H), 5.86 (ddd, *J* = 16.9, 10.3, 7.9 Hz, 1H; C(7)-H), 5.14 – 5.03 (m, 2H; 2 x C(8)-H), 3.73 (s, 3H; C(6)H3), 3.71 (s, 3H; C(6')H3), 3.40 (td, *J* = 7.6, 2.9 Hz, 2H; C(2) and C(4)), 2.41 – 2.21 (m, 2H; 2 x C(3)).

^{13}C NMR (101 MHz, $CDCl_3$) δ 169.9 (C=O), 169.8 (C=O), 143.4 (C(1)), 140.1 (C(7)), 127.1 (C(11)), 126.0 (C(10)), 120.7 (C(9)), 115.9 (C(8)), 52.7 (C(6)), 49.9 (C(4)), 43.3 (C(2)), 34.1 (C(3)).

IR ($CHCl_3$ film): 2955, 2918, 2850, 1753, 1736, 1501, 1462, 1434 cm^{-1}.

HRMS (ESI): m/z calcd for $C_{13}H_{16}O_4SNa^+$ $[M + Na]^+$ 291.0662 found 291.0660.

SFC: Chiralpak® IG, 1500 psi, 30 °C; flow: 1.5 mL/min; 1% to 30% MeOH over 5 min, then from 30% to 50% MeOH in 0.5 min, then hold 50% MeOH for 1.5 min; 97.5:2.5 er (major enantiomer t_R = 2.22 min, minor enantiomer t_R = 1.92 min).

$[\alpha]^{25}_D$ = +29.7 (c = 1.0, $CHCl_3$).

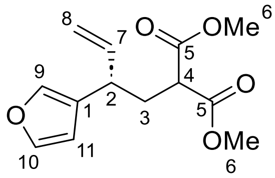

(+)-Dimethyl (*S*)-2-(2-(furan-3-yl)but-3-en-1-yl)malonate 3w

The corresponding compound was prepared following **General Procedure A** using vinyl cyclopropane **1** and furan-3-ylboronic acid **2w**. Purification by silica gel chromatography (hexane/EtOAc = 100/0 to 75/25) afforded the product **3x** as a pale yellow oil (45.5 mg, 36%). SFC analysis showed an enantiomeric excess of 95%.

^{1}H NMR (400 MHz, $CDCl_3$) δ 7.36 (t, *J* = 1.8 Hz, 1H), 7.23 (dt, *J* = 1.6, 0.8 Hz, 1H), 6.27 (dd, *J* = 1.8, 0.9 Hz, 1H), 5.79 (ddd, *J* = 16.6, 10.5, 7.9 Hz, 1H), 5.10 (t, *J* = 0.8 Hz, 1H), 5.09 – 5.04 (m, 1H), 3.73 (s, 3H), 3.71 (s, 3H), 3.42 (t, *J* = 7.4 Hz, 1H), 3.21 (q, *J* = 7.7 Hz, 1H), 2.36 – 2.08 (m, 2H).

^{13}C NMR (101 MHz, $CDCl_3$) δ 169.89, 169.82, 143.3, 139.8, 139.1, 126.5, 116.0, 109.8, 52.7, 49.8, 38.5, 33.7.

IR ($CHCl_3$ film): 3148, 3081, 3013, 2957, 2850,1756, 1738, 1640, 1503, 1438 cm^{-1}.

HRMS (ESI): m/z calcd for $C_{13}H_{16}O_5Na^+$ 275.0890 $[M + Na]^+$ found 275.0885.

SFC: Chiralpak® IG, 1500 psi, 30 °C; flow: 1.5 mL/min; 1% to 30% MeOH over 5 min, then from 30% to 50% MeOH in 0.5 min, then hold 50% MeOH for 1.5 min; 97.4:2.6 er (major enantiomer t_R = 1.54 min, minor enantiomer t_R = 1.41 min).

$[\alpha]^{25}_{D}$ = +25.1 (c = 1.0, $CHCl_3$).

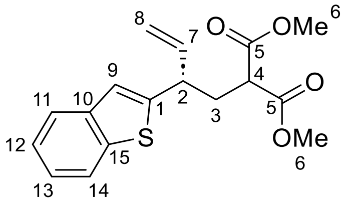

(+)-Dimethyl (*S*)-2-(2-(benzo[b]thiophen-2-yl)but-3-en-1-yl)malonate 3x

The corresponding compound was prepared following **General Procedure A** using vinyl cyclopropane **1** and benzo[b]thiophen-2-ylboronic acid **2x**. Purification by silica gel chromatography (hexane/EtOAc = 100/0 to 75/25) afforded the product **3x** as a pale yellow oil (80 mg, 65%). SFC analysis showed an enantiomeric excess of 92%.

^{1}H NMR (400 MHz, $CDCl_3$) δ 7.77 (ddt, *J* = 7.7, 1.5, 0.7 Hz, 1H; C_{Ar}-H), 7.74 – 7.65 (m, 1H; C_{Ar}-H), 7.34 – 7.25 (m, 2H; 2 x C_{Ar}-H), 7.08 (t, *J* = 0.8 Hz, 1H; C_{Ar}-H), 5.94 (ddd, *J* = 17.0, 10.1, 7.9 Hz, 1H; C(7)-H), 5.24 – 5.15 (m, 2H; 2 x C(8)-H), 3.75 (s, 3H; C(6)H_3), 3.72 (s, 3H; C(6')H_3), 3.68 (q, *J* = 7.9 Hz, 1H; C(2)-H), 3.48 (t, *J* = 7.4 Hz, 1H; C(4)-H), 2.52 – 2.34 (m, 2H; 2 x C(3)-H).

^{13}C NMR (101 MHz, $CDCl_3$) δ 169.7 (C=O), 169.6 (C=O), 147.2 (C(1)), 139.9 (C(10)), 139.4 (C(7)), 124.4 (C_{Ar}), 124.0 (C_{Ar}), 123.3 (C_{Ar}), 122.4 (C_{Ar}), 120.9 (C_{Ar}), 116.8 (C(8)), 52.8 (C(6)), 49.7 (C(4)), 43.7 (C(2)), 34.7 (C(3)).

IR ($CHCl_3$ film): 2954, 2919, 2850, 1751, 1735, 1640, 1458, 1436 cm^{-1}.

HRMS (ESI): m/z calcd for $C_{17}H_{18}O_4SNa^+$ $[M + Na]^+$ 341.0818 found 341.0816.

SFC: Chiralpak® IG, 1500 psi, 30 °C; flow: 1.5 mL/min; 1% to 30% MeOH over 5 min, then from 30% to 50% MeOH in 0.5 min, then hold 50% MeOH for 1.5 min; 96.2:3.8 er (major enantiomer t_R = 4.43 min, minor enantiomer t_R = 3.21 min).

$[\alpha]^{25}_D$ = +31.2 (c = 1.0, $CHCl_3$).

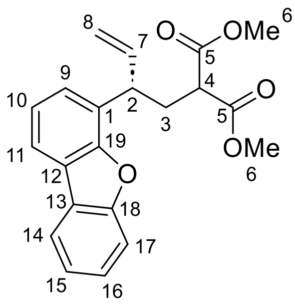

(+)-Dimethyl (*S*)-2-(2-(dibenzo[b,d]furan-4-yl)but-3-en-1-yl)malonate 3y

The corresponding compound was prepared following **General Procedure A** using vinyl cyclopropane **1** and dibenzo[b,d]furan-4-ylboronic acid **2y**. Purification by silica gel chromatography (hexane/EtOAc = 95/5 to 75/25) afforded the product **3y** as a pale yellow oil (151 mg, 86%). SFC analysis showed an enantiomeric excess of 88%.

^{1}H NMR (400 MHz, $CDCl_3$) δ 7.94 (ddd, *J* = 7.7, 1.4, 0.7 Hz, 1H; C_{Ar}-H), 7.83 (dd, *J* = 7.1, 1.8 Hz, 1H; C_{Ar}-H), 7.58 (dt, *J* = 8.2, 0.9 Hz, 1H; C_{Ar}-H), 7.46 (ddd, *J* = 8.3, 7.3, 1.4 Hz, 1H; C_{Ar}-H), 7.38 – 7.24 (m, 3H; 3 x C_{Ar}-H), 6.18 (ddd, *J* = 17.1, 10.2, 7.9 Hz, 1H; C(7)-H), 5.20 (dt, *J* = 17.1, 1.3 Hz, 1H; C(8)-H), 5.13 (dt, *J* = 10.2, 1.2 Hz, 1H; C(8')-H), 3.95 (q, *J* = 7.9 Hz, 1H; C(2)-H), 3.72 (s, 3H; C(6)H_3), 3.65 (s, 3H; C(6')H_3), 3.38 (t, *J* = 7.5 Hz, 1H; C(4)-H), 2.65 – 2.47 (m, 2H; 2 x C(3)-H).

^{13}C NMR (101 MHz, $CDCl_3$) δ 169.9 (C=O), 169.8 (C=O), 156.1 (C_{Ar}), 154.2 (C_{Ar}), 139.3 (C(7)), 127.3 (C_{Ar}), 126.7 (C(1)), 125.9 (C_{Ar}), 124.54 (C_{Ar}), 124.50 (C_{Ar}), 123.2 (C_{Ar}), 122.8 (C_{Ar}), 120.8 (C_{Ar}), 119.2 (C_{Ar}), 116.1 (C(8)), 111.9 (C_{Ar}), 52.65 (C(6)), 52.63 (C(6')), 50.0 (C(4)), 42.8 (C(2)), 33.4 (C(3)).

IR ($CHCl_3$ film): 2981, 2954, 2917, 1752, 1735, 1637, 1588, 1494, 1475, 1451, 1435 cm^{-1}.

HRMS (ESI): m/z calcd for $C_{21}H_{21}O_5^+$ 353.1384 $[M + H]^+$ found 353.1383.

SFC: Chiralpak® IG, 1500 psi, 30 °C; flow: 1.5 mL/min; 1% to 30% MeOH over 5 min, then from 30% to 50% MeOH in 0.5 min, then hold 50% MeOH for 1.5 min; 93.7:6.3 er (major enantiomer t_R = 2.75 min, minor enantiomer t_R = 2.81 min).

$[\alpha]^{25}_D$ = +21.8 (c = 1.0, $CHCl_3$).

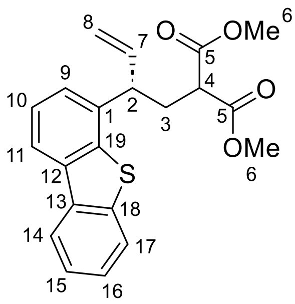

(+)-Dimethyl (*S*)-2-(2-(dibenzo[b,d]thiophen-4-yl)but-3-en-1-yl)malonate 3z

The corresponding compound was prepared following **General Procedure A** using vinyl cyclopropane **1** and dibenzo[b,d]thiophen-4-ylboronic acid **2z**. Purification by silica gel chromatography (hexane/EtOAc = 95/5 to 75/25) afforded the product **3z** as a colourless oil (116 mg, 63%). SFC analysis showed an enantiomeric excess of 74%.

^{1}H NMR (400 MHz, $CDCl_3$) δ 8.19 – 8.10 (m, 1H; C_{Ar}-H), 8.05 (dd, *J* = 7.9, 1.1 Hz, 1H; C_{Ar}-H), 7.92 – 7.81 (m, 1H; C_{Ar}-H), 7.51 – 7.40 (m, 3H; 3 x C_{Ar}-H), 7.35 – 7.32 (m, 1H; C_{Ar}-H), 6.01 (ddd, *J* = 17.1, 10.2, 7.7 Hz, 1H; C(7)-H), 5.23 (dt, *J* = 17.1, 1.3 Hz, 1H; C(8)-H), 5.17 (dt, *J* = 10.2, 1.1 Hz, 1H; C(8')-H), 3.72 (s, 3H; C(6)H_3), 3.71 (s, 3H; C(6')H_3), 3.67 (q, *J* = 8.1 Hz, 1H; C(2)-H), 3.44 (t, *J* = 7.4 Hz, 1H; C(4)-H), 2.63 – 2.47 (m, 2H; 2 x C(3)-H).

^{13}C NMR (101 MHz, $CDCl_3$) δ 169.8 (C=O) , 169.7 (C=O), 139.25 (C_{Ar}), 139.17 (C_{Ar}), 138.5 (C(7)), 136.9 (C(1)), 136.3 (C_{Ar}), 136.1 (C_{Ar}), 126.9 (C_{Ar}), 125.2 (C_{Ar}), 124.54 (C_{Ar}), 124.50 (C_{Ar}), 122.8 (C_{Ar}), 121.8 (C_{Ar}), 120.2 (C_{Ar}), 116.7 (C(8)), 52.7 (C(6)), 49.8 (C(4)), 46.9 (C(2)), 33.0 (C(3)).

IR ($CHCl_3$ film): 3064, 2999, 2981, 2953, 1750, 1734, 1636, 1573, 1443 cm^{-1}.

HRMS (ESI): m/z calcd for $C_{21}H_{20}O_4SNa^+$ $[M + Na]^+$ 391.0975 found 391.0970.

SFC: Chiralpak® IG, 1500 psi, 30 °C; flow: 1.5 mL/min; 1% to 30% MeOH over 5 min, then from 30% to 50% MeOH in 0.5 min, then hold 50% MeOH for 1.5 min; 86.8:13.2 er (major enantiomer t_R = 3.98 min, minor enantiomer t_R = 4.42 min).

$[\alpha]^{25}_D$ = +8.0 (c = 1.0, $CHCl_3$).

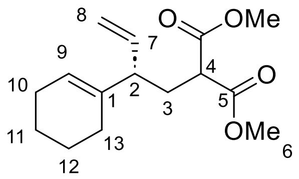

(+)-Dimethyl (*S*)-2-(2-(cyclohex-1-en-1-yl)but-3-en-1-yl)malonate 3aa

The corresponding compound was prepared following **General Procedure A** using vinyl cyclopropane **1** and cyclohex-1-en-1-ylboronic acid **2aa**. Purification by silica gel chromatography (hexane/EtOAc = 100 to 90/10) afforded the product **3aa** as a colourless oil (113 mg, 85%). SFC analysis showed an enantiomeric excess of 88%.

^{1}H NMR (400 MHz, $CDCl_3$) δ 5.67 (ddd, *J* = 16.9, 10.4, 7.8 Hz, 1H; C(7)-H), 5.47 (ddd, *J* = 4.7, 3.1, 1.5 Hz, 1H; C(9)-H), 5.06 – 5.01 (m, 1H; C(8)-H), 5.02 – 4.96 (m, 1H; C(8')-H), 3.73 (s, 3H; C(6)H_3), 3.72 (s, 3H; C(6')H_3), 3.39 (t, *J* = 7.3 Hz, 1H; C(4)-H), 2.53 (q, *J* = 7.8 Hz, 1H; C(2)-H), 2.16 – 1.97 (m, 4H; 2 x C(3)-H and C(13)-H), 1.87 (tt, *J* = 8.3, 2.3 Hz, 2H; C(10)-H), 1.66 – 1.48 (m, 4H; 2 x C(11)-H and C(12)-H).

^{13}C NMR (101 MHz, $CDCl_3$) δ 170.2 (C=O), 170.1 (C=O), 140.1 (C(7)), 138.0 (C(1)), 123.2 (C(9)), 115.3 (C(8)), 52.63 (C(6)H_3), 52.59 (C(6')H_3), 49.9 (C(4)), 49.5 (C(2)), 31.2 (C(3)), 25.8 (C(10)), 25.4 (C(15)), 23.0 (C(12)), 22.6 (C(11)).

IR ($CHCl_3$ film): 3079, 3005, 2934, 2859, 2839, 1756, 1739, 1637, 1437 cm^{-1}.

HRMS (ESI): m/z calcd for $C_{15}H_{23}O_4^+$ $[M+H]^+$ 267.1591 found 267.1587

SFC: Chiralpak® IG, 1500 psi, 30 °C; flow: 1.5 mL/min; 0% MeOH for 3 min, then 0% to 10% MeOH over 5 min, then from 10% to 30% MeOH for 0.5 min, then 30% to 50% MeOH for 0.5 min; 94.0:6.0 er (major enantiomer t_R = 3.37 min, minor enantiomer t_R = 3.13 min).

$[\alpha]^{25}_D$ = +3.6 (c = 1.0, $CHCl_3$).

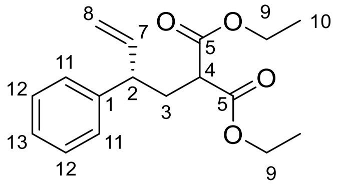

(+)-Diethyl (*S*)-2-(2-phenylbut-3-en-1-yl)malonate 4a

$[Rh(cod)OH]_2$ (5.7 mg, 0.0125 mmol, 0.025 eq) and Walphos-SL-W003-1 (20.1 mg, 0.03 mmol, 0.06 eq) were added to a flame dried 5 mL round bottom flask, sealed with a rubber septum under an argon atmosphere and dissolved in THP (0.3 mL). This solution was stirred at 60 °C (the solution turned dark brown). After 30 min, this catalyst solution was added to a flame dried 5 mL round bottom flask containing phenylboronic acid (182.9 mg, 1.5 mmol, 3.0 eq), diethyl 2-vinylcyclopropane-1,1-dicarboxylate **4** (106.1, 0.5 mmol, 1.0 eq), Cs_2CO_3 (162.9 mg, 0.5 mmol, 1 eq) and $Zn(OTf)_2$ (36.4 mg, 0.1 mmol, 0.20 eq) via syringe, and the flask rinsed with THP (0.2 mL) and added. The resulting dark brown reaction mixture was stirred at 50 °C overnight.

The reaction mixture was cooled to room temperature and Et_2O added. The reaction mixture was filtered through a plug of silica and washed with Et_2O (x3) and the filtrate collected and concentrated *in vacuo*. Purification by silica gel chromatography (hexane/EtOAc = 100/0 to 85/15) afforded the product **4a** as a colourless oil (116 mg, 80%). SFC analysis showed an enantiomeric ratio of 90%.

^{1}H NMR (400 MHz, $CDCl_3$) δ 7.35 – 7.27 (m, 2H; 2 x C_{Ar}-H), 7.25 – 7.16 (m, 3H; 3 x C_{Ar}-H), 6.00 – 5.86 (m, 1H); C(7)-H), 5.10 (t, *J* = 1.0 Hz, 1H; C(8)-H), 5.07 (dt, *J* = 5.6, 1.4 Hz, 1H; C(8')-H), 4.24 – 4.06 (m, 4H); 2 x C(9)H_2), 3.30 (td, *J* = 7.6, 3.0 Hz, 2H; C(2)-H and C(4)-H), 2.31 (td, *J* = 7.6, 2.4 Hz, 2H; 2 x C(3)-H), 1.25 (dt, *J* = 10.1, 7.1 Hz, 6H; 2 x C(10)H_3).

^{13}C NMR (101 MHz, $CDCl_3$) δ 169.6 (C=O), 169.5 (C=O), 142.8 (C(1)), 140.7 (C(7)), 128.8 (C(12)), 127.7 (C(11)), 126.8 (C(13)), 115.5 (C(8)), 61.5 (C(9)), 50.2 (C(4)), 47.7 (C(2)), 34.3 (C(3)), 14.22 (C(10)), 14.20 (C(10')).

IR ($CHCl_3$ film): 3029, 2983, 2937, 1749, 1733, 1638, 1601, 1494, 1454 cm^{-1}.

HRMS (ESI): m/z calcd for $C_{17}H_{23}O_4^+$ $[M + H]^+$ 291.1591 found 291.1588.

SFC: Chiralpak® IG, 1500 psi, 30 °C; flow: 1.5 mL/min; 1% to 30% MeOH over 5 min, then from 30% to 50% MeOH in 0.5 min, then hold 50% MeOH for 1.5 min; 94.7:5.3 er (major enantiomer t_R = 1.55 min, minor enantiomer t_R = 1.47 min).

$[\alpha]^{25}_D$ = +12.4 (c = 1.0, $CHCl_3$).

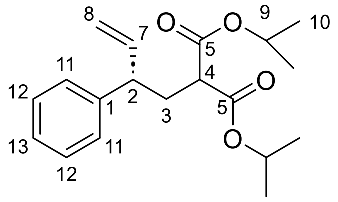

(+)-Diisopropyl (S)-2-(2-phenylbut-3-en-1-yl)malonate 5a

$[Rh(cod)OH]_2$ (5.7 mg, 0.0125 mmol, 0.025 eq) and Walphos-SL-W003-1 (20.1 mg, 0.03 mmol, 0.06 eq) were added to a flame dried 5 mL round bottom flask, sealed with a rubber septum under an argon atmosphere and dissolved in THP (0.3 mL). This solution was stirred at 60 °C (the solution turned dark brown). After 30 min, this catalyst solution was added to a flame dried 5 mL round bottom flask containing phenylboronic acid (182.9 mg, 1.5 mmol, 3.0 eq), diisopropyl 2-vinylcyclopropane-1,1-dicarboxylate **5** (120.2, 0.5 mmol, 1.0 eq), Cs_2CO_3 (162.9 mg, 0.5 mmol, 1 eq) and $Zn(OTf)_2$ (36.4 mg, 0.1 mmol, 0.20 eq) via syringe, and the flask rinsed with THP (0.2 mL) and added. The resulting dark brown reaction mixture was stirred at 50 °C overnight.

The reaction mixture was cooled to room temperature and Et_2O added. The reaction mixture was filtered through a plug of silica and washed with Et_2O (x3) and the filtrate collected and concentrated *in vacuo*. Purification by silica gel chromatography (hexane/EtOAc = 100/0 to 85/15) afforded the product **5a** as a pale yellow oil (122.6 mg, 77%). Chiral HPLC analysis showed an enantiomeric ratio of 88%

^{1}H NMR (400 MHz, $CDCl_3$) δ 7.35 – 7.27 (m, 2H; 2 x C_{Ar}-H), 7.25 – 7.14 (m, 3H; 3 x C_{Ar}-H), 6.00 – 5.87 (m, 1H; C(7)-H), 5.13 – 4.95 (m, 4H; 2 x C(8)-H and 2 x C(9)-H), 3.30 (q, J = 7.8 Hz, 1H; C(2)-H), 3.23 (t, J = 7.5 Hz, 1H; C(4)-H), 2.28 (td, J = 7.6, 2.0 Hz, 2H; 2 x C(3)-H), 1.28 – 1.20 (m, 12H; 4 x $C(10)H_3$).

^{13}C NMR (101 MHz, $CDCl_3$) δ 169.1 (C=O), 169.0 (C=O), 143.0 (C(1)), 140.8 (C(7)), 128.8 (C(12)), 127.7 (C(11)), 126.8 (C(13)), 115.5 (C(8)), 68.9 (C(9)), 50.6 (C(4)), 47.7 (C(2)), 34.2 (C(3)), 21.80 (C(10)), 21.78 (C(10’)), 21.74 (C(10”)), 21.71 (C(10’”)).

IR ($CHCl_3$ film): 3029, 2982, 2936, 1746, 1728, 1638, 1601, 1494, 1468, 1454 cm^{-1}.

HRMS (ESI): m/z calcd for $C_{19}H_{26}O_4Na^+$ $[M + H]^+$ 341.1723 found 341.1717.

HPLC Conditions: Chiralpak IC; Isocratic 2% IPA/Hexane 1mL/min; 94.3:5.7 er (major enantiomer tR = 5.70 min; minor enantiomer tR = 6.81 min), 88% ee.

$[\alpha]^{25}_{D}$ = +10.4 (c = 1.0, $CHCl_3$).

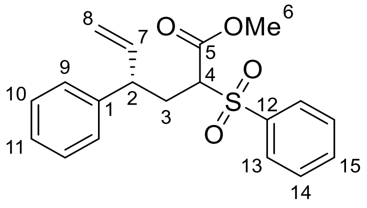

(+)-Methyl (4*S*)-4-phenyl-2-(phenylsulfonyl)hex-5-enoate 22a

The corresponding compound was prepared following **General Procedure A** using vinyl cyclopropane **22** and phenylboronic acid **2a**. Purification by silica gel chromatography (hexane/EtOAc = 100/0 to 70/30) afforded the product **22a** as a yellow oil (171.5 mg, 84%). SFC analysis showed an enantiomeric excess of 92%.

^{1}H NMR spectroscopy showed a 1:1 mixture of diastereomers was obtained. SFC analysis of one of the two diastereomers showed an enantiomeric excess of 92%, with the other diastereomer enantiomers inseparable. Stirring the diastereomeric mixture with LiOMe in THF resulted in no reduction in ee from 92%.

1:1 mixture of diastereomers:

^{1}H NMR (400 MHz, $CDCl_3$) δ 7.88 – 7.83 (m, 2H, 2 x C_{Ar}-H), 7.83 – 7.77 (m, 2H, 2 x C_{Ar}-H), 7.72 – 7.63 (m, 2H, 2 x C_{Ar}-H), 7.62 – 7.50 (m, 4H, 4 x C_{Ar}-H), 7.31 – 7.27 (m, 4H, 4 x C_{Ar}-H), 7.25 – 7.17 (m, 2H, 2 x C_{Ar}-H), 7.18 – 7.12 (m, 2H, 2 x C_{Ar}-H), 7.09 – 7.04 (m, 2H, 2 x C_{Ar}-H), 5.94 – 5.80 (m, 2H; C(7)-H), 5.13 (dt, J = 10.3, 1.0 Hz, 1H; C(8)-H), 5.10 – 5.03 (m, 2H; C(8)-H and C(8')-H), 5.01 (dt, J = 10.1, 1.2 Hz, 1H; C(8')-H), 4.07 (dd, J = 10.7, 3.6 Hz, 1H; C(4)-H), 3.80 – 3.74 (m, 1H; C(4)-H), 3.65 (s, 3H; $C(6)H_3$), 3.54 (s, 3H; $C(6)H_3$), 3.32 – 3.19 (m, 2H; 2 x C(2)-H), 2.52 – 2.31 (m, 4H; 4 x C(3)-H).

^{13}C NMR (101 MHz, $CDCl_3$) δ 166.4 (C=O), 166.3 (C=O), 142.0 (C(1)), 141.2 (C(1)), 140.3 (C(7)), 139.4 (C(7)), 137.2 (C(12)), 134.45 (C(15)), 134.40 (C(15)), 129.4 (C(Ar)), 129.3 (C(Ar)), 129.2 (C(Ar)), 129.1 (C(Ar)), 128.8 (C(Ar)), 127.6 (C(Ar)), 127.6 (C(Ar)), 127.3 (C(Ar)), 127.1 (C(Ar)), 116.7 ((C(8)), 115.5 (C(8)), 69.5 (C(4)), 69.0 (C(4)), 53.1 (C(6)), 53.0 (C(6)), 47.6 (C(2)), 47.4 (C(2)), 32.1 (C(3)), 31.9 (C(3)).

IR ($CHCl_3$ film): cm^{-1}: 3029, 2952, 2361, 1744, 1494, 1449 cm^{-1}.

HRMS (ESI): m/z calcd for $C_{19}H_{20}O_4SNa^+$ $[M + Na]^+$ 367.0975 found 367.0966.

SFC: Chiralpak® IG, 1500 psi, 30 °C; flow: 1.5 mL/min; 1% to 30% MeOH over 60 min, 96.2 :3.8 er (major enantiomer t_R = 17.62 min, minor enantiomer t_R = 18.63 min).

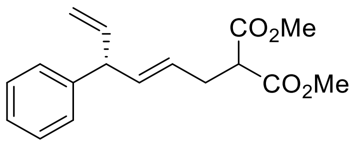

(-)-Dimethyl (*R,E*)-2-(4-phenylhexa-2,5-dien-1-yl)malonate 24

The corresponding compound was prepared following **General Procedure A** using dienyl cyclopropane **23** and phenylboronic acid **2a**. Purification by silica gel chromatography (hexane/EtOAc = 100/0 to 75/25) afforded the product **24** as a colourless oil (43 mg, 30%). SFC analysis showed an enantiomeric excess of 92%.

^{1}H NMR (400 MHz, $CDCl_3$) δ 7.33 – 7.27 (m, 2H), 7.23 – 7.13 (m, 3H), 5.98 (ddd, *J* = 17.0, 10.2, 6.6 Hz, 1H), 5.74 (ddt, *J* = 15.3, 7.2, 1.3 Hz, 1H), 5.45 (dtd, *J* = 15.3, 7.0, 1.2 Hz, 1H), 5.11 (dt, *J* = 10.2, 1.5 Hz, 1H), 5.03 (dt, *J* = 17.2, 1.6 Hz, 1H), 3.99 (t, *J* = 7.0 Hz, 1H), 3.71 (s, 3H), 3.67 (s, 3H), 3.45 (t, *J* = 7.6 Hz, 1H), 2.68 – 2.62 (m, 2H).

^{13}C NMR (101 MHz, $CDCl_3$) δ 169.5, 142.8, 140.3, 135.4, 128.6, 128.0, 126.6, 126.5, 115.4, 52.6, 52.6, 52.2, 51.9, 32.1.

IR ($CHCl_3$ film): 3030, 3006, 2953, 2849, 1755, 1739, 1636, 1601, 1542, 1493 cm^{-1}.

HRMS (ESI): m/z calculated for $C_{17}H_{20}O_4Na^+$ $[M+Na]^+$ 311.1254 found 311.1249.

SFC: Chiralpak® IG, 1500 psi, 30 °C; flow: 1.5 mL/min; 1% to 30% MeOH over 5 min, then from 30% to 50% MeOH in 0.5 min, then hold 50% MeOH for 1.5 min; 96.0:4.0 er (major enantiomer t_R = 3.07 min, minor enantiomer t_R = 2.59 min).

$[\alpha]^{25}_D$ = – 12.4 (c = 1.0, $CHCl_3$).

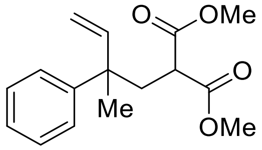

Dimethyl-2-(2-methyl-2-phenylbut-3-en-1-yl)malonate 27

The corresponding compound was prepared following a modified version of **General Procedure A** using vinyl cyclopropane **26**, phenylboronic acid **2a** and 0.5 equiv. $Zn(OTf)_2$. Purification by silica gel chromatography (hexane/EtOAc = 100/0 to 80/20) afforded the product **27** (90 mg, 65%) as a colourless oil. SFC analysis showed an enantiomeric excess of 18%.

^{1}H NMR (400 MHz, $CDCl_3$) δ 7.38 – 7.27 (m, 4H), 7.25 – 7.14 (m, 1H), 5.98 (dd, *J* = 17.5, 10.8 Hz, 1H), 5.20 – 5.06 (m, 2H), 3.64 (s, 3H), 3.61 (s, 3H), 3.34 (t, *J* = 6.1 Hz, 1H), 2.57 (dd, *J* = 14.4, 6.1 Hz, 1H), 2.47 (dd, *J* = 14.3, 6.1 Hz, 1H), 1.36 (s, 3H).

^{13}C NMR (101 MHz, $CDCl_3$) δ 170.4, 145.8, 145.4, 128.4, 126.9, 126.5, 113.1, 52.70, 52.69, 48.6, 44.1, 39.2, 24.8.

IR ($CHCl_3$ film): 2954, 2362, 2337, 1756, 1739, 1637, 1601, 1496, 1437 cm^{-1}.

HRMS (ESI): m/z calcd for $C_{16}H_{20}O_4Na^+$ $[M + Na]^+$ 299.1254 found 299.1247.

SFC: Chiralpak® IF, 1500 psi, 30 °C; flow: 1.5 mL/min; 1% to 30% MeOH over 5 min, then from 30% to 50% MeOH in 0.5 min, then hold 50% MeOH for 1.5 min; 59.1:40.9 er (major enantiomer t_R = 1.56 min, minor enantiomer t_R = 1.70 min).

4. Procedures for Product Derivatization

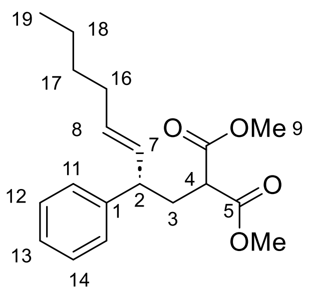

(+)-Dimethyl (*S*,*E*)-2-(2-phenyloct-3-en-1-yl)malonate 7a

Dimethyl 2-(2-phenylbut-3-en-1-yl)malonate **3a** (60 mg, 0.229 mmol, 1 equiv.) and hex-1-ene (38.5 mg, 0.457 , 2 equiv.) were dissolved in anhydrous CH_2Cl_2 (5 mL). Grubbs 2nd generation catalyst G2 (9.6 mg, 0.0115 mmol, 0.05 equiv.) was then added and a reflux condenser was attached. The reaction vessel was then purged with argon and the reaction brought to reflux. Upon completion by TLC analysis the solvent was removed and the residue was purified by silica gel chromatography (hexane/EtOAc = 100/0 to 85/15) to yield product dimethyl (*E*)-2-(2-phenyloct-3-en-1-yl)malonate as a brown oil (63 mg, 86%). 6.67 *E:Z* ratio (determined by ^{1}H NMR spectroscopy). SFC analysis of the (*E*)-product showed an enantiomeric excess of 94%.

^{1}H NMR (400 MHz, $CDCl_3$) δ 7.33 – 7.27 (m, 2H; C_{Ar}-H), 7.23 – 7.14 (m, 3H; C_{Ar}-H), 5.56 – 5.42 (m, 2H; C(7/8)-H), 3.73 (s, 3H; C(9)H_3), 3.69 (s, 3H; C(9')H_3), 3.35 (t, *J* = 7.4 Hz, 1H; C(4)-H), 3.29 – 3.16 (m, 1H; C(2)-H), 2.37 – 2.18 (m, 2H; C(16)-H), 2.08 – 1.93 (m, 2H; C(3)-H), 1.40 – 1.20 (m, 4H; C(17)-H and C(18)-H), 0.88 (t, *J* = 7.0 Hz, 3H; C(19)-H).

^{13}C NMR (101 MHz, $CDCl_3$) δ 170.1 (C=O), 169.9 (C=O), 143.7 (C(1)), 132.1 (C Olefin), 132.0 (C Olefin), 128.7 (C(12)), 127.6 (C(13)), 126.6 (C(11)), 52.7 (C(9)), 52.6 (C(9')), 50.0 (C(4)), 46.8 (C(2)), 35.0 (C(16)), 32.3 (C(3)), 31.6 (C(17)), 22.4, (C(18)), 14.1 (C(19)).

IR ($CHCl_3$ film): 3029, 2956, 2930, 1754, 1737, 1494, 1453, 1436 cm^{-1}.

HRMS (ESI): m/z calcd for $C_{19}H_{27}O_4$ 319.1904 $[M + H]^+$ found 319.1896.

SFC: Chiralpak® IC, 1500 psi, 30 °C; flow: 1.5 mL/min; 1% to 30% MeOH over 5 min, then from 30% to 50% MeOH in 0.5 min, then hold 50% MeOH for 1.5 min; 96.7:3.3 er (major enantiomer t_R = 1.59 min, minor enantiomer t_R = 1.67 min).

$[\alpha]^{25}_D$ = +27.7 (c = 1.0, $CHCl_3$).

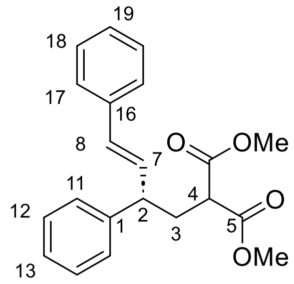

(+)-Dimethyl (*S*,*E*)-2-(2,4-diphenylbut-3-en-1-yl)malonate 7b

Dimethyl 2-(2-phenylbut-3-en-1-yl)malonate **3a** (60 mg, 0.229 mmol, 1 equiv.) and styrene (71.6 mg, 0.687 mmol, 3 equiv.) were dissolved in anhydrous CH_2Cl_2 (5 mL). Grubbs 2nd generation catalyst G2 (9.6 mg, 0.0115 mmol, 0.05 equiv.) was then added and a reflux condenser attached. The reaction vessel was then purged with argon and the reaction brought to reflux. Upon completion by TLC analysis the solvent was removed and the residue was purified by silica gel chromatography (hexane/EtOAc = 100/0 to 80/20) to yield product dimethyl (*E*)-2-(2,4-diphenylbut-3-en-1-yl)malonate as a pale yellow oil (71 mg, 92%). SFC analysis showed an enantiomeric excess of 94%.

^{1}H NMR (400 MHz, $CDCl_3$) δ 7.35 – 7.29 (m, 5H; 5 x C_{Ar}-H), 7.28 – 7.25 (m, 3H; 3 x C_{Ar}-H), 7.24 – 7.17 (m, 2H; 2 x C_{Ar}-H), 6.43 (d, *J* = 16.0 Hz, 1H; C(8)-H), 6.27 (dd, *J* = 15.9, 8.1 Hz, 1H; C(7)-H), 3.70 (s, 3H; C(9)H_3), 3.68 (s, 3H; C(9')H_3), 3.46 (q, *J* = 7.0 Hz, 1H; C(2)-H), 3.39 (t, *J* = 7.4 Hz, 1H; C(4)-H), 2.52 – 2.35 (m, 2H; 2 x C(3)-H).

^{13}C NMR (101 MHz, $CDCl_3$) δ 169.9 (C=O), 169.8 (C=O), 142.8 (C(1)), 137.1 (C(16)), 132.3 (C(7)), 130.7 (C(8)), 128.9 (C(12)), 128.6 (C(18)), 127.7 (C(11)), 127.5 (C(19)), 126.9 (C(13)), 126.4 (C(17)), 52.7 (C(23)), 50.1 (C(4)), 47.2 (C(2)), 34.7 (C(1)).

IR ($CHCl_3$ film): 3028, 2954, 2848, 1753, 1736, 1601, 1495, 1452, 1437 cm^{-1}.

HRMS (ESI): m/z calcd for $C_{21}H_{23}O_4$ 339.1591 $[M + H]^+$ found 339.1584.

SFC: Chiralpak® IG, 1500 psi, 30 °C; flow: 1.5 mL/min; 1% to 30% MeOH over 5 min, then from 30% to 50% MeOH in 0.5 min, then hold 50% MeOH for 1.5 min; 96.9:3.1 er (major enantiomer t_R = 3.16 min, minor enantiomer t_R = 2.95 min).

$[\alpha]^{25}_D$ = +14.1 (c = 1.0, $CHCl_3$).

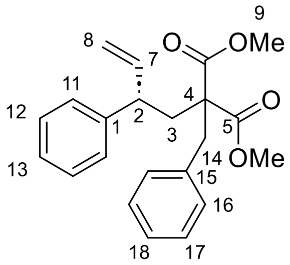

(+)-Dimethyl (*S*)-2-benzyl-2-(2-phenylbut-3-en-1-yl)malonate 8

Dimethyl 2-(2-phenylbut-3-en-1-yl)malonate **3a** (60 mg, 0.229 mmol, 1 equiv.) and NaH (11.0 mg, 0.274 mmol, 1.2 equiv.) were dissolved in anhydrous THF (5 mL) and stirred at r.t. for 30 minutes. BnBr (46.9 mg, 0.274, 1.2 equiv.) was then added and the reaction mixture stirred at r.t. overnight. Water and EtOAc were added and the layers separated. The aqueous was washed with EtOAc (x3), and the combined organics washed with brine, dried ($MgSO_4$) and concentrated *in vacuo*. The residue was purified by silica gel chromatography (hexane/EtOAc = 98/2 to 70/30) to yield product dimethyl 2-benzyl-2-(2-phenylbut-3-en-1-yl)malonate (65 mg, 81%). SFC analysis showed an enantiomeric excess of 92%.

^{1}H NMR (500 MHz, $CDCl_3$) δ 7.32 – 7.27 (m, 2H; 2 x C_{Ar}-H), 7.21 (tdd, *J* = 11.0, 8.3, 6.4 Hz, 6H; 6 x C_{Ar}-H), 7.08 – 7.03 (m, 2H; 2 x C_{Ar}-H), 5.88 (ddd, *J* = 16.9, 10.4, 7.9 Hz, 1H; C(7)-H), 4.99 (dt, *J* = 7.4, 1.3 Hz, 1H; C(8)-H), 4.96 (d, *J* = 1.1 Hz, 1H; C(8')-H), 3.60 (s, 3H' C(9)H_3), 3.54 (q, *J* = 7.0 Hz, 1H; C(2)-H), 3.42 – 3.34 (m, 2H; C(14)H_2), 3.32 (s, 3H; C(9')H_3), 2.40 – 2.28 (m, 2H; 2 x C(3)-H).

^{13}C NMR (101 MHz, $CDCl_3$) δ 171.5 (C=O), 171.2 (C=O), 143.5 (C(1)), 142.2 (C(7)), 136.0 (C(15)), 130.1 (C(Ar)), 128.6 (C(Ar)), 128.5 (C(Ar)), 128.1 (C(Ar)), 127.2 (C(13)), 126.6 (C(18)), 114.2 (C(8)), 58.0 (C(4)), 52.2 (C(9)), 52.1 (C(9')), 45.6 (C(2)), 38.6 (C(14)), 36.9 (C(3)).

IR ($CHCl_3$ film): 3030, 2952, 2360, 1736, 1637, 1602, 1496, 1453, 1435 cm^{-1}.
HRMS (ESI): m/z calcd for $C_{22}H_{24}O_4Na$ 375.1567 $[M + Na]^+$ found 375.1555.
SFC: Chiralpak® IB, 1500 psi, 30 °C; flow: 1.5 mL/min; 1% to 30% MeOH over 5 min, then from 30% to 50% MeOH in 0.5 min, then hold 50% MeOH for 1.5 min; 96.4:3.6 er (major enantiomer t_R = 1.92 min, minor enantiomer t_R = 1.82 min).

[α]$^{25}_{D}$ = +44.7 (c = 1.0, $CHCl_3$).

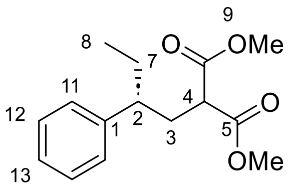

(-)-Dimethyl (*R*)-2-(2-phenylbutyl)malonate 9

Dimethyl 2-(2-phenylbut-3-en-1-yl)malonate **3a** (60 mg, 0.229 mmol, 1 equiv.) and $Rh(PPh_3)_3Cl$ (10.6 mg, 0.0115 mmol , 0.05 equiv.) were dissolved in anhydrous THF (1.5 mL). H_2 was bubbled from a balloon through the reaction mixture (x2). A H_2 balloon was then fitted and the reaction mixture stirred at r.t. for 16 h. Upon completion by TLC analysis the solvent was removed and the residue purified by silica gel chromatography (hexane/EtOAc = 98/2 to 70/30) to yield product dimethyl 2-(2-phenylbutyl)malonate as a colourless oil (56 mg, 93%). SFC analysis showed an enantiomeric excess of 94%.

^{1}H NMR (400 MHz, $CDCl_3$) δ 7.33 – 7.27 (m, 2H; 2 x C_{Ar}-H), 7.24 – 7.15 (m, 1H; 1 x C_{Ar}-H), 7.14 – 7.07 (m, 2H; 2 x C_{Ar}-H), 3.74 (s, 3H; C(9)H_3), 3.60 (s, 3H; C(9')H_3), 3.15 (dd, *J* = 10.0, 5.0 Hz, 1H; C(4)-H), 2.46 – 2.26 (m, 2H; C(2)-H and C(3)-H), 2.11 (ddd, *J* = 13.8, 10.9, 5.0 Hz, 1H; C(3')-H), 1.76 – 1.57 (m, 2H; 2 x C(7)-H), 0.77 (t, *J* = 7.4 Hz, 3H; C(8)H_3).

^{13}C NMR (101 MHz, $CDCl_3$) δ 170.08 (C=O), 170.07 (C=O), 143.6 (C(1)), 128.7 (C(12)), 128.0 (C(11)), 126.7 (C(13)), 52.6 (C(9)), 50.1 (C(4)), 45.8 (C(2)), 35.5 (C(3)), 30.0 (C(7)), 12.2 (C(8)).

IR ($CHCl_3$ film): 3029, 2960, 2933, 2876, 1754, 1737, 1495, 1437 cm^{-1}.

HRMS (ESI): m/z calcd for $C_{15}H_{21}O_4$ 265.1434 $[M + H]^+$ found 265.1430.

SFC: Chiralpak® IC, 1500 psi, 30 °C; flow: 1.5 mL/min; 1% to 30% MeOH over 5 min, then from 30% to 50% MeOH in 0.5 min, then hold 50% MeOH for 1.5 min; 97.1:2.9 er (major enantiomer t_R = 1.49 min, minor enantiomer t_R = 1.55 min).

[α]$^{25}_{D}$ = –14.9 (c = 1.0, $CHCl_3$).

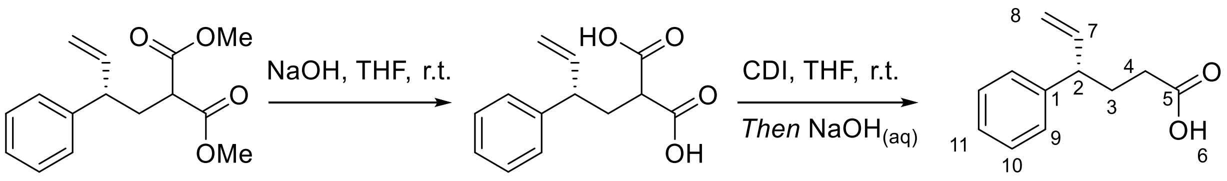

Step a: (*S*)-2-(2-phenylbut-3-en-1-yl)malonic acid

Dimethyl 2-(2-phenylbut-3-en-1-yl)malonate **3a** (60 mg, 0.229 mmol, 1 equiv.) and 1 M NaOH (4.0 mL, 4.0 mmol, 17.5 equiv.) were dissolved in anhydrous THF (2.5 mL). The reaction mixture was stirred at r.t. for 16 h. The mixture was acidified to pH <5 using 1M HCl, and EtOAc was added. The layers were separated and the aqueous layer washed with EtOAc (x2). The organic layers were combined, washed with brine, dried ($MgSO_4$) and the solvent was removed. ^{1}H NMR spectroscopy of the crude reaction mixture showed desired product; taken forward to next step without further purification.

Step b: (+)-(*S*)-4-phenylhex-5-enoic acid 10

2-(2-phenylbut-3-en-1-yl)malonic acid (53.8 mg, 0.229 mmol, 1 equiv.) and CDI (87 mg, 0.537 mmol, 2.3 equiv.) were dissolved in anhydrous THF (4.5 mL). 1 M NaOH (4.4 mL, 4.4 mmol, 19.2 equiv.) The reaction mixture was stirred at r.t. for 48 h. The mixture was acidified to pH <5 using 1M HCl, and EtOAc was added. The layers were separated and the aqueous layer washed with EtOAc (x2). The organic layers were combined, washed with brine, dried ($MgSO_4$) and the solvent was removed. The crude mixture was purified using silica gel chromatography (hexane/EtOAc = 80/20 to 0/100) to yield product 4-phenylhex-5-enoic acid as a yellow oil (36 mg, 83% over two steps). SFC analysis showed an enantiomeric excess of 94%.

^{1}H NMR (400 MHz, $CDCl_3$) δ 9.77 (s, 1H; OH), 7.32 (td, *J* = 7.4, 1.6 Hz, 2H;), 7.22 (ddq, *J* = 7.7, 6.3, 1.5 Hz, 3H), 6.02 – 5.88 (m, 1H; C(7)-H), 5.13 – 5.09 (m, 1H; C(8)-H), 5.09 – 5.06 (m, 1H; C(8')-H), 3.30 (q, *J* = 7.6 Hz, 1H; C(2)-H), 2.45 – 2.24 (m, 2H'; 2 x C(4)-H), 2.16 – 1.97 (m, 2H; 2 x C(3)-H).

^{13}C NMR (101 MHz, $CDCl_3$) δ 180.0 (C=O), 143.3 (C(1)), 141.2 (C(7)), 128.8 (C(10)), 127.7 (C(9)), 126.7 (C(11)), 115.1 (C(8)), 49.1 (C(2)), 32.2 (C(4)), 30.1 (C(3).

IR ($CHCl_3$ film): 3029, 2926, 2853, 1710, 1638, 1602, 1493, 1454, 1415, 1291, 1254, 1029 cm^{-1}.

HRMS (ESI): m/z calcd for $C_{12}H_{13}O_2$ 213.0886 $[M - H]^-$ 189.0921 found 189.0916.

SFC: Chiralpak® ID, 1500 psi, 30 °C; flow: 1.5 mL/min; 1% to 30% MeOH over 5 min, then from 30% to 50% MeOH in 0.5 min, then hold 50% MeOH for 1.5 min; 97.2:2.8 er (major enantiomer t_R = 1.92 min, minor enantiomer t_R = 2.02 min).

$\mathbf{[\alpha]^{25}_D}$ = +26.0 (c = 1.0, $CHCl_3$).

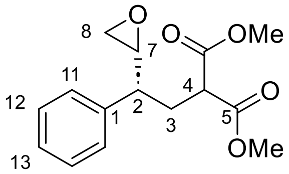

(-)-Dimethyl 2-((*R*)-2-((S)-oxiran-2-yl)-2-phenylethyl)malonate 12

Dimethyl 2-(2-phenylbut-3-en-1-yl)malonate **3a** (60 mg, 0.229 mmol, 1 equiv.) and *m*-CPBA (158.1 mg, 0.916 mmol, 4 equiv.) were dissolved in anhydrous DCM (11.5 mL) under argon. The reaction mixture was stirred at r.t. for 48 h before being quenched with $NaHCO_{3(aq)}$, and the phases separated. The organic layer was washed with H_2O, brine and dried ($MgSO_4$) and the solvent was removed by rotary evaporator. The residue was purified by silica gel chromatography (hexane/EtOAc = 80/20 to 60/40) to yield product dimethyl 2-(2-(oxiran-2-yl)-2-phenylethyl)malonate as a pale yellow oil (51 mg, 80%). ^{1}H NMR spectroscopy showed a 1:1 mixture of diastereomers was obtained. SFC analysis of one of the two diastereomers showed an enantiomeric excess of 94%.

1:1 mixture of diastereomers:

^{1}H NMR (400 MHz, $CDCl_3$) δ 7.33 (dddd, *J* = 10.5, 4.6, 2.6, 1.0 Hz, 4H), 7.29 – 7.26 (m, 1H), 7.26 – 7.23 (m, 1H), 7.23 – 7.16 (m, 4H), 3.73 (s, 6H), 3.64 (d, *J* = 1.1 Hz, 6H), 3.46 – 3.36 (m, 1H), 3.27 (dd, *J* = 9.4, 5.7 Hz, 1H), 3.13 (ddd, *J* = 6.3, 3.9, 2.6 Hz, 1H), 3.10 – 3.02 (m, 1H), 2.78 (dd, *J* = 4.9, 3.9 Hz, 1H), 2.69 (dd, *J* = 4.8, 4.0 Hz, 1H), 2.62 (dt, *J* = 11.0, 5.7 Hz, 1H), 2.58 – 2.48 (m, 3H), 2.46 – 2.34 (m, 3H), 2.34 – 2.23 (m, 1H).

^{13}C NMR (101 MHz, $CDCl_3$) δ 169.76, 169.75, 169.68, 169.61, 139.8, 139.3, 129.0, 128.9, 128.3, 128.0, 127.5, 56.1, 55.4, 52.7, 52.7, 49.6, 49.6, 47.0, 46.4, 46.4, 45.6, 32.5, 31.3.

IR ($CHCl_3$ film): 3031, 3002, 2956, 2921, 2850, 1754, 1737, 1603, 1496, 1438 cm^{-1}.

HRMS (ESI): m/z calcd for $C_{15}H_{18}O_5Na$ [M + Na]$^+$ 301.1047 found 301.1042.

SFC: Chiralpak® IG, 1500 psi, 30 °C; flow: 1.5 mL/min; 1% to 30% MeOH over 5 min, then from 30% to 50% MeOH in 0.5 min, then hold 50% MeOH for 1.5 min; 97.3:2.7 er (major enantiomer t_R = 2.38 min, minor enantiomer t_R = 2.58 min).

$[\alpha]^{25}_D$ = –9.1 (c = 1.0, $CHCl_3$).

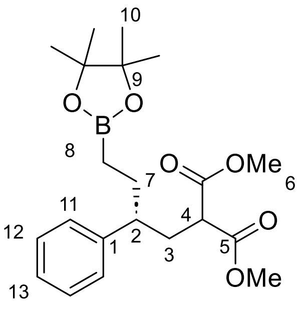

(-)-Dimethyl (*R*)-2-(2-phenyl-4-(4,4,5,5-tetramethyl-1,3,2-dioxaborolan-2-yl)butyl)malonate 13

Wilkinson's catalyst (5.3 mg, 0.00573 mmol, 2.5 mol%) Cs_2CO_3 (74.6 mg, 0.229 mmol, 1 equiv.) and anhydrous THF (0.25 mL) were added to a round bottom flask under an argon atmosphere, and the resulting mixture was stirred at r.t. After 5 minutes, dimethyl 2-(2-phenylbut-3-en-1-yl)malonate **3a** (60 mg, 0.229 mmol, 1 equiv.) in 0.8 mL of THF was added to the flask, before HBpin (66 µL, 0.458 mmol, 2 equiv.) was added dropwise and the reaction mixture stirred overnight at r.t.

The mixture was filtered through a plug of silica and the residue purified by flash column chromatography (silica gel, hexane/EtOAc = 100/0 to 50/50) to yield product dimethyl 2-(2-phenyl-4-(4,4,5,5-tetramethyl-1,3,2-dioxaborolan-2-yl)butyl)malonate (49 mg, 55%) as a pale yellow oil. SFC analysis showed an enantiomeric excess of 93%.

^{1}H NMR (400 MHz, $CDCl_3$) δ 7.32 – 7.23 (m, 2H; 2 x C_{Ar}-H), 7.22 – 7.14 (m, 1H; C_{Ar}-H), 7.14 – 7.06 (m, 2H; 2 x C_{Ar}-H), 3.72 (s, 3H; C(6)H_3), 3.59 (s, 3H; C(6')H_3), 3.15 (dd, *J* = 9.9, 5.1 Hz, 1H; C(4)-H), 2.44 (ddt, *J* = 10.9, 9.5, 4.8 Hz, 1H; C(2)-H), 2.33 (ddd, *J* = 14.2, 9.9, 4.3 Hz, 1H; C(3)-H), 2.11 (ddd, *J* = 13.9, 10.9, 5.1 Hz, 1H; C(3')-H), 1.84 – 1.62 (m, 2H; C(7)H_2), 1.21 (s, 12H; 4 x C(10)H_3), 0.70 – 0.52 (m, 2H; C(8)H_2).

^{13}C NMR (101 MHz, $CDCl_3$) δ 170.1 (C=O), 170.0 (C=O), 143.4 (C(1)), 128.6 (C(12)), 128.1 (C(11)), 126.6 (C(13)), 83.1 (C(9)), 52.5 (C(6)), 50.1 (C(4)), 46.2 (C(2)), 35.4 (C(3)), 31.4 (C(7)), 25.0 (C(10)), 24.9 (C(10')). No C(8) signal observed.

IR ($CHCl_3$ film): 3028, 2980, 2954, 2920, 2850, 2361, 2341, 1754, 1737, 1453 cm^{-1}.

HRMS (ESI): m/z calcd for $C_{21}H_{31}BO_6Na$ $[M + Na]^+$ 413.2106 found 413.2099.

SFC: Chiralpak® IC, 1500 psi, 30 °C; flow: 1.5 mL/min; 1% to 30% MeOH over 5 min, then from 30% to 50% MeOH in 0.5 min, then hold 50% MeOH for 1.5 min; 96.5:3.5 er (major enantiomer t_R = 1.82 min, minor enantiomer t_R = 1.88 min).

$[\alpha]^{25}_D$ = –5.9 (c = 1.0, $CHCl_3$).

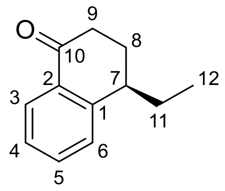

(+)-(*R*)-4-Ethyl-3,4-dihydronaphthalen-1(2*H*)-one 14

Trifluoroacetic acid (70 µL, 0.91 mmol, 7 equiv.) and trifluoroacetic anhydride (72 µL, 0.52 mmol, 4 equiv.) were added to a round bottom flask at 0 °C containing a stirring bar and dimethyl 2-(2-phenylbutyl)malonate (25 mg, 0.130 mmol, 1 equiv.). The mixture was stirred overnight and the temperature allowed to raise to room temperature. The flask was again cooled (ice bath, 0 °C) before $NaHCO_{3\ (aq)}$ and then Et_2O were added and the phases partitioned. The aqueous phase was washed with Et_2O (x3), and the combined organic layers washed with brine, dried ($MgSO_4$) and the solvent was removed *in vacuo*. The residue was purified by silica gel chromatography (hexane/EtOAc = 100/0 to 80/20) to yield product 4-ethyl-3,4-dihydronaphthalen-1(2H)-one (12 mg, 53%) as a yellow oil. SFC analysis showed an enantiomeric excess of 92%.

^{1}H NMR (400 MHz, $CDCl_3$) δ 8.02 (dd, *J* = 7.8, 1.5 Hz, 1H; C_{Ar}-H), 7.49 (td, *J* = 7.5, 1.5 Hz, 1H; C_{Ar}-H), 7.35 – 7.27 (m, 2H; 2 x C_{Ar}-H), 2.89 – 2.70 (m, 2H; C(7)-H and C(9)-H), 2.64 – 2.52 (m, 1H; C(9')-H), 2.24 (ddt, *J* = 13.5, 11.5, 4.6 Hz, 1H; C(8)-H), 2.07 (dq, *J* = 13.6, 5.1 Hz, 1H; C(8')-H), 1.86 – 1.64 (m, 2H; C(11)H_2), 1.02 (t, *J* = 7.4 Hz, 3H; C(12)H_3).

^{13}C NMR (101 MHz, $CDCl_3$) δ 198.6 (C=O), 148.4 (C_{Ar}), 133.5 (C_{Ar}), 132.1 (C_{Ar}), 128.4 (C_{Ar}), 127.5 (C_{Ar}), 126.7 (C_{Ar}), 39.7 (C(7)), 35.1 (C(9)), 27.53 (C(11)), 26.4 (C(8)), 12.3 (C(5)).

IR ($CHCl_3$ film): 3062, 3026, 2964, 2934, 1687, 1602, 1478, 1454 cm^{-1}.

HRMS (ESI): m/z calcd for $C_{12}H_{15}O$ $[M + H]^+$ 175.1117 found 175.1114.

SFC: Chiralpak® IG, 1500 psi, 30 °C; flow: 1.5 mL/min; 1% to 30% MeOH over 5 min, then from 30% to 50% MeOH in 0.5 min, then hold 50% MeOH for 1.5 min; 96.1:3.9 er (major enantiomer t_R = 2.73 min, minor enantiomer t_R = 2.90 min).

$[\alpha]^{25}_D$ = +44.1 (c = 1.0, $CHCl_3$).

5. Procedures for the Synthesis of Starting Materials

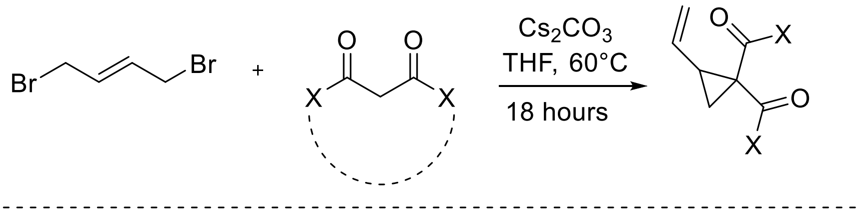

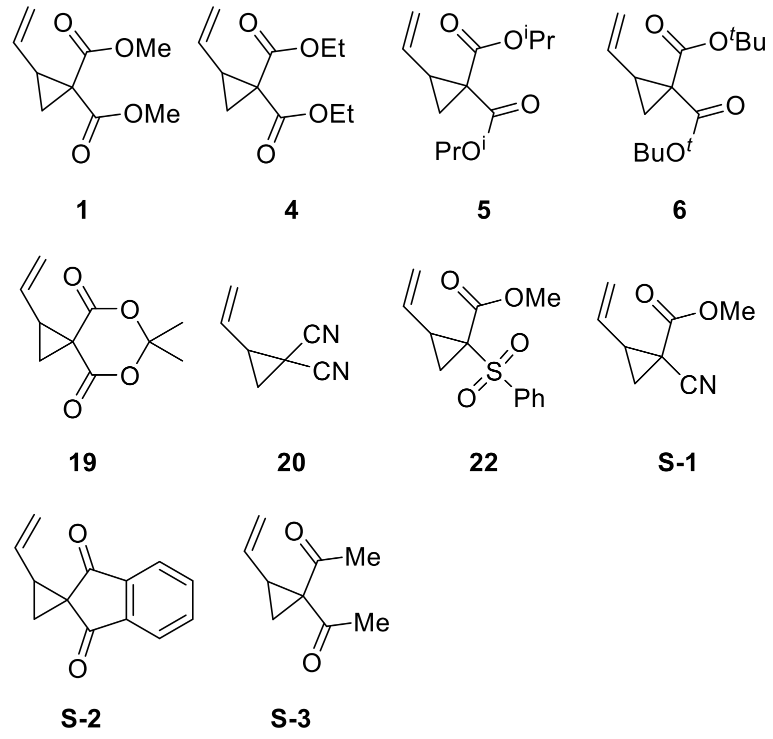

Dimethyl malonate (2.5 g, 0.0189 mol, 1 equiv.) and 1,4-dibromobut-2-ene (4.04 g, 0.0189 mol, 1 equiv.) were placed in a round bottomed flask with a stirrer bar. THF (100 mL) and cesium carbonate (15.39 g, 0.0472 mol, 2.5 equiv.) were added and the reaction mixture stirred at 60 °C overnight. After being allowed to cool to room temperature, the reaction mixture was filtered over celite and washed with diethyl ether (x3). The organic phase was washed with saturated aqueous $NaHCO_3$, followed by water and brine. After drying with $MgSO_4$, the solvent was removed under reduced pressure to give a crude yellow oil.

The crude product was purified by silica gel chromatography (20:1 petroleum ether / ethyl acetate) to afford dimethyl 2-vinylcyclopropane-1,1-dicarboxylate (**1**) as a colourless oil (2.40 g, 69%). Characterisation data was consistent with that previously reported in the literature.[1]

^{1}H NMR (400 MHz, CDCl$_3$) δ 5.52-5.35 (m, 1H), 5.30 (d, *J* = 17.2 Hz, 1H), 5.15 (d, *J* = 10.0 Hz, 1H), 3.75 (s, 6H), 2.59 (dd, *J* = 16.4, 8.4 Hz, 1H), 1.73 (dd, *J* = 7.6, 4.8Hz, 1H), 1.59 (dd, *J* = 8.8, 4.8 Hz, 1H).

Other D-A cyclopropanes **4, 5, 6**, **19**, **20**, **22**, **S-1**, **S-2**, **S-3** were prepared from the corresponding malonate according to the procedure described above. The spectroscopic data for cyclopropanes **4, 5, 6**, **19**, **20**, **22**, **S-1**, **S-2** and **S-3** satisfactorily matched that previously reported.[2–9]

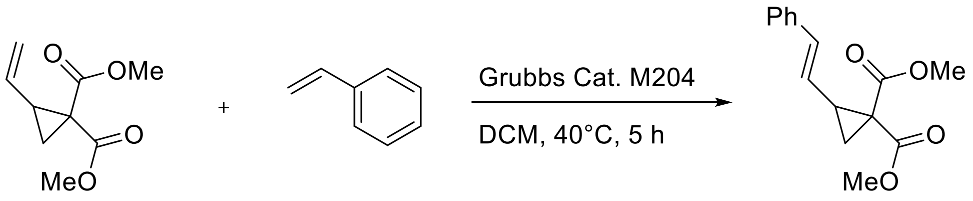

1 **15**

Vinyl cyclopropane **1** (350 mg, 1.90 mmol, 1 equiv.) and styrene (591 mg, 5.67 mmol, 3 equiv.) were dissolved in DCM (22 mL) under argon. Grubbs catalyst M204 (161.3 mg, 0.190 mmol, 0.1 equiv.) was added and the mixture heated to reflux for 5 hours.

The solvent was removed *in vacuo* and the crude product was purified by using silica gel chromatography (0 to 30% Hexane / ethyl acetate) to afford dimethyl (*E*)-2-styrylcyclopropane-1,1-dicarboxylate (**15**) as a yellow oil (57%, 280 mg). Characterisation data in accordance with reported literature.[10]

^{1}H NMR (400 MHz, CDCl$_3$) δ 7.33 – 7.26 (m, 4H), 7.25 – 7.18 (m, 1H), 6.64 (dd, *J* = 15.8, 0.8 Hz, 1H), 5.81 (dd, *J* = 15.8, 8.7 Hz, 1H), 3.77 (s, 3H), 3.73 (s, 3H), 2.81 – 2.70 (m, 1H), 1.85 (dd, *J* = 7.6, 5.0 Hz, 1H), 1.70 (dd, *J* = 9.0, 5.0 Hz, 1H).

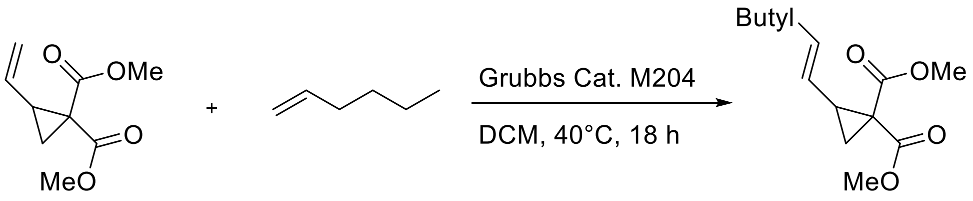

1 **16**

Vinyl cyclopropane **1** (700 mg, 3.8 mmol, 1 equiv.) and hex-1-ene (640 mg, 7.6 mmol, 2 equiv.) were dissolved in DCM (70 mL) under argon. Grubbs catalyst M204 (322.6 mg, 0.380 mmol, 0.1 equiv.)

was added and the mixture refluxed for 18 hours. The solvent was removed *in vacuo* and the crude product was purified by using silica gel chromatography (0 to 15% Hexane / ethyl acetate) to afford dimethyl-2-(hex-1-en-1-yl)cyclopropane-1,1-dicarboxylate (**16**) as a yellow oil (64%, 584 mg). Isolated as a 5.5:1 E/Z mixture. Characterisation data in accordance with reported literature.[10]

^{1}H NMR (400 MHz, $CDCl_3$) δ 5.81 – 5.50 (m, 1H), 5.12 – 4.76 (m, 1H), 3.73 (s, 6H), 2.77 – 2.45 (m, 1H), 2.21 – 1.93 (m, 2H), 1.73 – 1.52 (m, 3H), 1.40 – 1.23 (m, 3H), 0.88 (td, J = 6.9, 5.4 Hz, 3H).

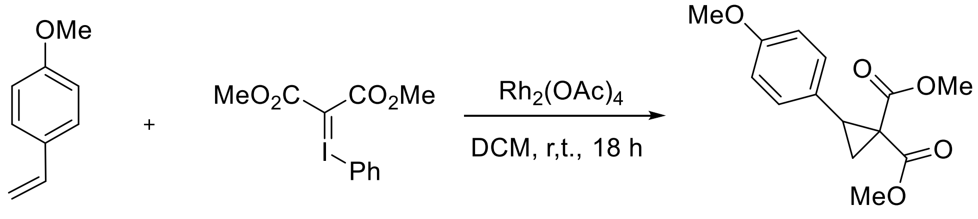

17

1-Methoxy-4-vinylbenzene (1.17 mL, 8.83 mmol, 2.5 equiv.), $Rh_2(OAc)_4$ (78 mg, 0.18 mmol, 0.05 equiv.) and DCM (50 mL) were added to a round bottom flask under argon. Ionium ylide (1.18 g, 3.53 mmol, 1 equiv.) was added over small portions over 1 hour and the mixture was stirred for one additional hour. The solvent was removed *in vacuo* and the crude product was purified using silica gel chromatography (4 : 1 hexane / ethyl acetate) to afford dimethyl 2-(4-methoxyphenyl)cyclopropane-1,1-dicarboxylate (**17**) as a colourless oil (52%, 487 mg). Characterisation data in accordance with reported literature.[11]

^{1}H NMR ($CDCl_3$, 400 MHz) δ 7.17-7.11 (m, 2H), 6.86-6.79 (m, 2H), 3.81 (s, 3H), 3.80 (s, 3H), 3.41 (s, 3H), 3.20 (t, J = 8.2 Hz, 1H), 2.18 (dd, J= 8.1, 5.2 Hz, 1H), 1.74 (dd, J= 9.3, 5.2 Hz).

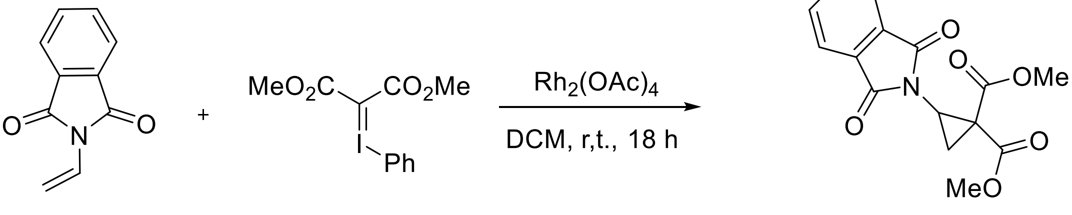

18

2-Vinylisoindoline-1,3-dione (902 mg, 5.20 mmol, 1 equiv.), $Rh_2(OAc)_4$ (23 mg, 0.052 mmol, 0.25 equiv.) and DCM (40 mL) were added to a round bottom flask under argon at 0 °C. Ionium ylide (1.91 g, 5.71 mmol, 1.1 equiv.) was added and the mixture allowed to warm to r.t. and stirred for 18 hours. The solvent was removed *in vacuo* and the crude product was purified by using silica gel

chromatography (6:4 Hexane / ethyl acetate) to afford dimethyl 2-(1,3-dioxoisoindolin-2-yl)cyclopropane-1,1-dicarboxylate (**18**) as a white solid (56%, 892 mg). Characterisation data in accordance with reported literature.[4]

^{1}H NMR (400 MHz, $CDCl_3$) δ 7.92 – 7.79 (m, 2H), 7.79 – 7.68 (m, 2H), 3.83 (s, 3H), 3.69 (dd, J = 8.5, 6.6 Hz, 1H), 3.61 (s, 3H), 2.70 (t, J = 6.5 Hz, 1H), 2.08 – 1.99 (m, 1H).

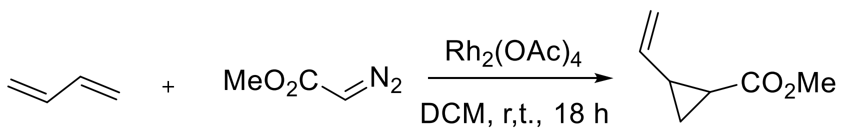

21

Methyl 2-diazoacetate (10 mmol) in 5 mL of DCM was added to a sealed tube containing 20% butadiene in toluene (30 mmol) and $Rh_2(OAc)_4$ (1 mmol) at -78 °C. The tube was allowed to warm to r.t. and stirred for 18 hours. The solvent was removed *in vacuo* and the crude purified by flash column chromatography (0-40% Et_2O in pentane) to give **21** as a yellow oil (1:1 d.r., 430 mg, 34%). Characterisation data in accordance with reported literature.[12]

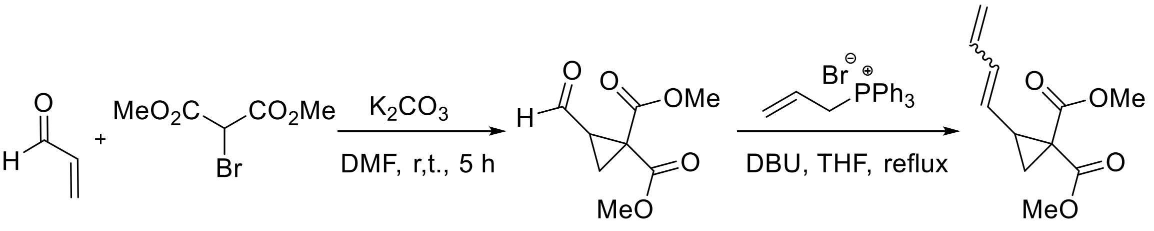

S-4 **23**

Acrolein (0.80 mL, 12.0 mmol, 1.2 equiv.), DMF (30 mL), dimethyl bromomalonate (90%, 1.46 mL, 10.0 mmol, 1 equiv.) and potassium carbonate (2.76 g, 20.0 mmol, 2 equiv.) were added sequentially to a round bottom flask under an argon atmosphere. The reaction mixture was stirred vigorously for 5 hours, before being cooled to 0 °C and diluted with Et_2O (50 mL) and H_2O (50 mL). Then, glacial acetic acid (2.40 mL, 42.0 mmol) was added, and the aqueous layer was extracted 5 times with Et_2O. The combined organic layers were washed with saturated $NaHCO_3$ solution, brine, dried ($MgSO_4$) and then concentrated *in vacuo* at 30 °C. Purification by flash column chromatography (10 to 70% Et_2O in petroleum ether) gave **S-4** as a colorless oil (949 mg, 51%). Characterisation data in

accordance with reported literature.[13] To prepare greater quantities of aldehyde, multiple reactions were performed on the above scale in order to ensure the reaction mixture was rapidly stirred.

^{1}H NMR (400 MHz, $CDCl_3$) δ 9.36 (d, *J* = 4.2 Hz, 1H), 3.78 (s, 6H), 2.82 – 2.72 (m, 1H), 2.09 (dd, *J* = 6.9, 5.0 Hz, 1H), 1.82 (dd, *J* = 8.8, 5.0 Hz, 1H).

Allyl phosphonium bromide (11.8 g, 0.0308 mol, 2 equiv.) and THF (60 mL) were added to a round bottom flask under argon. The mixture was cooled to 0 °C (ice bath) before 1,8-Diazabicyclo[5.4.0]undec-7-ene (4.14 mL, 0.0277 mol, 1.8 equiv.) was added and the mixture was then stirred for 2 hours at room temperature. **S-4** (2.87 g, 0.0154 mol, 1 equiv.) was added and the mixture refluxed for 2 hours. Upon reaction completion the mixture was diluted with Et_2O and H_2O (100 mL) and the aqueous layer extracted 3 times with Et_2O. The combined organic layers were washed with brine, dried ($MgSO_4$) and concentrated *in vacuo.* Purification by flash column chromatography (hexane / ethyl acetate) gave **23** as a colorless oil (1.60 g, 50%). The product was isolated as a mixture 2:1 *E*/*Z* mixture. Characterisation data in accordance with reported literature.[14]

^{1}H NMR (400 MHz, $CDCl_3$) δ 6.83 – 6.66 (m, 0H), 6.36 – 6.20 (m, 2H), 6.18 – 6.10 (m, 1H), 5.37 – 5.11 (m, 3H), 5.10 – 4.98 (m, 1H), 4.94 (ddq, *J* = 10.7, 9.5, 1.1 Hz, 0H), 3.77 – 3.72 (m, 9H), 2.95 – 2.80 (m, 0H), 2.66 – 2.55 (m, 1H), 1.77 – 1.59 (m, 3H).

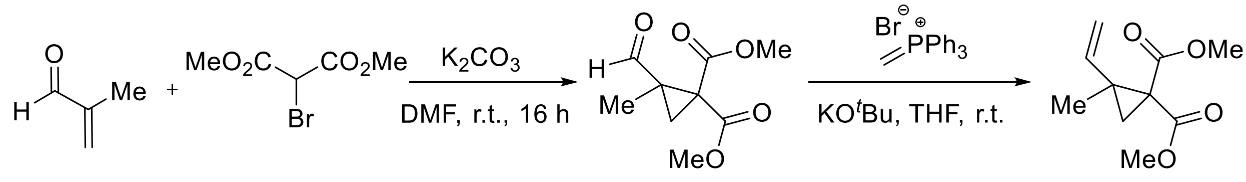

S-5 **26**

Methacrolein (0.99 mL, 12.0 mmol, 1.2 equiv.), DMF (30 mL) dimethyl bromomalonate (90%, 1.46 mL, 10.0 mmol, 1 equiv.) and potassium carbonate (2.76 g, 20.0 mmol, 2 equiv.) were sequentially added to a round bottom flask containing a stirring bar under an argon atmosphere. The resulting mixture was stirred vigorously for 16 hours, before the reaction mixture was cooled to 0 °C and diluted with Et_2O and H_2O (50 mL). Glacial acetic acid (2.40 mL, 42.0 mmol) was added, and the aqueous layer extracted 5 times with Et_2O. The combined organic layers were washed with saturated

$NaHCO_3$ solution followed by brine, dried ($MgSO_4$) and concentrated *in vacuo* at 30 °C. Purification by flash column chromatography (0 to 40% Et_2O in petroleum ether) gave **S-5** as a colorless oil (1.06 g, 53%). Characterisation data in accordance with reported literature.[15] To prepare more aldehyde, the reaction was prepared in multiple smaller batches at the above scale to ensure rapid stirring of mixture.

^{1}H NMR (400 MHz, $CDCl_3$) δ 9.26 (s, 1H), 3.78 (s, 3H), 3.75 (s, 3H), 2.11 (d, *J* = 5.6 Hz, 1H), 1.89 (d, *J* = 5.5 Hz, 1H), 1.35 (s, 3H).

Methyltriphenylphosphonium bromide (2.21 g, 6.18 mmol, 1.2 equiv.) and KO^{t}Bu (0.578 g, 5.15 mmol, 1 equiv.) were added to a round bottom flask under argon and the flask cooled to 0 °C. THF (25 mL) was added and the mixture stirred at 0 °C (ice bath) for 30 minutes before **S-5** (1.04 g, 5.15 mmol, 1 equiv.) was added dropwise over 15 minutes, and then the cooling bath was removed and the reaction mixture stirred for 4 additional hours Upon completion the reaction mixture was partitioned between Et_2O and H_2O, and the aqueous layer extracted three times with Et_2O. The combined organic layers were washing with brine, dried ($MgSO_4$) and concentrated *in vacuo.* Purification by flash column chromatography (0-40% Et_2O in pentane) gave **26** as a colorless oil (204 mg, 20%). Characterisation data in accordance with reported literature.[15]

^{1}H NMR (400 MHz, $CDCl_3$) δ 5.81 (dd, *J* = 17.3, 10.7 Hz, 1H), 5.23 – 5.10 (m, 2H), 3.76 (s, 3H), 3.71 (s, 3H), 1.81 (d, *J* = 5.3 Hz, 1H), 1.64 (d, *J* = 5.3 Hz, 1H), 1.35 (s, 3H).

6. Reactivity of Donor-Acceptor Cyclopropanes

The cyclopropanes synthesized were tested under standard conditions using **General Procedure A**, with the reactivities shown below:

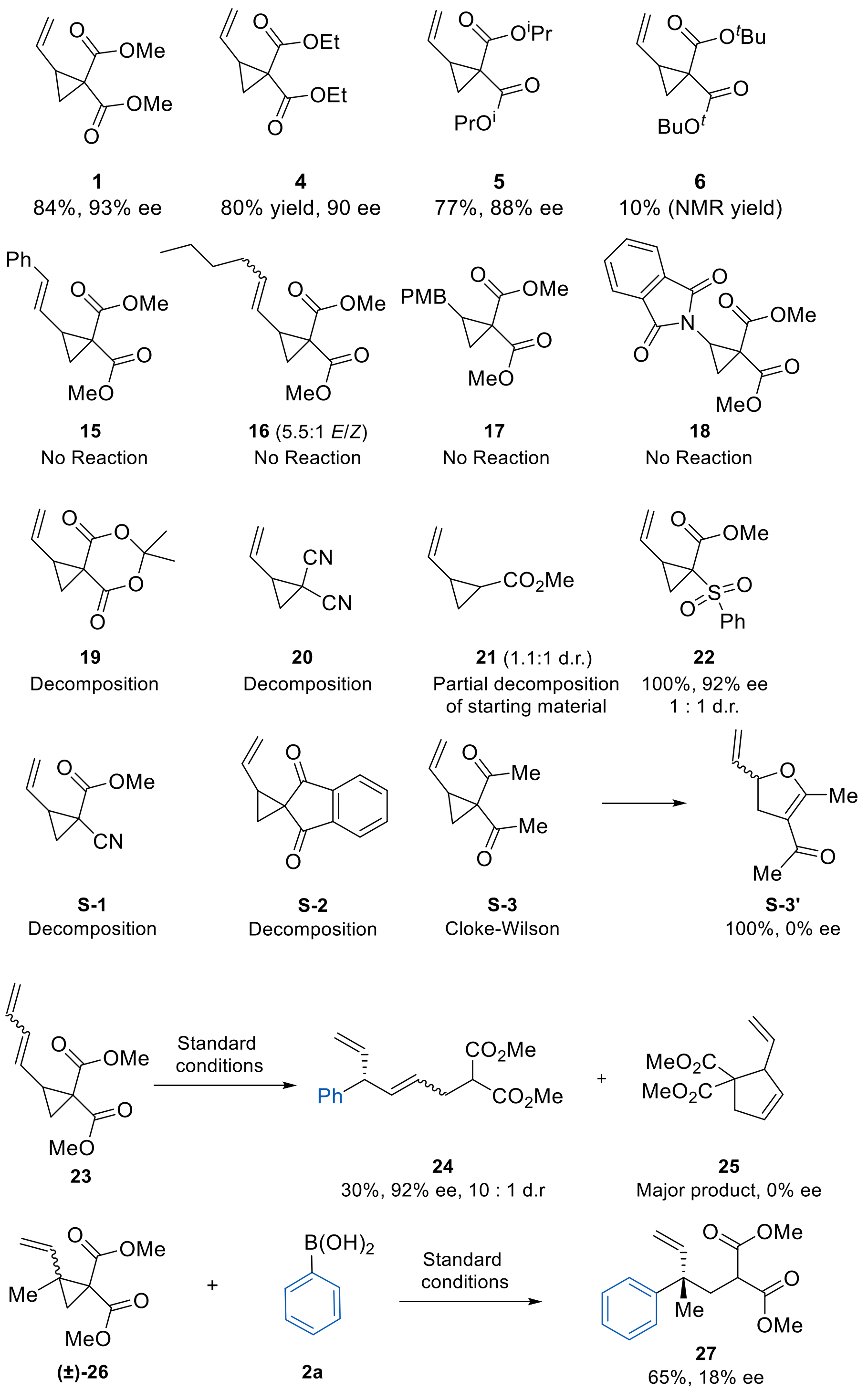

7. Mechanistic Studies

We first investigated the coordination of **L1** to [Rh(cod)OH]$_2$, where [Rh(cod)OH]$_2$ is stirred with Walphos-SL-W003-1 (**L1**) in a solvent (Toluene, THF or THP) for 30 minutes at 60 °C[16] using a 1:1 ratio of metal to ligand and a ^{31}P{^{1}H} NMR spectrum recorded. In each case, much of the **L1** remained uncoordinated, with only a small amount of bidentate species observed in solution (**Figure 1** and **2**; signals observed with a higher chemical shift are due to coordination to metal; NMR splitting pattern determines mono vs bidentate coordination).

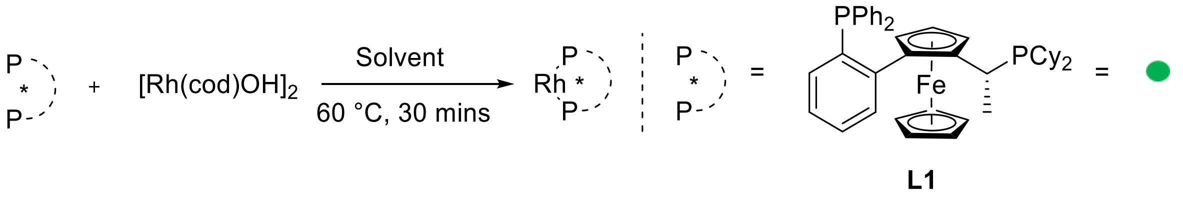

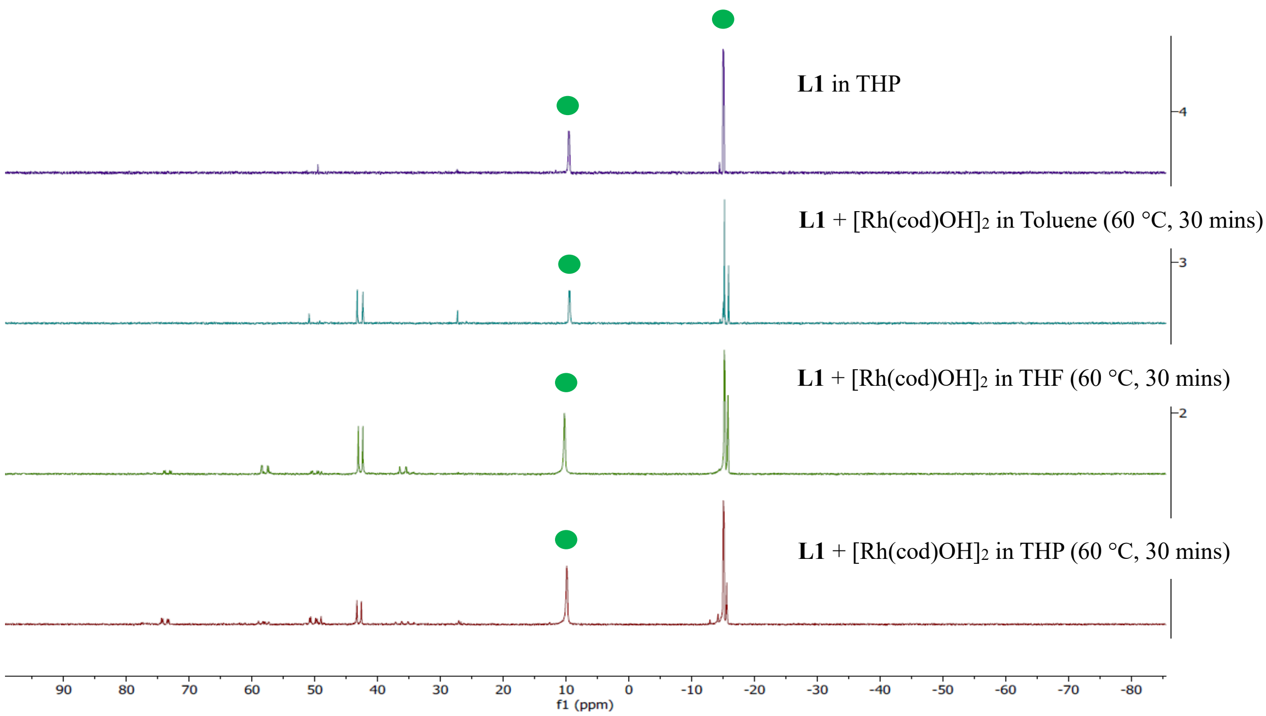

Figure 1 **L1** coordination to [Rh(cod)OH]$_2$ in Toluene, THF and THP after 30 min at 60 ºC.

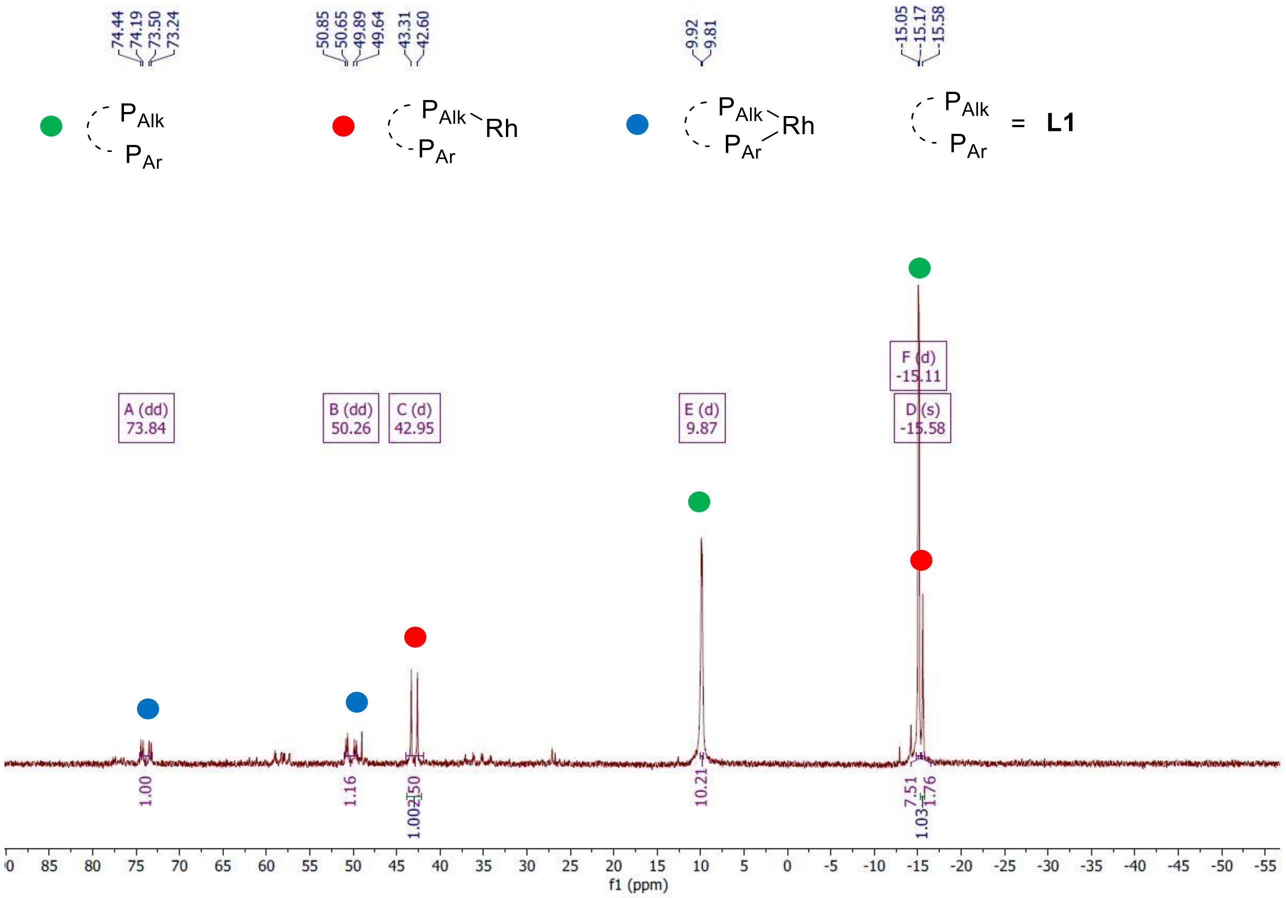

Figure 2 Coordination of Walphos-SL-W003-1 (**L1**) with $[Rh(cod)OH]_2$ in THP after 30 minutes. Uncoordinated **L1** (green dot), mono-coordinated **L1** to rhodium (observed as a doublet- no ^{31}P-^{31}P coupling seen; red dot), a bidentate **L1**-rhodium species (observed as doublet of doublets; blue dot).

Figure 2 shows the $^{31}P\{^{1}H\}$ NMR spectrum of a 1:2 ratio of $[Rh(cod)(OH)]_2$ and **L1** in THP at 60 °C after stirring for 30 min. We identified three major species in the spectrum. The uncoordinated ligand at 9.87 (d, J_{PP} = 20.0 Hz) and –15.11 (d, J_{PP} = 23.6 Hz) ppm as doublets. The phosphorus bearing alkyl groups (P_{Alk}) is located at 9.87 ppm and the phosphorus bearing aromatic groups at –15.11 ppm. The mono-coordinated complex gives signals at 42.98 (d, J_{PRh}=145.3 Hz) and –15.58 (s) ppm. The high coupling value (145.3 Hz) of the doublet at 42.98 ppm suggests that phosphorus is coordinated to Rh. The peak at –15.58 ppm represents uncoordinated P_{Ar} phosphorus. Interestingly, the P-P coupling cannot be detected in this complex. The third major complex is seen at 73.85 (dd, J_{PRh}=190.5, J_{PP}=49.9 Hz) and 50.25 (dd, J_{PRh}=201.4, J_{PP}=44.5 Hz) ppm.

In THP, after 3 and 5 hours only small amounts of bidentate rhodium species are present, with mono-coordinated compound present among a variety of unknown species (**Figure 3**).

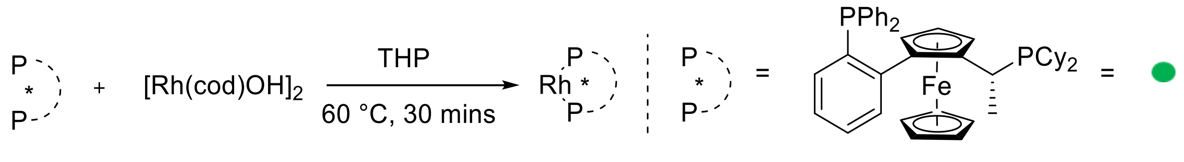

L1

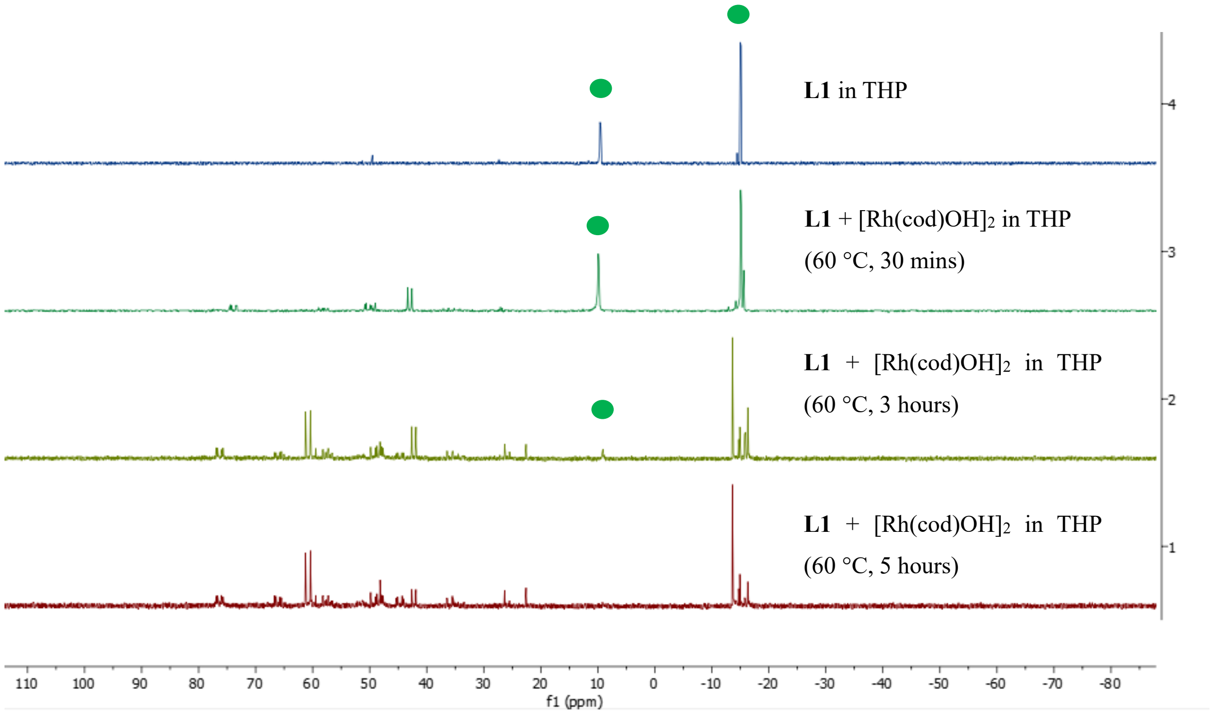

Figure 3 Coordination of **L1** with $[Rh(cod)OH]_2$ in THP over 5 hours. Uncoordinated **L1**, mono-coordinated **L1** coupling to Rhodium (observed as a doublet- no ^{31}P-^{31}P coupling seen) and bidentate **L1** coupling to Rhodium (observed as doublet of doublets) seen after 3 hours. After 5 hours, no unreacted **L1** remained in solution, but mixtures of Rh-ligand complexes are observed.

Addition of $PhB(OH)_2$ to the Rh-**L1** mixture decreased the amount of uncomplexed **L1** present but failed to give smooth ligand coordination to rhodium (**Figure 4**). However, addition of $Zn(OTf)_2$ dramatically simplified the NMR spectrum to give virtually a single rhodium-ligand complex, with phosphorous signals at 16.4 and 37.9 ppm (**Figure 4**). These signals were doublet of doublets and deshielded [37.89 (dd, J_{PRh} = 136.3, J_{PP} = 25.0 Hz) and 16.36 (dd, J_{PRh} = 147.6, J_{PP} = 23.9 Hz)], suggesting the complex is bidentate.

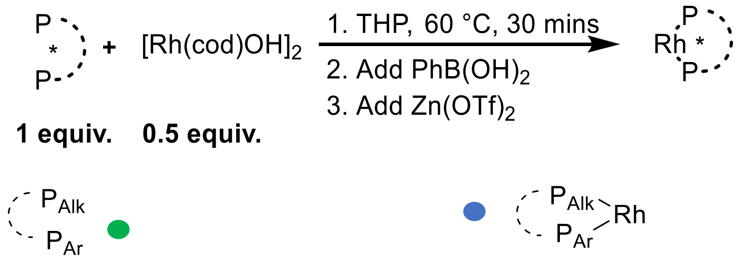

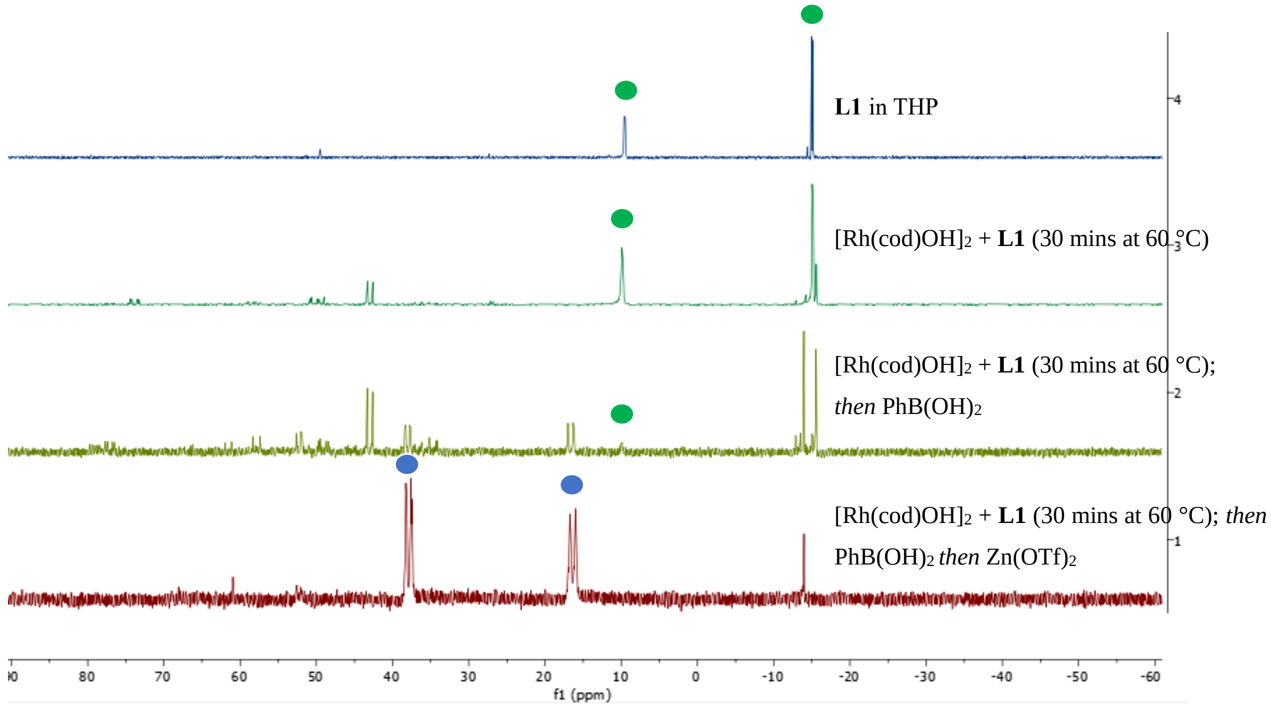

Figure 4 Effect of $Zn(OTf)_2$ on a mixture of **L1**, $[Rh(cod)OH]_2$ and $PhB(OH)_2$. After mixture for 30 minutes, much of the **L1** remained uncoordinated, with only a small amount of bidentate Rh-species in solution. Addition of $PhB(OH)_2$ decreased the amount of free **L1**, but produced a complex mixture of species; addition of $Zn(OTf)_2$ greatly simplified the spectra, with one dominant bidentate Rh-ligand complex (blue dots).

Further investigation showed $Zn(OTf)_2$ can promote clean formation of a bidentate Rh-complex (**Figure 5**). Complete mono-coordination of **L1** to zinc is achieved after 30 min, and zinc to rhodium exchange is rapid (<3 min) at room temperature.

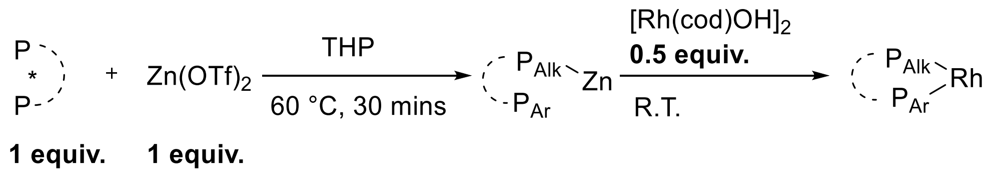

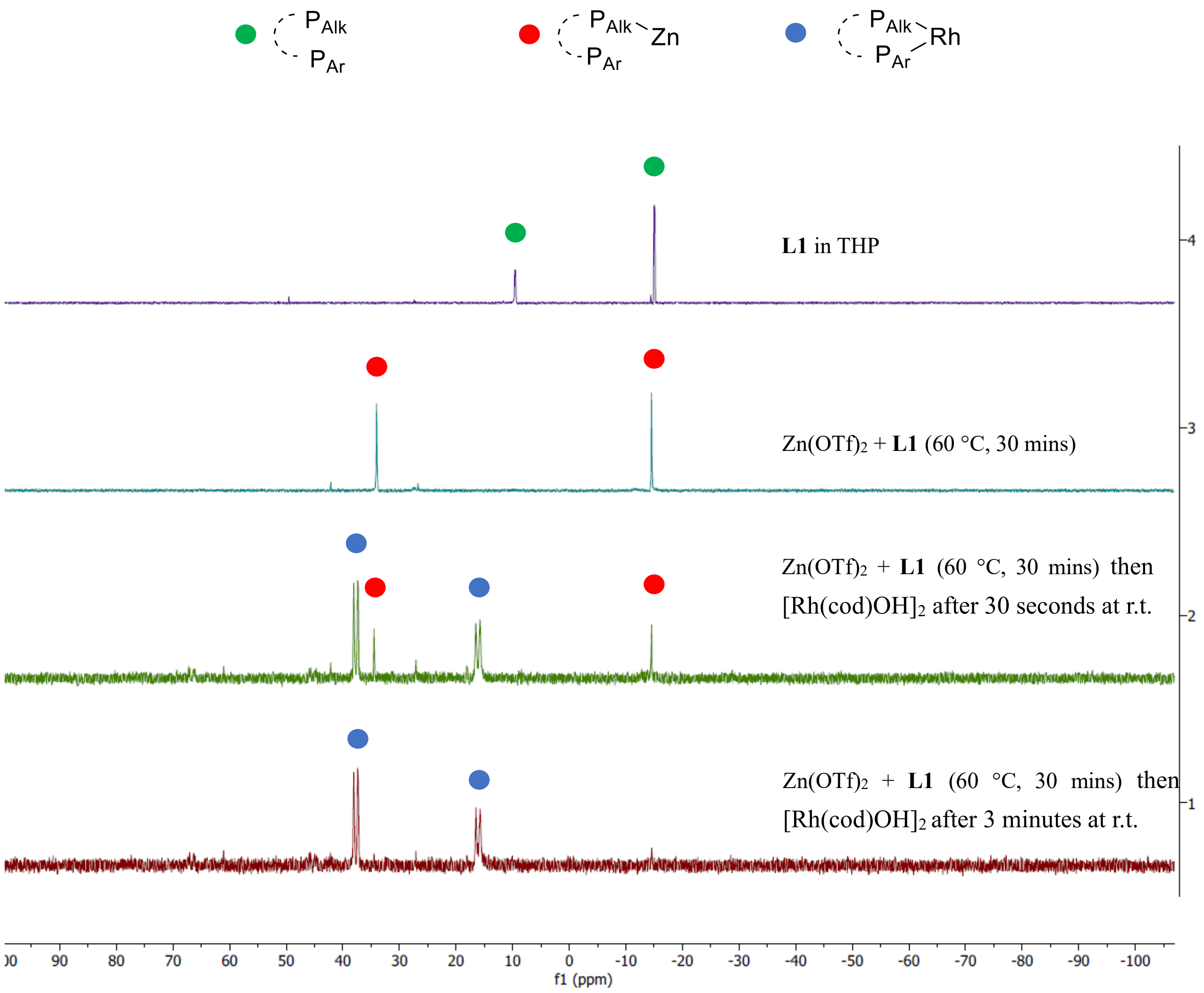

Figure 5 Formation of bidentate Rh-**L1** complex from $Zn(OTf)_2$ and **L1**. **L1** and $Zn(OTf)_2$ were stirring for 30 mins at 60 ºC to give mono-coordinated Zn-**L1** (red dots) $^{31}P\{^1H\}$ NMR (162 MHz, THP) δ 33.81 (d, J = 17.5 Hz), –14.62 (d, J = 17.8 Hz) followed by room temperature addition of $[Rh(cod)OH]_2$. Mono-coordination identified through shift of alkyl Phosphorous signal; aryl signal remained unchanged. Full mono-coordination of $Zn(OTf)_2$ with **L1** occurs within 5 minutes. Catalyst components are soluble under the conditions used.

We were curious as to how $Zn(OTf)_2$ aided complex formation and so tested additives with triflate ($La(OTf)_3$) and zinc ($ZnBr_2$) components. $^{31}P\{^1H\}$ NMR spectroscopy studies showed that both of these additives also promote formation of bidentate rhodium species (**Figure 6**).

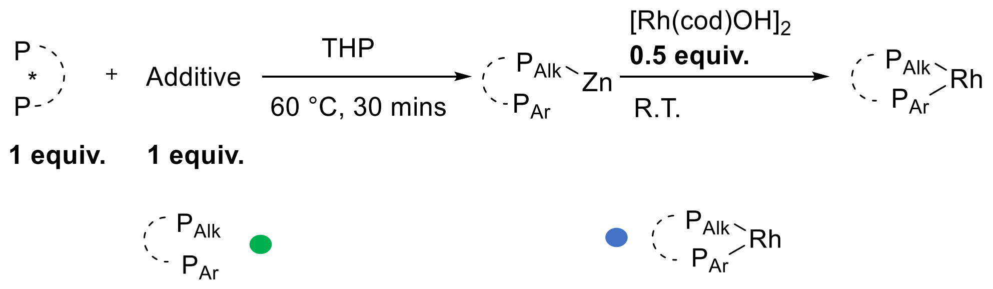

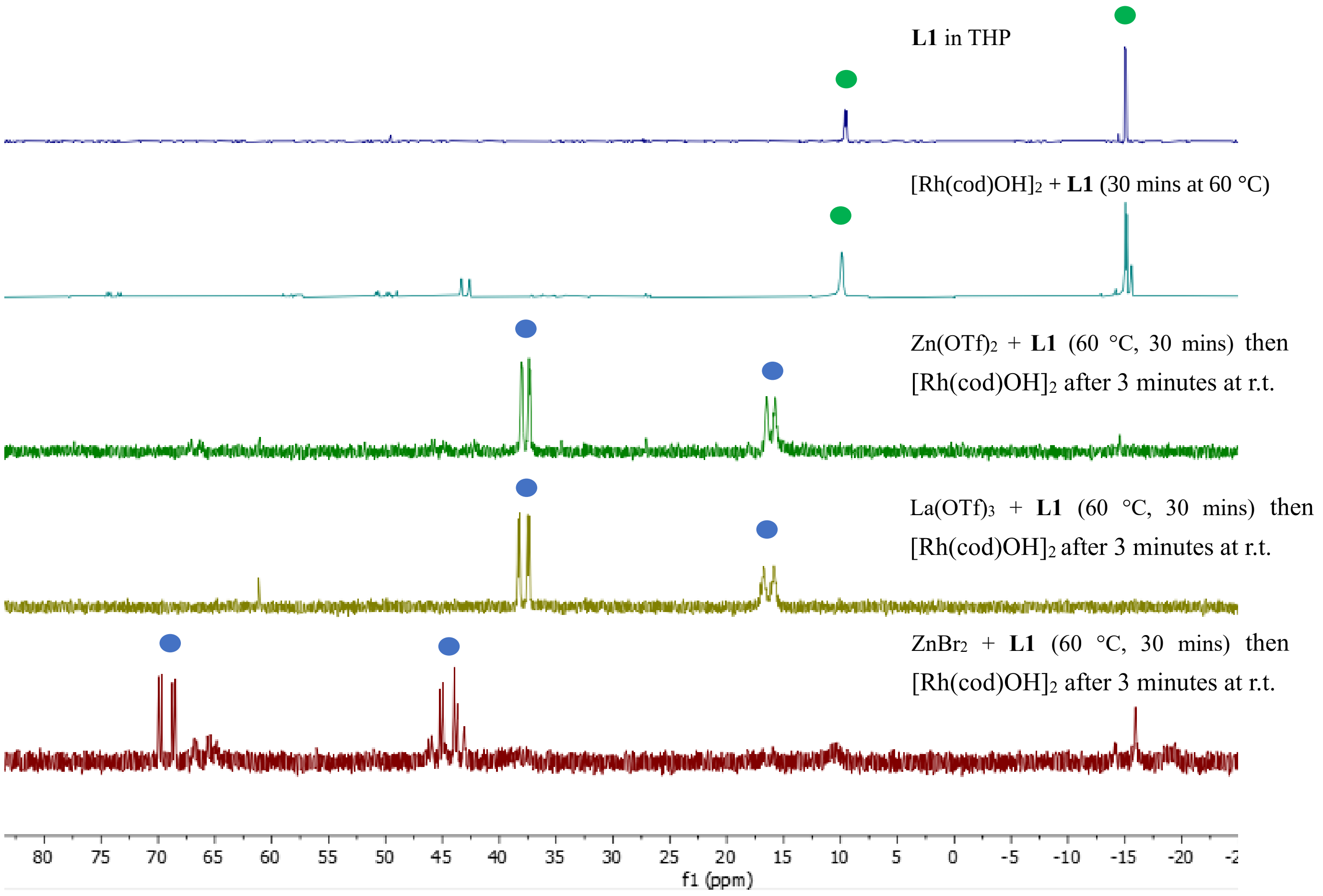

Figure 6 Rh-ligand complexation using $La(OTf)_3$and $ZnBr_2$ both gave bidentate rhodium-ligand. $ZnBr_2$ gave a different bidentate rhodium complex to $La(OTf)_3$ and $Zn(OTf)_2$ $^{31}P\{^1H\}$ NMR (162 MHz, THP) δ 69.20 (dd, J = 194.7, 41.6 Hz), 44.45 (dd, J = 207.4, 42.4 Hz).

We also found using $La(OTf)_3$ (70%, 92% ee) and $ZnBr_2$ (68%, 90% ee) both gave results comparable to $Zn(OTf)_2$ in the standard reaction to form **3a**.

The standard procedure was modified where **L1** (6 mol%) was first stirred with $Zn(OTf)_2$ (5 mol%) for 30 mins at 60 °C, before the reaction mixture was cooled and $[Rh(cod)OH]_2$ was then added at room temperature (**Figure 7**). $^{31}P\{^1H\}$NMR spectroscopy showed a bidentate Rh-complex was cleanly formed, and the solution was transferred to a flask containing the remaining reaction components (**1**, $PhB(OH)_2$, Cs_2CO_3, remaining 15 mol% $Zn(OTf)_2$) and stirred at 50 °C for 24 hours as per the standard reaction. Analysis of the crude reaction mixture showed full conversion (identical reaction profile to the standard reaction set up) to **3a**, and the ee of product **3a** was 92% (also identical to the standard reaction set up).

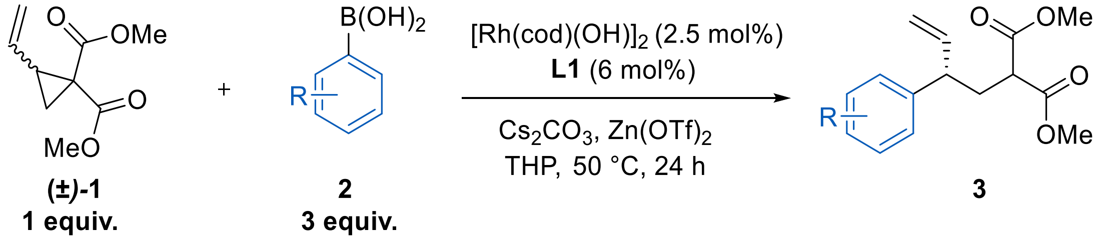

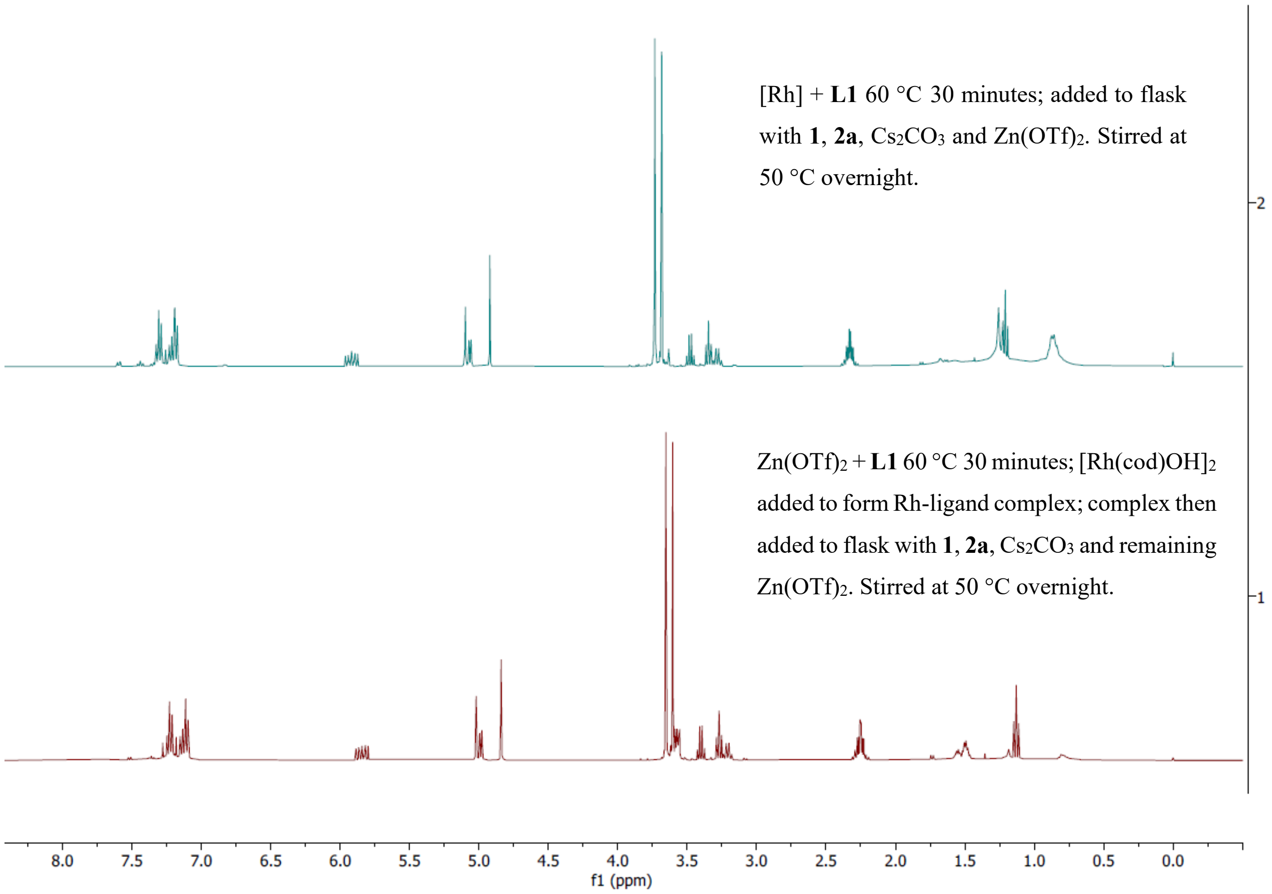

Figure 7 Crude reaction mixtures of reactions from a) standard reaction procedure; and b) **L1** and [Zn] first mixed followed by addition of $[Rh(cod)OH]_2$.

7.1 Determining the structure of the Rh-ligand complex

Further NMR spectroscopy studies were conducted to elucidate the structure of the $Zn(OTf)_2$ promoted bidentate Rh-complex. ^{1}H, ^{13}C, ^{19}F and ^{31}P analysis showed the structure to be:

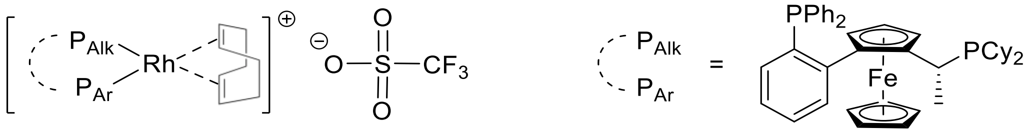

As discussed previously, the $^{31}P\{^{1}H\}$ spectrum of the complex shows two major signals at 37.3 (dd, J = 136.4, 24.1 Hz) ppm and 15.7 (dd, J = 148.2, 24.1 Hz) ppm, significantly shifted from their positions in the free **L1**. The larger coupling is consistent with $^{1}J_{Rh\text{-}P}$ whilst the smaller 24 Hz coupling was shown to be the homonuclear ^{31}P-^{31}P coupling using a COSY experiment.

A ^{13}C-HSQC experiment confirmed the presence of a rhodium-bound cyclooctadiene (cod) in the complex (Figure 8). The ^{13}C chemical shift and lineshape – broadened due to unresolved ^{103}Rh-^{13}C scalar couplings – is consistent with metal binding and the resolution of all four alkene CH groups of the cod molecule is a result of the asymmetry of **L1**.

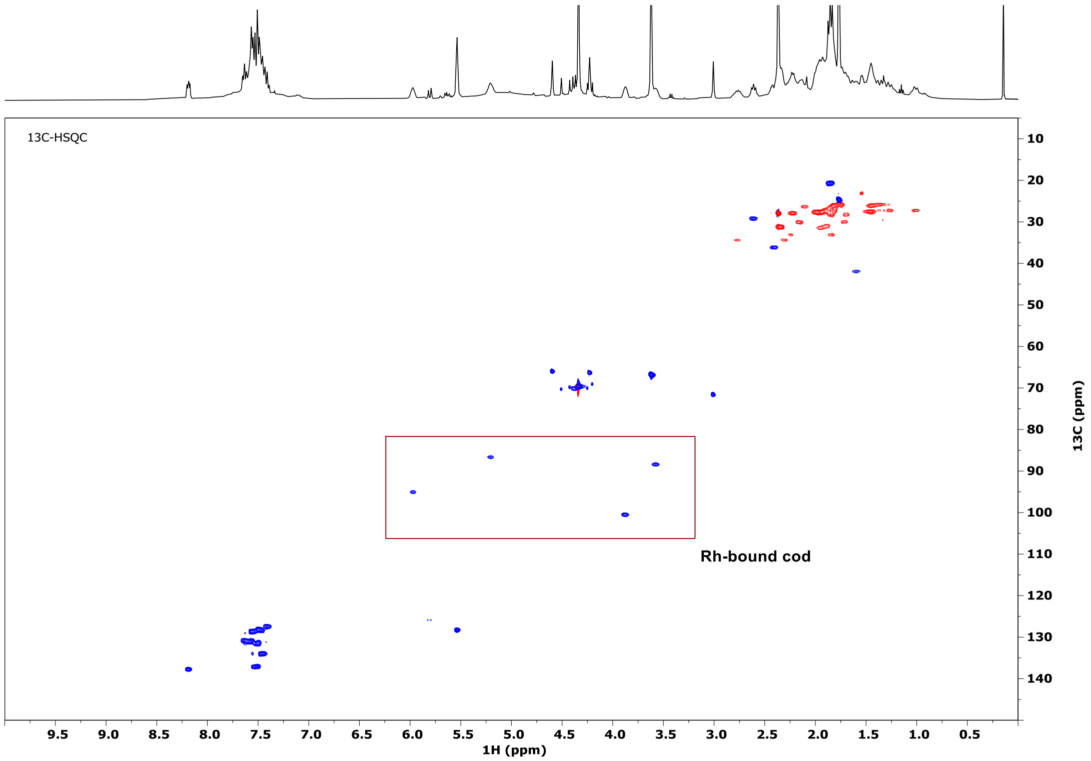

Figure 8 ^{1}H-^{13}C HSQC (500 MHz, THF-d_8) of the complex with the rhodium-bound cod CH cross-peaks highlighted.

Through-space ROESY correlations between the cod protons identified in the HSQC and protons on **L1** confirm that both **L1** and cod are part of the same complex (Figure 9).

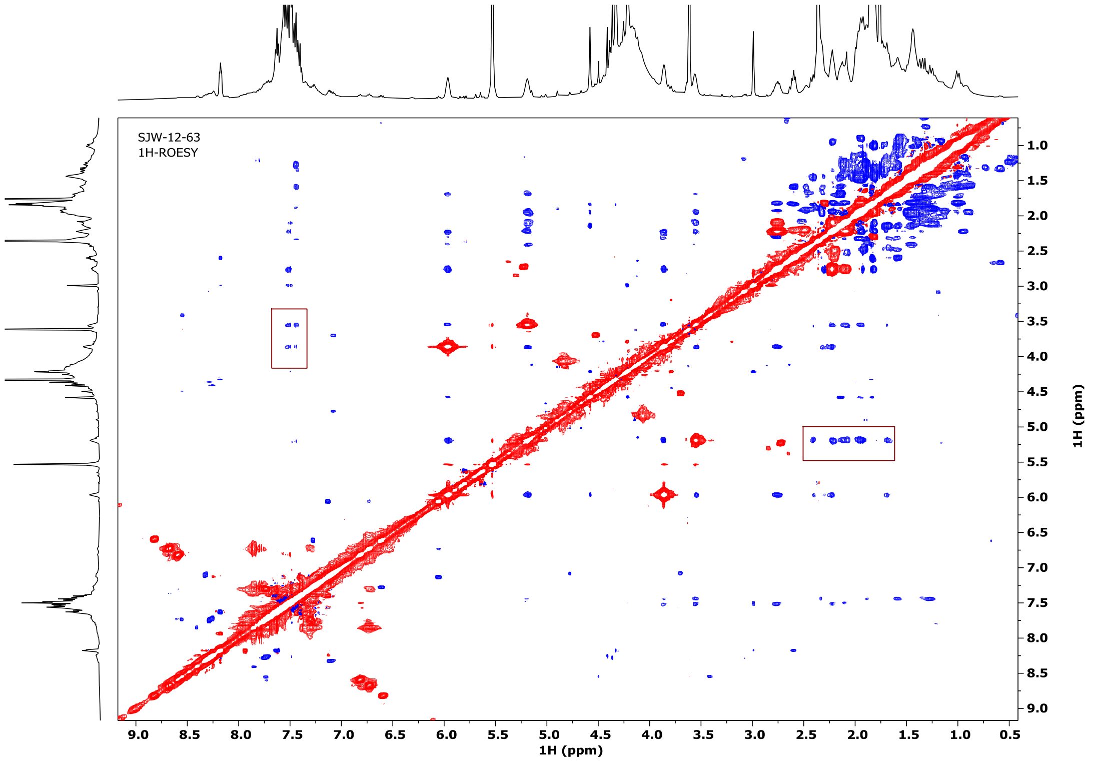

Figure 9 ^{1}H-^{1}H ROESY (500 MHz, THF-d$_8$) recorded with a spin-lock duration of 200 ms of the complex. Through-space correlations identified between cod protons and protons on the **L1** are highlighted.

^{1}H, ^{31}P and ^{19}F DOSY experiments confirm that the cod, **L1** and triflate counterion are diffusing at the same rate within the NMR tube (Figure 10, 11). Furthermore, ^{1}H-DOSY (500 MHz, THF-d8) determined the diffusion coefficient of the complex at 298K to be 6.504 ± 0.119 x 10-10 m^{2}s^{-1}, equivalent to an estimated molecular weight of 1116 ± 45 Da.[17] This is close to the expected molecular weight of the proposed complex of 1030 Da.

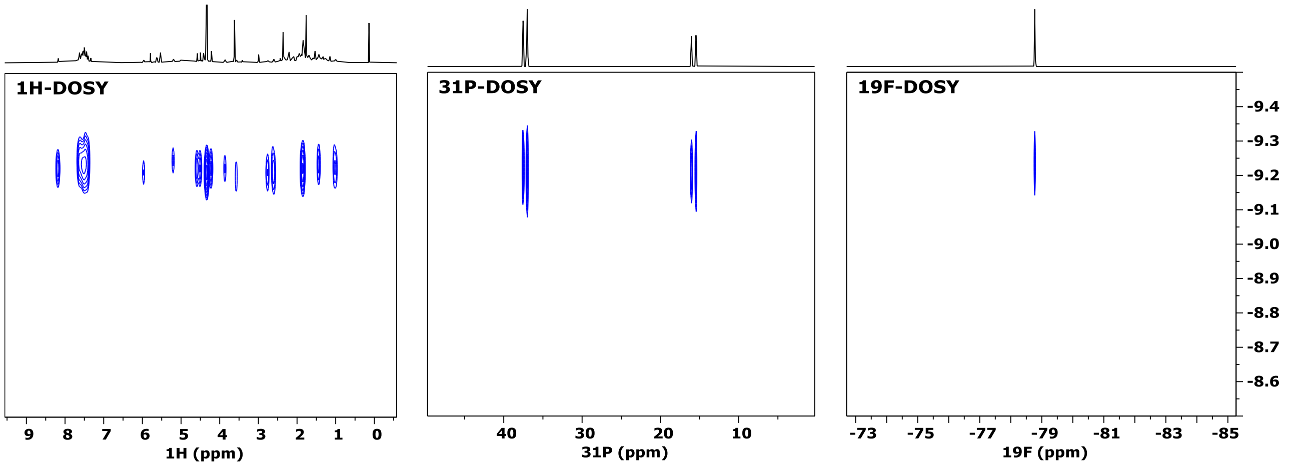

Figure 10 ^{1}H, ^{31}P and ^{19}F DOSY plots recorded in THF-d$_8$ at 298 K.

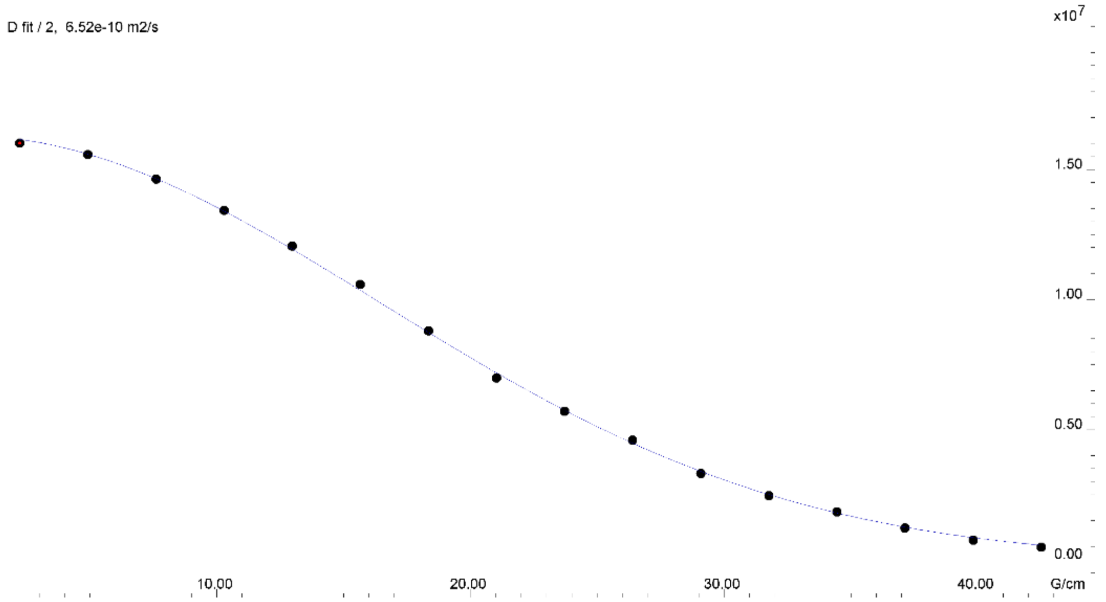

Figure 11 Example plot of ^{1}H signal intensity against applied gradient strength for the multiplet at 8.17 ppm, recorded in THF-d_8 at 298 K. The gradient strength was calibrated on a sample of D_2O and care was taken to avoid the effects of convection in the sample. Note that the figure of 6.504 ± 0.119 x 10-10 m^2s^{-1} reported in the text is an average across numerous signals in the spectrum. The data fitting and plots were constructed using the Dynamics Center software package (Bruker Biospin).

Synthesis of [Rh((*S*)-BINAP)(cod)]OTf using $Zn(OTf)_2$

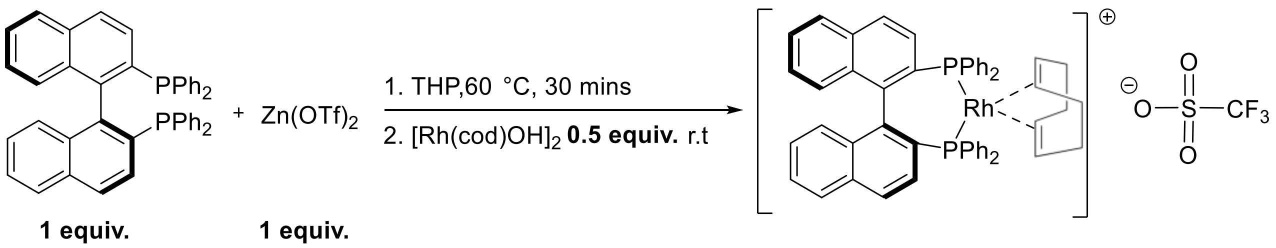

Finally, we did a control experiment where $Zn(OTf)_2$ was used to coordinate (*S*)-BINAP to $[Rh(cod)OH]_2$. The characterization data was consistent with the literature for [Rh(BINAP)(cod)]OTf.[18]

7.2 Comparison of **L1** complexation with other rhodium pre-catalysts

We investigated if $Zn(OTf)_2$ could enhance coordination of **L1** to the rhodium when using other Rh pre-catalysts. We observed that $Zn(OTf)_2$ improved coordination of **L1** to Rh with $[Rh(cod)OMe]_2$. However, $Zn(OTf)_2$ did not enhance **L1** complexation to rhodium with $[Rh(coe)_2Cl]_2$. Finally, **L1** smoothly coordinated when using $[Rh(C_2H_4)_2Cl]_2$ in the absence of $Zn(OTf)_2$.

Complexation with $[Rh(cod)OMe]_2$:

Stirring of **L1** with $[Rh(cod)OMe]_2$ for 30 minutes at 60 °C gave poor Rh-ligand complexation. However, using $Zn(OTf)_2$ gave smooth Rh-ligand coordination (**Figure 12**).

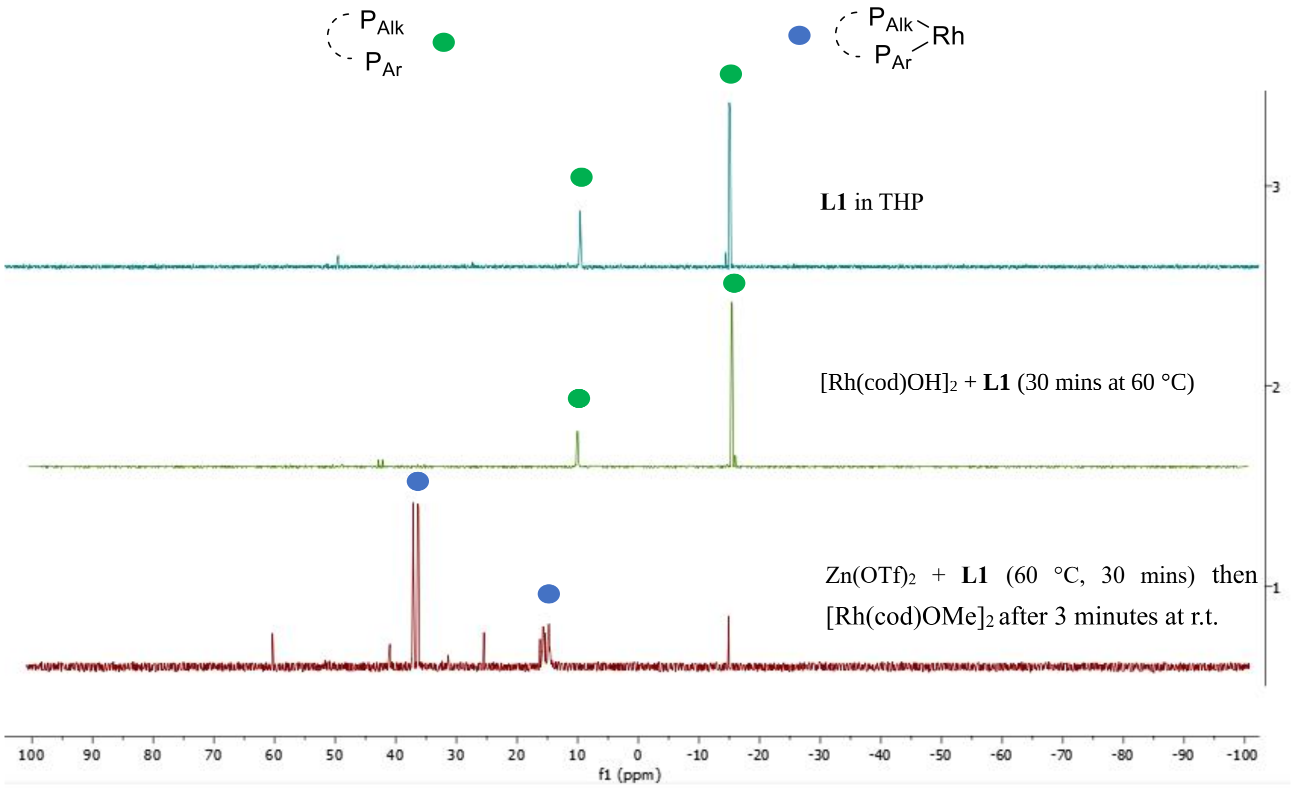

Figure 12 a) **L1** (green dots) b) $[Rh(cod)OMe]_2$ + **L1** for 30 mins at 60 °C in THP; c) Formation of bidentate Rh-**L1** complex using $Zn(OTf)_2$. **L1** and $Zn(OTf)_2$ were stirred for 30 mins at 60 °C to give mono-coordinated Zn-**L1** followed by room temperature addition of $[Rh(cod)OMe]_2$ to give [Rh(**L1**)(cod)]OTf (blue dots).

Complexation with $[Rh(coe)_2Cl]_2$:

Stirring of **L1** with $[Rh(coe)_2Cl]_2$ for 30 minutes at 60 °C gave some complexation of the Walphos ligand to the rhodium, with a large amount of uncoordinated ligand present. In this case, $Zn(OTf)_2$

was unable to facilitate improved Rh-ligand complexation, with Zn-**L1** identified in the $^{31}P\{^{1}H\}$ NMR spectrum after addition of $[Rh(coe)_2Cl]_2$ (**Figure 13**).

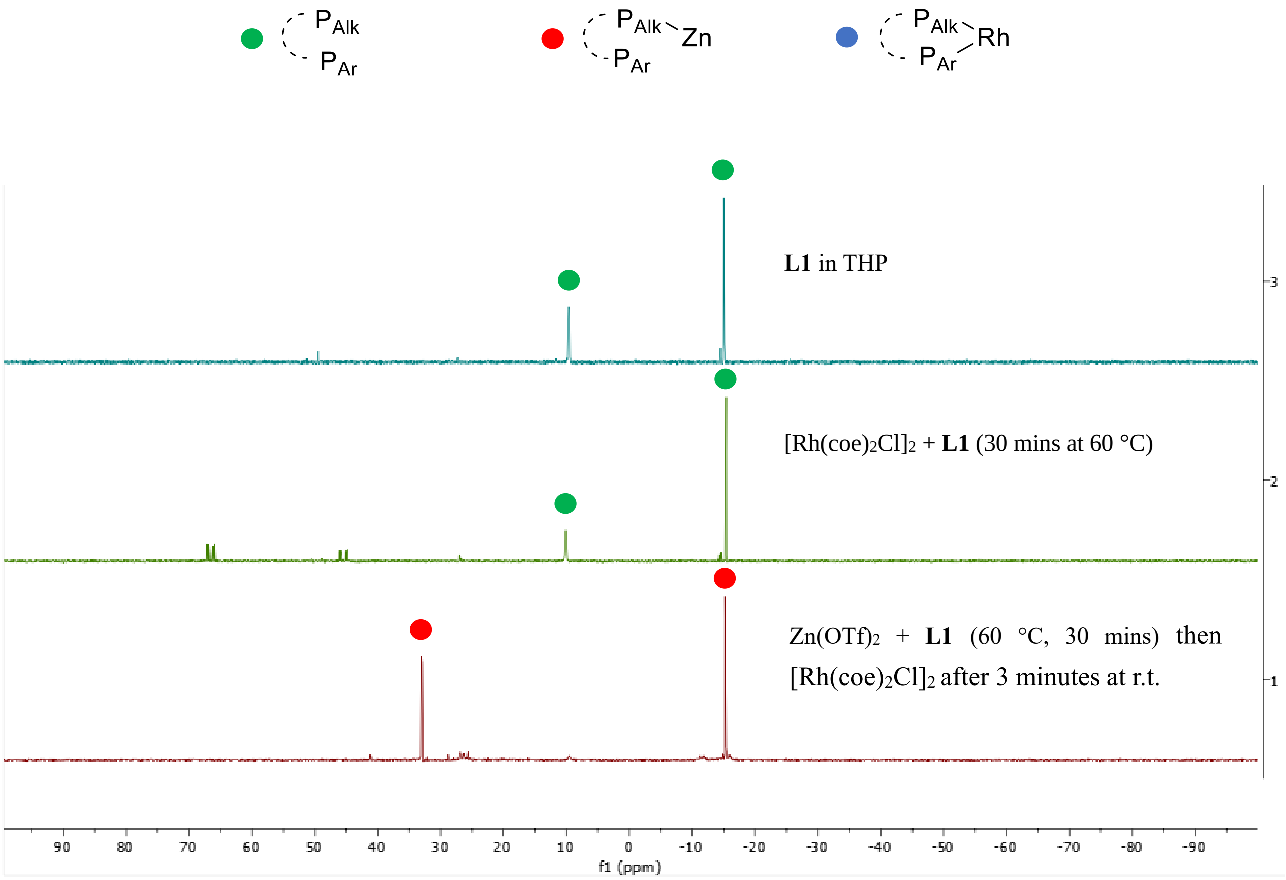

Figure 13 a) **L1** (green dots) b) $[Rh(coe)_2Cl]_2$ + **L1** for 30 mins at 60 °C in THP; c) Little formation of bidentate Rh-**L1** complex using $Zn(OTf)_2$. **L1** and $Zn(OTf)_2$ were stirred for 30 mins at 60 °C to give mono-coordinated Zn-**L1** followed by room temperature addition of $[Rh(coe)_2Cl]_2$ which did not result in **L1** coordination to rhodium, with Zn-**L1** (red dots) observed in the NMR spectrum (red dot).

Complexation with $[Rh(C_2H_4)_2Cl]_2$:

Stirring of **L1** with $[Rh(C_2H_4)_2Cl]_2$ for 30 minutes at 60 °C gave excellent complexation of **L1** to the rhodium, with no uncoordinated **L1** in solution by $^{31}P\{^{1}H\}$ NMR spectroscopy. Interestingly, adding $Zn(OTf)_2$ here actually gave a messier reaction than omitting the zinc salt. (**Figure 14**).

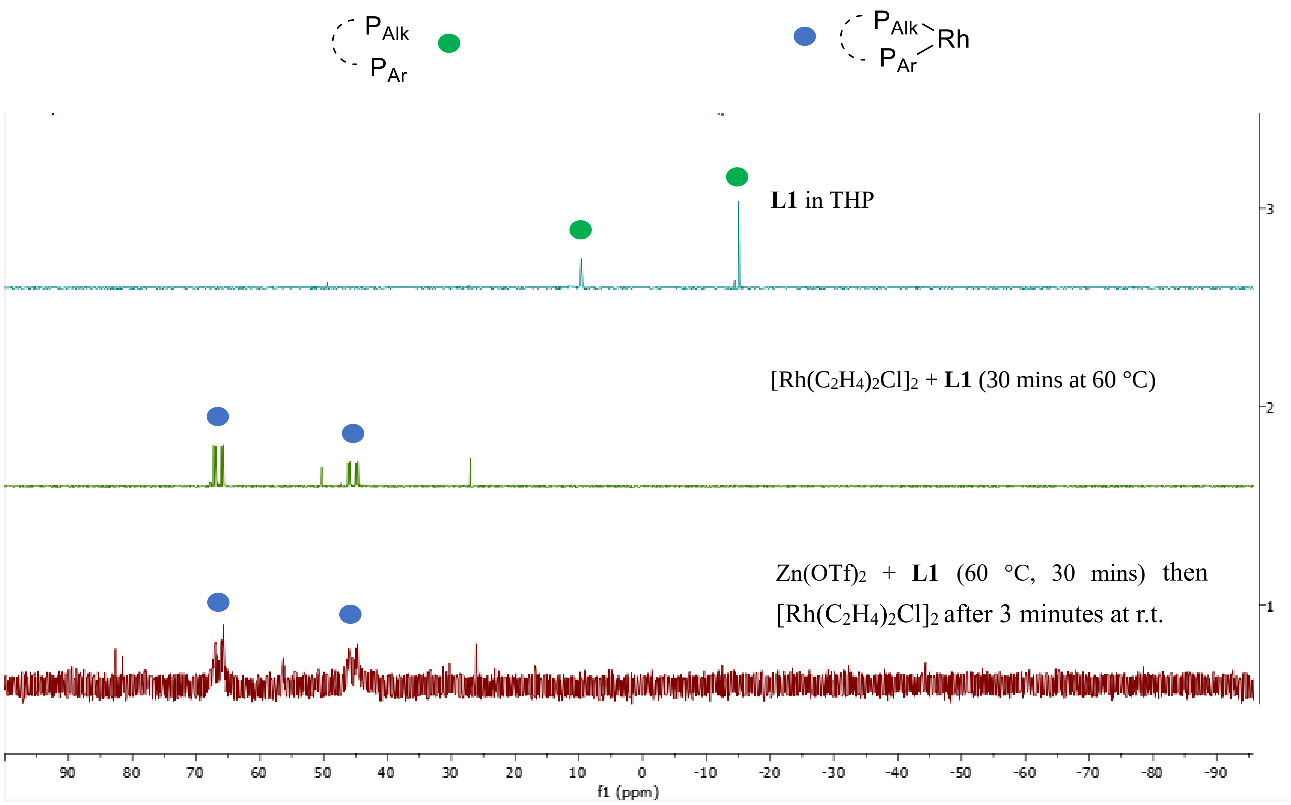

Figure 14 a) **L1** (green dots) b) $[Rh(C_2H_4)_2Cl]_2$ + **L1** for 30 mins at 60 ºC in THP gave clean bidentate Rh-**L1** complex c) Attempts to form bidentate Rh-**L1** complex using $Zn(OTf)_2$. **L1** and $Zn(OTf)_2$ was stirred for 30 mins at 60 ºC to give mono-coordinated Zn-**L1** followed by room temperature addition of $[Rh(C_2H_4)_2Cl]_2$, giving very little Rh-ligand complex (blue dots).

7.3 Kinetic studies in the presence and absence of $Zn(OTf)_2$

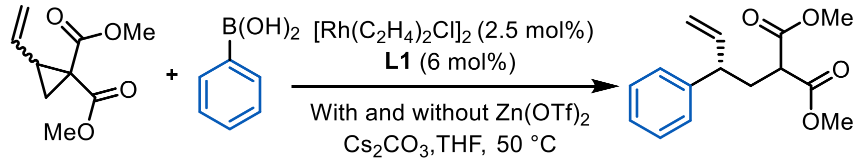

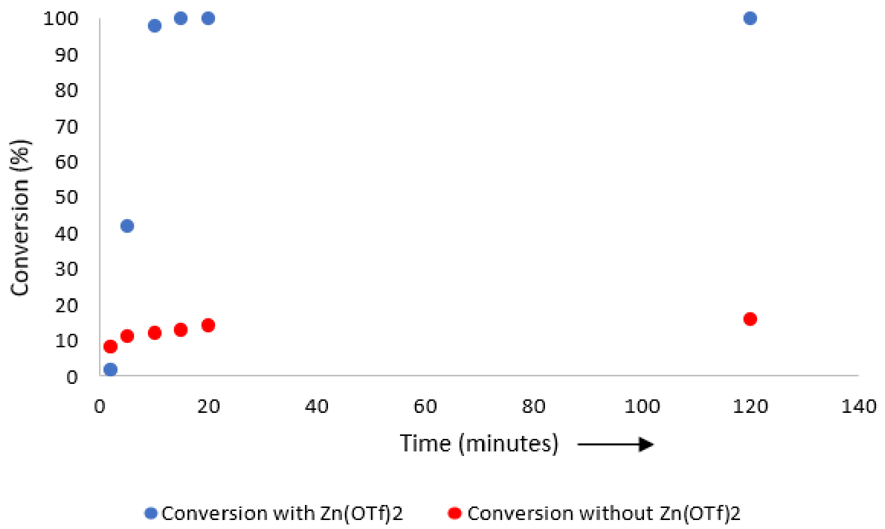

Figure 15 Conversion of starting material with $Zn(OTf)_2$ (blue dots) and without $Zn(OTf)_2$ (red dots) over time using $[Rh(C_2H_4)_2Cl]_2$ as the pre-catalyst.

<u>Procedure for monitoring of reaction using $[Rh(C_2H_4)_2Cl]_2$ as the pre-catalyst with $Zn(OTf)_2$</u>

$[Rh(C_2H_4)_2Cl]_2$ (9.7 mg, 0.0250 mmol, 0.025 eq) and Walphos-SL-W003-1 (40.2 mg, 0.06 mmol, 0.06 eq) were added to a flame dried 5 mL round bottom flask, sealed with a rubber septum under an argon atmosphere and dissolved in THF (0.6 mL). This solution was stirred at 60 °C (the solution turned dark brown). After 30 min, this catalyst solution was added to a flame dried 5 mL round bottom flask containing phenylboronic acid (3.0 mmol, 3.0 eq), dimethyl 2-vinylcyclopropane-1,1-dicarboxylate **1** (184.2, 1.0 mmol, 1.0 eq), Cs_2CO_3 (325.8 mg, 1.0 mmol, 1 eq) and $Zn(OTf)_2$ (72.7 mg, 0.2 mmol, 0.20 eq) via syringe, and the flask rinsed with THF (0.4 mL) and added. The resulting dark brown reaction mixture was stirred at 50 °C, with ≈0.05 mL aliquots taken at 2, 5, 10, 15, 20 minutes and 2 hours, and the reaciton mixture stirred for 18 h and the final aliquot (0.5 mL) taken.

The aliquots were mixed with Et_2O and filtered through a plug of silica and washed with Et_2O (x3) and the filtrate collected and concentrated *in vacuo*. Conversion determined by ^{1}H NMR spectroscopy, with CH_2Br_2 used as an internal standard.

<u>Procedure for monitoring of reaction using $[Rh(C_2H_4)_2Cl]_2$ as the pre-catalyst without $Zn(OTf)_2$</u>

$[Rh(C_2H_4)_2Cl]_2$ (9.7 mg, 0.0250 mmol, 0.025 eq) and Walphos-SL-W003-1 (40.2 mg, 0.06 mmol, 0.06 eq) were added to a flame dried 5 mL round bottom flask, sealed with a rubber septum under an argon atmosphere and dissolved in THF (0.6 mL). This solution was stirred at 60 °C (the solution turned dark brown). After 30 min, this catalyst solution was added to a flame dried 5 mL round bottom flask containing phenylboronic acid (3.0 mmol, 3.0 eq), dimethyl 2-vinylcyclopropane-1,1-dicarboxylate **1** (184.2, 1.0 mmol, 1.0 eq) and Cs_2CO_3 (325.8 mg, 1.0 mmol, 1 eq) via syringe, and the flask rinsed with THF (0.4 mL) and added. The resulting dark brown reaction mixture was stirred at 50 °C, with ≈0.05 mL aliquots taken at 2, 5, 10, 15, 20 minutes, 2 hours, and the reaciton mixture stirred for 18 h and the final aliquot (0.5 mL) taken.

The aliquots were mixed with Et_2O and filtered through a plug of silica and washed with Et_2O (x3) and the filtrate collected and concentrated *in vacuo*. Conversion determined by ^{1}H NMR spectroscopy, with CH_2Br_2 used as an internal standard.

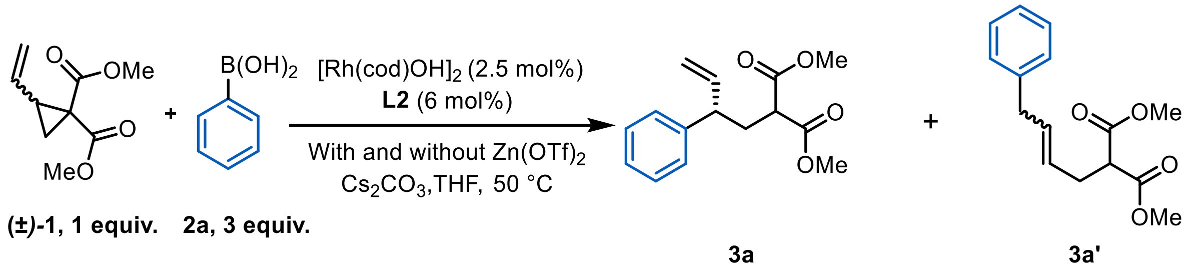

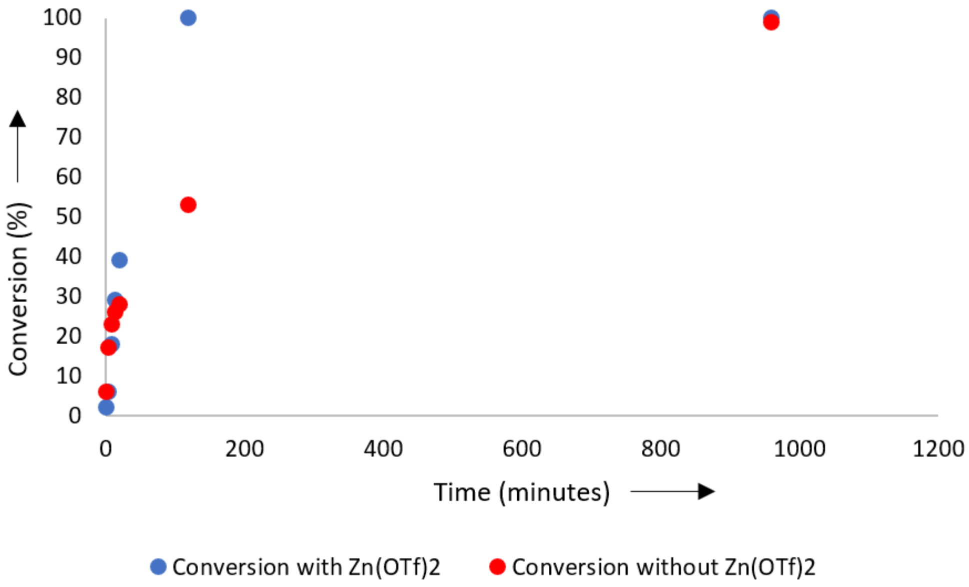

Figure 16 Conversion of starting material with $Zn(OTf)_2$ (blue dots) and without $Zn(OTf)_2$ (red dots) over time using (*S*)-BINAP **L2** as the ligand.

<u>Procedure for monitoring of reaction using (*S*)-BINAP as the ligand with $Zn(OTf)_2$</u>

$[Rh(cod)OH]_2$ (11.4 mg, 0.0250 mmol, 0.025 eq) and (*S*)-BINAP (37.4 mg, 0.06 mmol, 0.06 eq) were added to a flame dried 5 mL round bottom flask, sealed with a rubber septum under an argon atmosphere and dissolved in THF (0.6 mL). This solution was stirred at 60 °C (the solution turned dark brown). After 30 min, this catalyst solution was added to a flame dried 5 mL round bottom flask containing phenylboronic acid (3.0 mmol, 3.0 eq), dimethyl 2-vinylcyclopropane-1,1-dicarboxylate **1** (184.2, 1.0 mmol, 1.0 eq), Cs_2CO_3 (325.8 mg, 1.0 mmol, 1 eq) and $Zn(OTf)_2$ (72.7 mg, 0.2 mmol, 0.20 eq) via syringe, and the flask rinsed with THF (0.4 mL) and added. The resulting dark brown reaction mixture was stirred at 50 °C, with ≈0.05 mL aliquots taken at 2, 5, 10, 15, 20 minutes and 2 hours, and the reaciton mixture stirred for 18 h and the final aliquot (0.5 mL) taken.

The aliquots were mixed with Et_2O and filtered through a plug of silica and washed with Et_2O (x3) and the filtrate collected and concentrated *in vacuo*. Conversion determined by ^{1}H NMR spectroscopy, with CH_2Br_2 used as an internal standard.

Procedure for monitoring of reaction using (*S*)-BINAP as the ligand without $Zn(OTf)_2$

$[Rh(cod)OH]_2$ (11.4 mg, 0.0250 mmol, 0.025 eq) and (*S*)-BINAP (37.4 mg, 0.06 mmol, 0.06 eq) were added to a flame dried 5 mL round bottom flask, sealed with a rubber septum under an argon atmosphere and dissolved in THF (0.6 mL). This solution was stirred at 60 °C (the solution turned dark brown). After 30 min, this catalyst solution was added to a flame dried 5 mL round bottom flask containing phenylboronic acid (3.0 mmol, 3.0 eq), dimethyl 2-vinylcyclopropane-1,1-dicarboxylate **1** (184.2, 1.0 mmol, 1.0 eq) and Cs_2CO_3 (325.8 mg, 1.0 mmol, 1 eq) via syringe, and the flask rinsed with THF (0.4 mL) and added. The resulting dark brown reaction mixture was stirred at 50 °C, with ≈0.05 mL aliquots taken at 2, 5, 10, 15, 20 minutes and 2 hours, and the reaciton mixture stirred for 18 h and the final aliquot (0.5 mL) taken.

The aliquots were mixed with Et_2O and filtered through a plug of silica and washed with Et_2O (x3) and the filtrate collected and concentrated *in vacuo*. Conversion determined by ^{1}H NMR spectroscopy, with CH_2Br_2 used as an internal standard.

7.4 Procedures from Mechanistic Studies

Procedure for mixing **L1** with $[Rh(cod)OH]_2$ in Toluene/THF/THP

Walphos-SL-W003-1 (**L1**; 20 mg, 0.0298 mmol, 1 equiv.) and $[Rh(cod)OH]_2$ (6.8 mg, 0.0149 mmol, 0.5 equiv.) were added to a flame dried 5 mL round bottom flask, sealed with a rubber septum under an argon atmosphere and dissolved in the appropriate solvent (0.5 mL). This solution was stirred at 60 °C (the solution turned dark brown). After 30 min, this catalyst solution was cooled to r.t. and transferred to an NMR tube under argon and the $^{31}P\{^1H\}$ NMR measured.

Procedure for mixing **L1** with $[Rh(cod)OH]_2$ in THP over 5 hours

Walphos-SL-W003-1 (**L1**; 20 mg, 0.0298 mmol, 1 equiv.) and $[Rh(cod)OH]_2$ (6.8 mg, 0.0149 mmol, 0.5 equiv.) were added to a flame dried 5 mL round bottom flask, sealed with a rubber septum under an argon atmosphere and dissolved in THP (0.5 mL). This solution was stirred at 60 °C (the solution turned dark brown). After 1h, 3h and 5h, 0.1 mL of catalyst solution was transferred to an NMR tube under argon, and THP (0.4 mL) was added and the $^{31}P\{^1H\}$ NMR measured.

Procedure for mixing $PhB(OH)_2$ and $Zn(OTf)_2$ after standard complexation

$[Rh(cod)OH]_2$ (6.8 mg, 0.0149 mmol, 0.5 equiv.) and Walphos-SL-W003-1 **L1** (20 mg, 0.0298 mmol, 1 equiv.) were added to a flame dried 5 mL round bottom flask, sealed with a rubber septum under an argon atmosphere and dissolved in THP (0.3 mL). This solution was stirred at 60 °C (the solution turned dark brown), and after 30 min the solution was added to an NMR tube under argon. Phenylboronic acid (10.9 mg, 0.0895 mmol, 3.0 eq) was added to the NMR tube and the $^{31}P\{^1H\}$ NMR measured. $Zn(OTf)_2$ (10.8 mg, 0.0298 mmol, 1 equiv.) was added to the NMR tube under argon and the $^{31}P\{^1H\}$ NMR measured.

Procedure for complex formation via addition of **L1** to $Zn(OTf)_2$ then addition of $[Rh(cod)OH]_2$

$Zn(OTf)_2$ (16.2 mg, 0.0447 mmol, 1 equiv.) and Walphos-SL-W003-1 **L1** (30 mg, 0.0447 mmol, 1 equiv.) were added to a flame dried 5 mL round bottom flask, sealed with a rubber septum under an argon atmosphere and dissolved in THP (0.4 mL). This solution was stirred at 60 °C (the solution turned dark orange). After 30 min, this catalyst solution was added to an NMR tube under argon, the flask rinsed with 0.1 mL THP and added to the NMR tube. The $^{31}P\{^1H\}$ NMR was measured showing

clean formation of a **L1**-Zinc complex $^{31}P\{^1H\}$ NMR (162 MHz, THP) δ 33.81 (d, *J* = 17.5 Hz), -14.62 (d, *J* = 17.8 Hz). The cap was removed from the NMR tube and $[Rh(cod)OH]_2$ (10.2 mg, 0.0224 mmol, 0.5 equiv.) was quickly added and the tube capped. The tube was shaken vigorously and the $^{31}P\{^1H\}$ NMR was measured.

$^{31}P\{^1H\}$ NMR (202 MHz, THP) δ 37.89 (dd, *J* = 136.3, 25.0 Hz), 16.36 (dd, *J* = 147.6, 23.9 Hz).

Procedure for complex formation using $La(OTf)_3$

$La(OTf)_3$ (26.1 mg, 0.0447 mmol, 1 equiv.) and Walphos-SL-W003-1 **L1** (30 mg, 0.0447 mmol, 1 equiv.) were added to a flame dried 5 mL round bottom flask, sealed with a rubber septum under an argon atmosphere and dissolved in THP (0.4 mL). This solution was stirred at 60 °C (the solution turned dark orange). After 30 min, $[Rh(cod)OH]_2$ (10.2 mg, 0.0224 mmol, 0.5 equiv.) was quickly added and the flask shaken for a minute. The dark solution was transferred to an NMR tube under argon and the $^{31}P\{^1H\}$ NMR was measured.

Procedure for complex formation using $ZnBr_2$

$ZnBr_2$ (10.1 mg, 0.0447 mmol, 1 equiv.) and Walphos-SL-W003-1 (30 mg, 0.0447 mmol, 1 equiv.) were added to a flame dried 5 mL round bottom flask, sealed with a rubber septum under an argon atmosphere and dissolved in THP (0.4 mL). This solution was stirred at 60 °C (the solution turned dark orange). After 30 min, $[Rh(cod)OH]_2$ (10.2 mg, 0.0224 mmol, 0.5 equiv.) was quickly added and the flask shaken for a minute. The dark solution was transferred to an NMR tube under argon and the $^{31}P\{^1H\}$ NMR was measured.

$^{31}P\{^1H\}$ NMR (162 MHz, THP) δ 69.20 (dd, *J* = 194.7, 41.6 Hz), 44.45 (dd, *J* = 207.4, 42.4 Hz.

Procedure for complex formation via addition of **L2** to $Zn(OTf)_2$ then addition of $[Rh(cod)OH]_2$

$Zn(OTf)_2$ (16.2 mg, 0.0447 mmol, 1 equiv.) and (*S*)-BINAP **L2** (27.8 mg, 0.0447 mmol, 1 equiv.) were added to a flame dried 5 mL round bottom flask, sealed with a rubber septum under an argon atmosphere and dissolved in THP (0.4 mL). This solution was stirred at 60 °C (the solution turned dark orange). After 30 min, $[Rh(cod)OH]_2$ (10.2 mg, 0.0224 mmol, 0.5 equiv.) was quickly added and the mixture transferred to an NMR tube under argon and the $^{31}P\{^1H\}$ NMR was measured.

$^{31}P\{^1H\}$ NMR (162 MHz, THP) δ 25.25 (d, J = 146.4 Hz).

Procedure for mixing **L1** with $[Rh(cod)OMe]_2$ in THP for 30 minutes

Walphos-SL-W003-1 (**L1**; 30 mg, 0.0447 mmol, 1 equiv.) and $[Rh(cod)OMe]_2$ (10.8 mg, 0.0224 mmol, 0.5 equiv.) were added to a flame dried 5 mL round bottom flask, sealed with a rubber septum

under an argon atmosphere and dissolved in THP (0.5 mL). This solution was stirred at 60 °C (the solution turned dark brown). After 30 minutes, 0.1 mL of catalyst solution was transferred to an NMR tube under argon, and THP (0.4 mL) was added and the $^{31}P\{^{1}H\}$ NMR measured.

Procedure for complex formation via addition of **L1** to $Zn(OTf)_2$ then addition of $[Rh(cod)OMe]_2$

$Zn(OTf)_2$ (16.2 mg, 0.0447 mmol, 1 equiv.) and Walphos-SL-W003-1 **L1** (30 mg, 0.0447 mmol, 1 equiv.) were added to a flame dried 5 mL round bottom flask, sealed with a rubber septum under an argon atmosphere and dissolved in THP (0.4 mL). This solution was stirred at 60 °C (the solution turned dark orange). After 30 min, $[Rh(cod)OMe]_2$ (10.8 mg, 0.0224 mmol, 0.5 equiv.) was quickly added, and the solution transferred to an NMR tube under argon, and the tube capped. The tube was shaken vigorously and the $^{31}P\{^{1}H\}$ NMR was measured.

Procedure for mixing **L1** with $[Rh(coe)_2Cl]_2$ in THP for 30 minutes

Walphos-SL-W003-1 (**L1**; 30 mg, 0.0447 mmol, 1 equiv.) and $[Rh(coe)_2Cl]_2$ (16.0 mg, 0.0224 mmol, 0.5 equiv.) were added to a flame dried 5 mL round bottom flask, sealed with a rubber septum under an argon atmosphere and dissolved in THP (0.5 mL). This solution was stirred at 60 °C (the solution turned brown). After 30 minutes, 0.1 mL of catalyst solution was transferred to an NMR tube under argon, and THP (0.4 mL) was added and the $^{31}P\{^{1}H\}$ NMR measured.

Procedure for addition of **L1** to $Zn(OTf)_2$ then addition of $[Rh(coe)_2Cl]_2$

$Zn(OTf)_2$ (16.2 mg, 0.0447 mmol, 1 equiv.) and Walphos-SL-W003-1 **L1** (30 mg, 0.0447 mmol, 1 equiv.) were added to a flame dried 5 mL round bottom flask, sealed with a rubber septum under an argon atmosphere and dissolved in THP (0.4 mL). This solution was stirred at 60 °C (the solution turned dark orange). After 30 min, $[Rh(coe)_2Cl]_2$ (16.0 mg, 0.0224 mmol, 0.5 equiv.) was quickly added, and the solution transferred to an NMR tube under argon, and the tube capped. The tube was shaken vigorously and the $^{31}P\{^{1}H\}$ NMR was measured.

Procedure for mixing **L1** with $[Rh(C_2H_4)_2Cl]_2$ in THP for 30 minutes

Walphos-SL-W003-1 (**L1**; 30 mg, 0.0447 mmol, 1 equiv.) and $[Rh(C_2H_4)_2Cl]_2$ (8.7 mg, 0.0224 mmol, 0.5 equiv.) were added to a flame dried 5 mL round bottom flask, sealed with a rubber septum under an argon atmosphere and dissolved in THP (0.5 mL). This solution was stirred at 60 °C (the solution turned brown). After 30 minutes, 0.1 mL of catalyst solution was transferred to an NMR tube under argon, and THP (0.4 mL) was added and the $^{31}P\{^{1}H\}$ NMR measured.

Procedure for addition of **L1** to $Zn(OTf)_2$ then addition of $[Rh(C_2H_4)_2Cl]_2$

$Zn(OTf)_2$ (16.2 mg, 0.0447 mmol, 1 equiv.) and Walphos-SL-W003-1 **L1** (30 mg, 0.0447 mmol, 1 equiv.) were added to a flame dried 5 mL round bottom flask, sealed with a rubber septum under an argon atmosphere and dissolved in THP (0.4 mL). This solution was stirred at 60 °C (the solution turned dark orange). After 30 min, $[Rh(C_2H_4)_2Cl]_2$ (8.7 mg, 0.0224 mmol, 0.5 equiv.) was quickly added, and the solution transferred to an NMR tube under argon, and the tube capped. The tube was shaken vigorously and the $^{31}P\{^1H\}$ NMR was measured.

Procedure for reaction with 0.25 mol% $[Rh(C_2H_4)_2Cl]_2$

A stock solution was made by adding $[Rh(C_2H_4)_2Cl]_2$ (10.0 mg, 0.025 mmol, 0.025 eq) and Walphos-SL-W003-1 (41.4 mg, 0.06 mmol, 0.06 eq) to a flame dried 5 mL round bottom flask, sealing with a rubber septum under an argon atmosphere and dissolving in THF (2.0 mL). This solution was stirred at r.t. (the solution turned dark brown). After 30 min, 0.2 mL (0.0025 mmol, 0.0025 eq) was added to a flame dried 5 mL round bottom flask containing phenylboronic acid (365.7 mg, 3.0 mmol, 3.0 eq), dimethyl 2-vinylcyclopropane-1,1-dicarboxylate **1** (184.2, 1.0 mmol, 1.0 eq), Cs_2CO_3 (325.9 mg, 1.0 mmol, 1 eq) and $Zn(OTf)_2$ (181.8 mg, 0.5 mmol, 0.50 eq) via syringe, and the flask rinsed with THF (0.3 mL) and added. The resulting dark brown reaction mixture was stirred at 50 °C for 18 h.

The reaction mixture was cooled to room temperature and Et_2O added. The reaction mixture was filtered through a plug of silica and washed with Et_2O (x3) and the filtrate collected and concentrated *in vacuo*. The crude product was purified by using silica gel chromatography (0 to 20% EtOAc in hexane) to afford **3a** in 80% yield and 94% ee.

8. X-ray crystallographic analysis

3a was derivatized to **11** (**Figure 17**). The absolute stereochemistry of **11** determined by x-ray crystallographic analysis of a single crystal of **11**. The absolute configuration of **3a**, and the absolute configuration of all other examples in this paper was assigned in analogy to **11**.

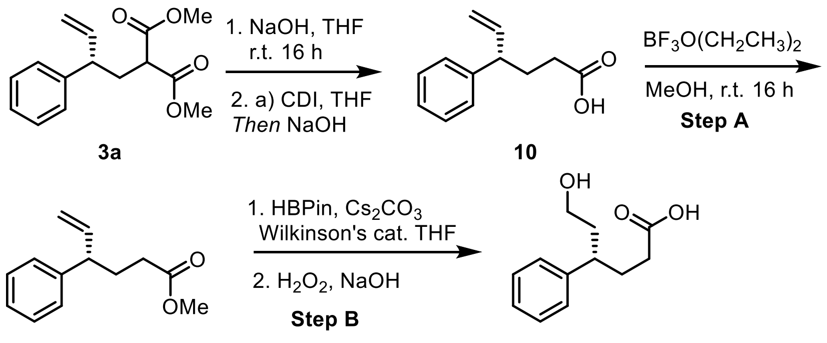

Figure 17 Derivatization of **3a** to form **11**

Step A:

10 (486 mg, 2.55 mmol, 1 equiv.) was dissolved in MeOH (10 mL) and $BF_3O(CH_2CH_3)_2$ was added and the reaction mixture stirred at r.t. for 16 hours. After consumption of starting material the solvent was removed *in vacuo*. Taken forward without further purification to the next step.

Step B:

Wilkinson's catalyst and Cs_2CO_3 were added to a flame dried rbf under argon. Anhydrous THF (3.0 mL) was added and the reaction mixture stirred at r.t. for 5 minutes. **S-6** (435 mg, 2.13 mmol, 1 equiv.) was added in 1 mL THF. HBpin (0.62 mL, 4.26 mmol, 2 equiv.) was added dropwise to the mixture and the reaction mixture stirred at r.t. overnight.

NaOH (11.8 mL) and H_2O_2 was added at 0 °C and the mixture allowed to warm to room temperature and stirred for 16 hours.

The mixture was quenched with $Na_2S_2O_3$ and the aqueous extracted with EtOAc (x3). The combined organics were washed with brine, dried ($MgSO_4$) and the solvent removed *in vacuo* to give crude **11**. The residue purified by silica gel chromatography (hexane/EtOAc = 50/50) to yield product (*S*)-6-hydroxy-4-phenylhexanoic acid as a white solid.

Single crystal suitable for X-ray crystallographic analysis were obtained from a mixture of hexane/DCM/Et_2O. Other characterisation date of **11** was consistent with that reported in the literature.[24]

Low temperature single crystal X-ray diffraction data were collected using a (Rigaku) Oxford Diffraction SuperNova diffractometer for **11a** at 150 K and a Rigaku Synergy-DW diffractometer (EP/V028995/1) for **11b** at 100 K. In all cases, Cu-Kα (λ = 1.54184 Å) radiation was used and the instrument was equipped with a nitrogen gas Oxford Cryosystems Cryostream unit. Raw frame data were reduced using CrysAlisPro and the structures were solved using 'Superflip' [L. Palatinus and G. Chapuis, J. Appl. Cryst., 2007, 40, 786-790.] before refinement with CRYSTALS [(a) P. Parois, R.I. Cooper and A.L. Thompson, Chem. Cent. J., 2015, 9:30. (b) R.I. Cooper, A.L. Thompson and D.J. Watkin, J. Appl. Cryst. 2010, 43, 1100-1107.] as per the SI (CIF). Crystallographic data have been deposited with the Cambridge Crystallographic Data Centre as supplementary publication no. CCDC 2311390-91 and can be obtained via www. ccdc.cam.ac.uk/data_request/cif.

Crystals for **11** were collected twice (a and b) as it became clear during refinement that the structure contained positional disorder that involved the chiral centre. Both crystals contained similar disorder but in both cases the Flack parameter were determined well.

Table 1. Crystal data and structure refinement for **11a**.

CCDC number	2311390	
Empirical formula	C12 H16 O3	
Formula weight	208.26	
Temperature	150 K	
Wavelength	1.54184 Å	
Crystal system / Space group	Monoclinic	P 2_1
Unit cell dimensions	a = 5.8248(3) Å	α= 90°.
	b = 7.6176(4) Å	β= 99.075(4)°.
	c = 12.8894(5) Å	γ = 90°.
Volume	564.76(5) Å^3	
Z	2	
Crystal size	0.20 x 0.18 x 0.03 mm^3	
Independent reflections	2305 [R(int) = 0.033]	
Completeness to theta = 74.681°	99.8 %	
Absorption correction	Semi-empirical from equivalents	
Max. and min. transmission	0.98 and 0.86	
Refinement method	Full-matrix least-squares on F^2	
Data / restraints / parameters	2305 / 91 / 153	
Goodness-of-fit on F^2	0.9972	
Final R indices [I>2sigma(I)]	R1 = 0.0421, wR2 = 0.1109	
R indices (all data)	R1 = 0.0444, wR2 = 0.1140	
Absolute structure parameter	0.11(10)	
Largest diff. peak and hole	0.27 and -0.14 e.Å^{-3}	

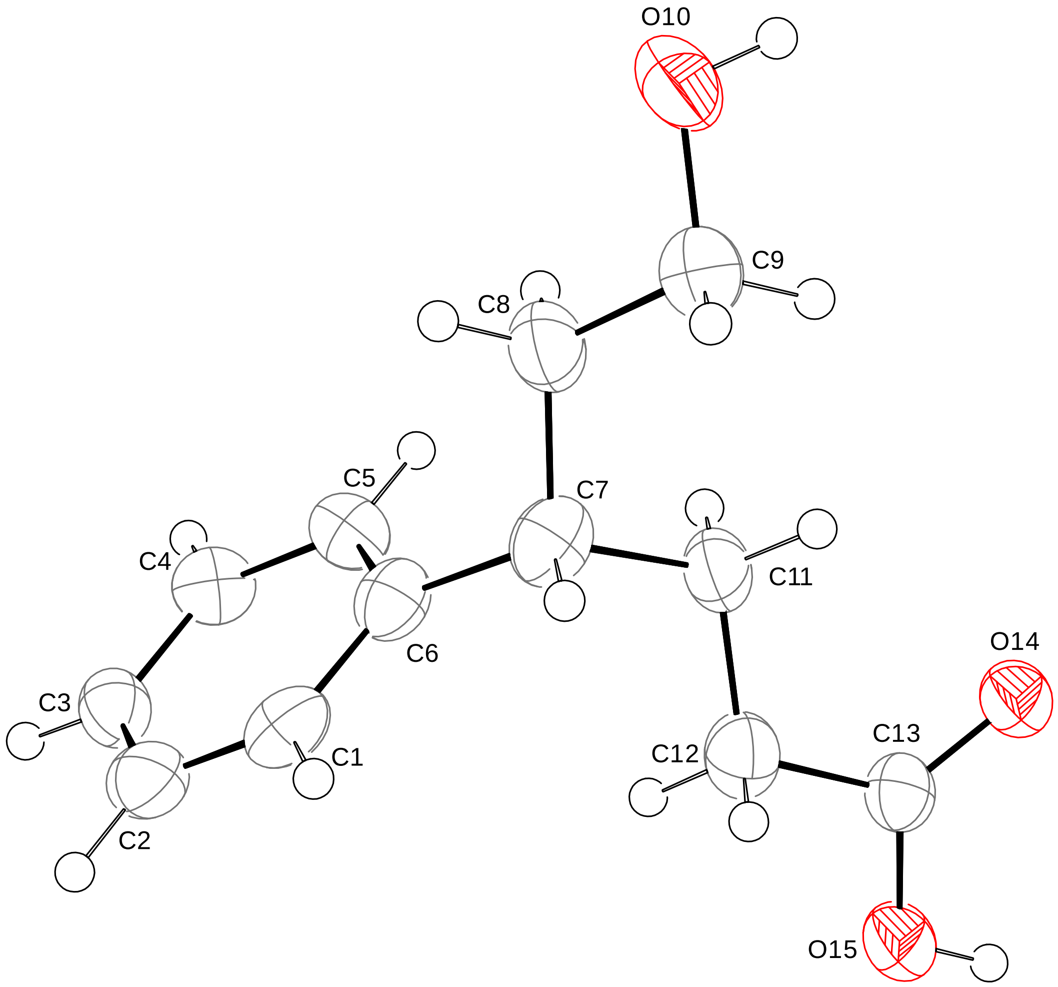

Displacement ellipsoids plot of **11a** drawn at 50% probability. Any disorder has been removed for clarity.

Table 2. Crystal data and structure refinement for **11b**.

CCDC number	2311391	
Empirical formula	C12 H16 O3	
Formula weight	208.26	
Temperature	100 K	
Wavelength	1.54184 Å	
Crystal system / Space group	Monoclinic	P 2_1
Unit cell dimensions	a = 5.79150(10) Å	α= 90°.
	b = 7.59010(10) Å	β= 99.190(2)°.
	c = 12.8954(2) Å	γ = 90°.
Volume	559.581(15) Å^3	
Z	2	
Crystal size	0.12 x 0.10 x 0.04 mm^3	
Independent reflections	2279 [R(int) = 0.031]	

Completeness to theta = 74.451°	99.5 %
Absorption correction	Semi-empirical from equivalents
Max. and min. transmission	0.97 and 0.80
Refinement method	Full-matrix least-squares on F^2
Data / restraints / parameters	2279 / 11 / 166
Goodness-of-fit on F^2	0.9968
Final R indices [I>2sigma(I)]	R1 = 0.0513, wR2 = 0.1352
R indices (all data)	R1 = 0.0535, wR2 = 0.1373
Absolute structure parameter	-0.02(7)
Extinction coefficient	23(5)
Largest diff. peak and hole	0.05 and -0.05 e.Å^{-3}

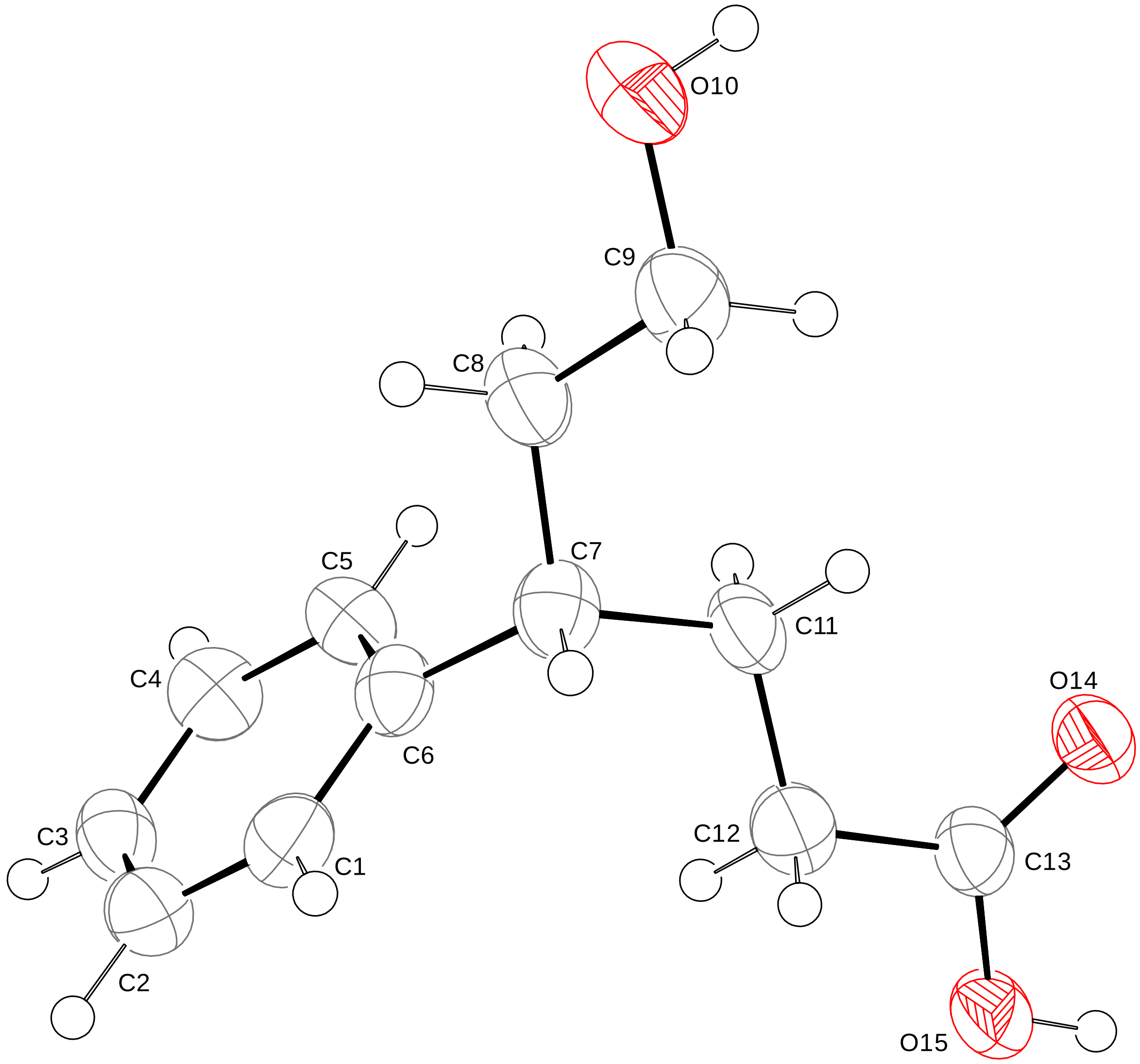

Displacement ellipsoids plot of **11b** drawn at 50% probability. Any disorder has been removed for clarity.

9. NMR Spectra

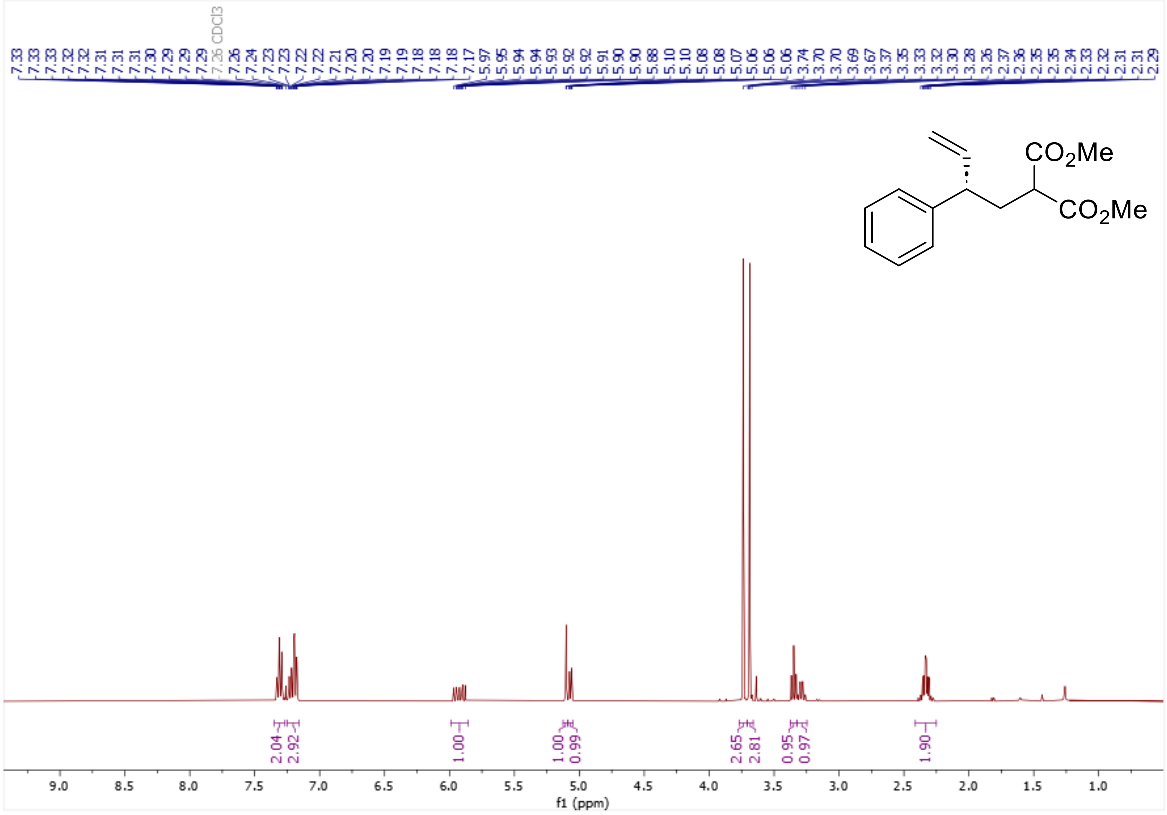

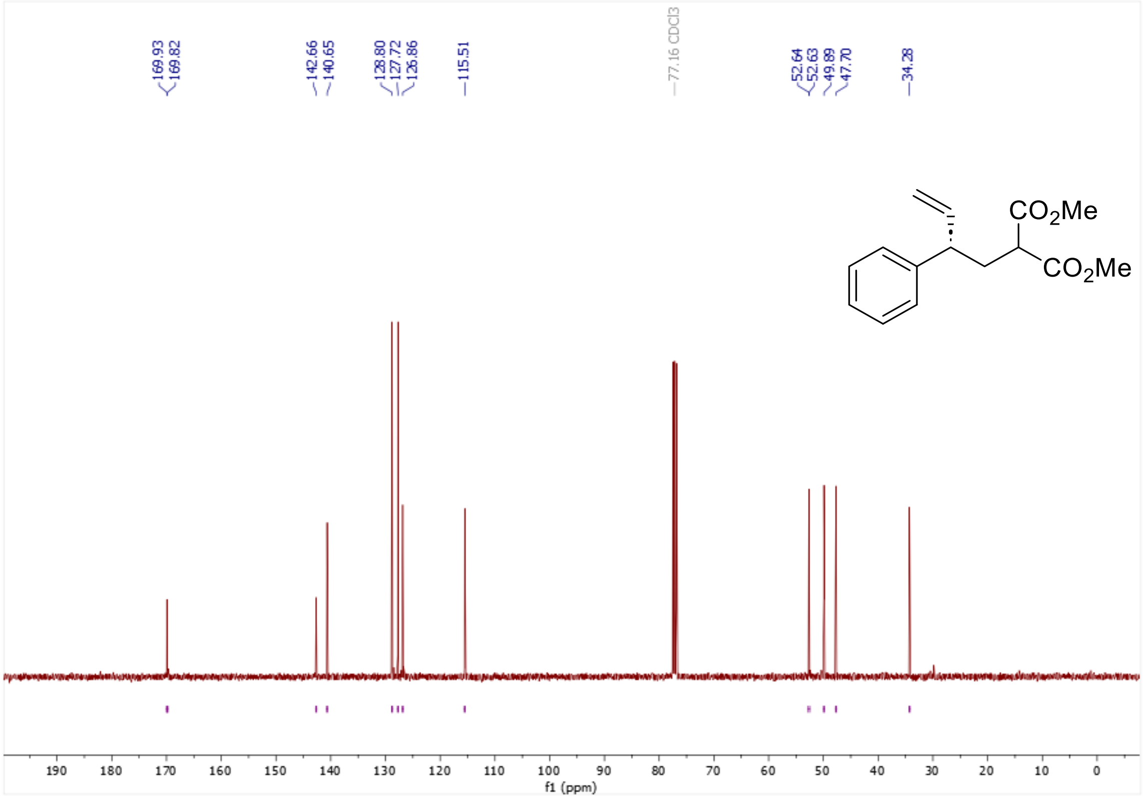

(top) ^{1}H NMR (400 MHz) and (bottom) ^{13}C NMR (101 MHz) spectra of **3a**.

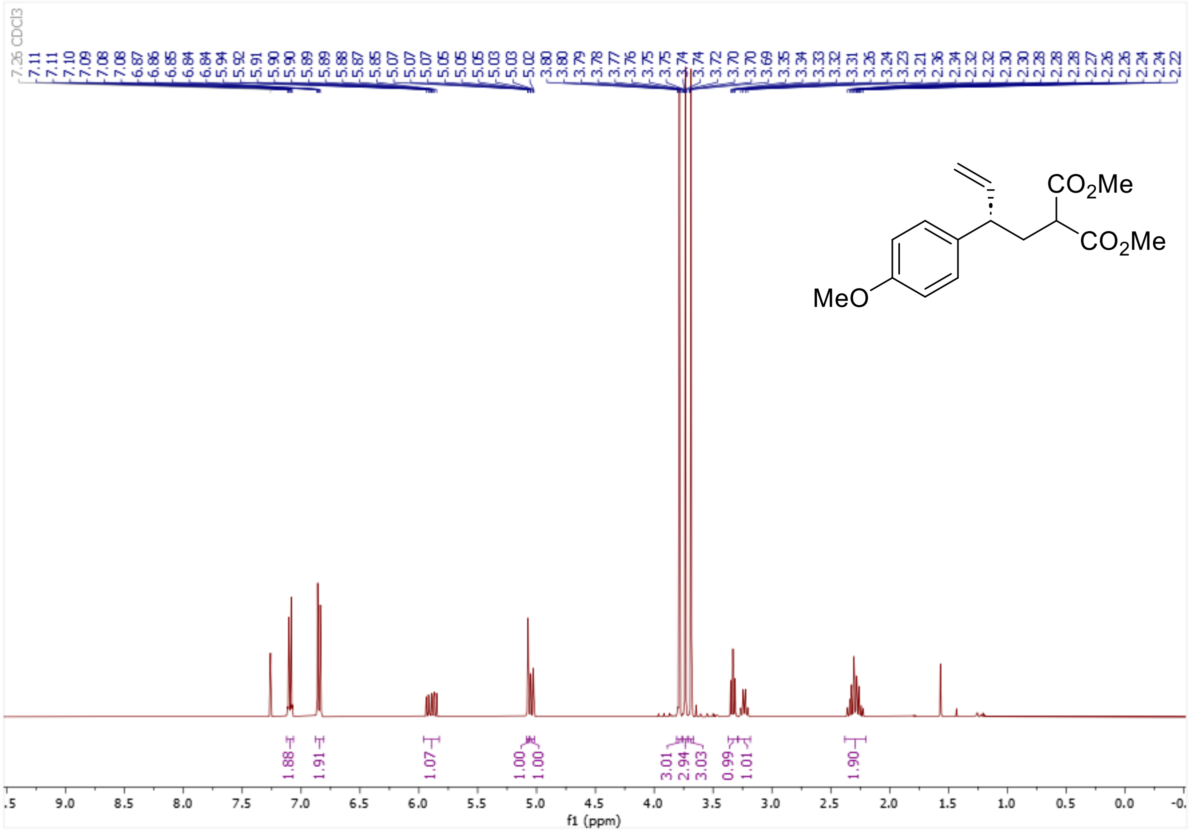

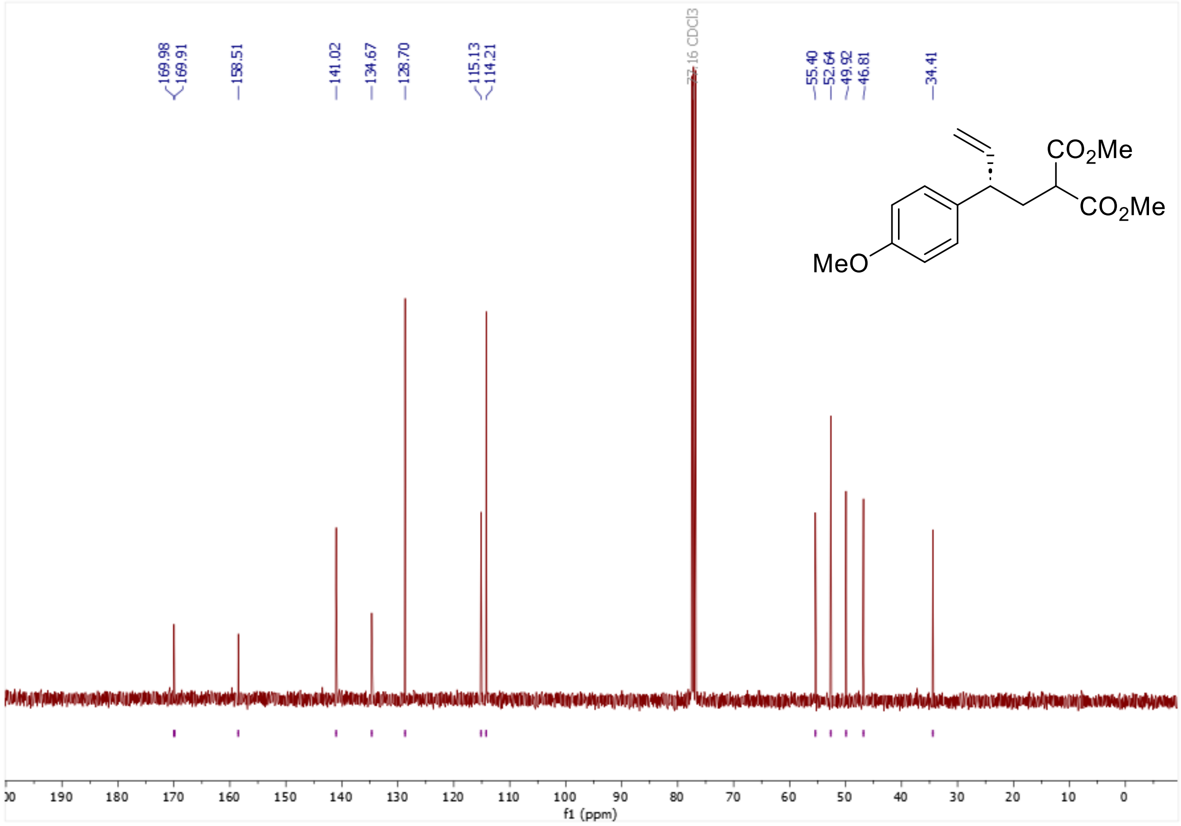

(top) ^{1}H NMR (400 MHz) and (bottom) ^{13}C NMR (101 MHz) spectra of **3b**.

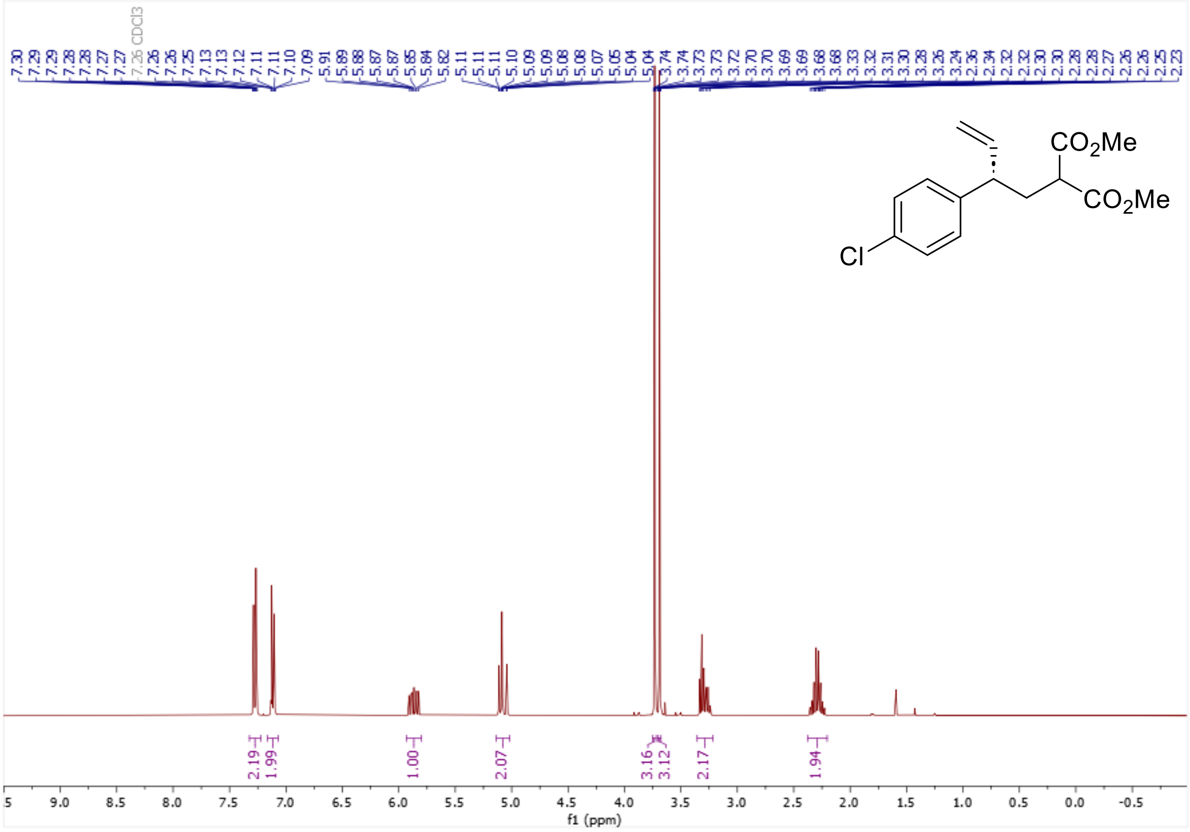

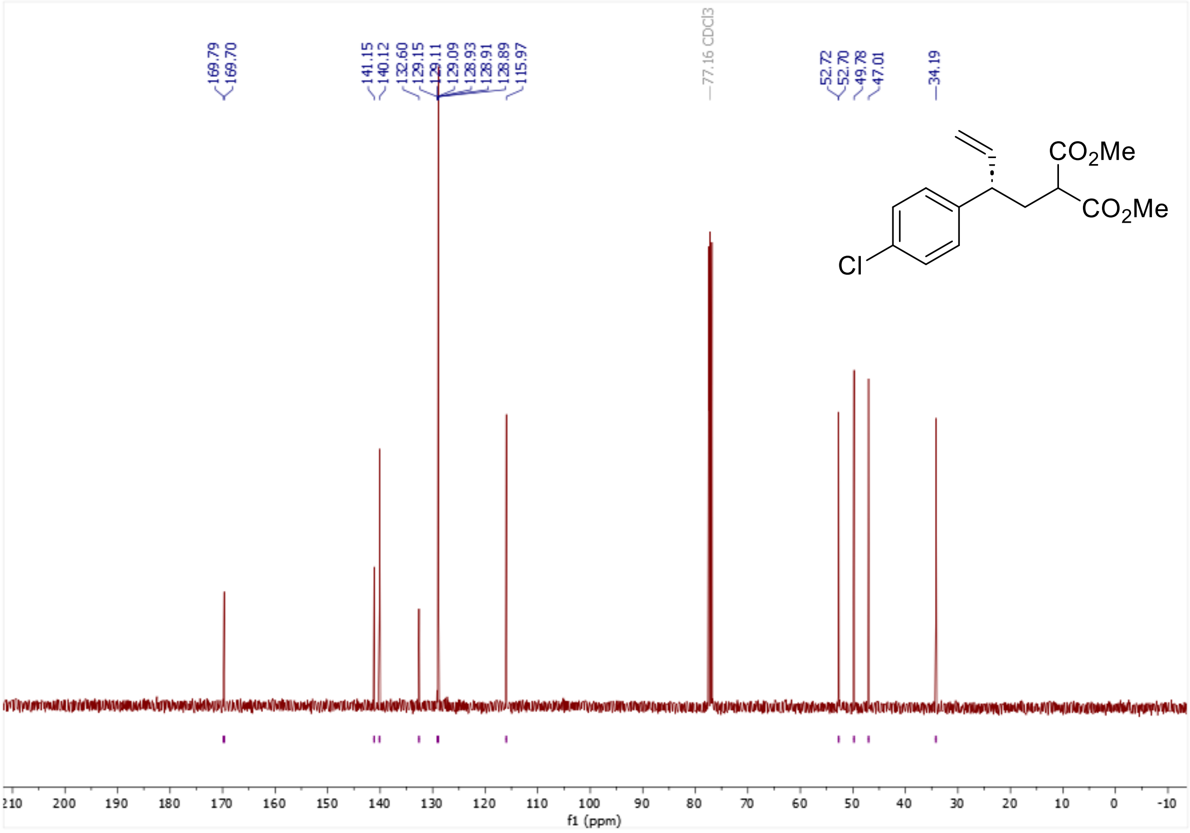

(top) ^{1}H NMR (400 MHz) and (bottom) ^{13}C NMR (101 MHz) spectra of **3c**.

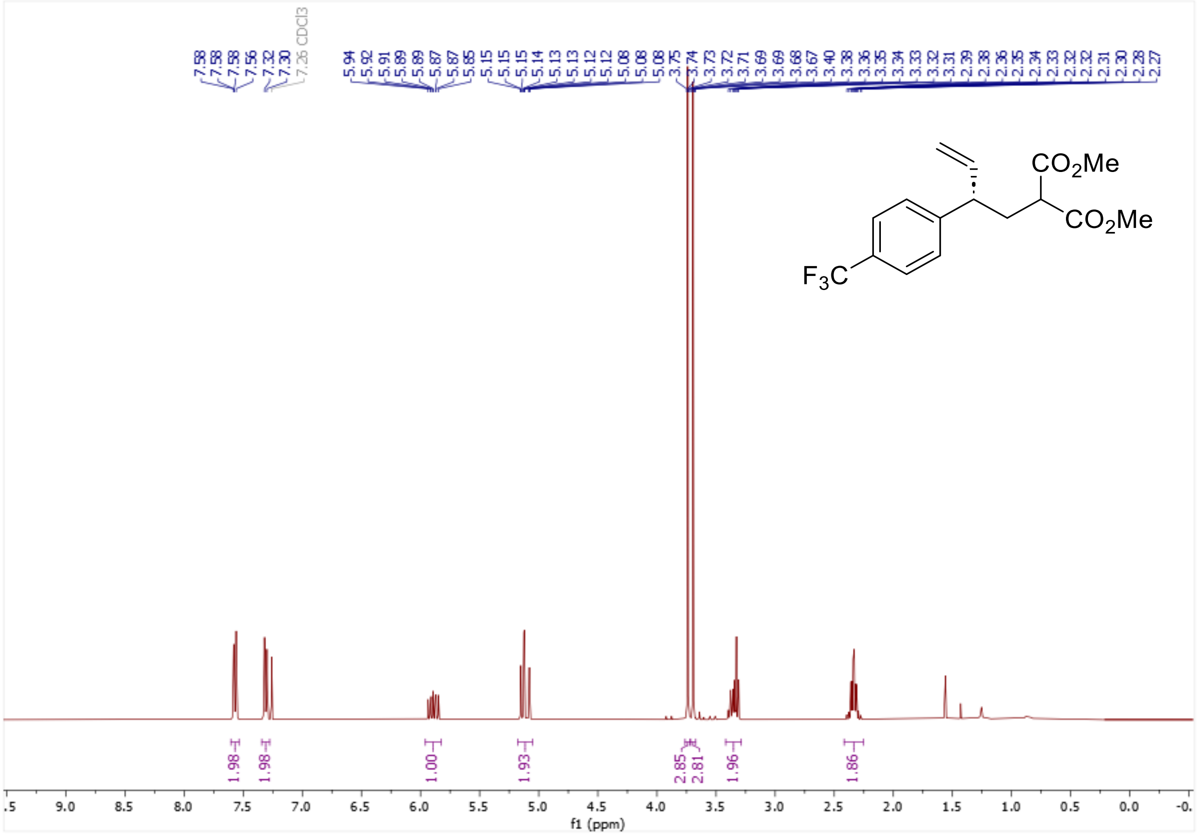

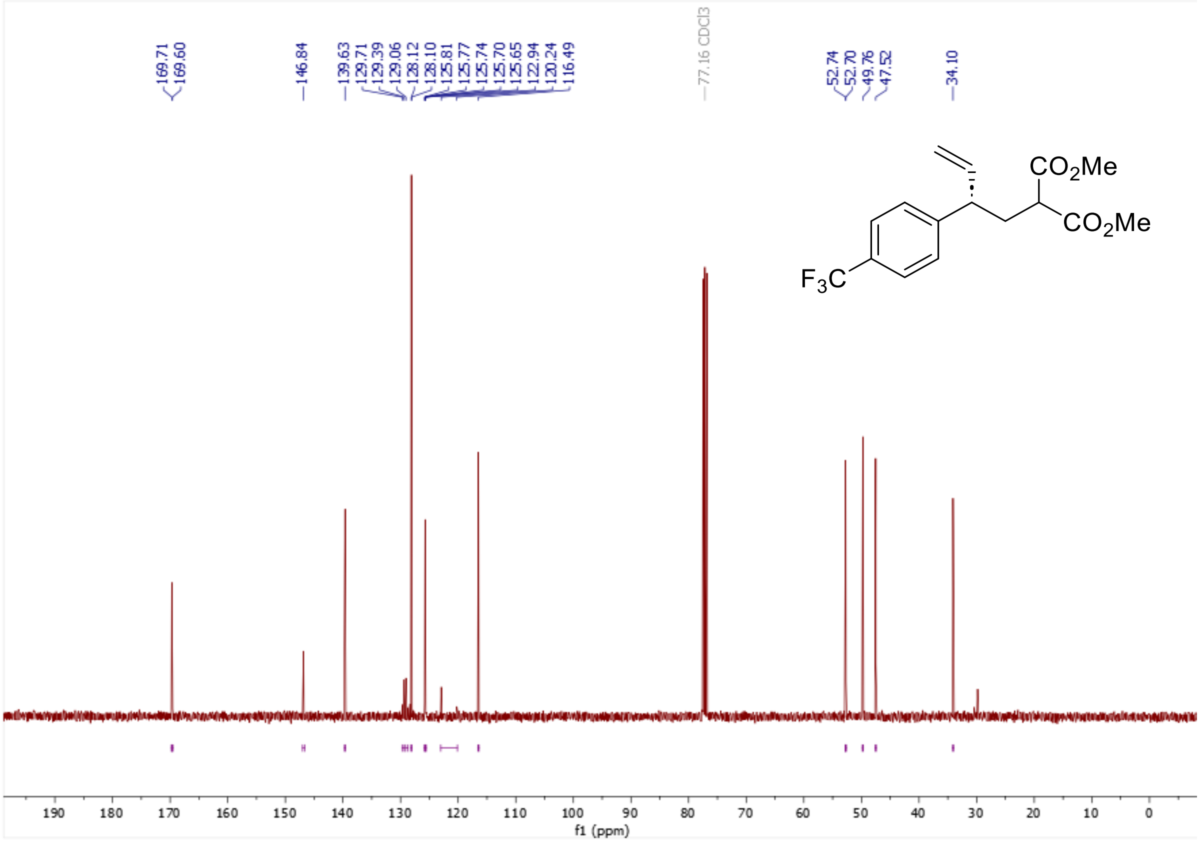

(top) ^{1}H NMR (400 MHz) and (bottom) ^{13}C NMR (101 MHz) spectra of **3d**.

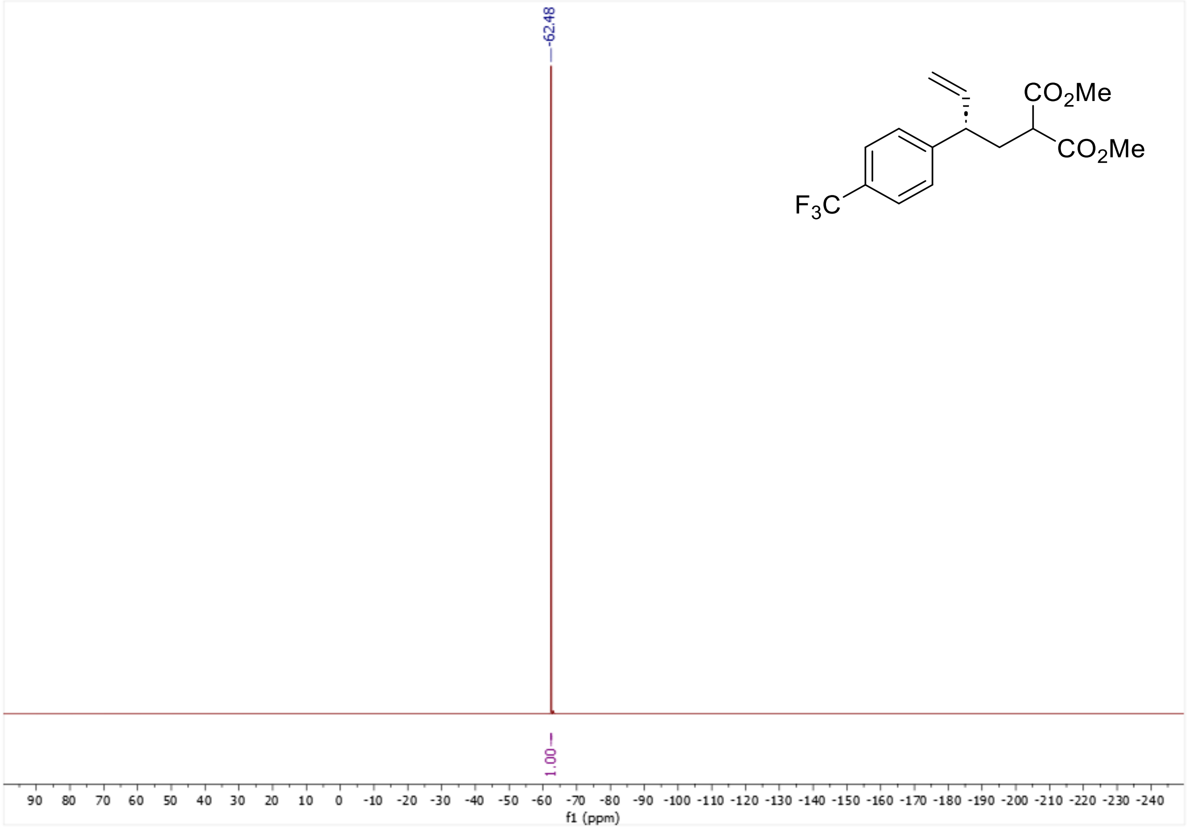

^{19}F NMR (376 MHz) spectra of **3d**.

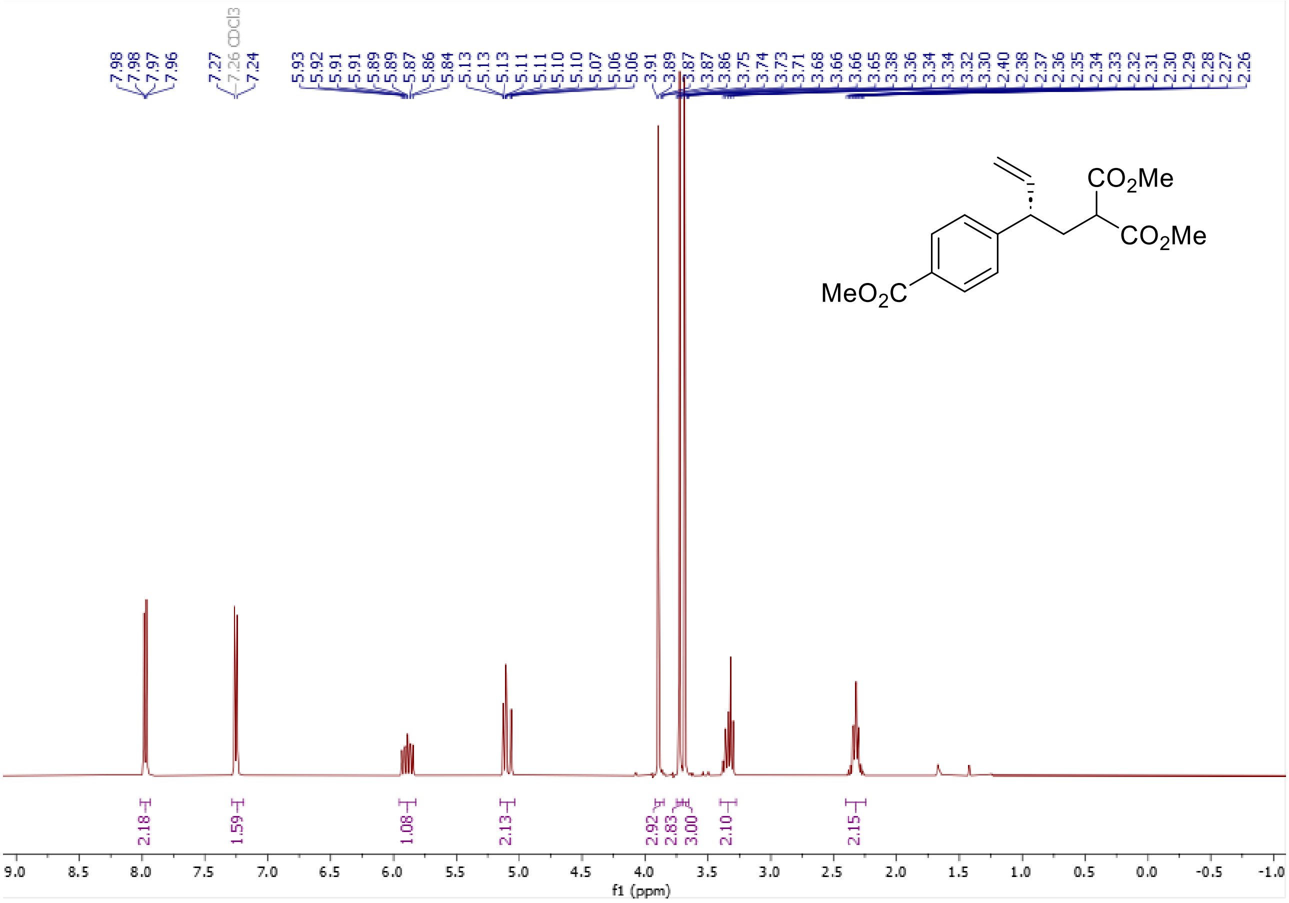

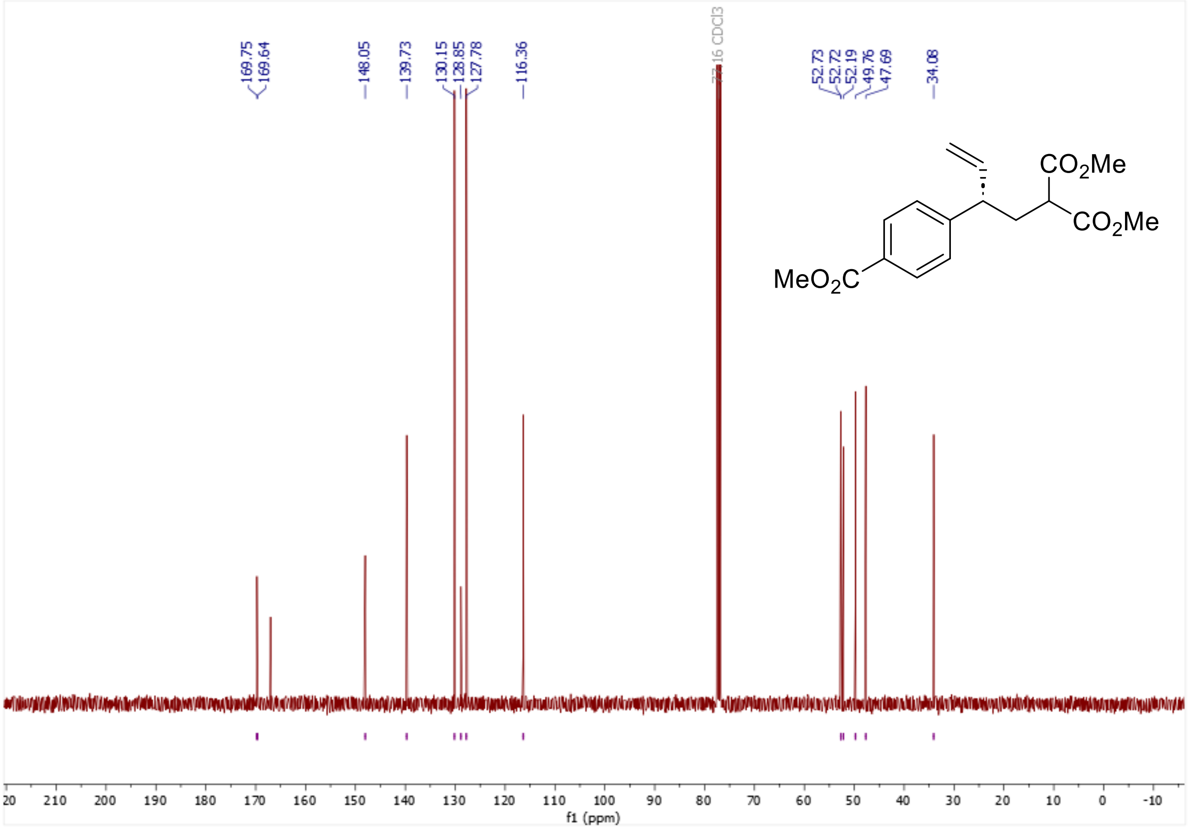

(top) ^{1}H NMR (400 MHz) and (bottom) ^{13}C NMR (101 MHz) spectra of **3e**.

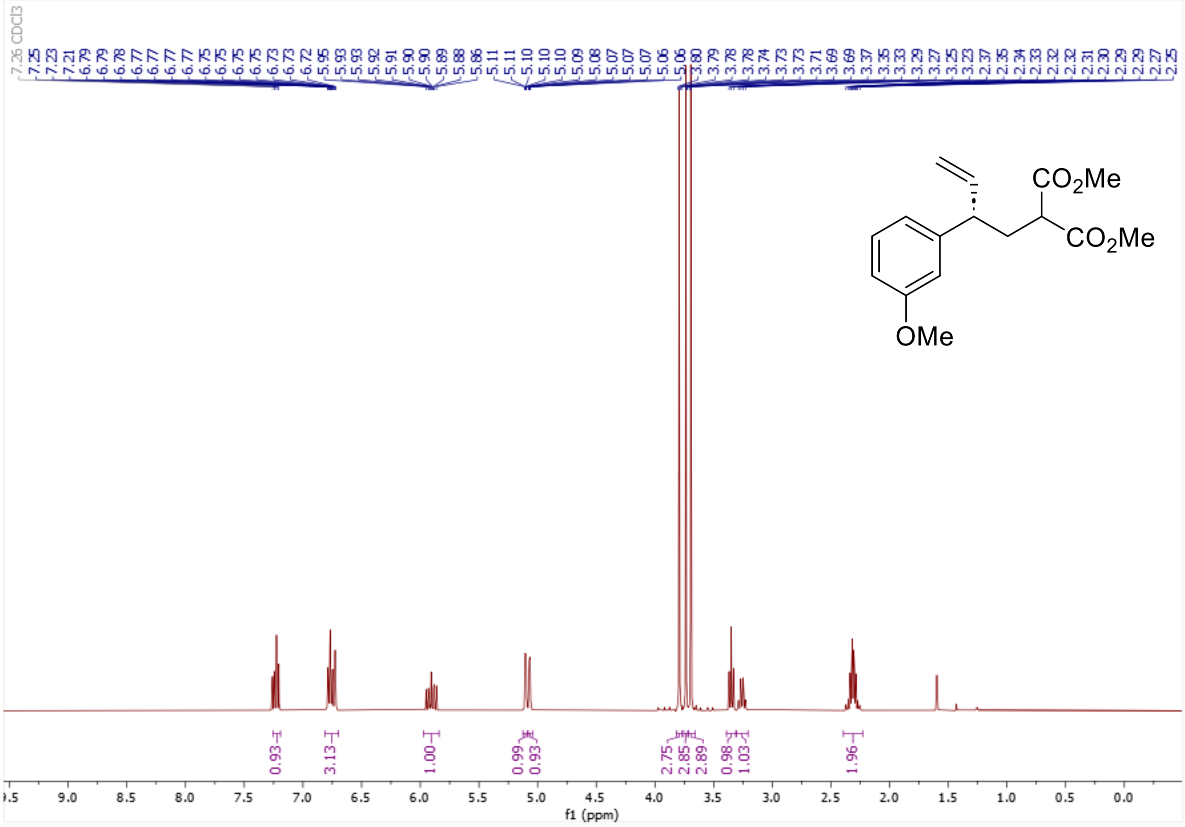

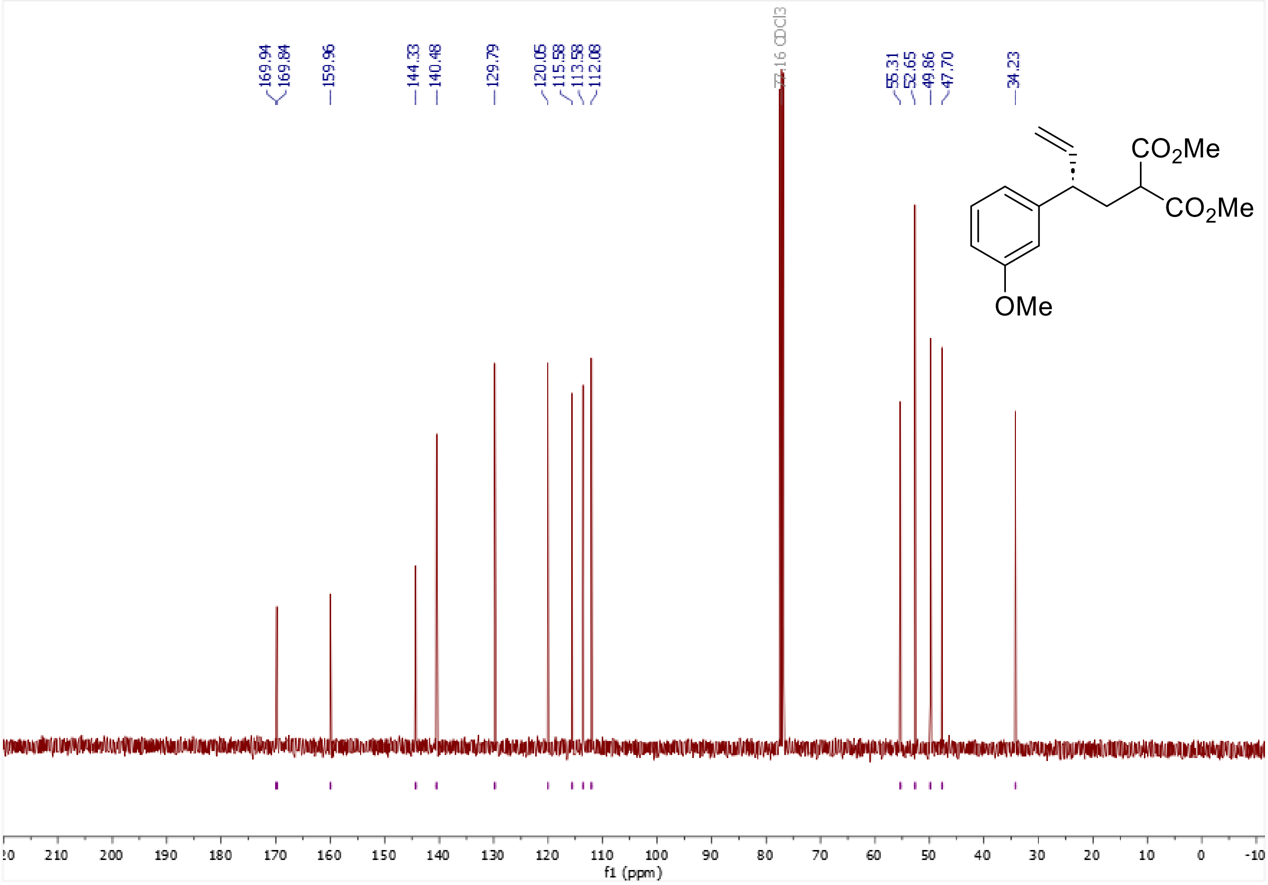

(top) ^{1}H NMR (400 MHz) and (bottom) ^{13}C NMR (101 MHz) spectra of **3f**

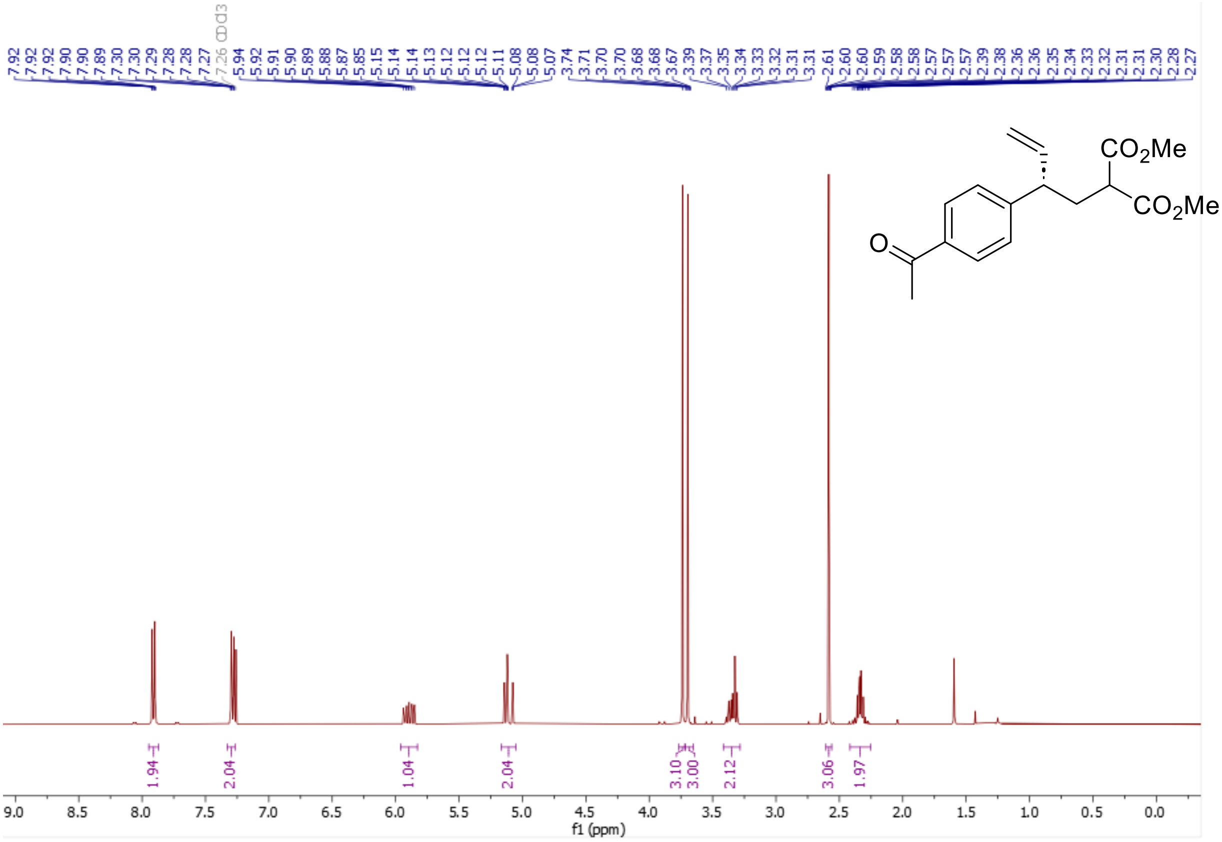

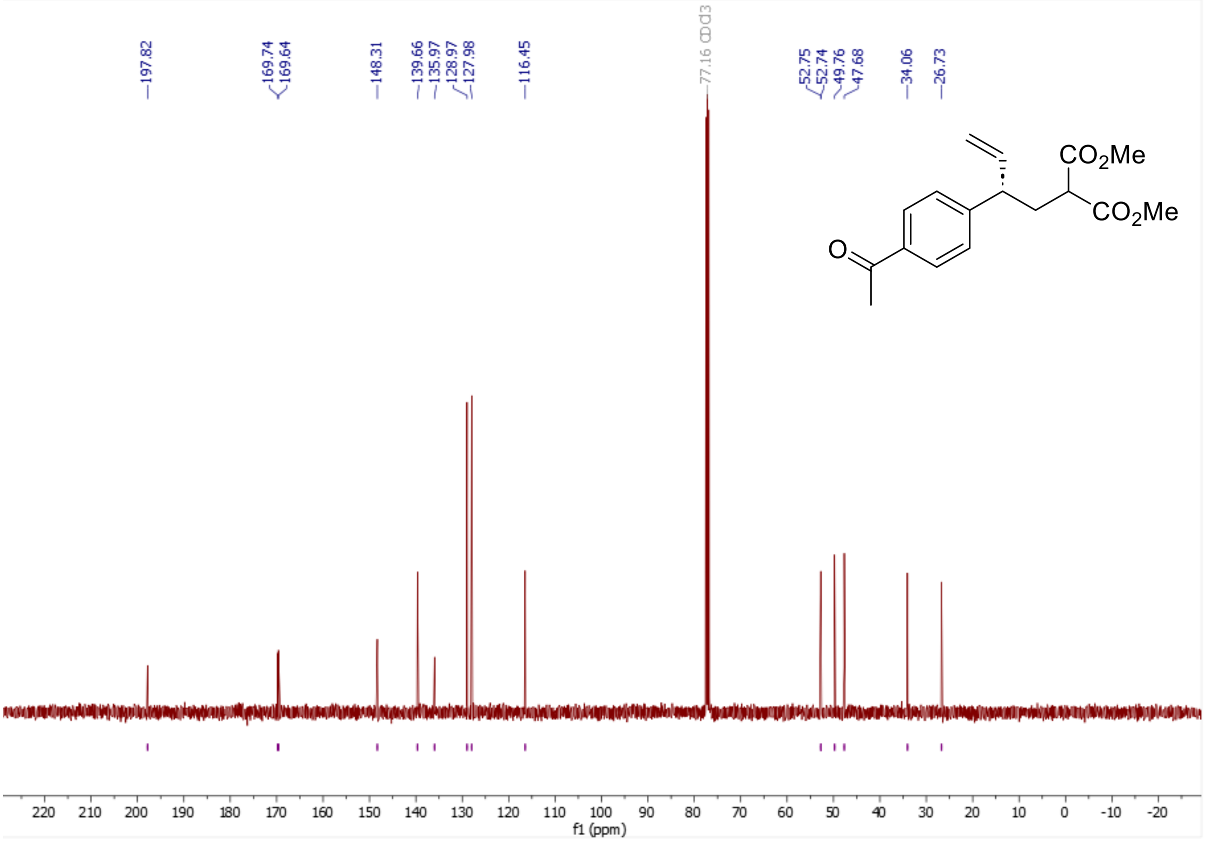

(top) ^{1}H NMR (400 MHz) and (bottom) ^{13}C NMR (101 MHz) spectra of **3g**.

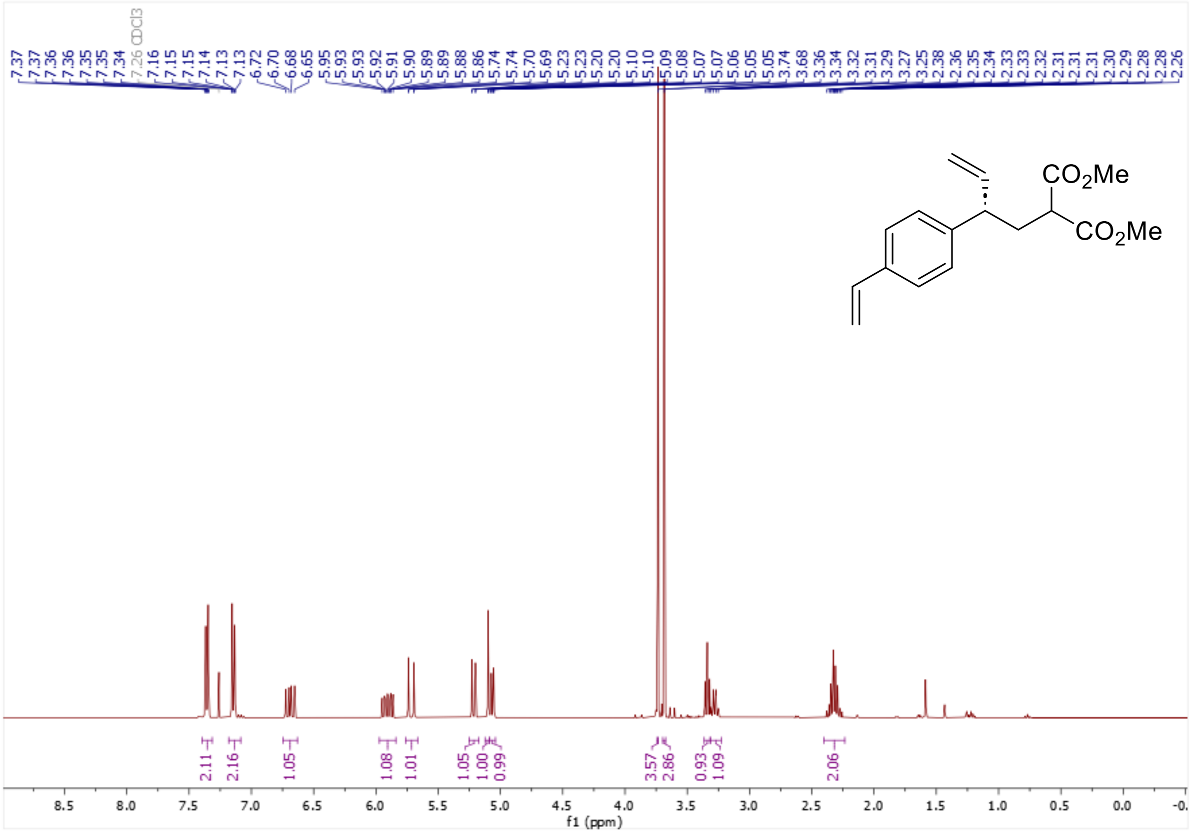

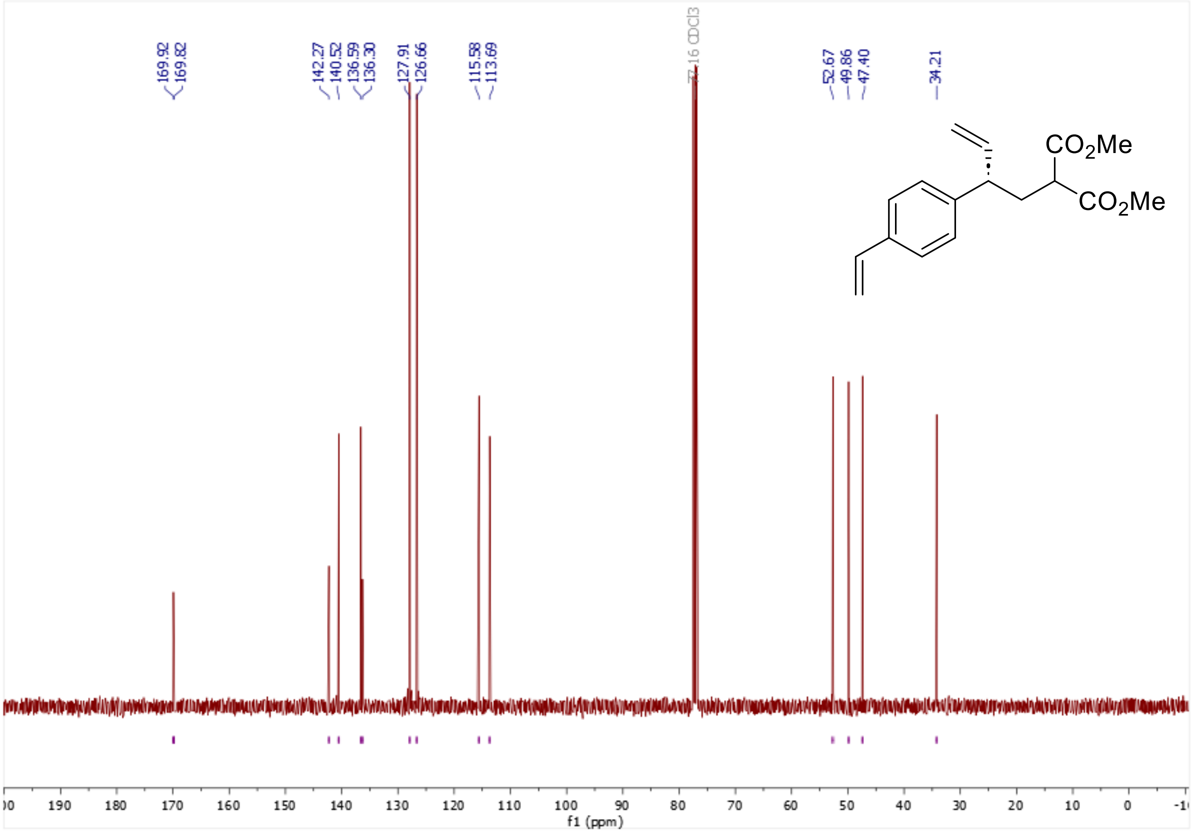

(top) ^{1}H NMR (400 MHz) and (bottom) ^{13}C NMR (101 MHz) spectra of **3h**.

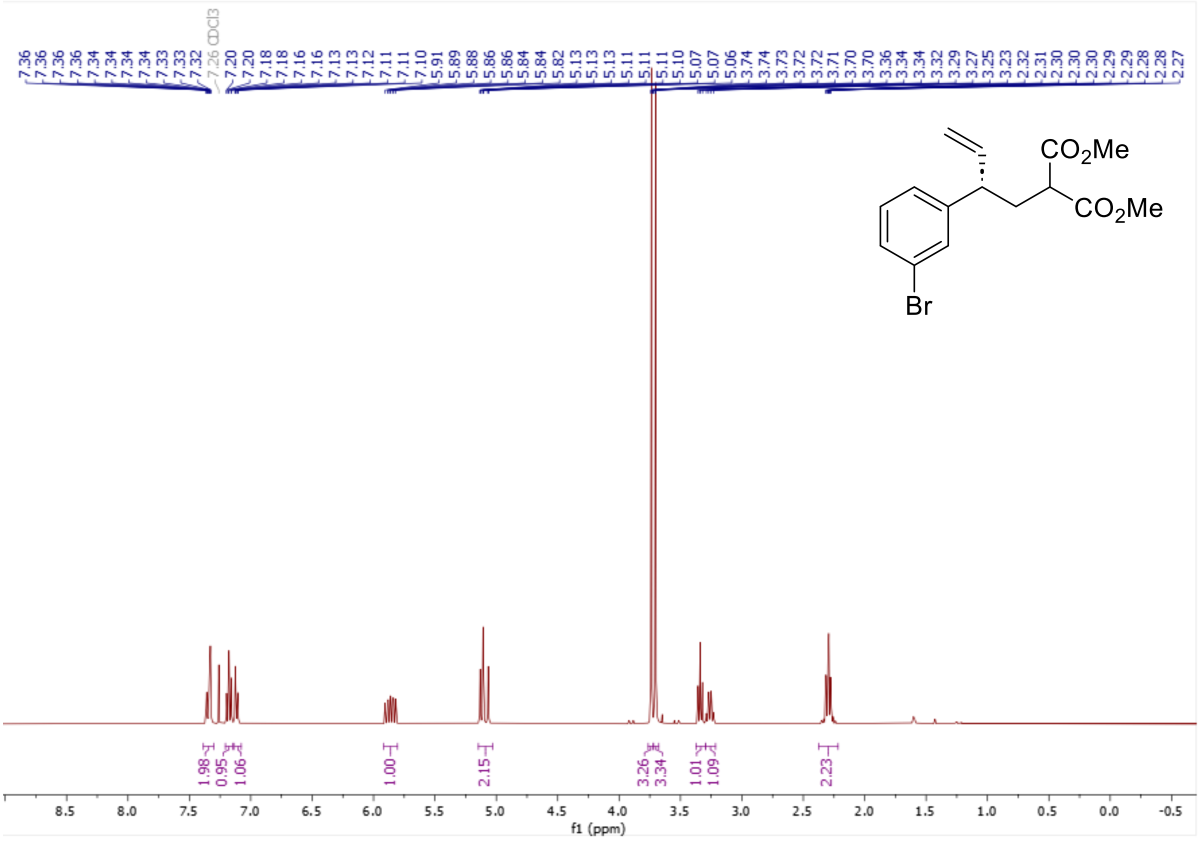

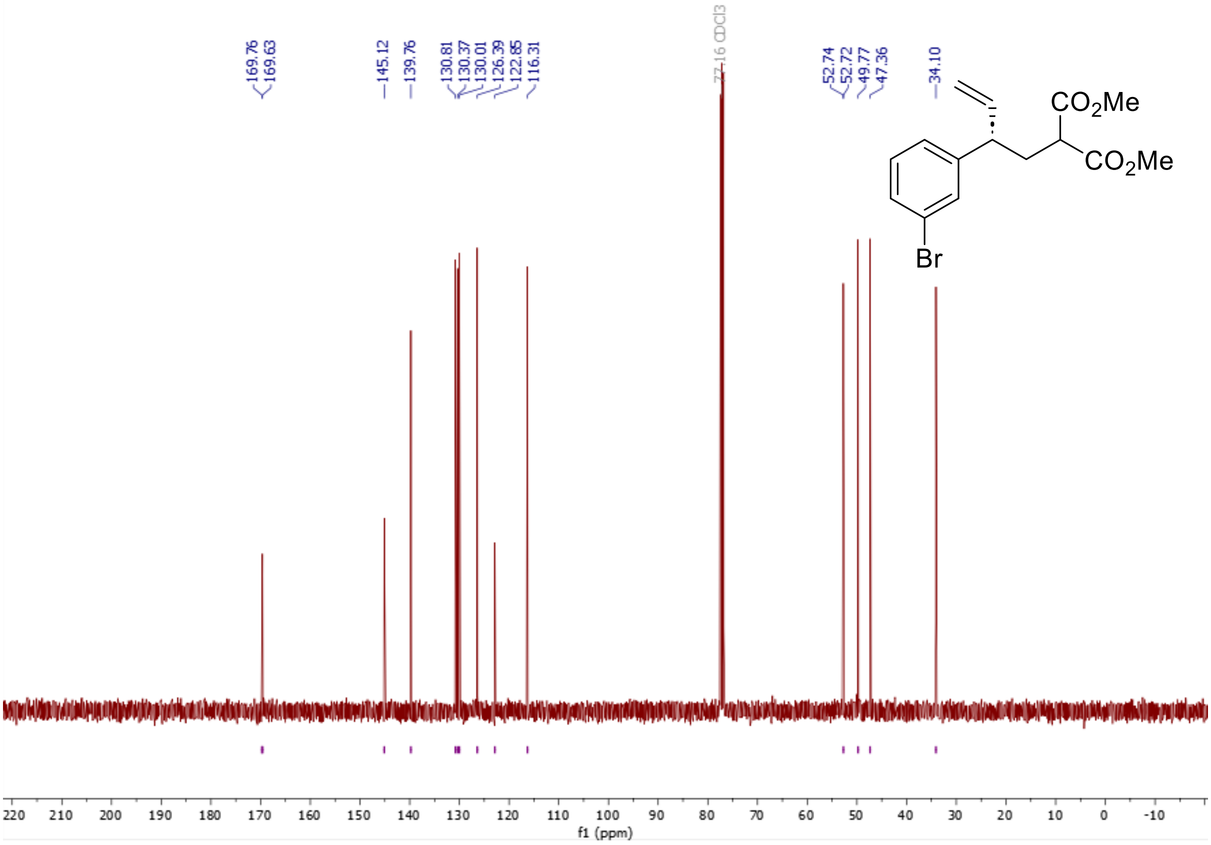

(top) ^{1}H NMR (400 MHz) and (bottom) ^{13}C NMR (101 MHz) spectra of **3i**.

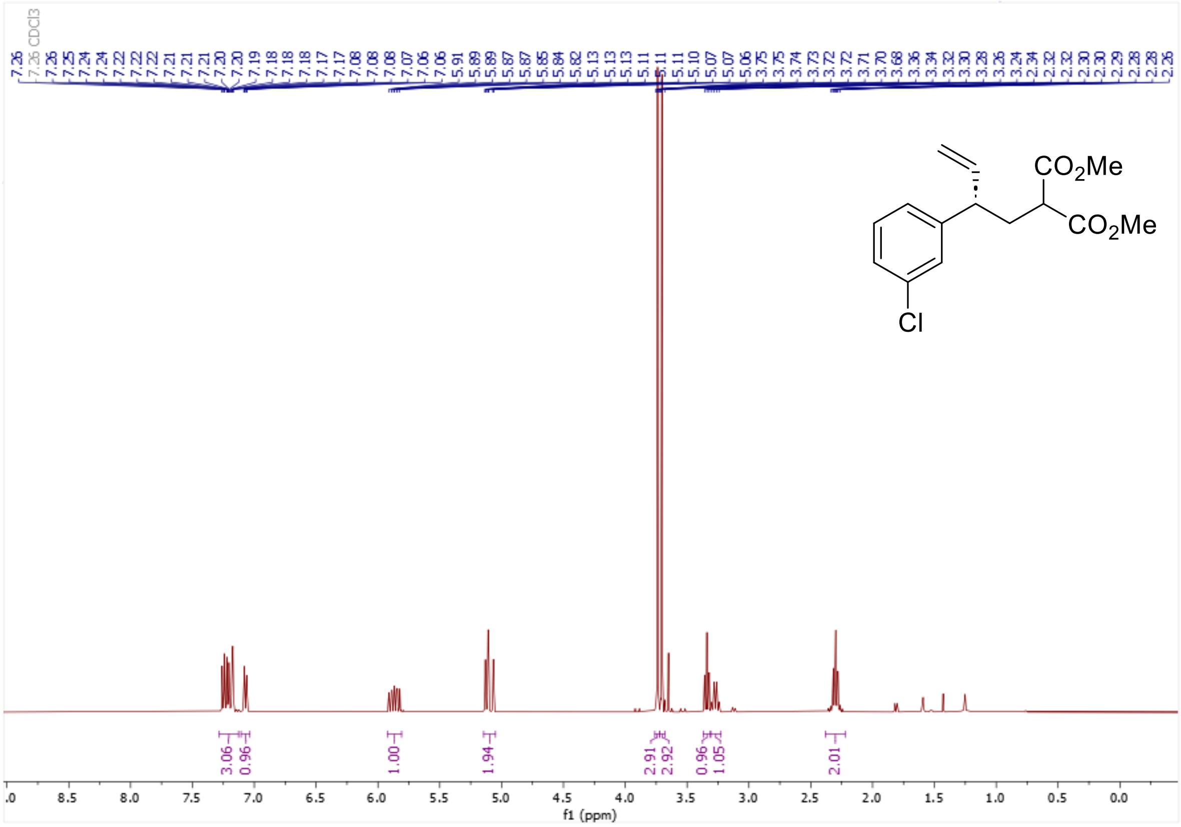

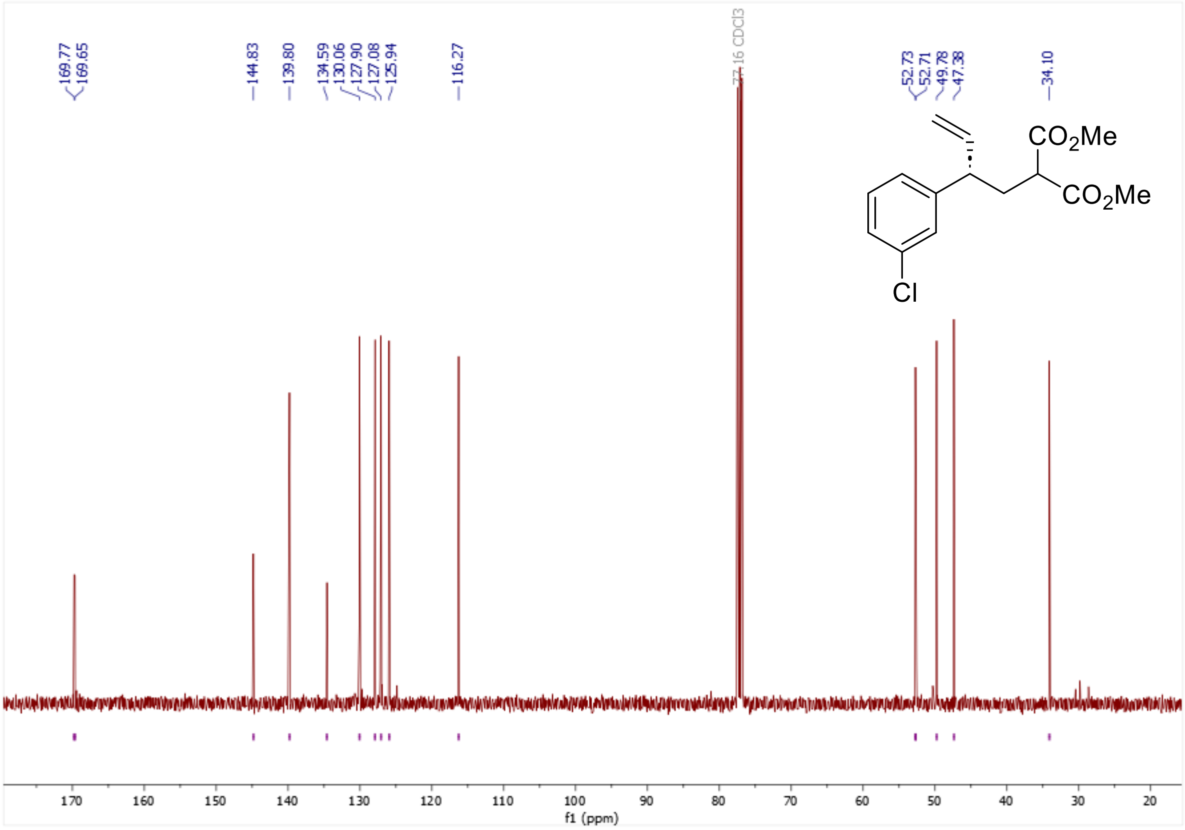

(top) ^{1}H NMR (400 MHz) and (bottom) ^{13}C NMR (101 MHz) spectra of **3j**

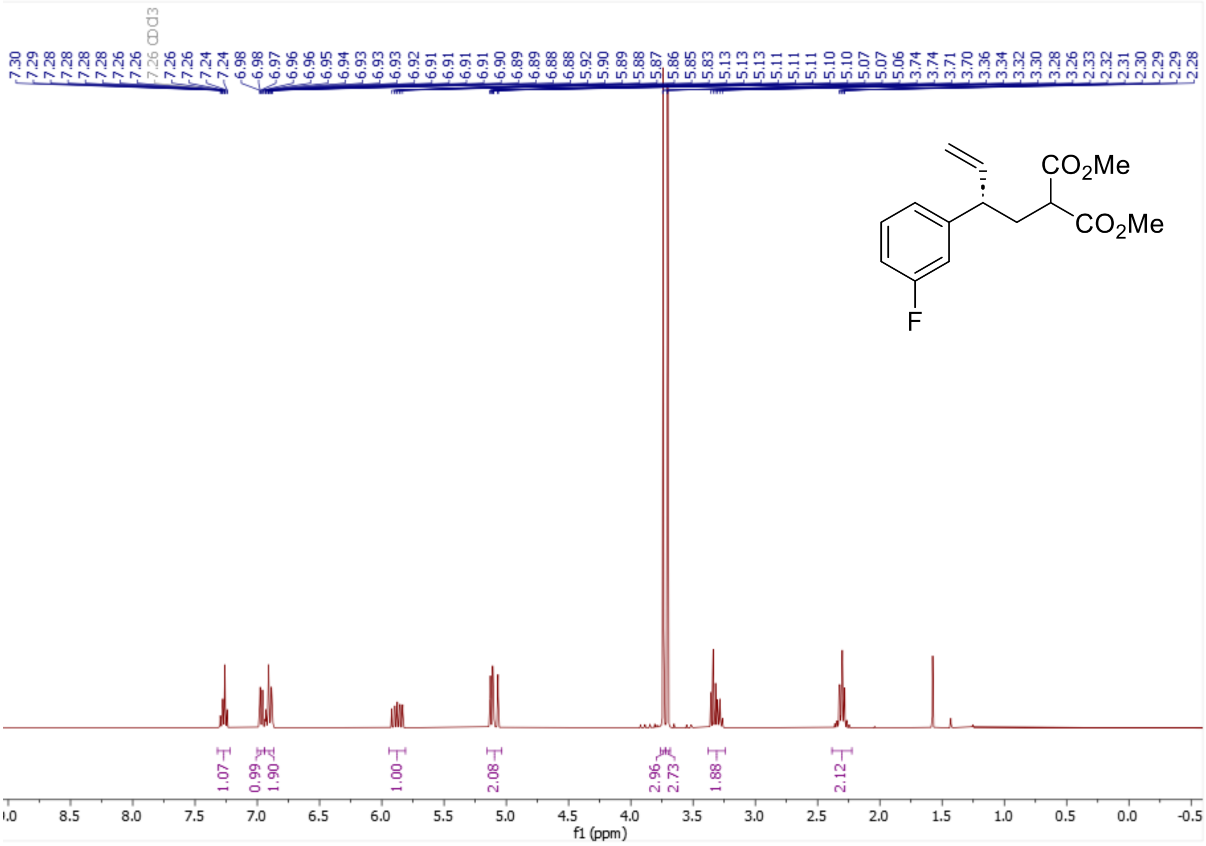

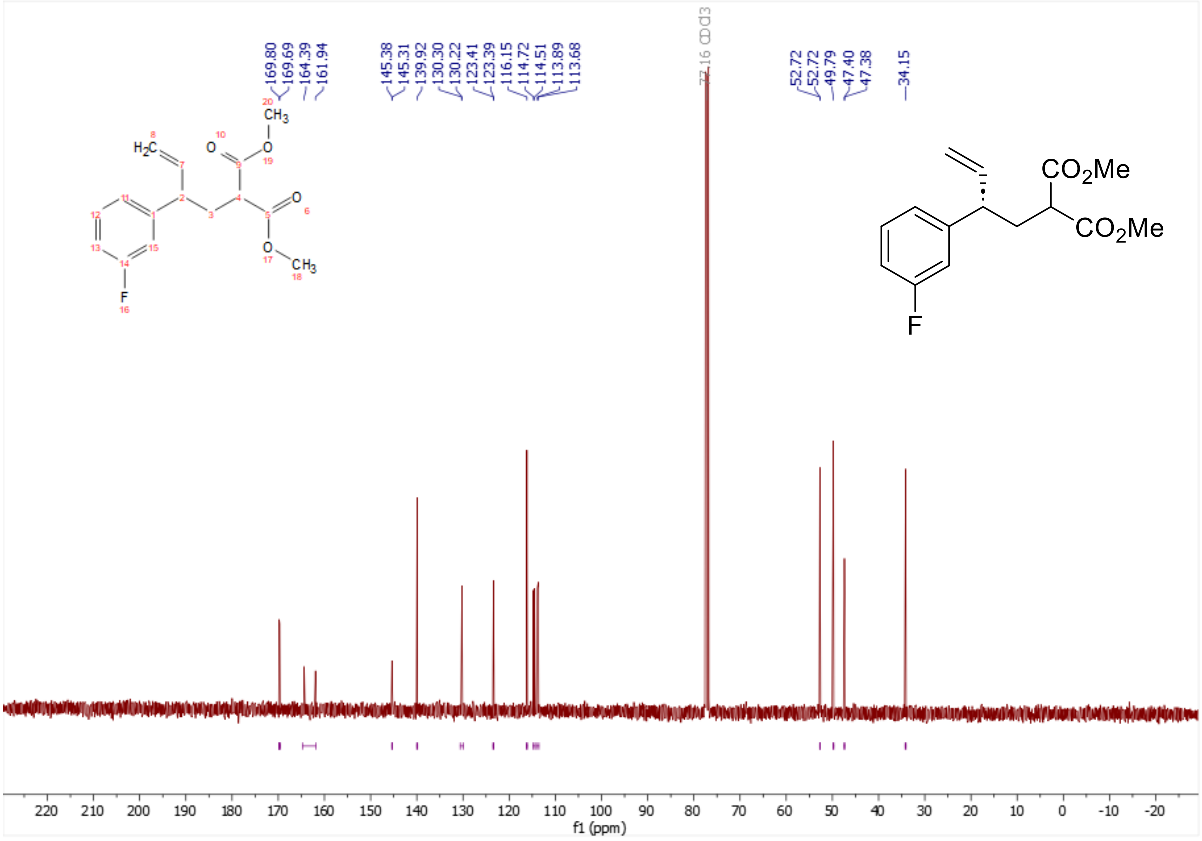

(top) ^{1}H NMR (400 MHz) and (bottom) ^{13}C NMR (101 MHz) spectra of **3k**.

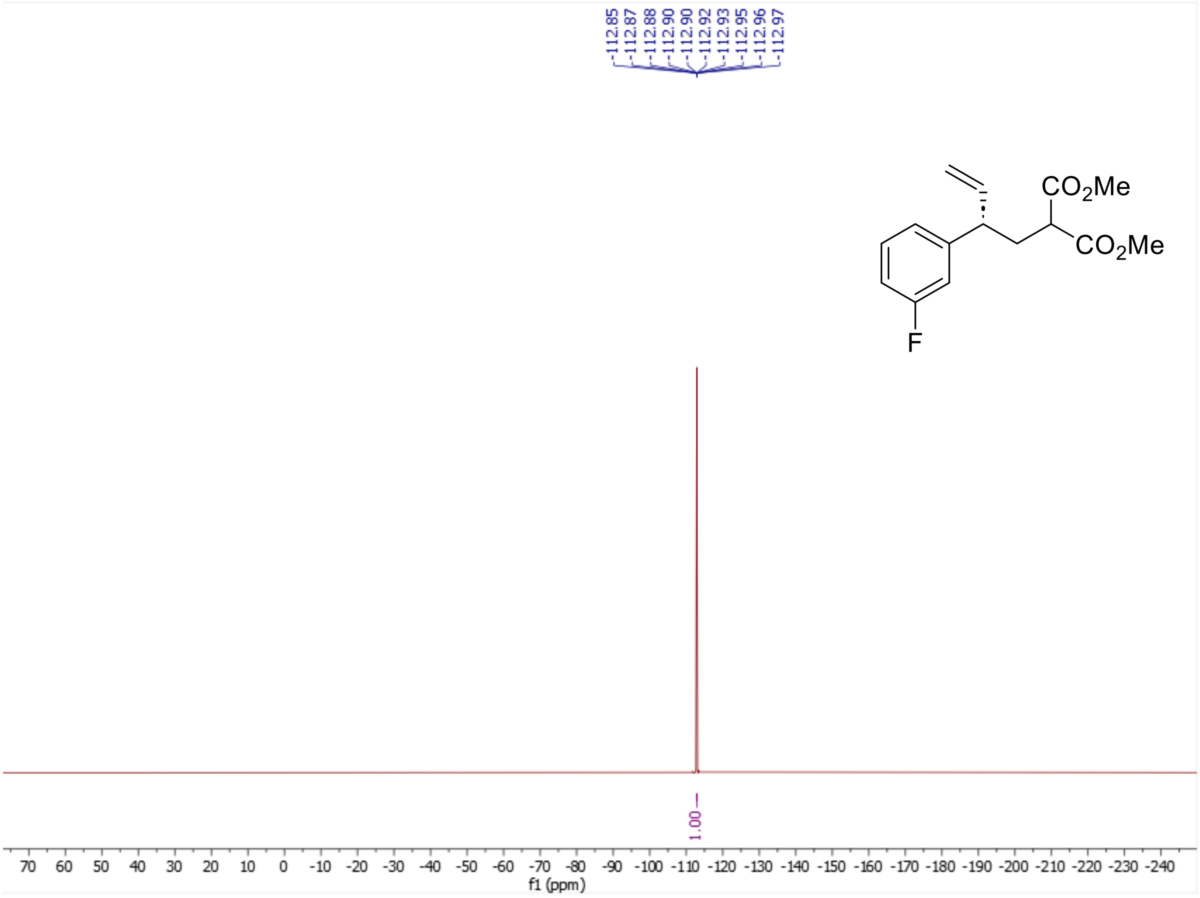

^{19}F NMR (376 MHz) spectra of **3k**.

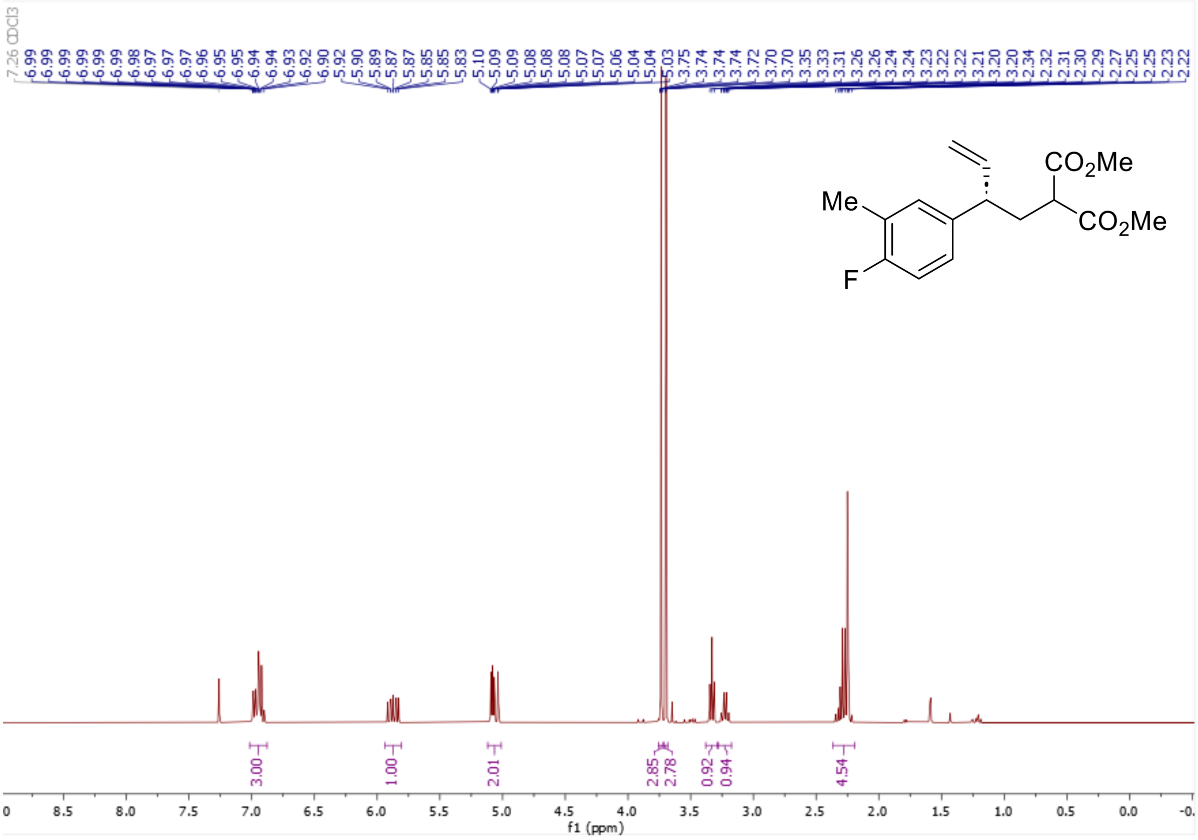

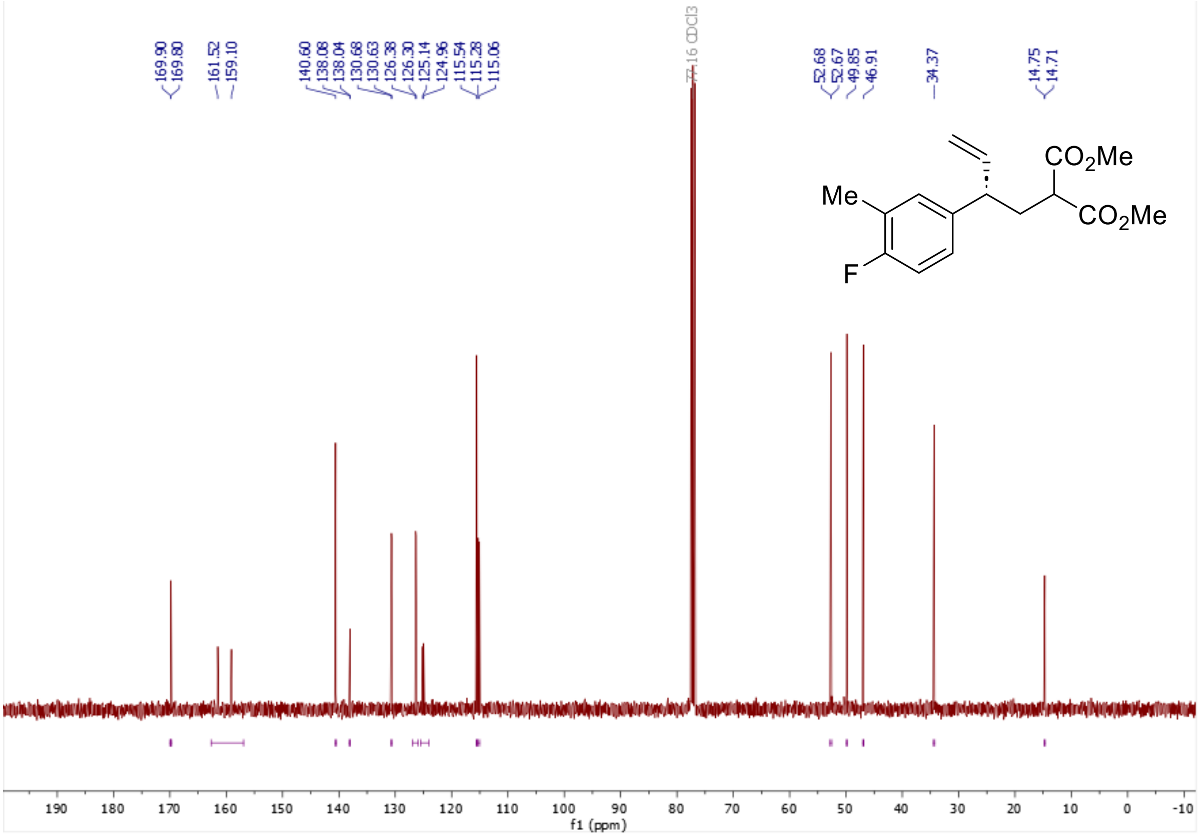

(top) ^{1}H NMR (400 MHz) and (bottom) ^{13}C NMR (101 MHz) spectra of **3l**.

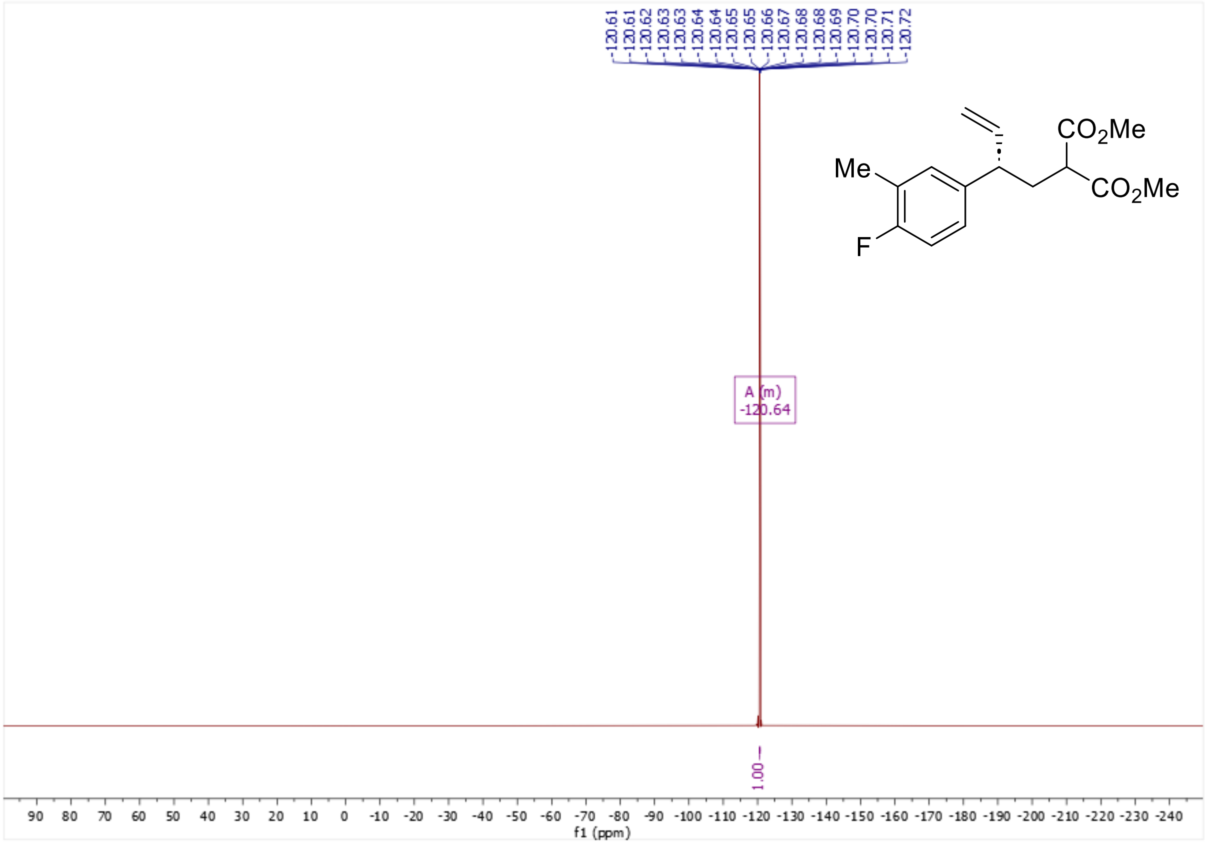

^{19}F NMR (376 MHz) spectra of **3l**.

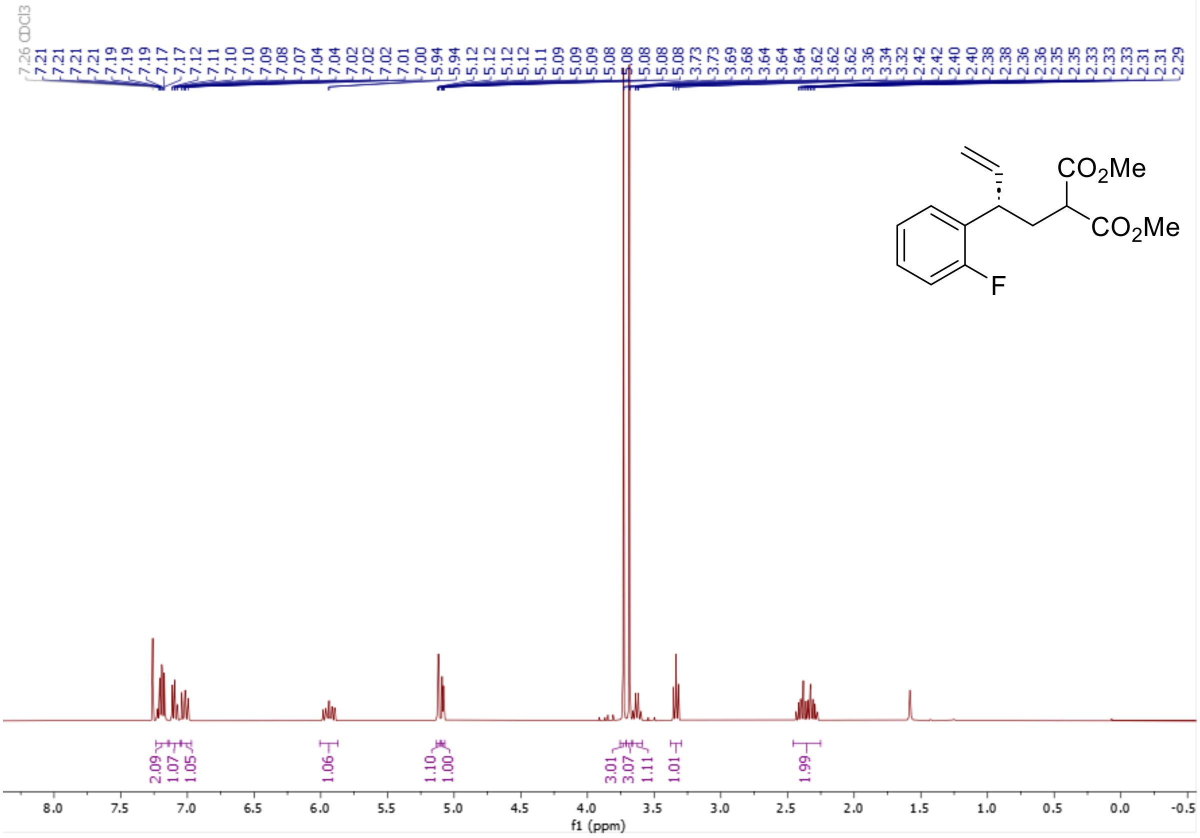

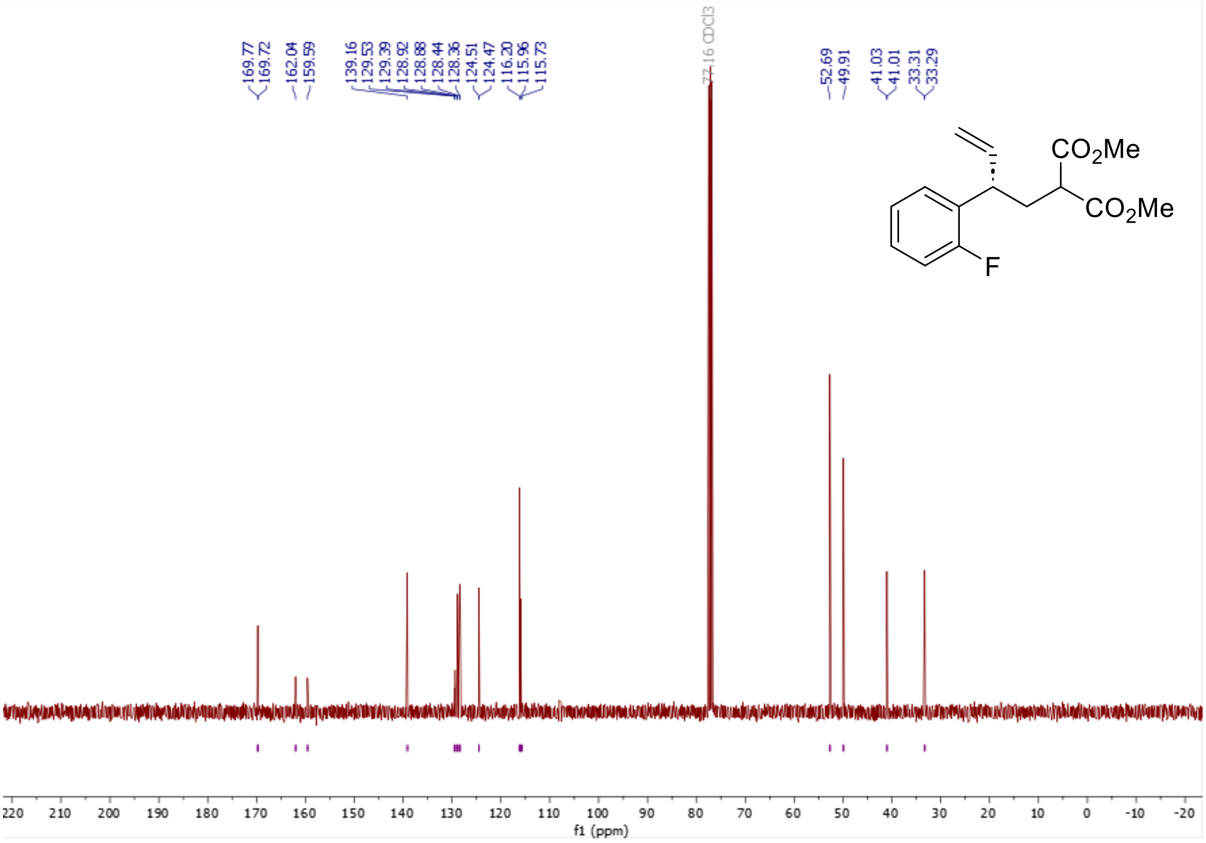

(top) ^{1}H NMR (400 MHz) and (bottom) ^{13}C NMR (101 MHz) spectra of **3m**.

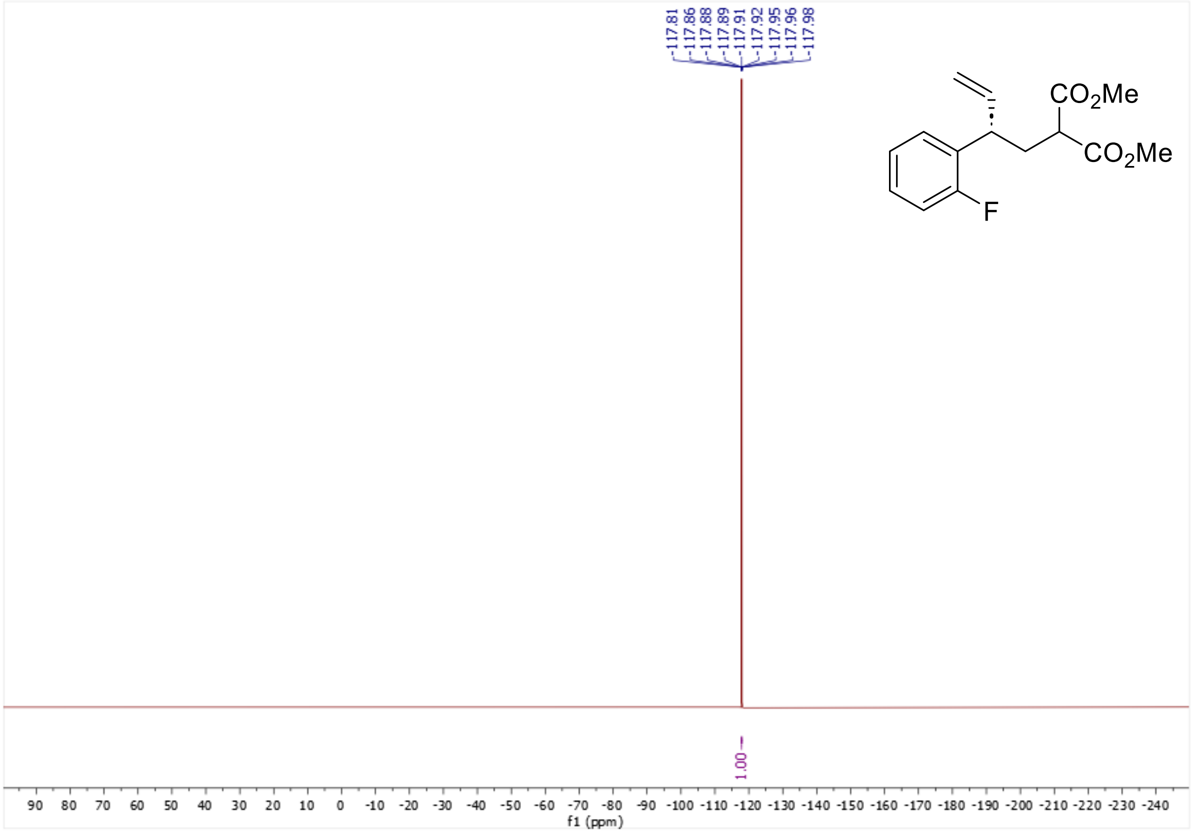

^{19}F NMR (376 MHz) spectra of **3m**.

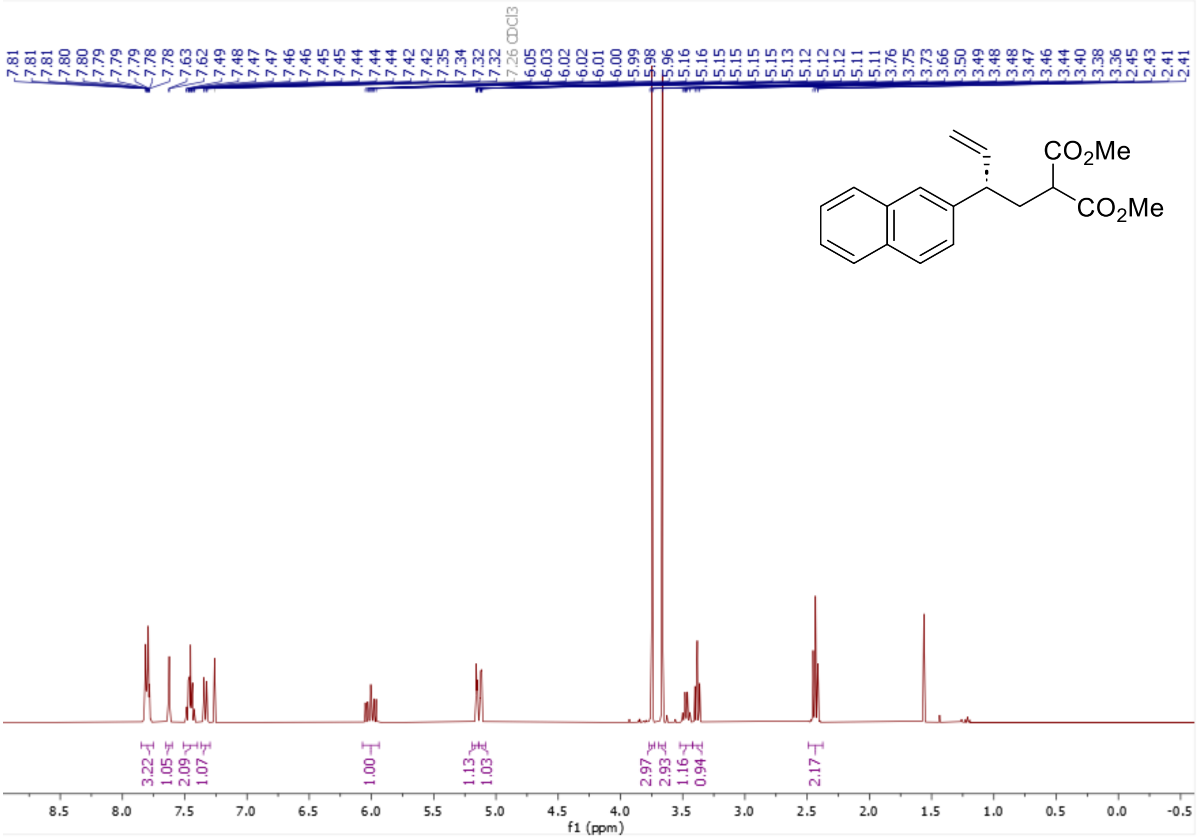

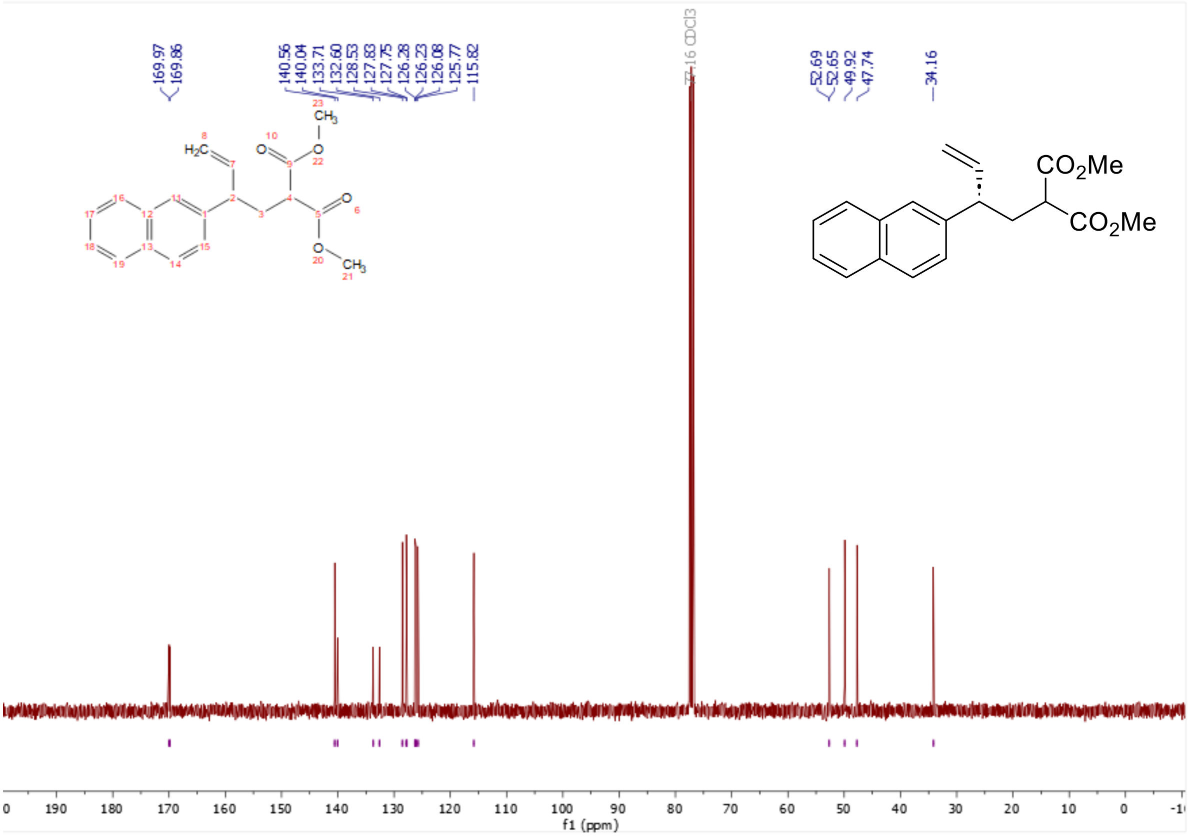

(top) ^{1}H NMR (400 MHz) and (bottom) ^{13}C NMR (101 MHz) spectra of **3n**.

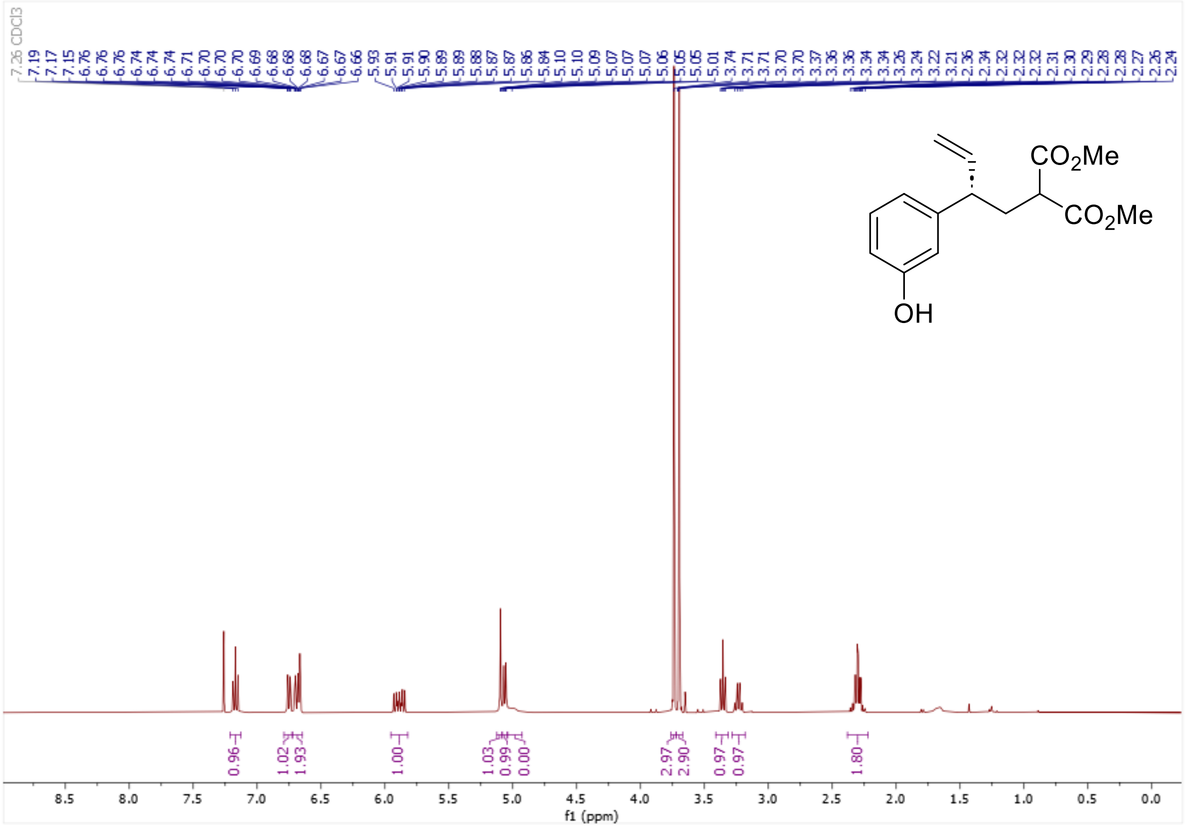

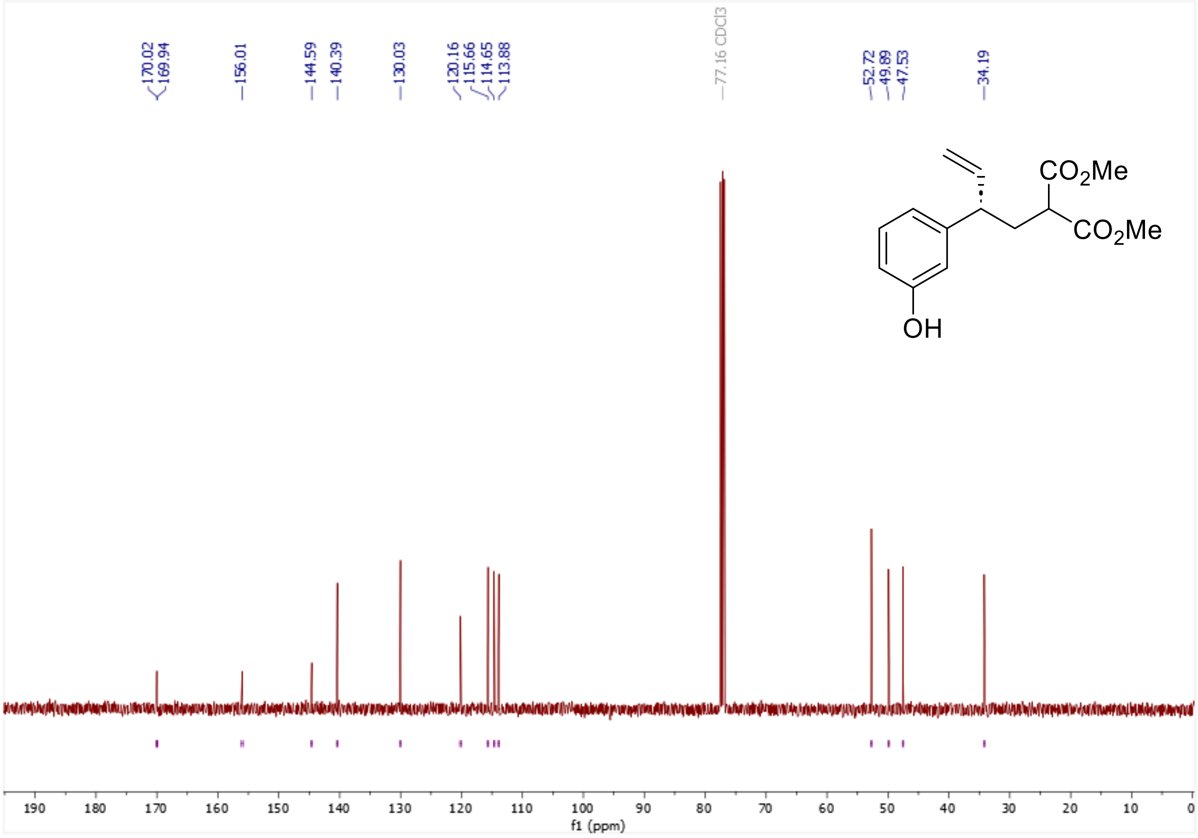

(top) ^{1}H NMR (400 MHz) and (bottom) ^{13}C NMR (101 MHz) spectra of **3o**.

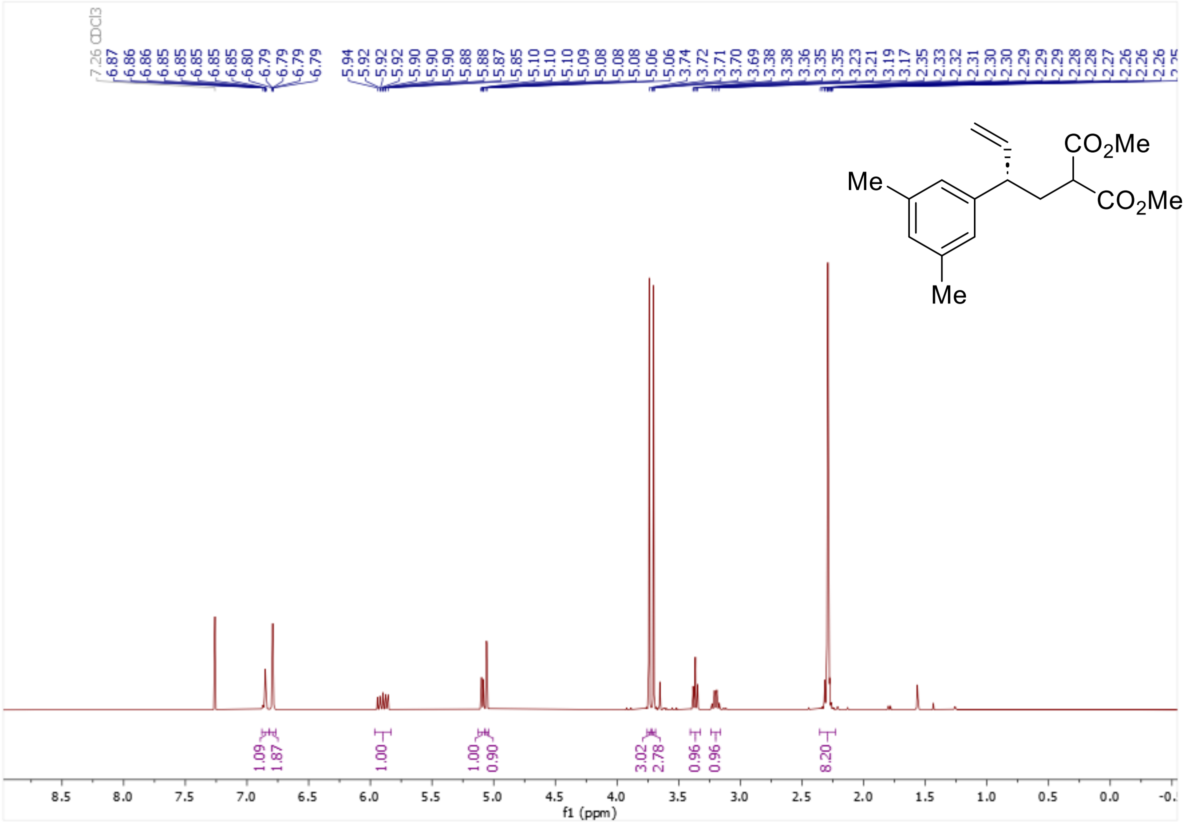

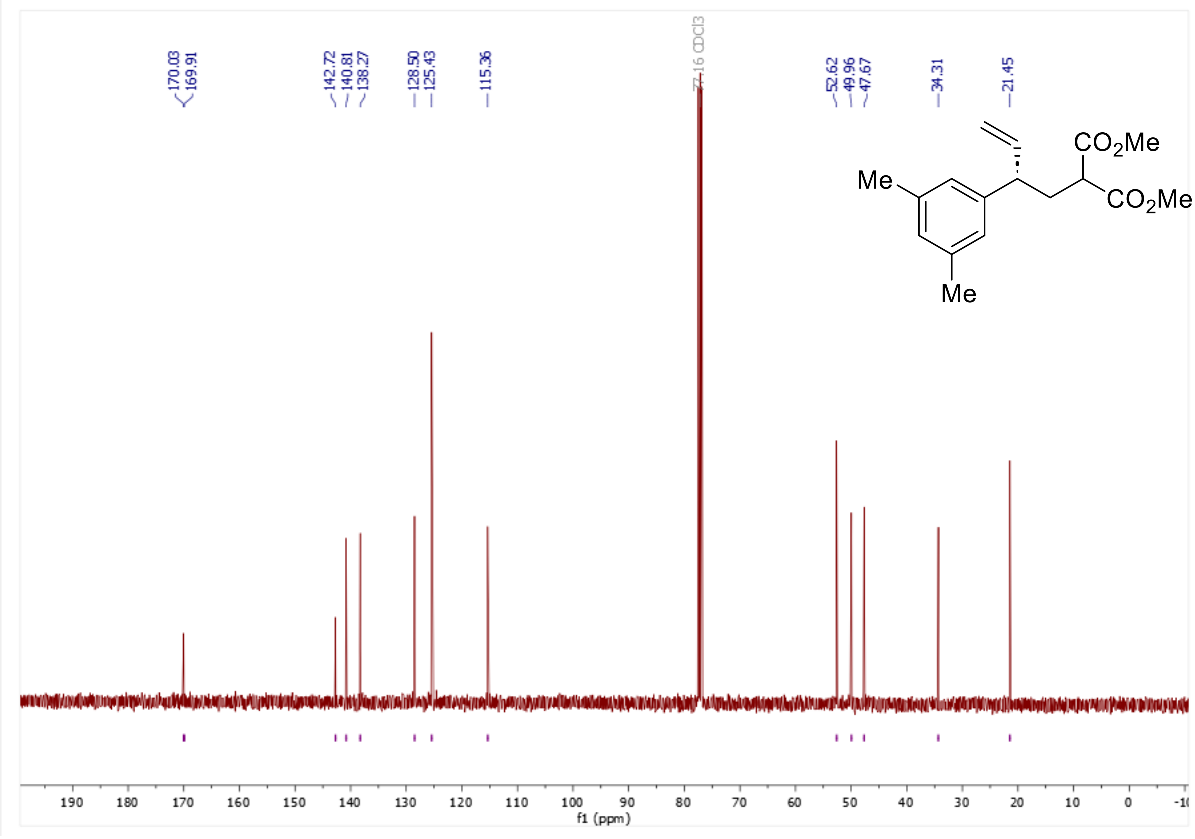

(top) ^{1}H NMR (400 MHz) and (bottom) ^{13}C NMR (101 MHz) spectra of **3p**.

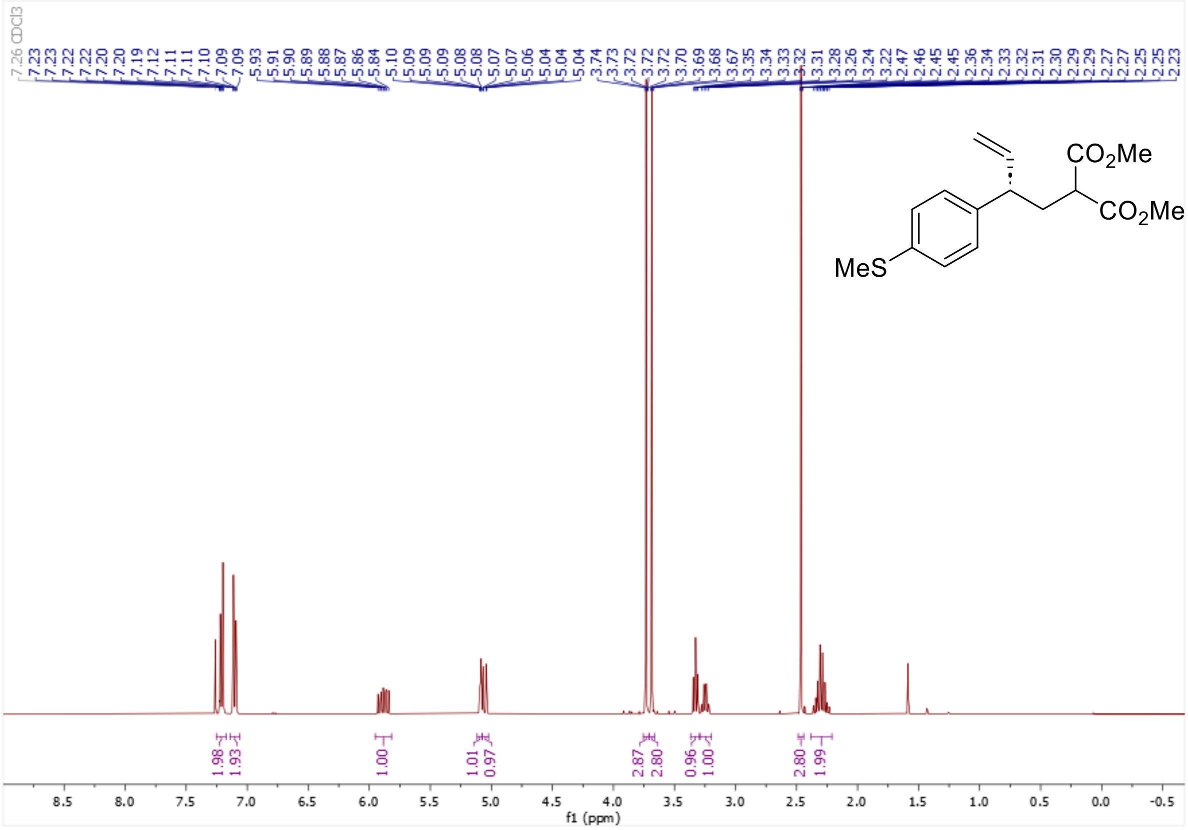

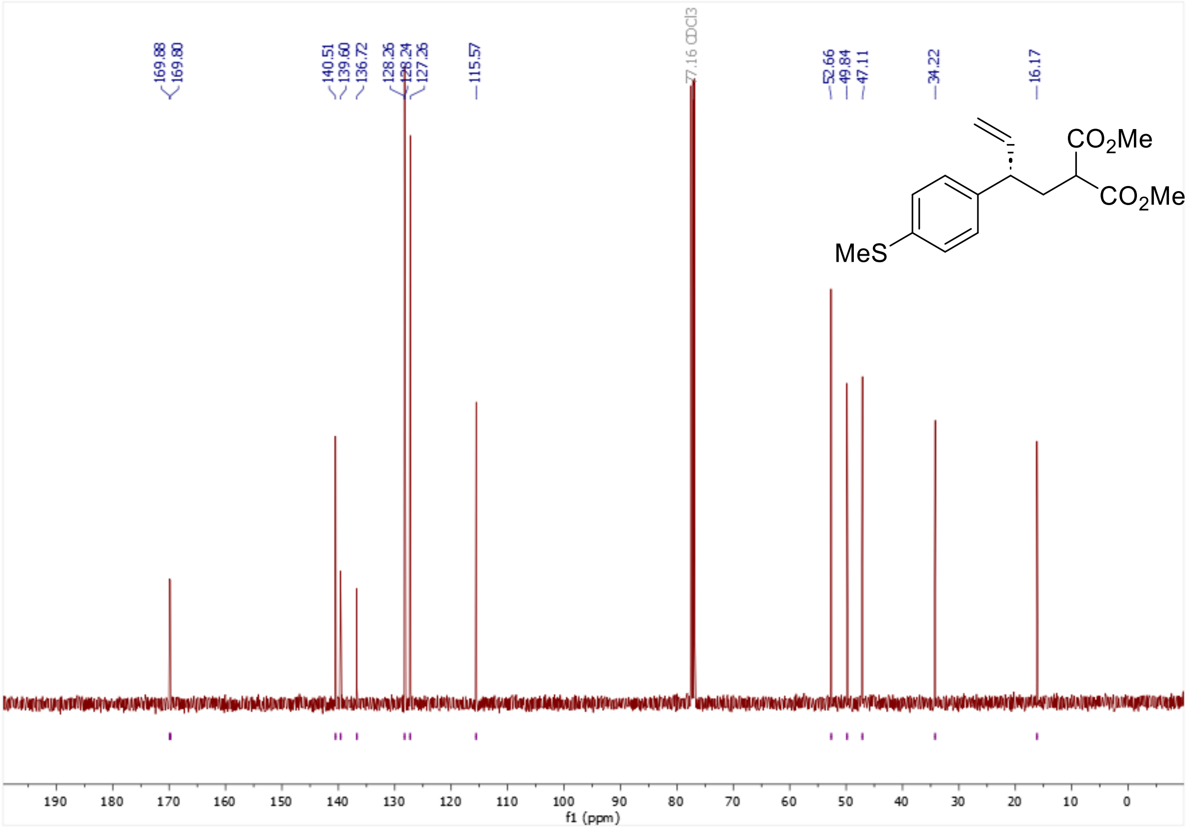

(top) ^{1}H NMR (400 MHz) and (bottom) ^{13}C NMR (101 MHz) spectra of **3q**.

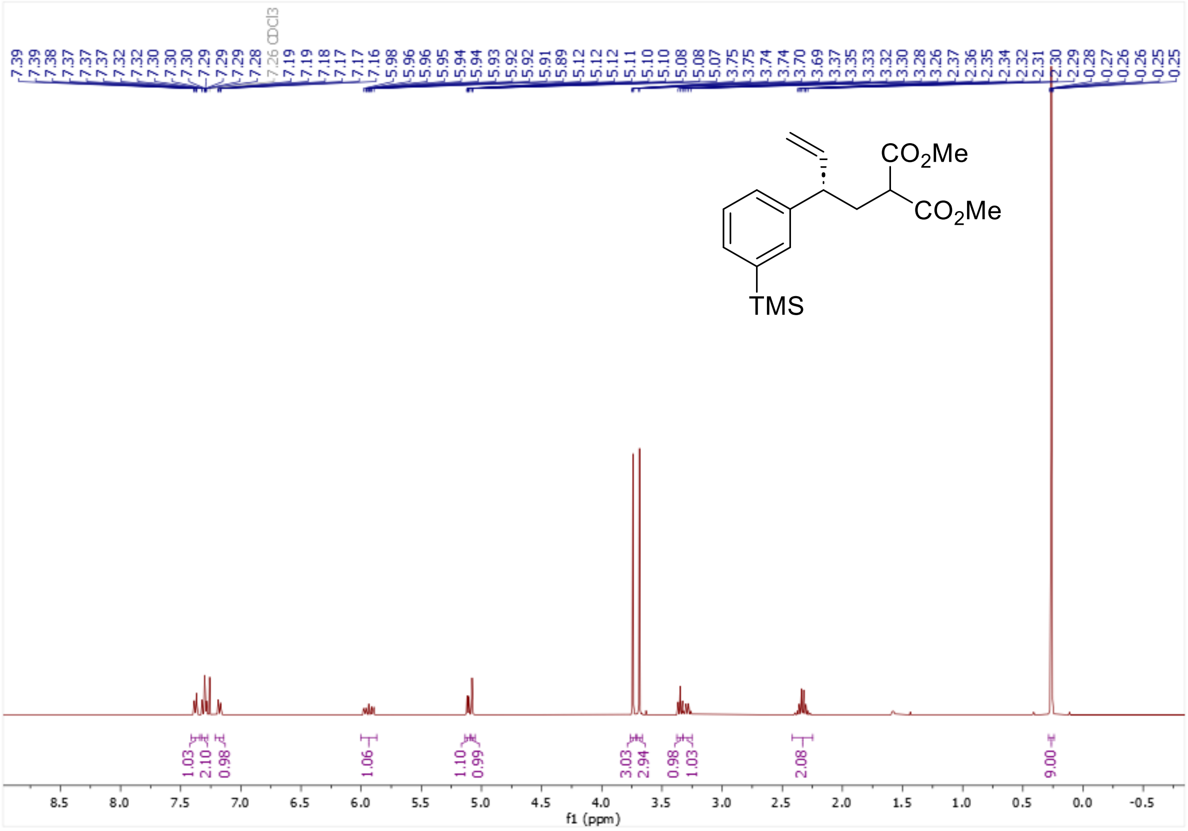

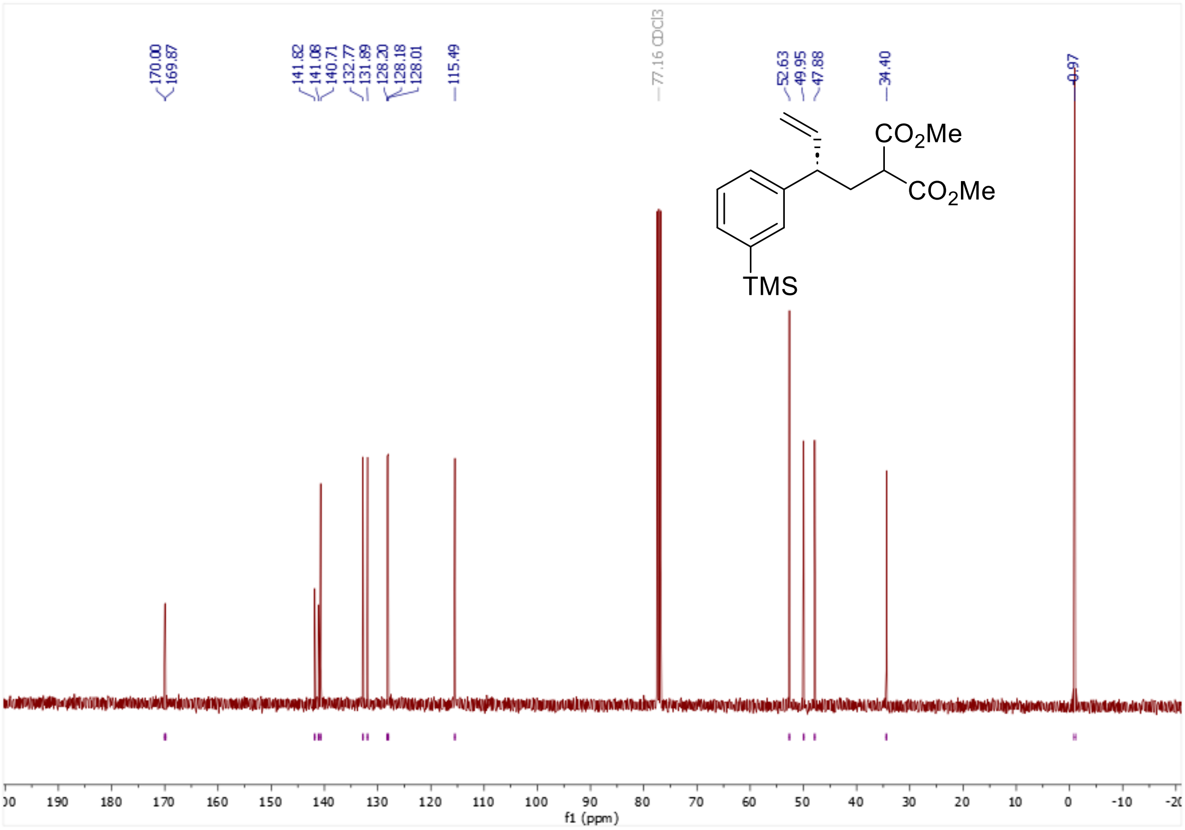

(top) ^{1}H NMR (400 MHz) and (bottom) ^{13}C NMR (101 MHz) spectra of **3r**.

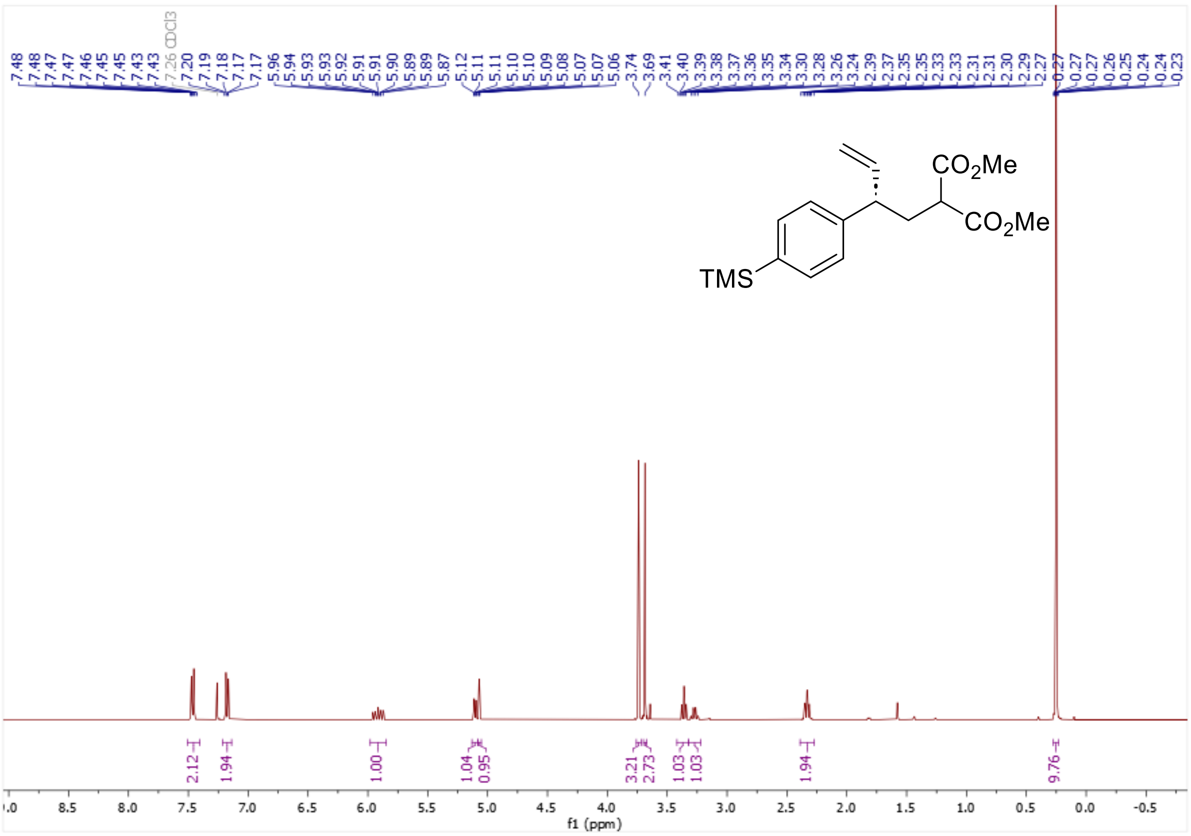

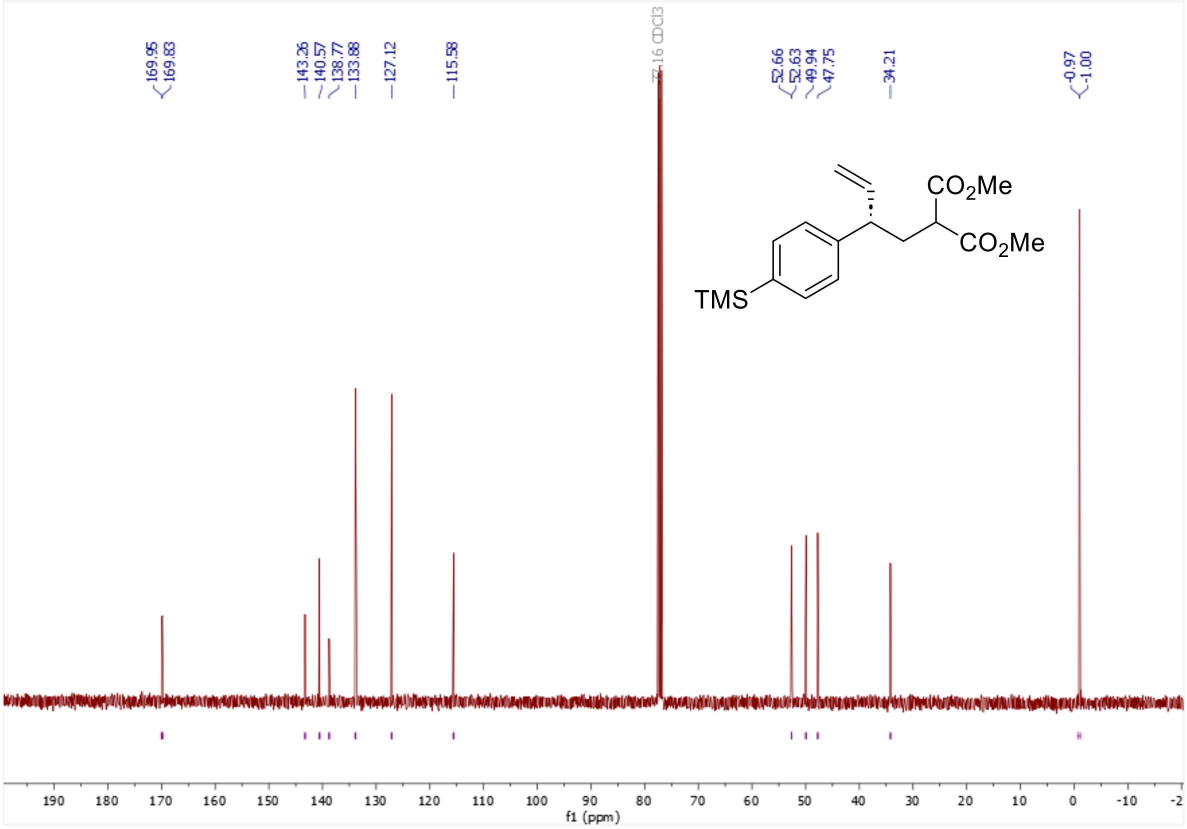

(top) ^{1}H NMR (400 MHz) and (bottom) ^{13}C NMR (101 MHz) spectra of **3s**.

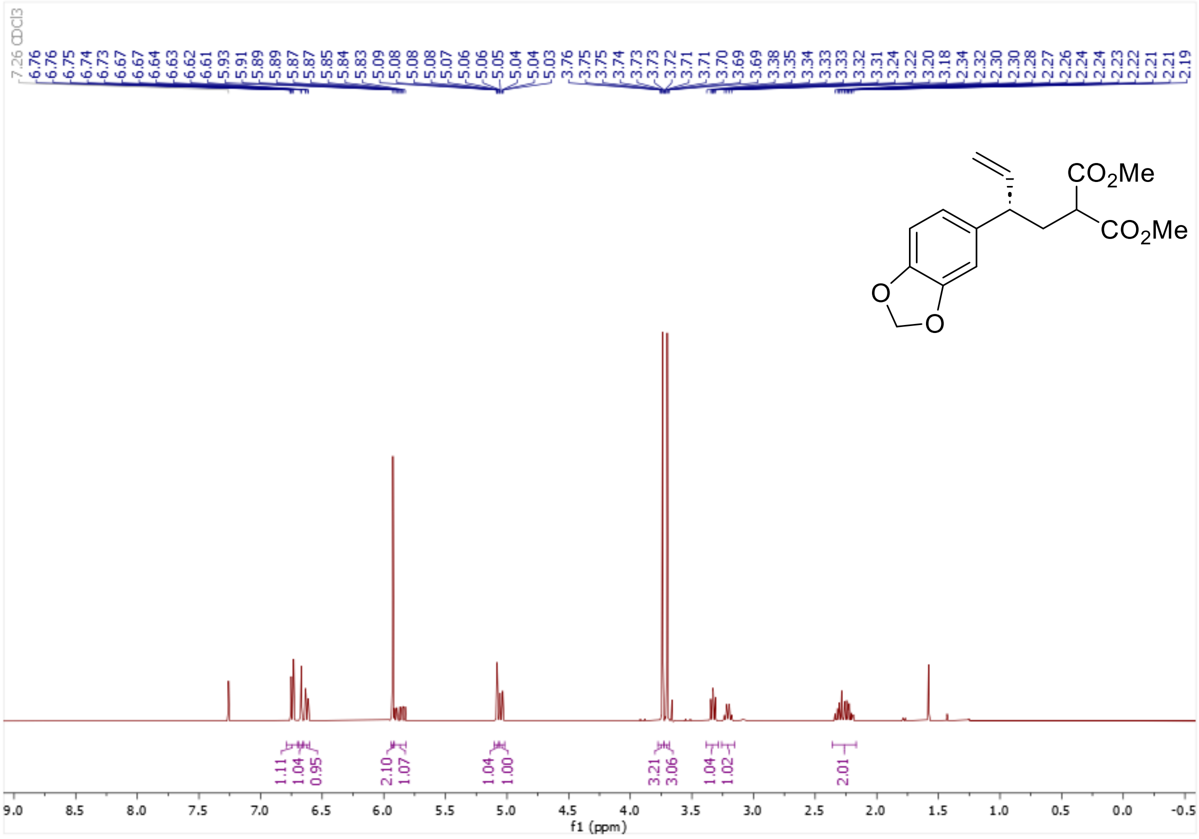

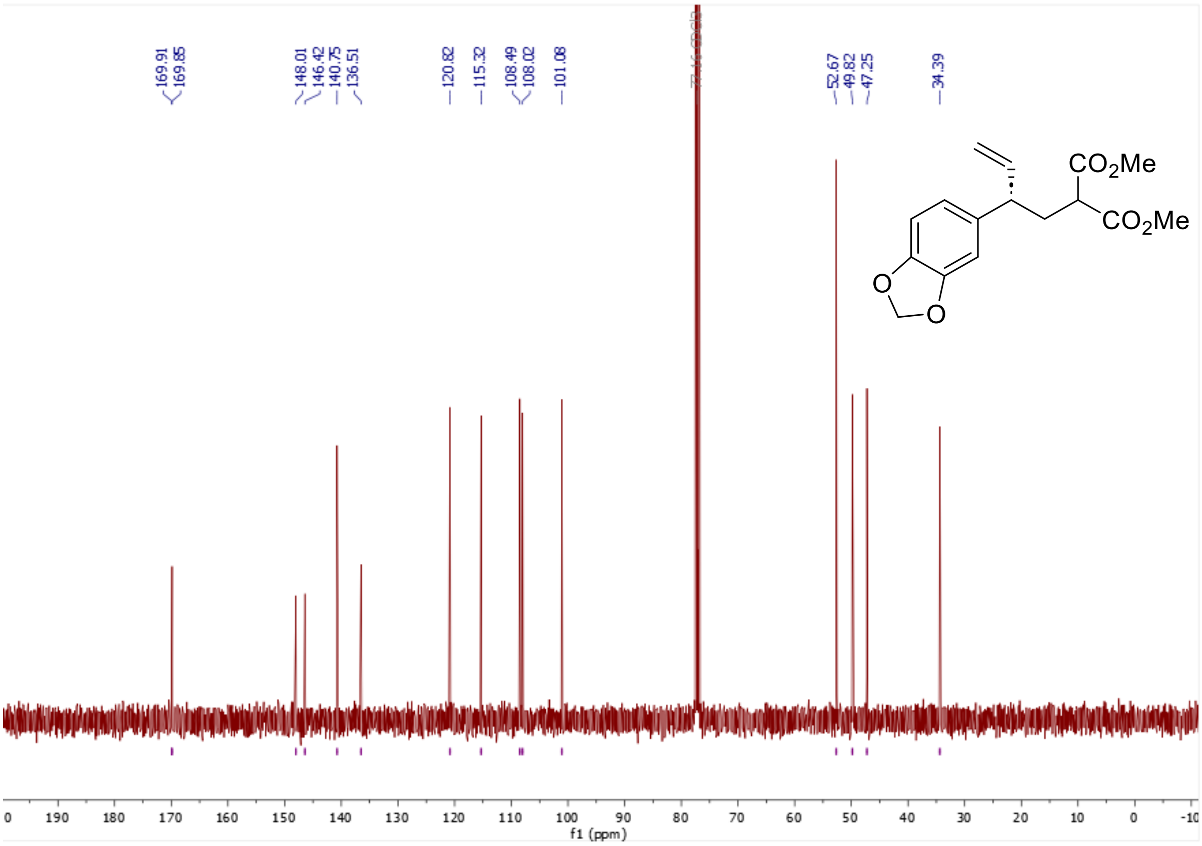

(top) ^{1}H NMR (400 MHz) and (bottom) ^{13}C NMR (101 MHz) spectra of **3t**.

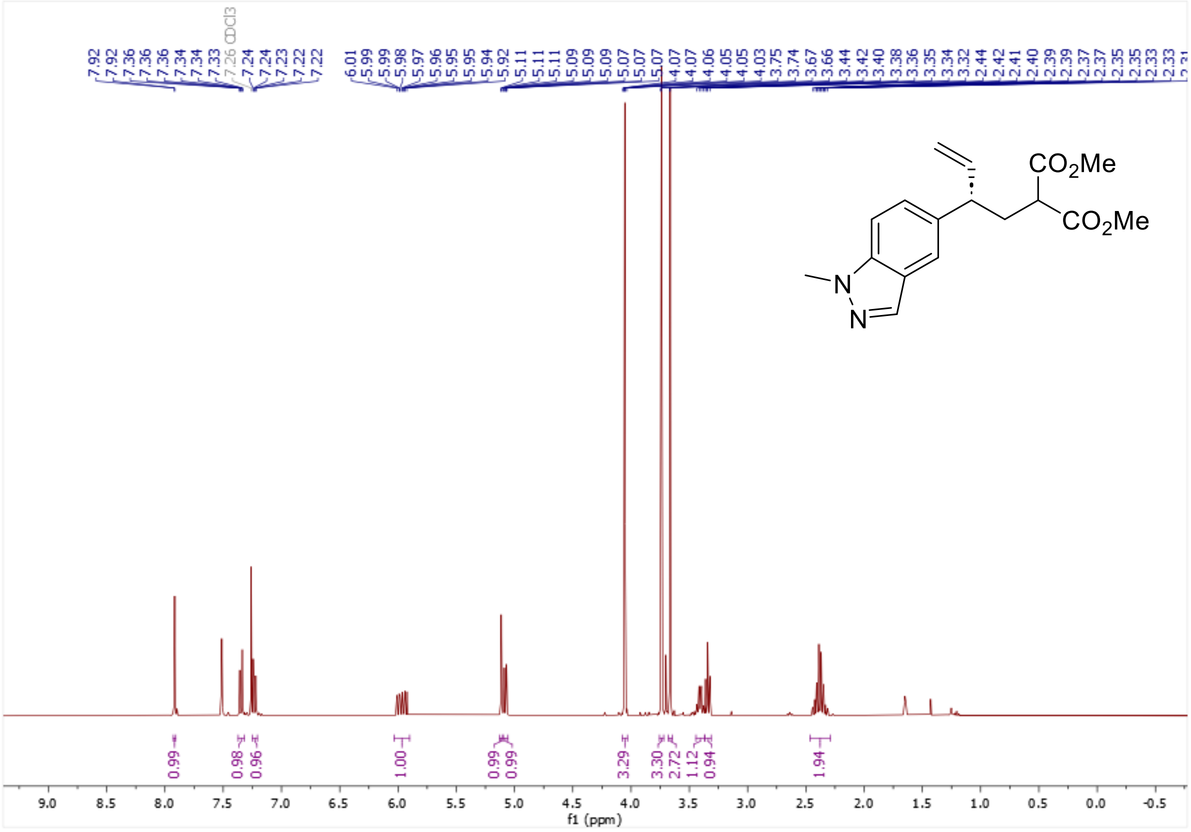

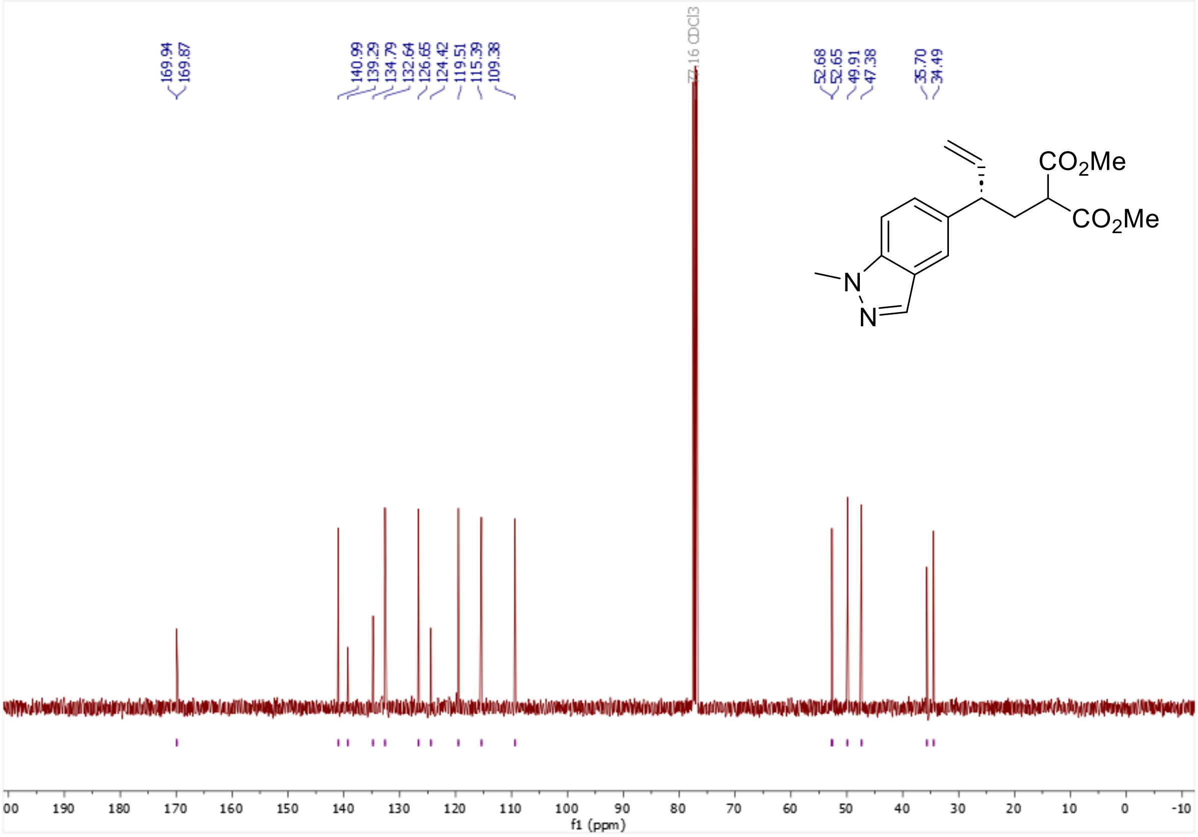

(top) ^{1}H NMR (400 MHz) and (bottom) ^{13}C NMR (101 MHz) spectra of **3u**.

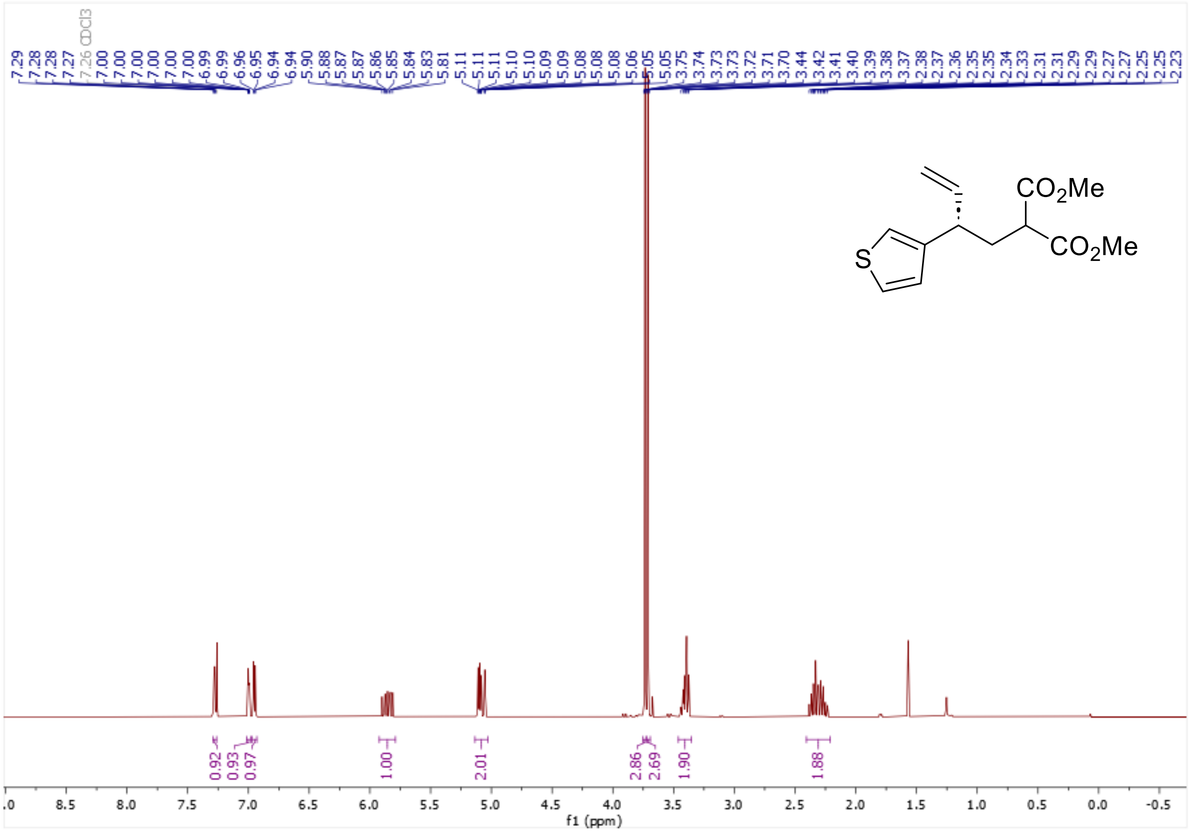

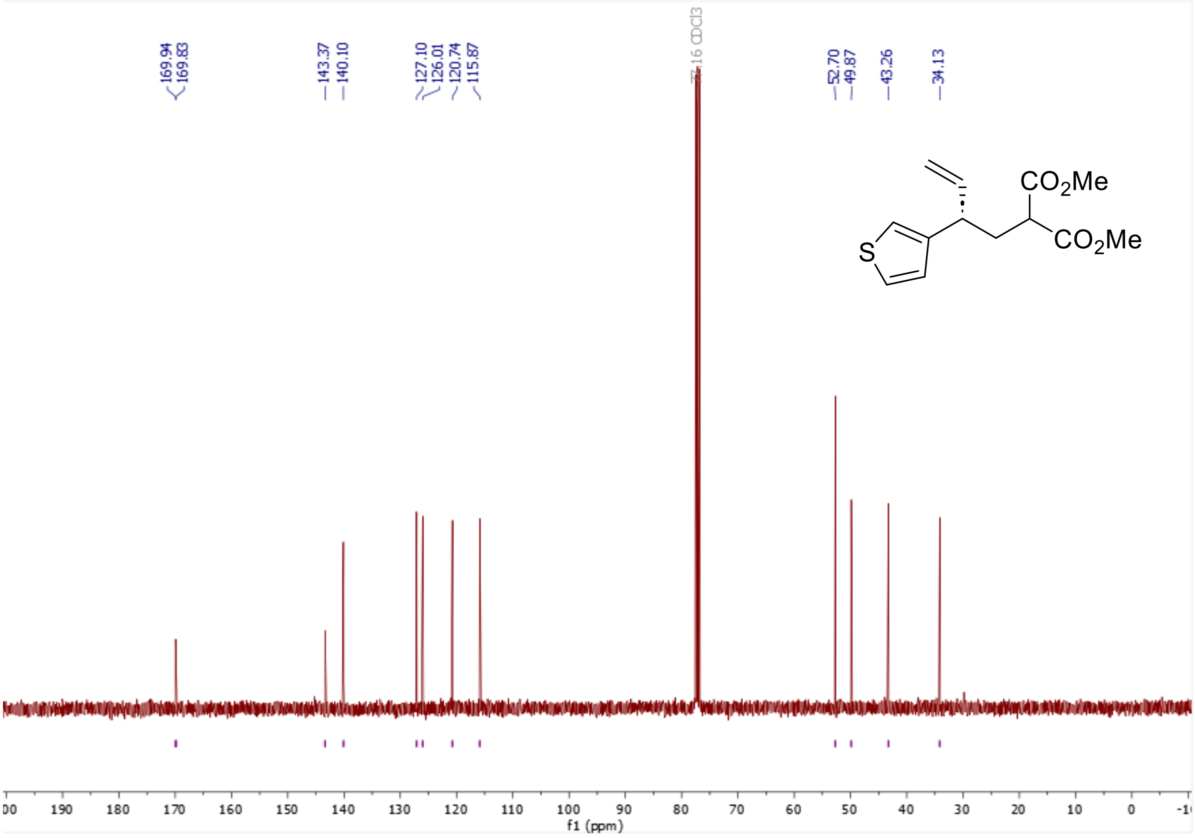

(top) ^{1}H NMR (400 MHz) and (bottom) ^{13}C NMR (101 MHz) spectra of **3v**.

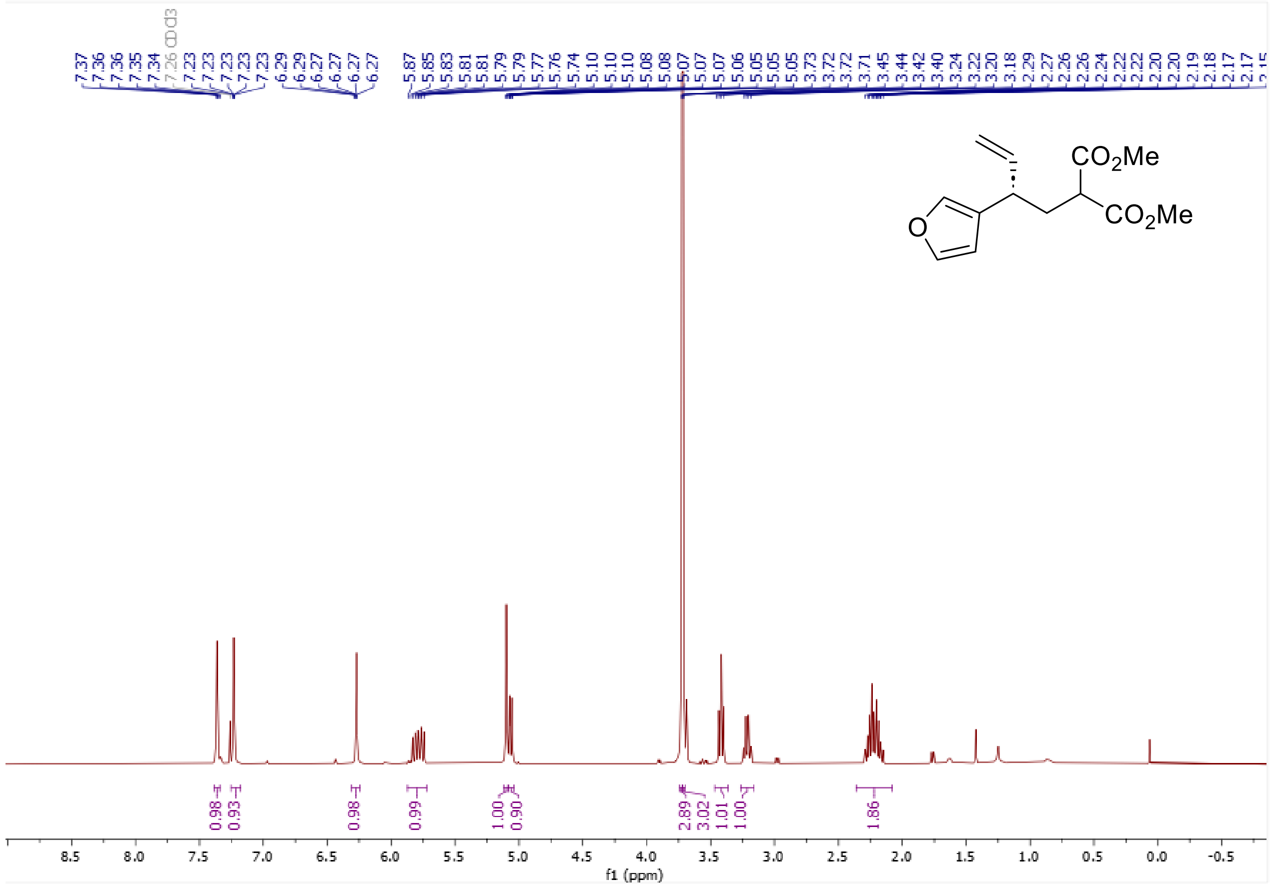

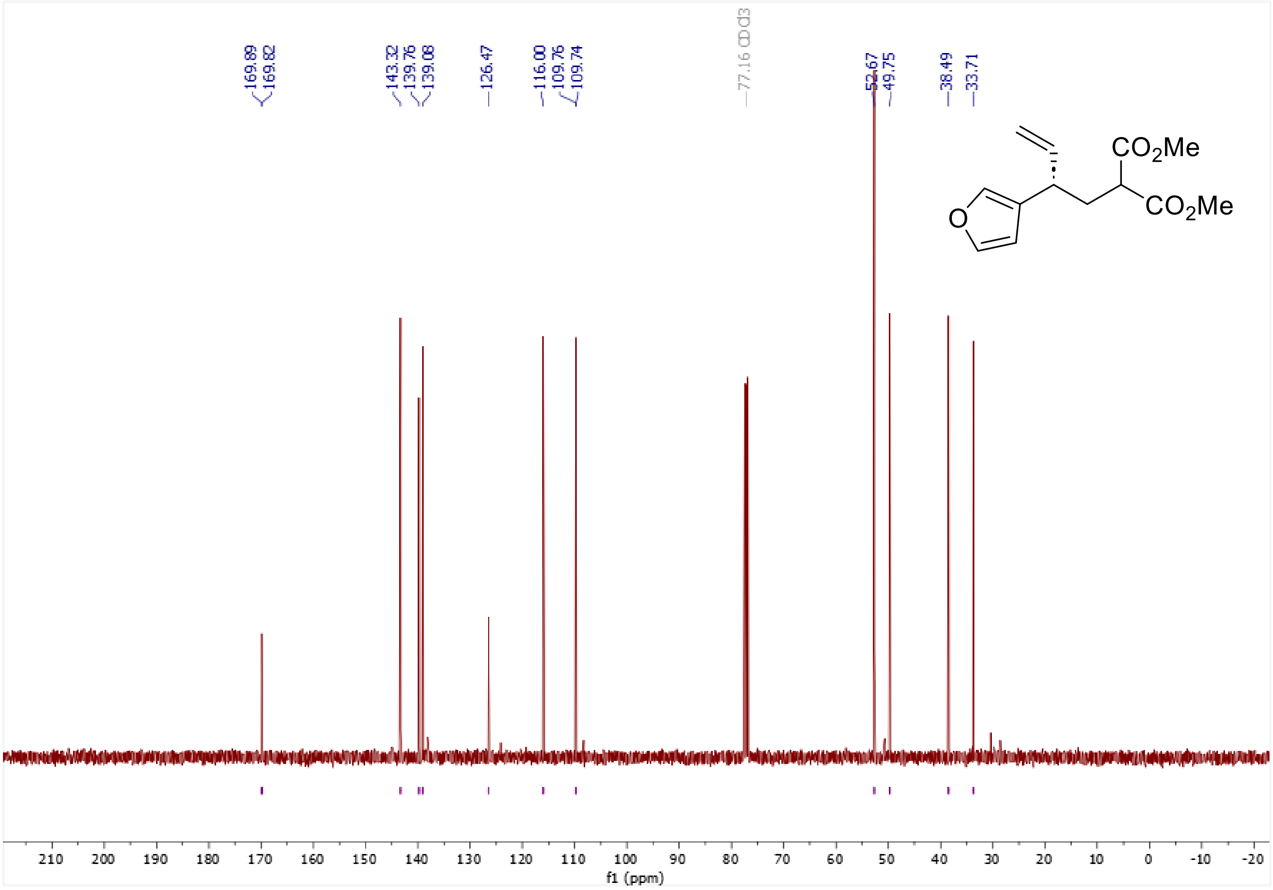

(top) ^{1}H NMR (400 MHz) and (bottom) ^{13}C NMR (101 MHz) spectra of **3w**.

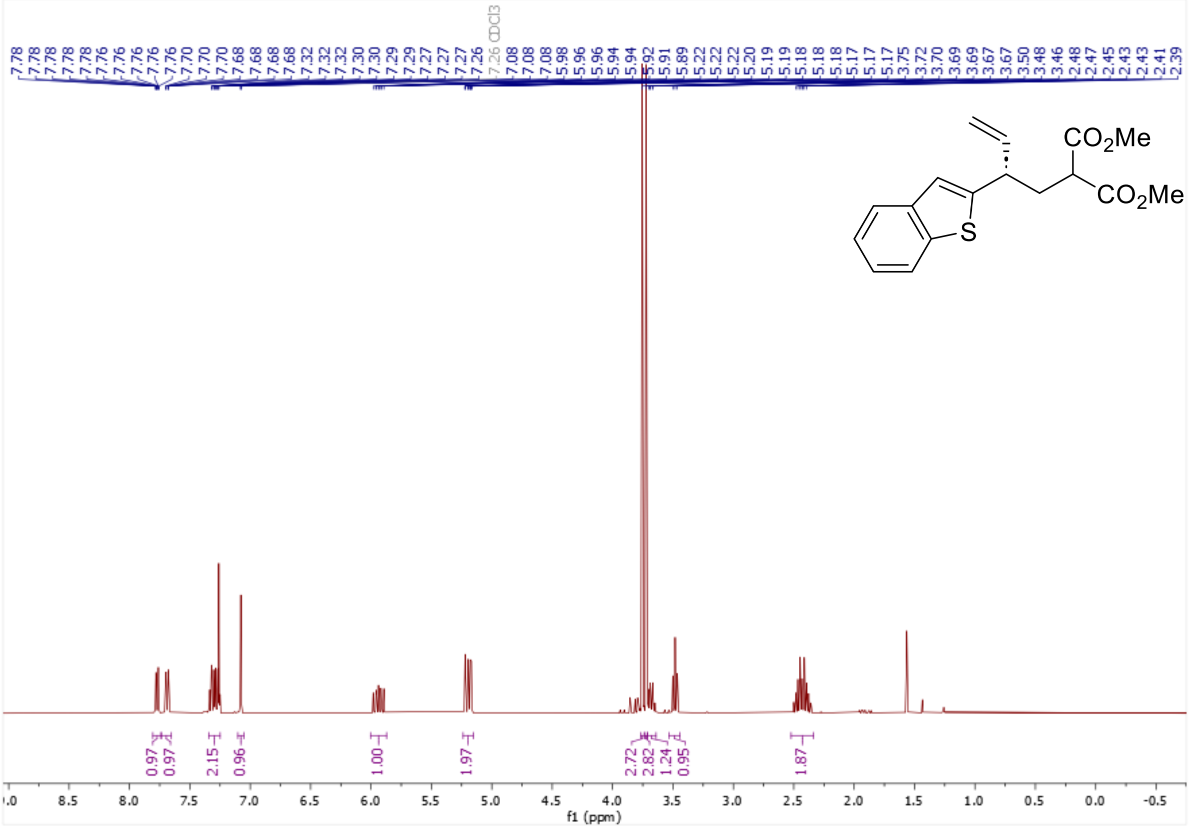

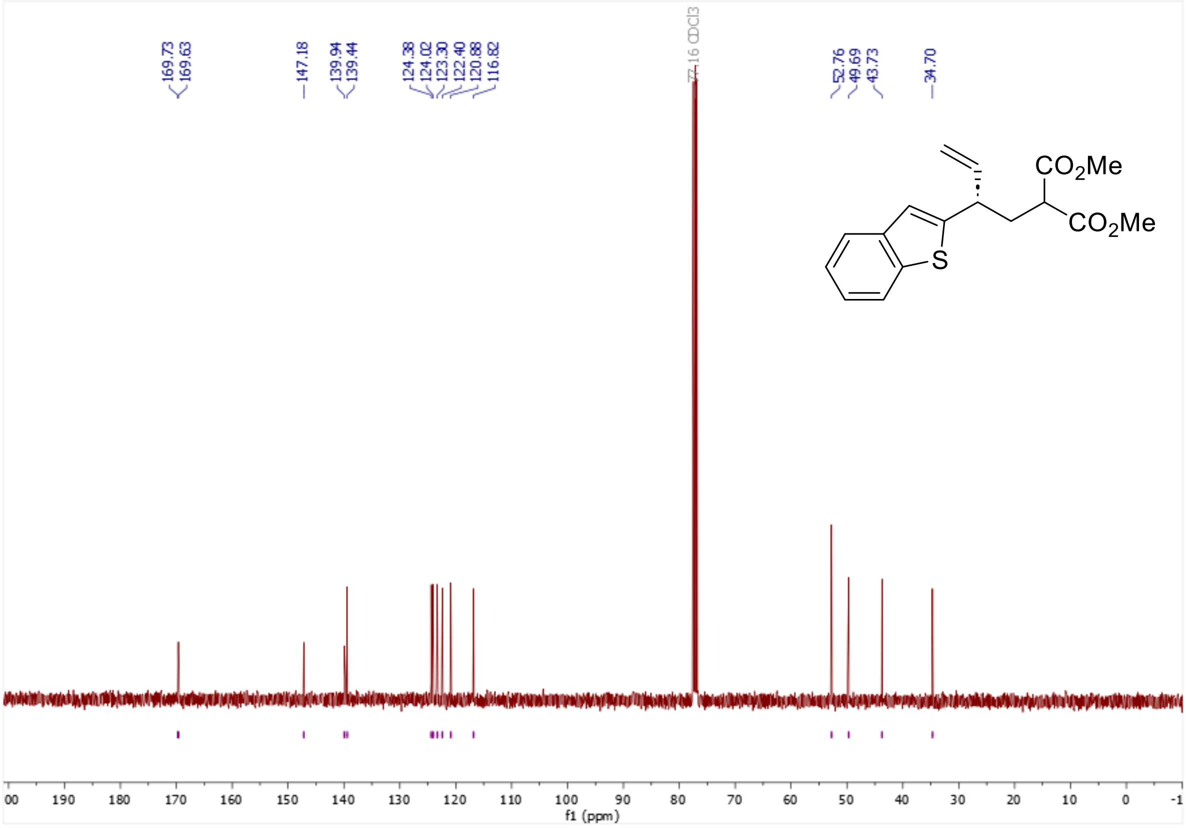

(top) ^{1}H NMR (400 MHz) and (bottom) ^{13}C NMR (101 MHz) spectra of **3x**.

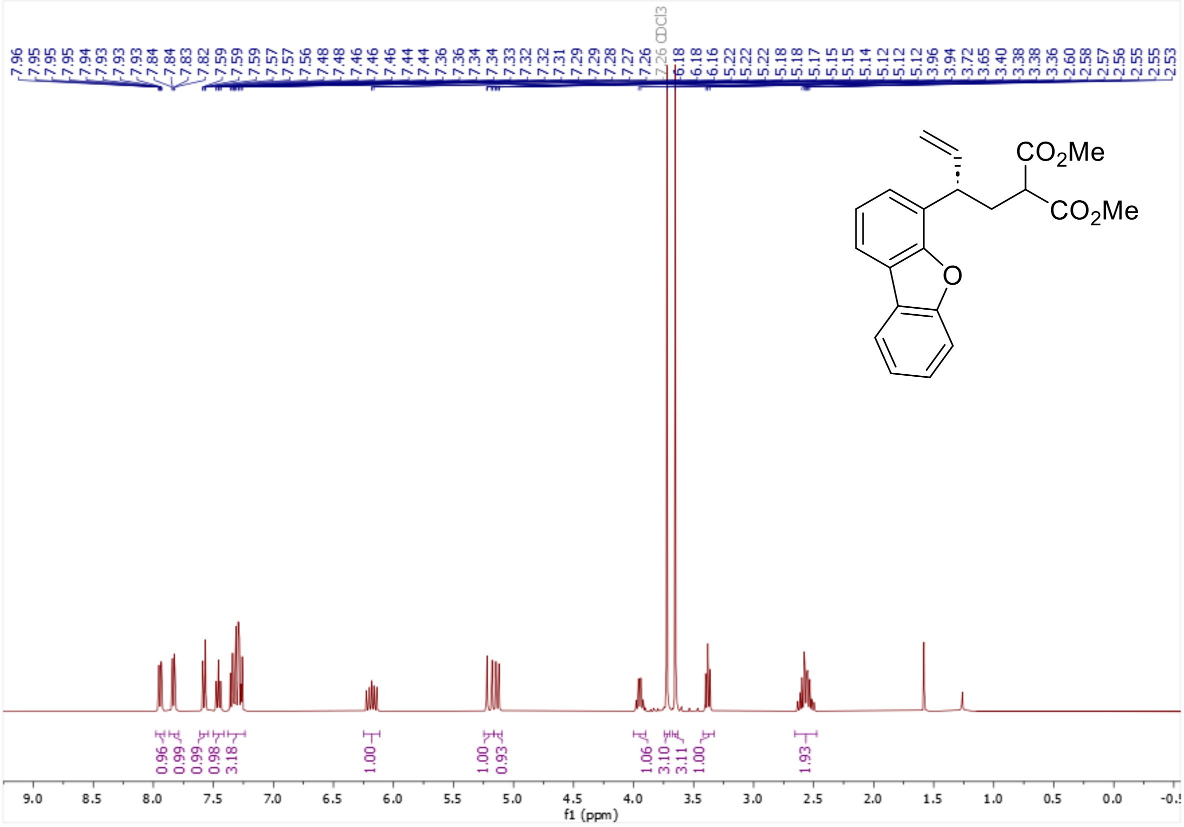

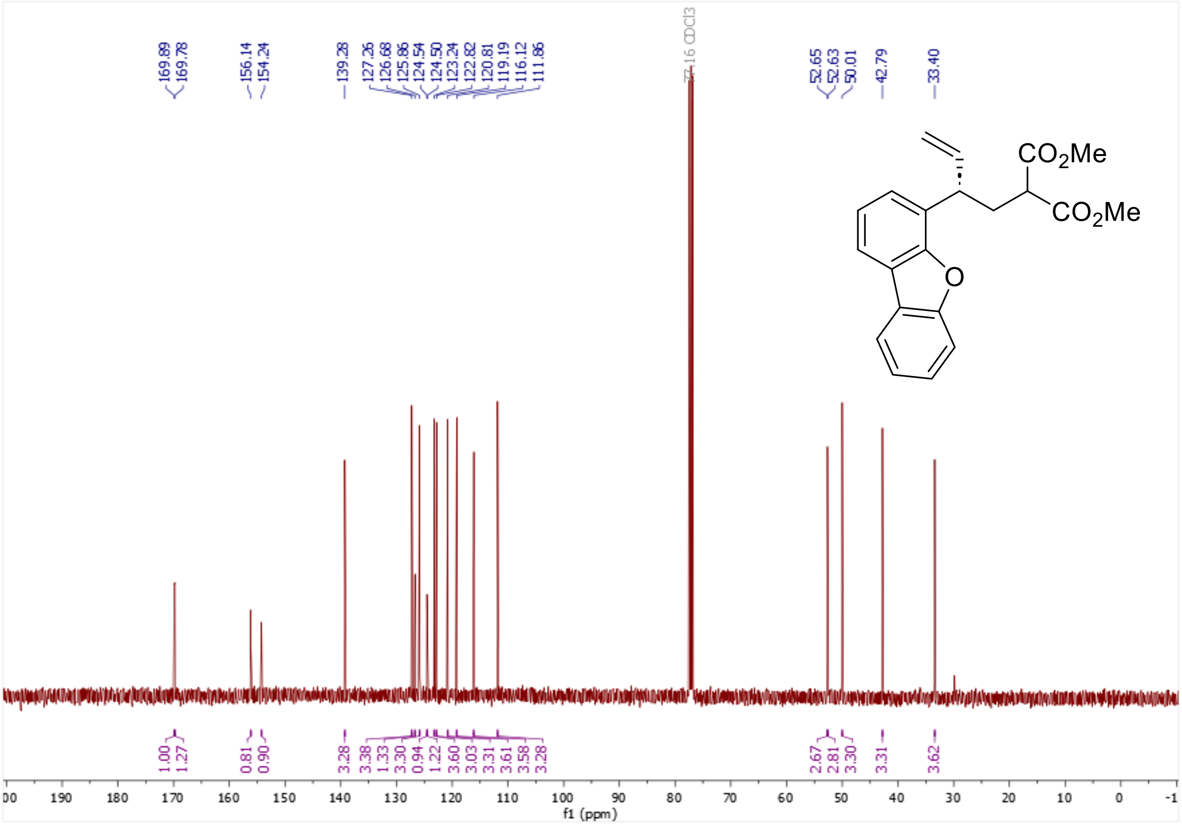

(top) ^{1}H NMR (400 MHz) and (bottom) ^{13}C NMR (101 MHz) spectra of **3y**.

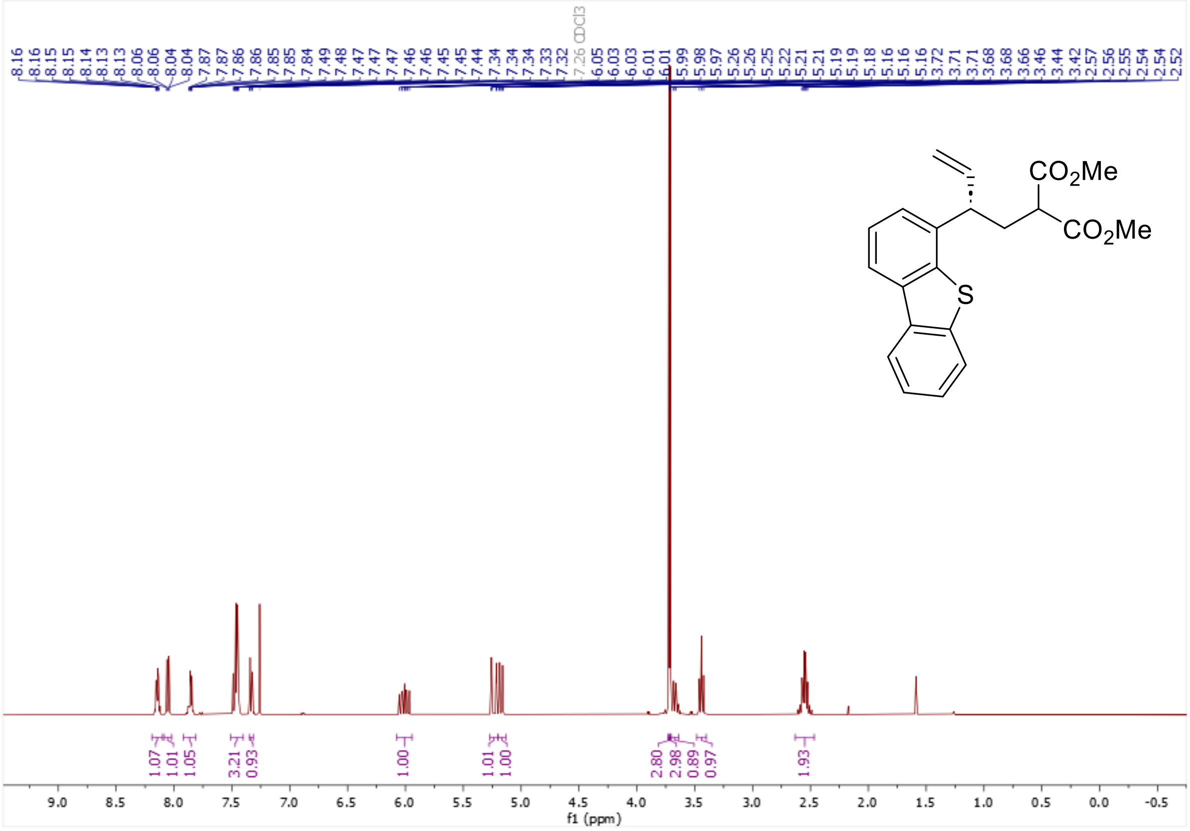

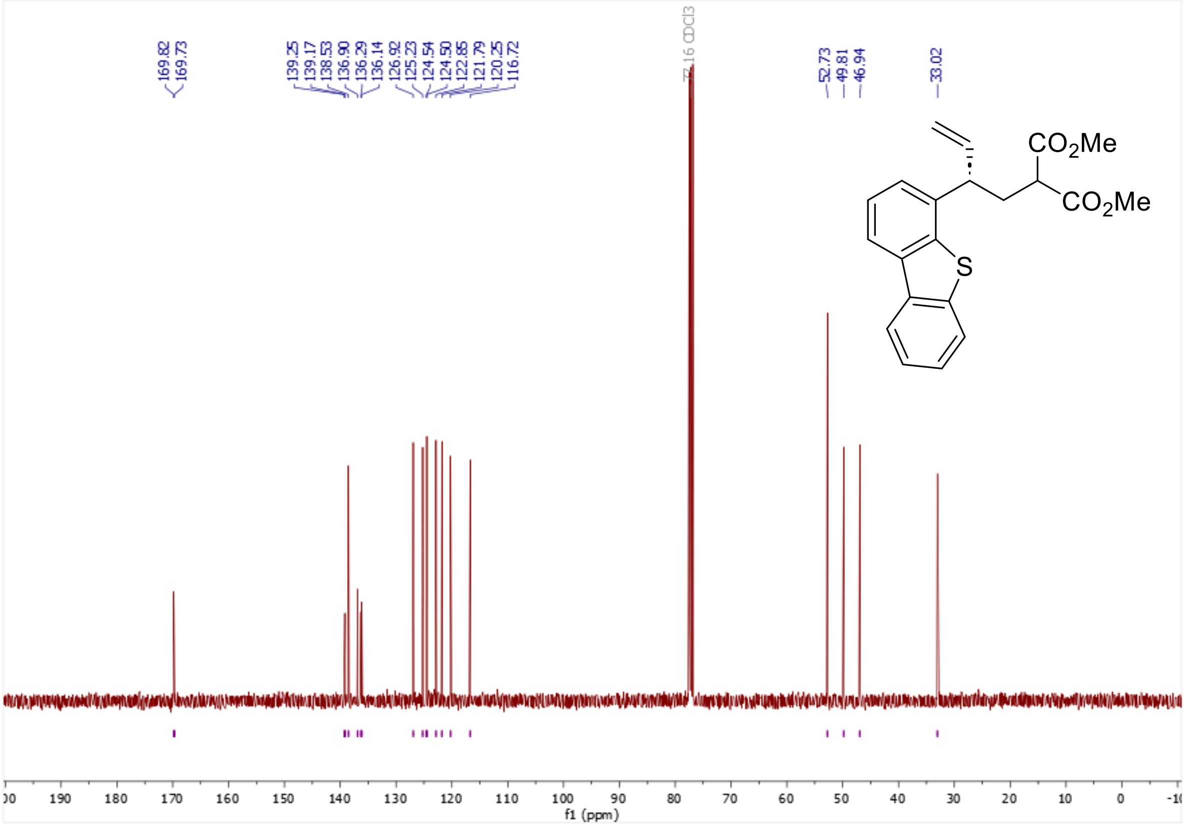

(top) ^{1}H NMR (400 MHz) and (bottom) ^{13}C NMR (101 MHz) spectra of **3z**.

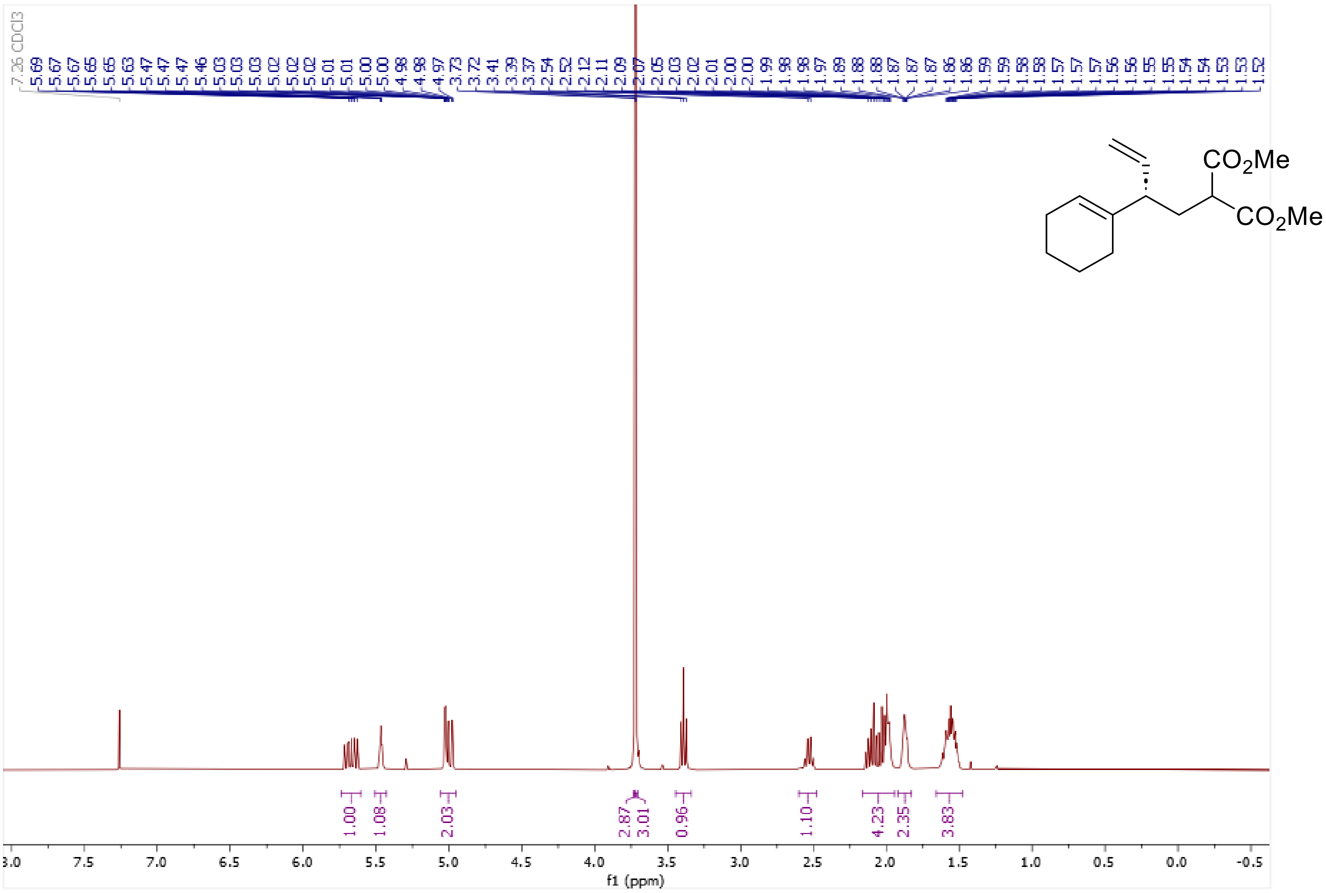

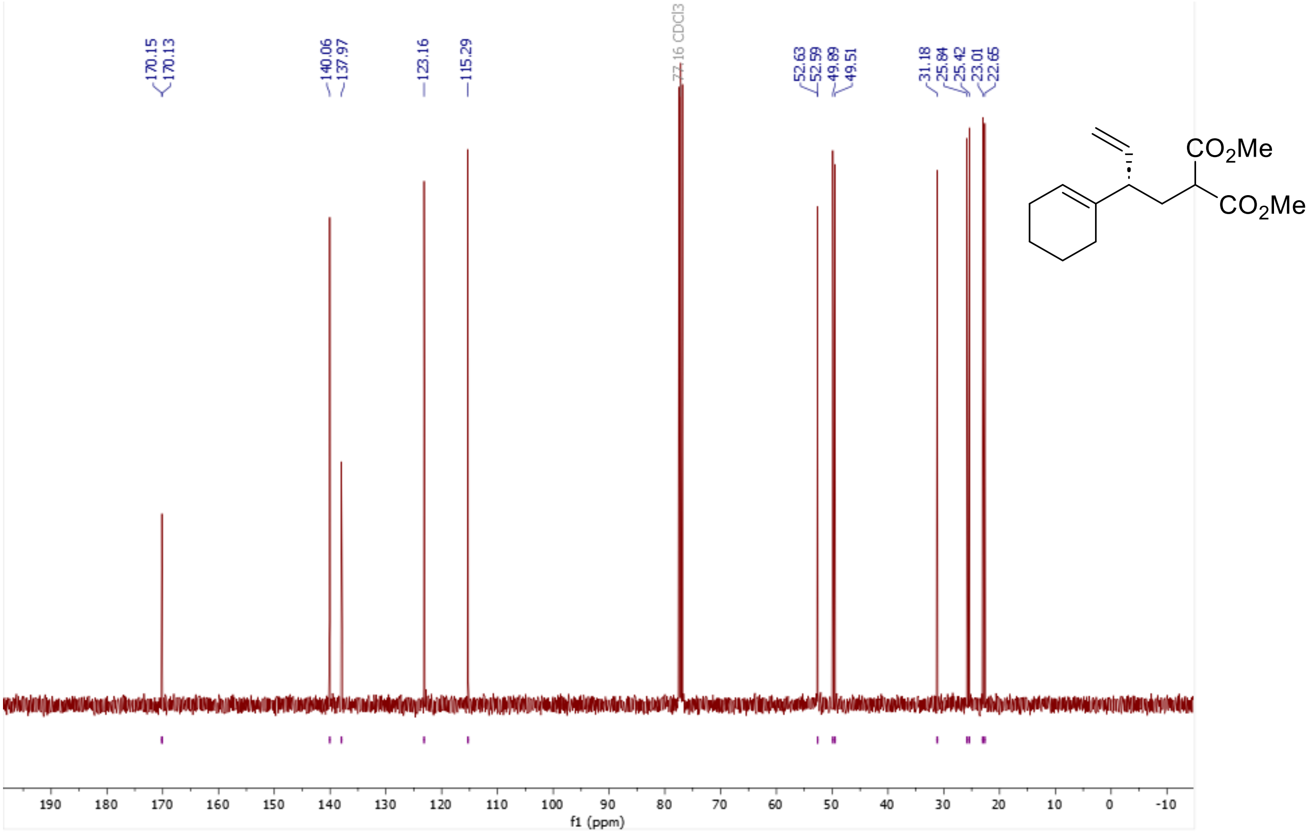

(top) ^{1}H NMR (400 MHz) and (bottom) ^{13}C NMR (101 MHz) spectra of **3aa**.

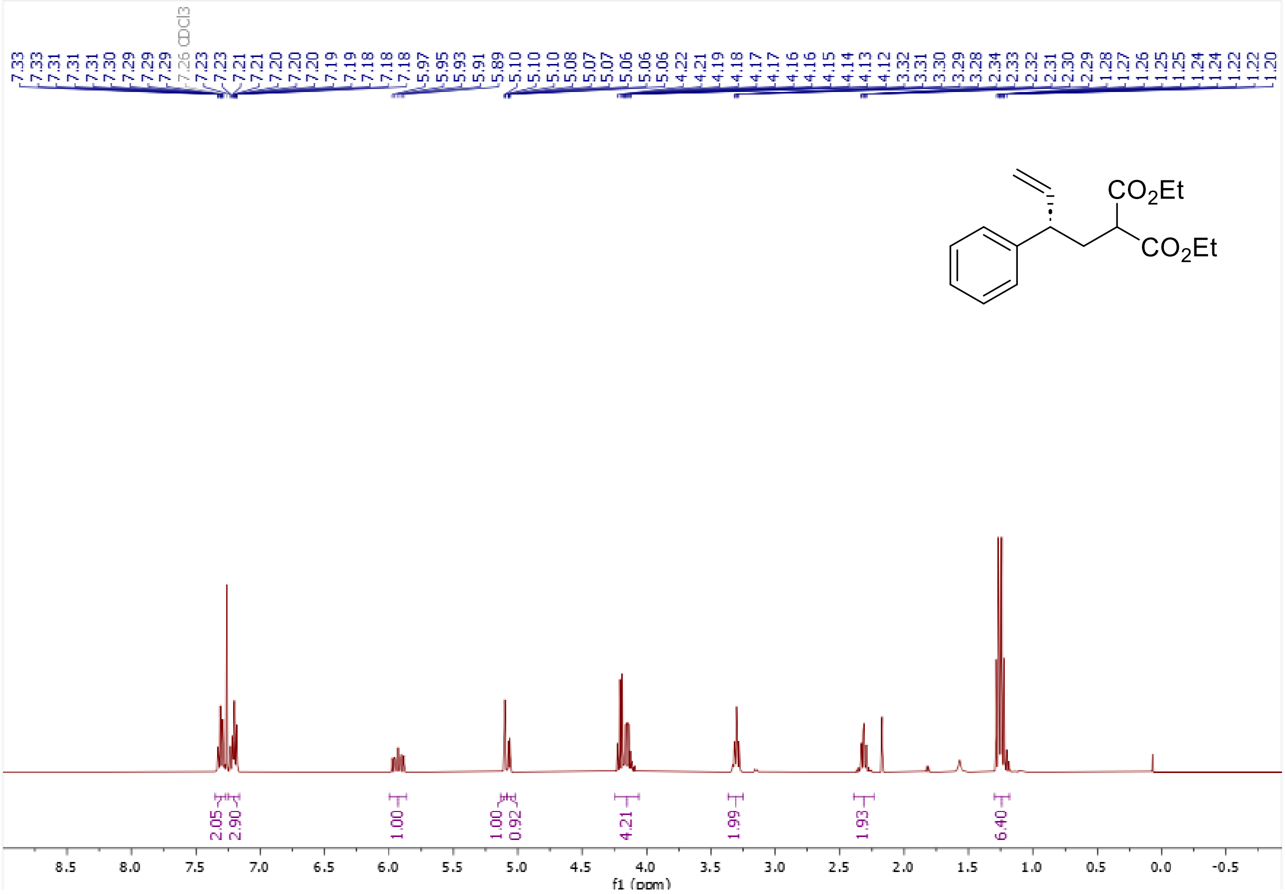

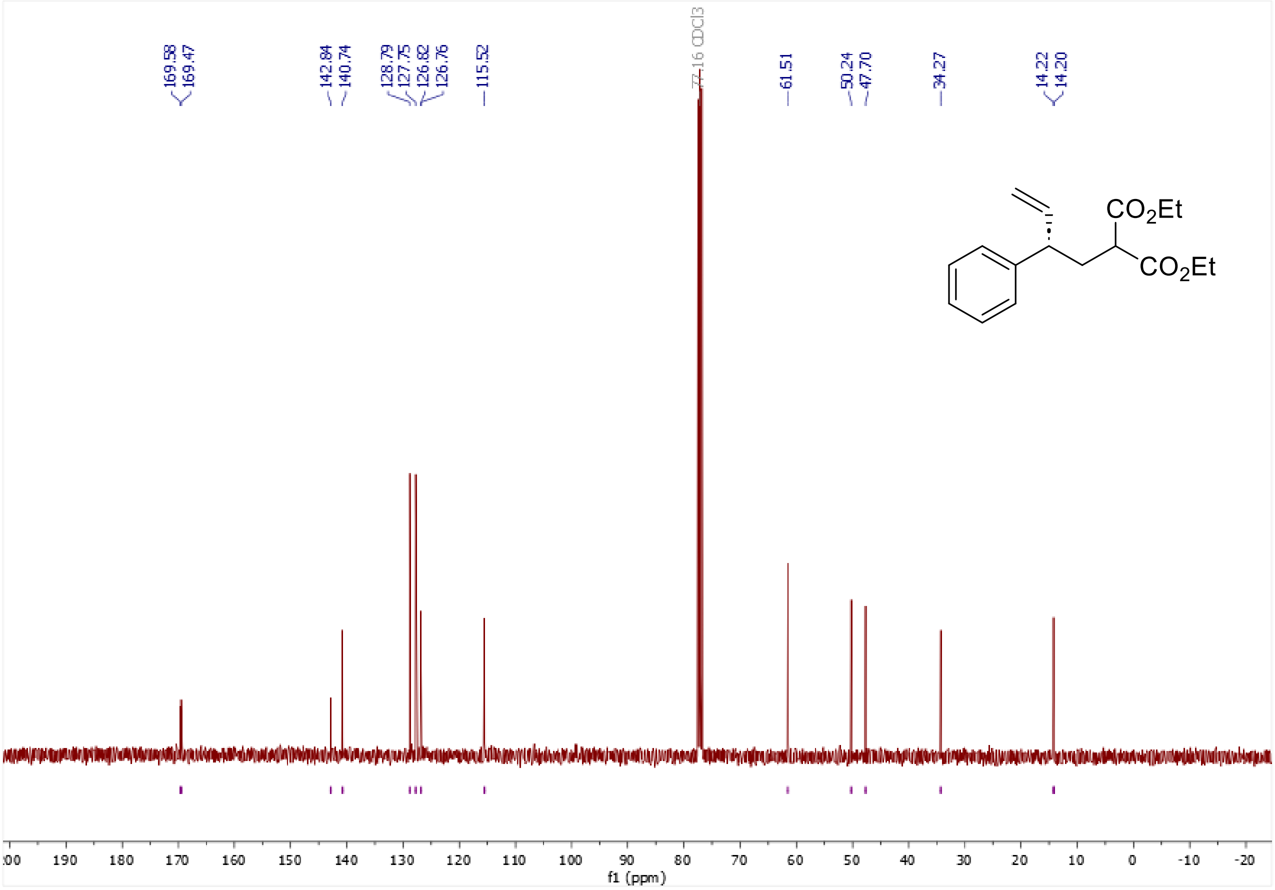

(top) ^{1}H NMR (400 MHz) and (bottom) ^{13}C NMR (101 MHz) spectra of **4a**.

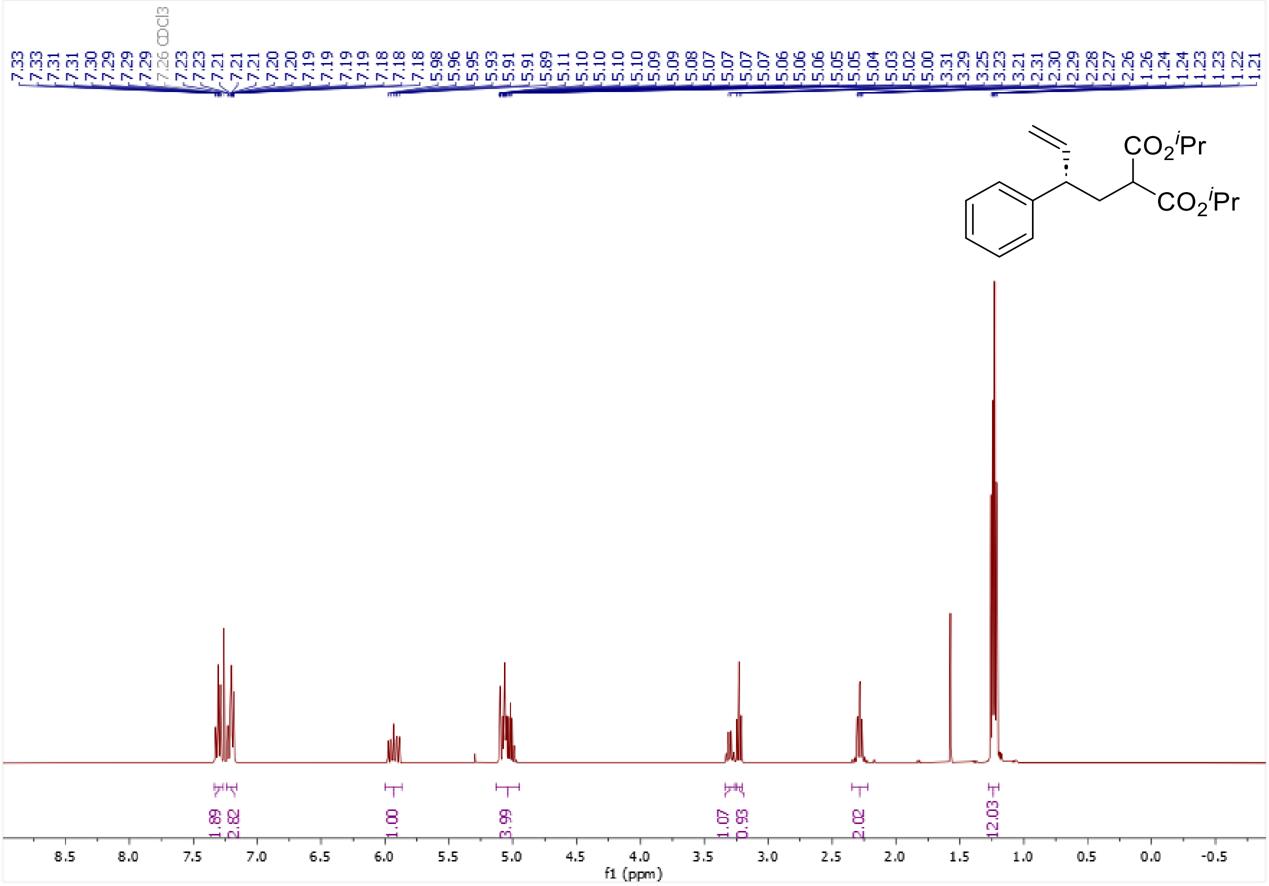

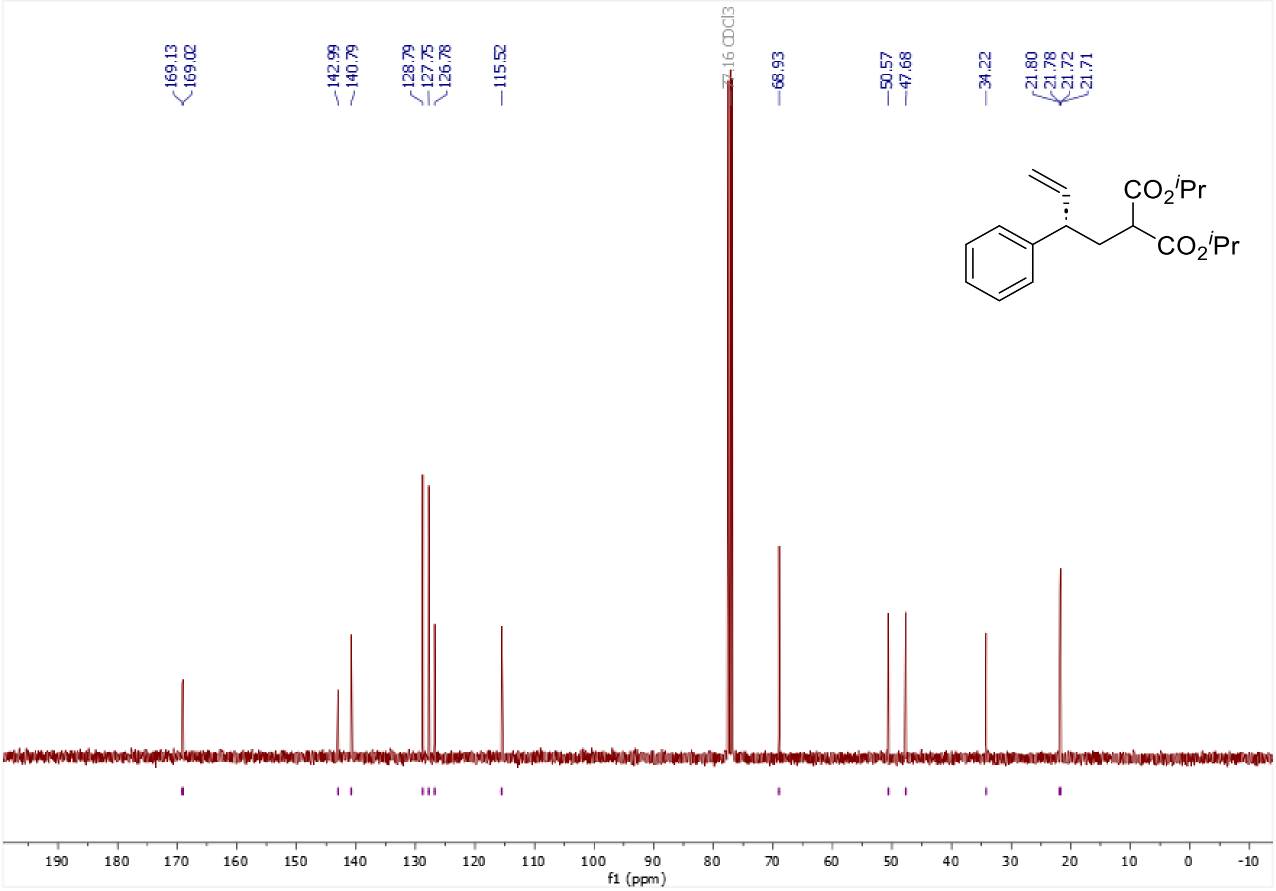

(top) ^{1}H NMR (400 MHz) and (bottom) ^{13}C NMR (101 MHz) spectra of **5a**.

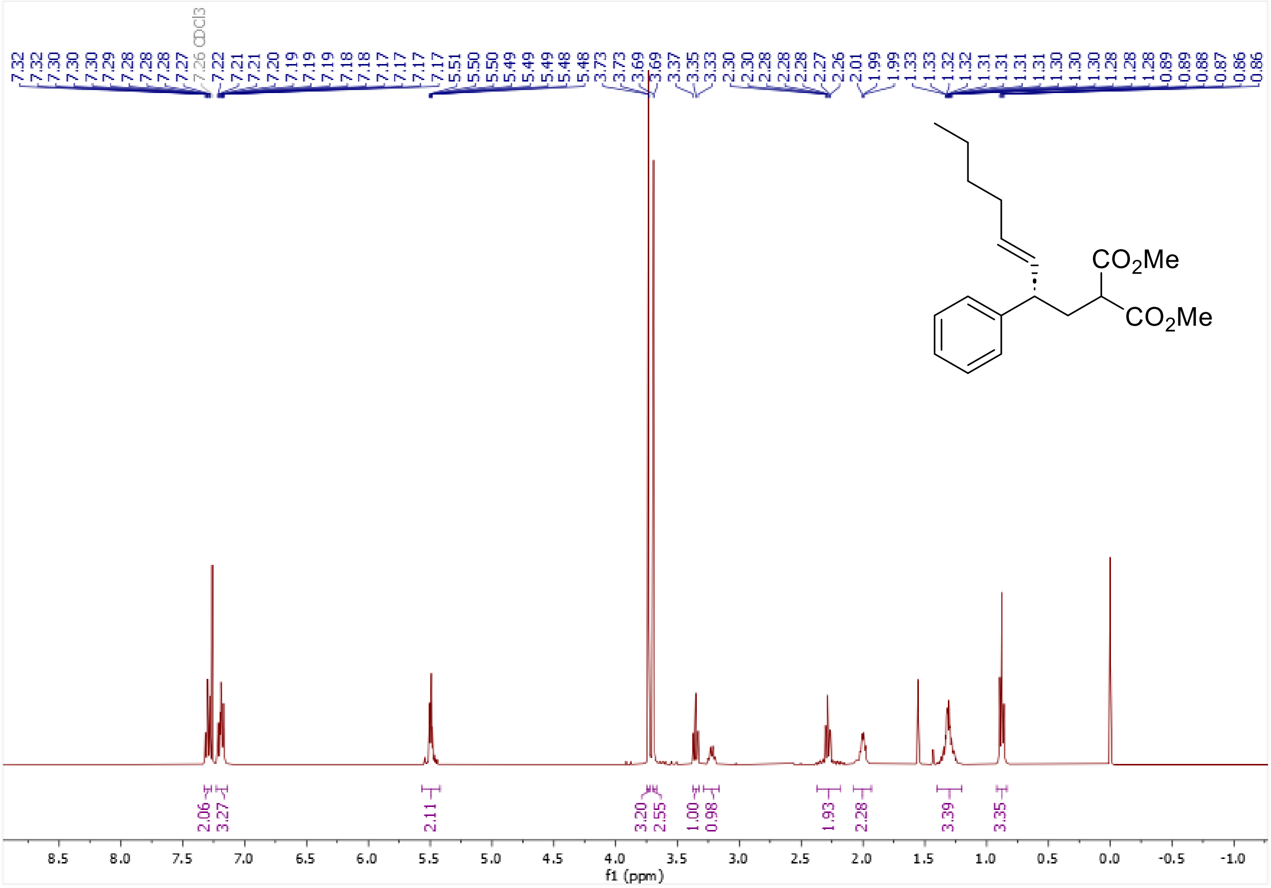

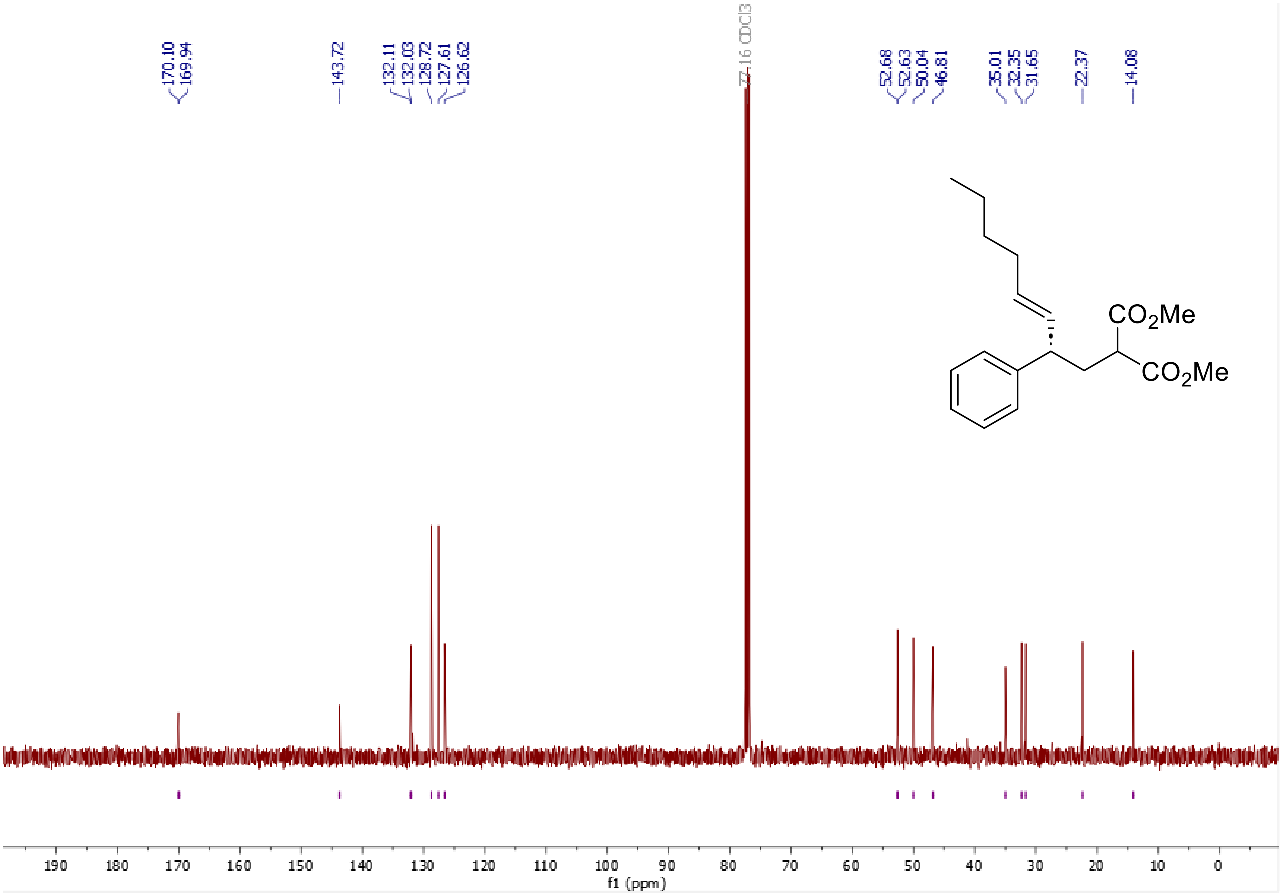

(top) ^{1}H NMR (400 MHz) and (bottom) ^{13}C NMR (101 MHz) spectra of **7a**.

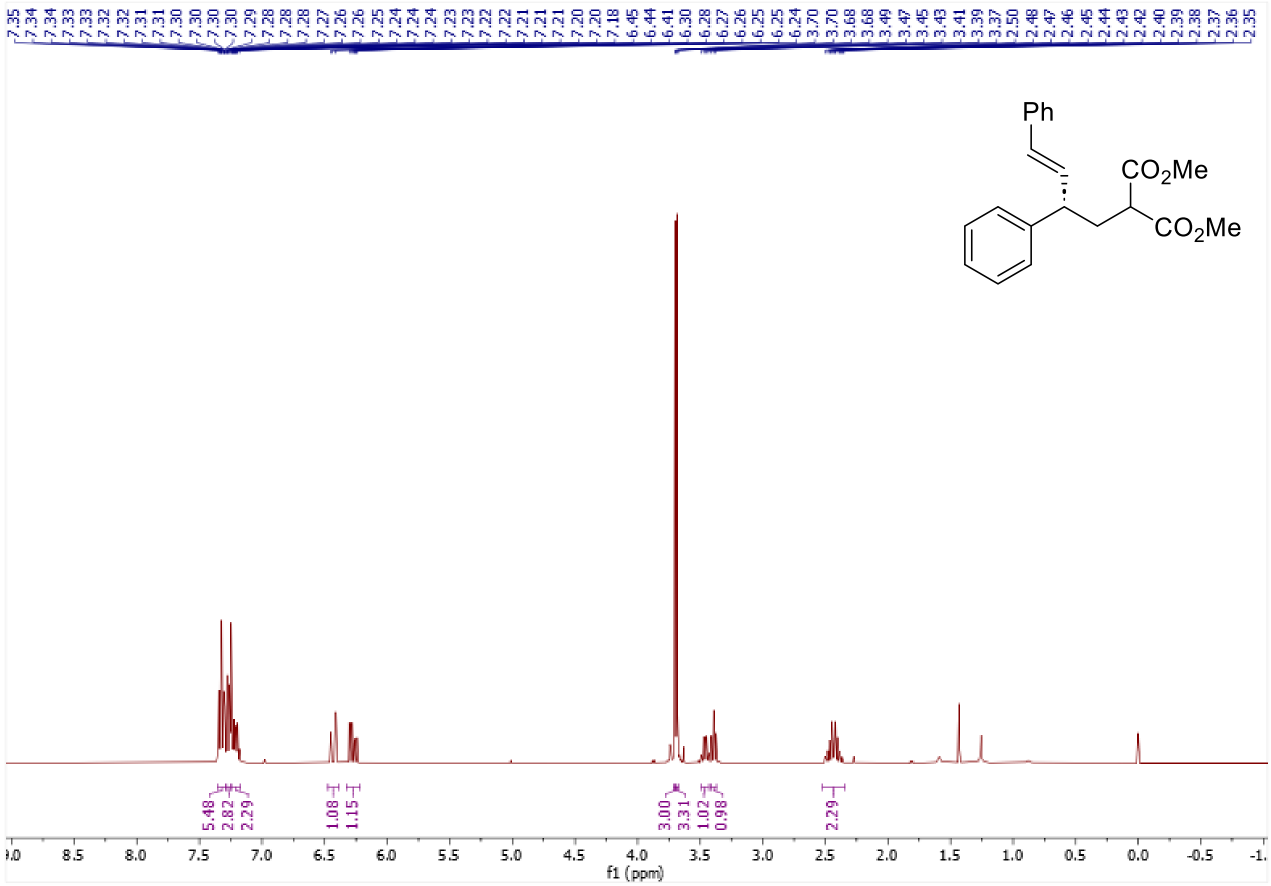

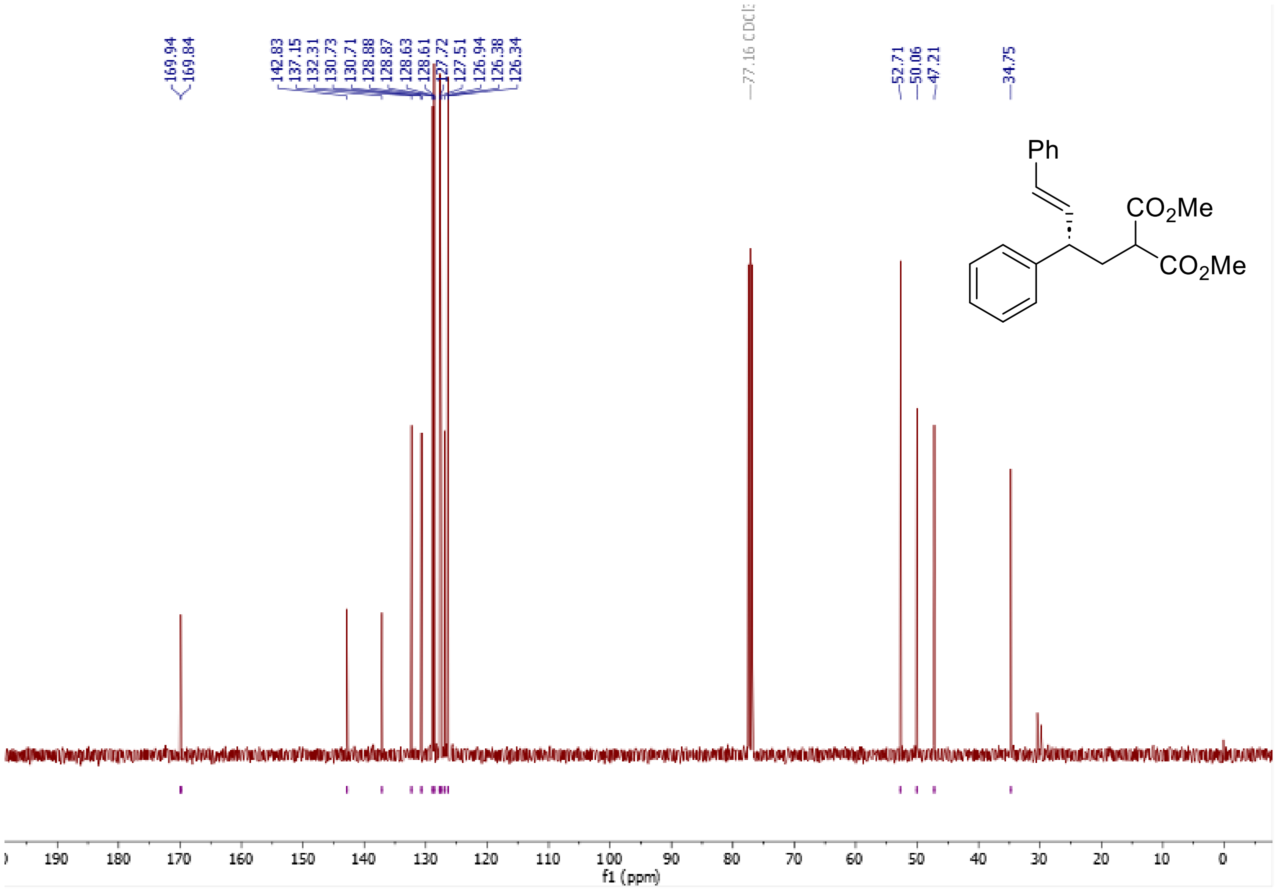

(top) ^{1}H NMR (400 MHz) and (bottom) ^{13}C NMR (101 MHz) spectra of **7b**.

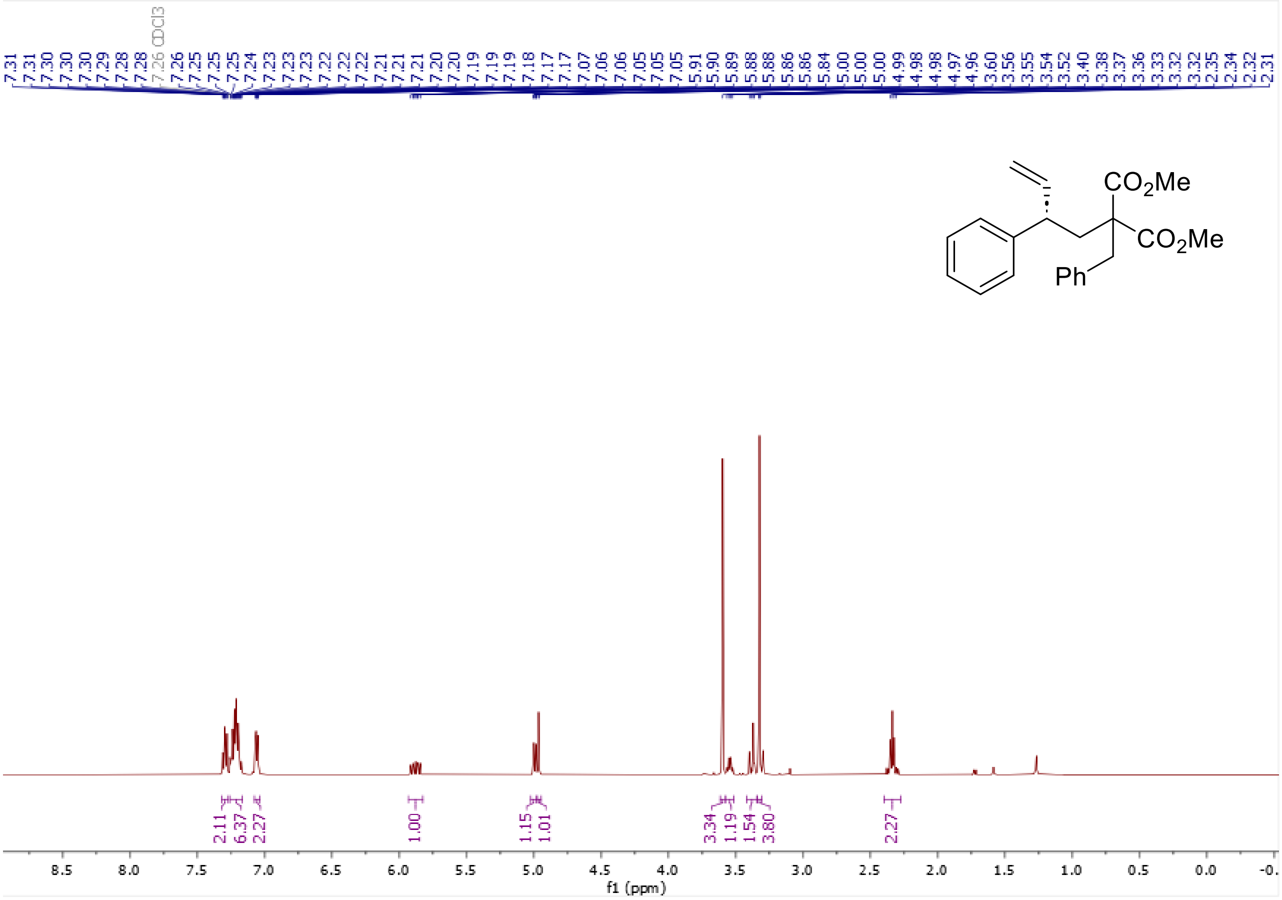

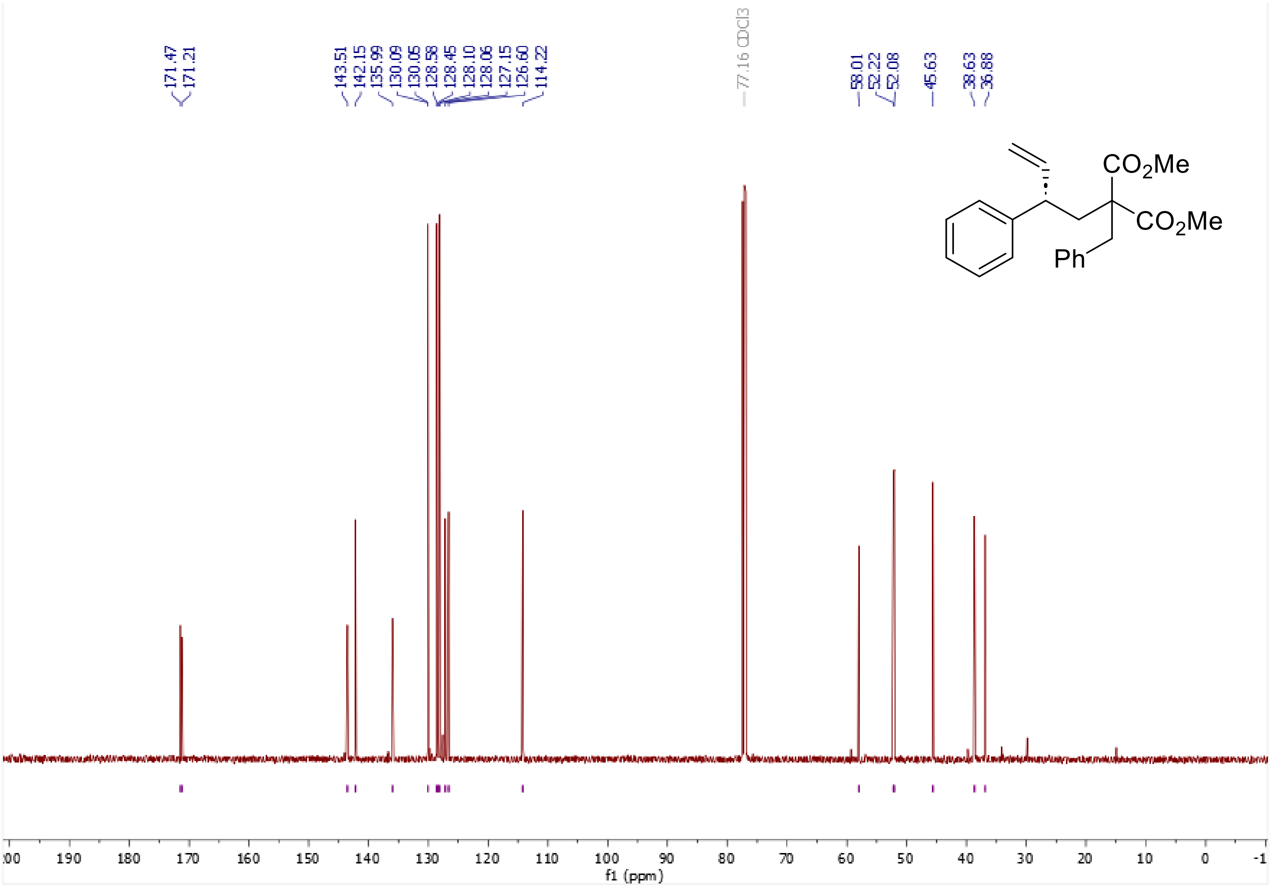

(top) ^{1}H NMR (400 MHz) and (bottom) ^{13}C NMR (101 MHz) spectra of **8**.

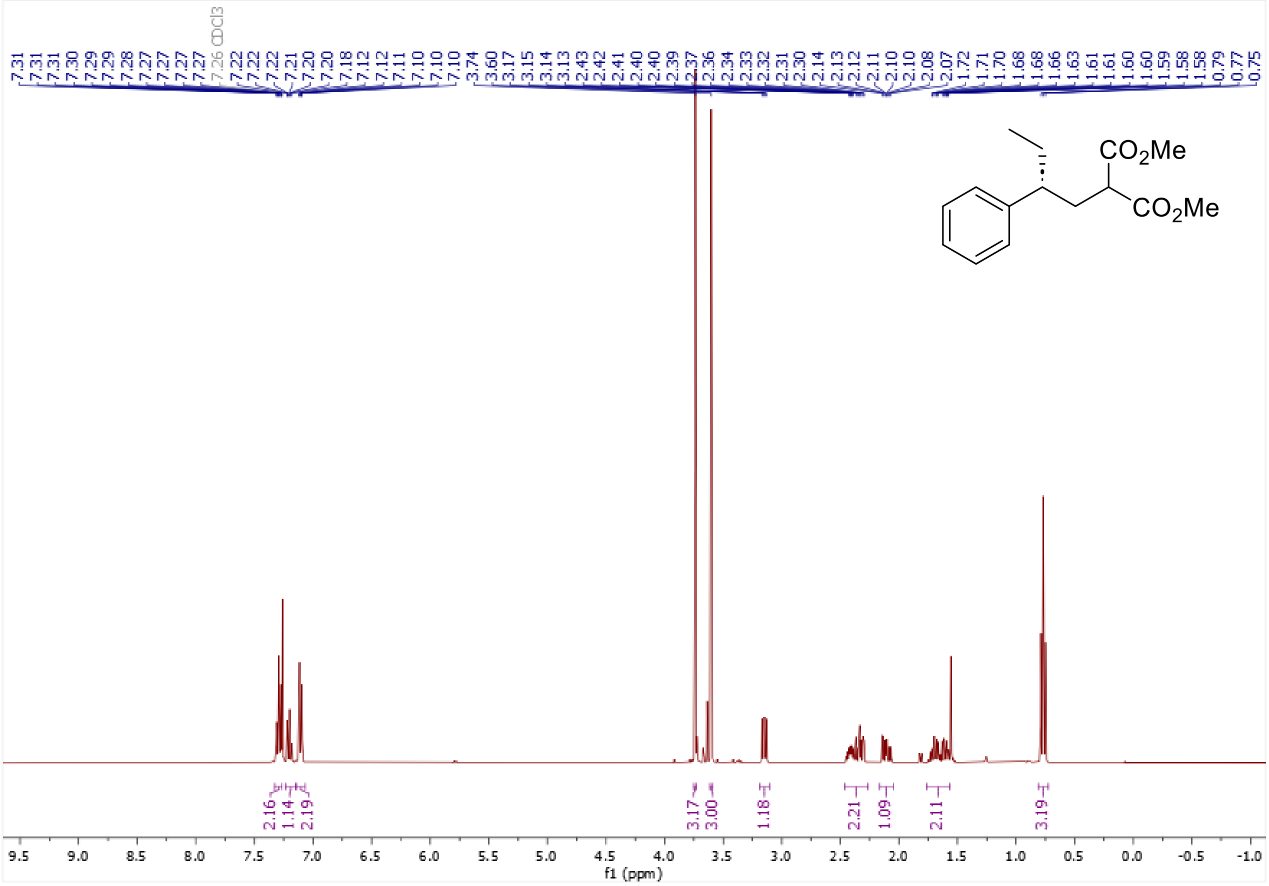

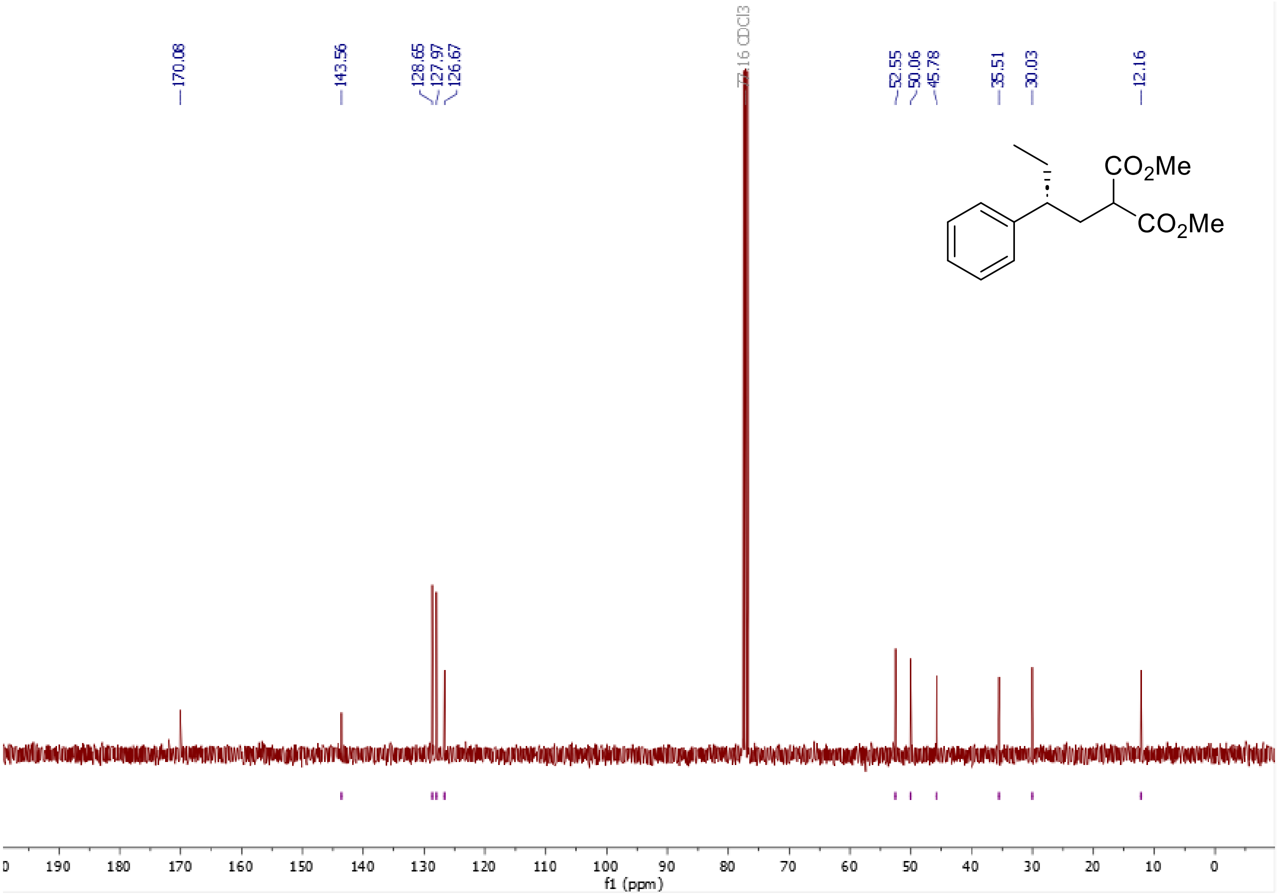

(top) ^{1}H NMR (400 MHz) and (bottom) ^{13}C NMR (101 MHz) spectra of **9**

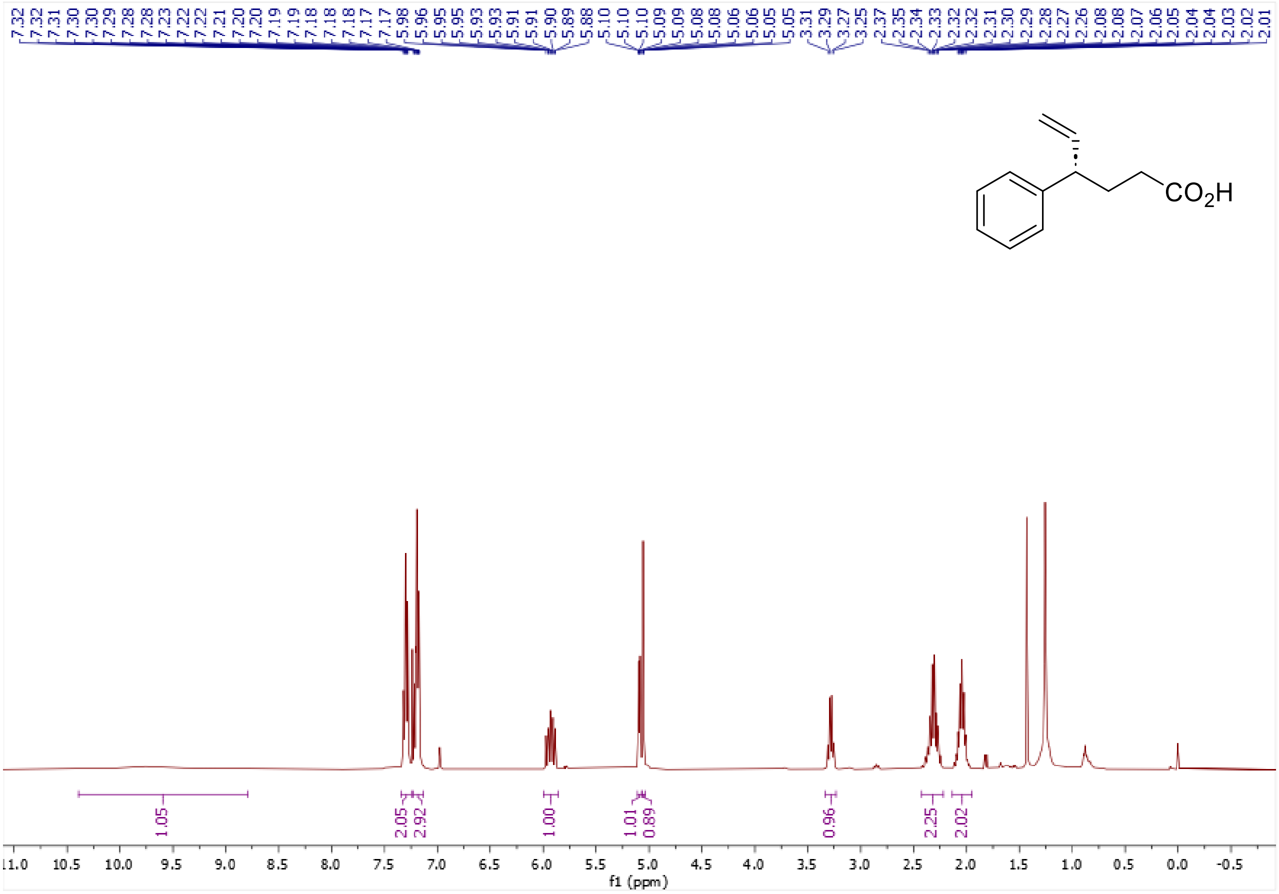

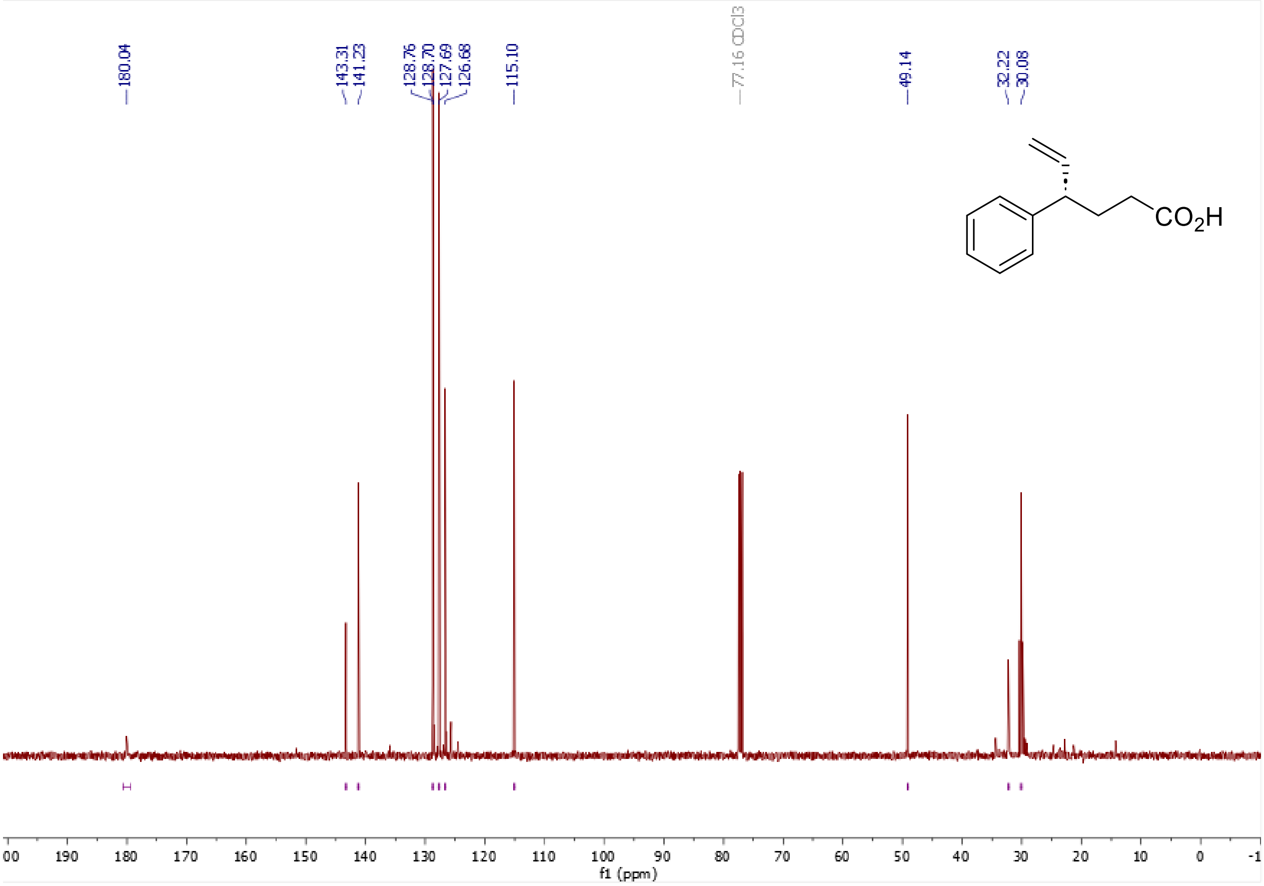

(top) ^{1}H NMR (400 MHz) and (bottom) ^{13}C NMR (101 MHz) spectra of **10**.

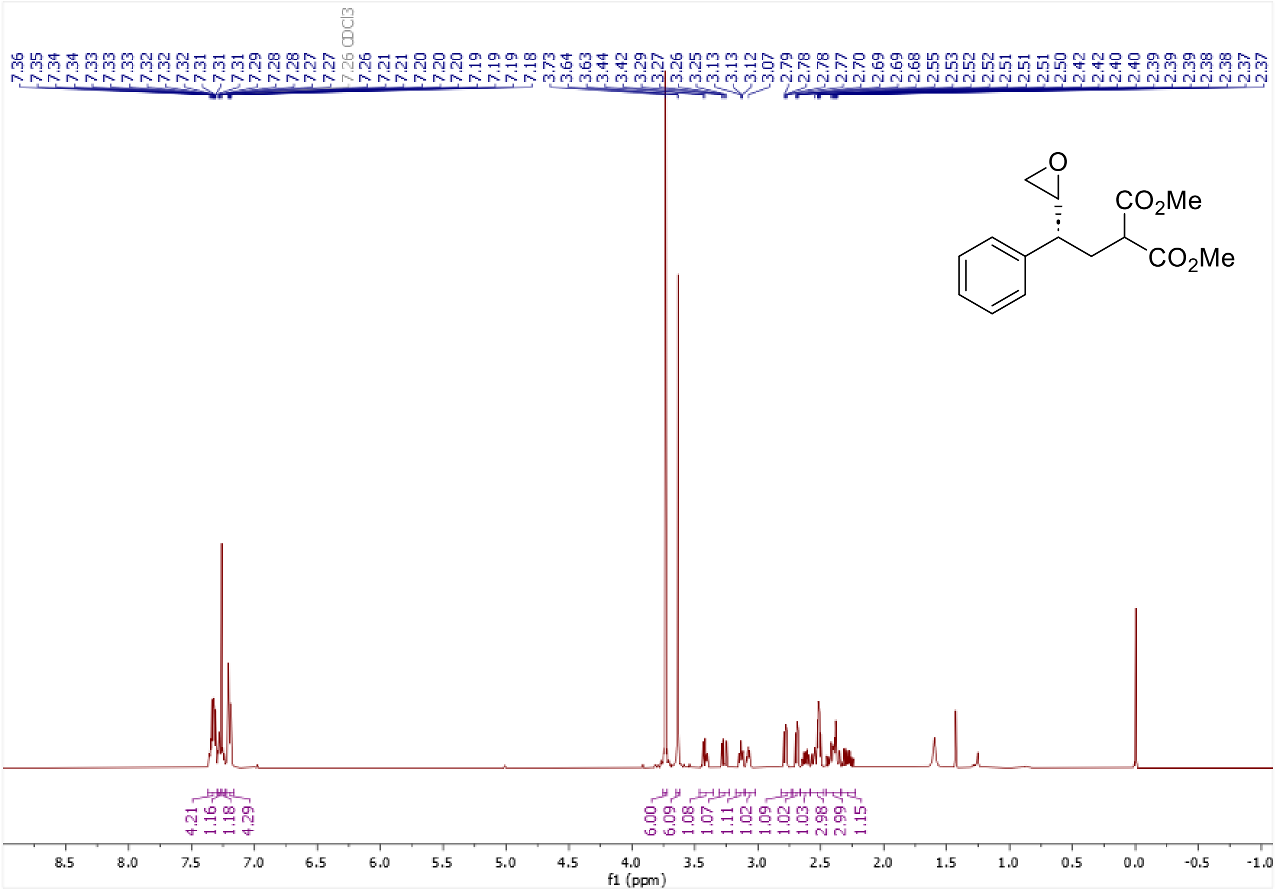

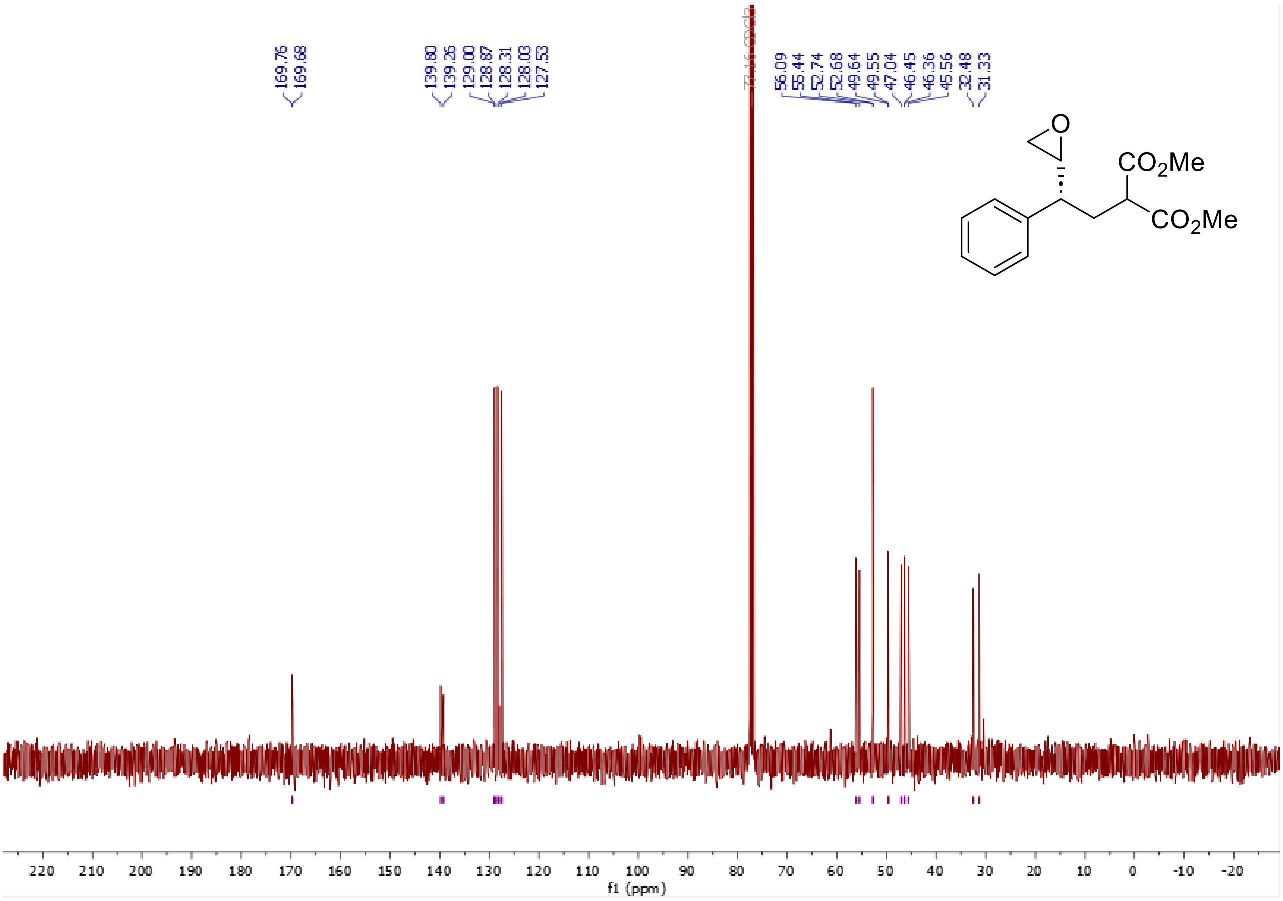

(top) ^{1}H NMR (400 MHz) and (bottom) ^{13}C NMR (101 MHz) spectra of **12**.

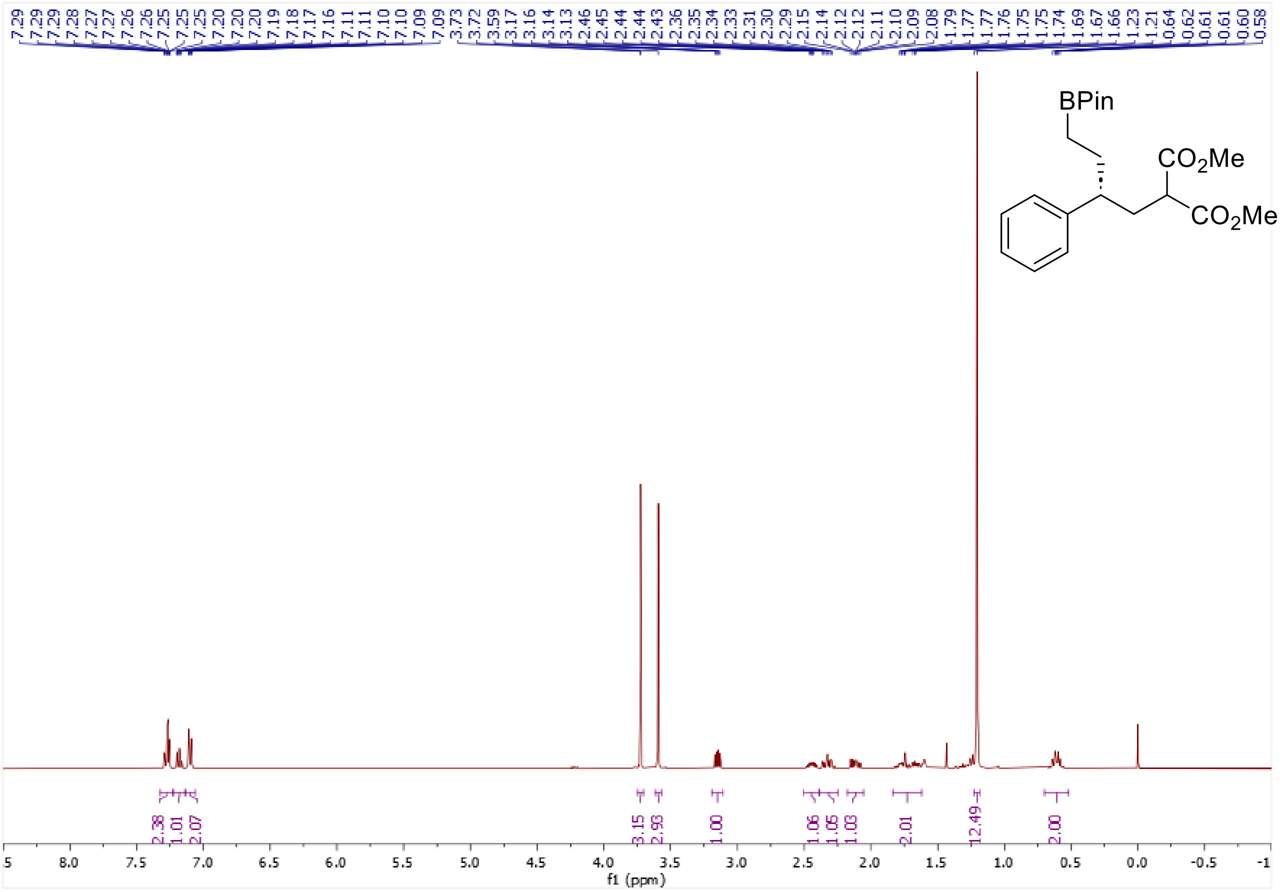

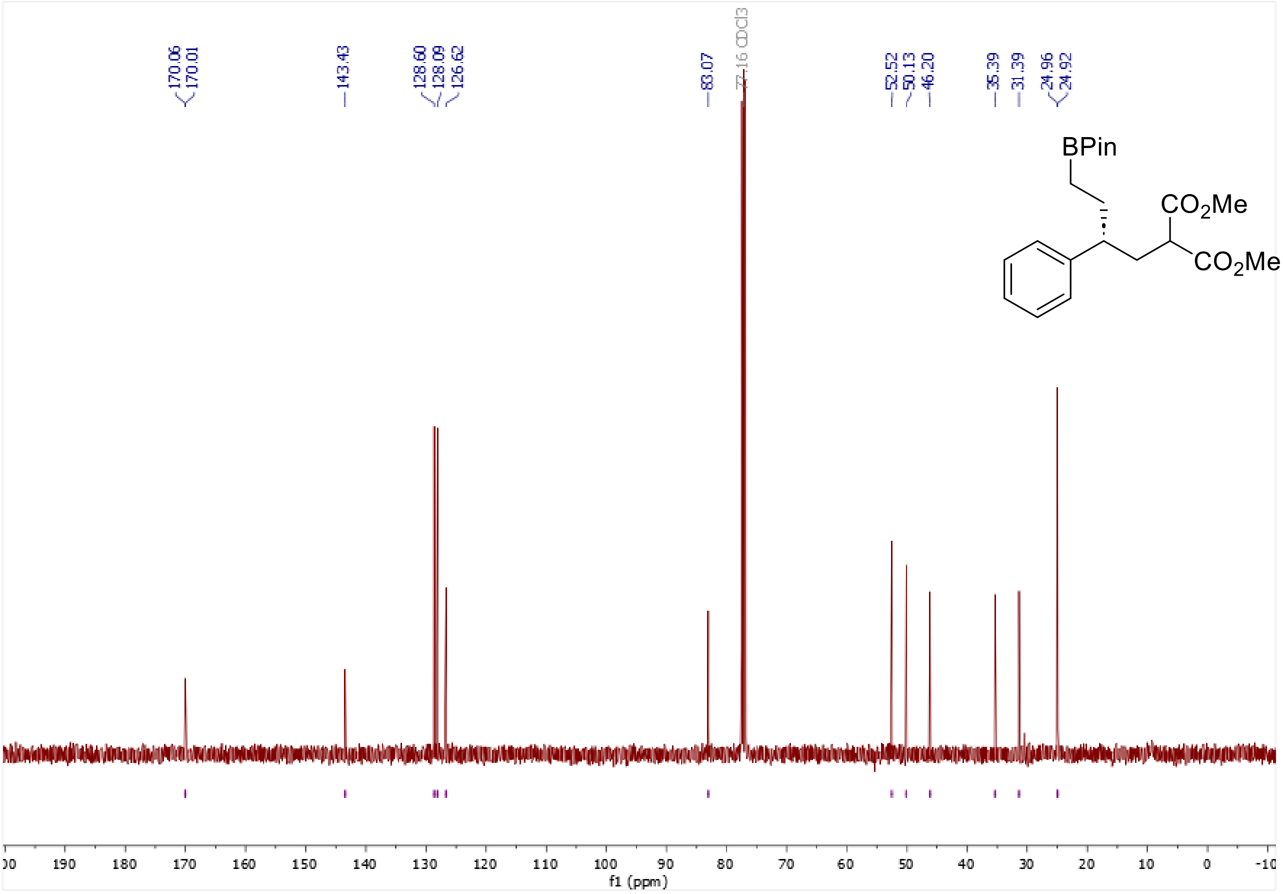

(top) ^{1}H NMR (400 MHz) and (bottom) ^{13}C NMR (101 MHz) spectra of **13**.

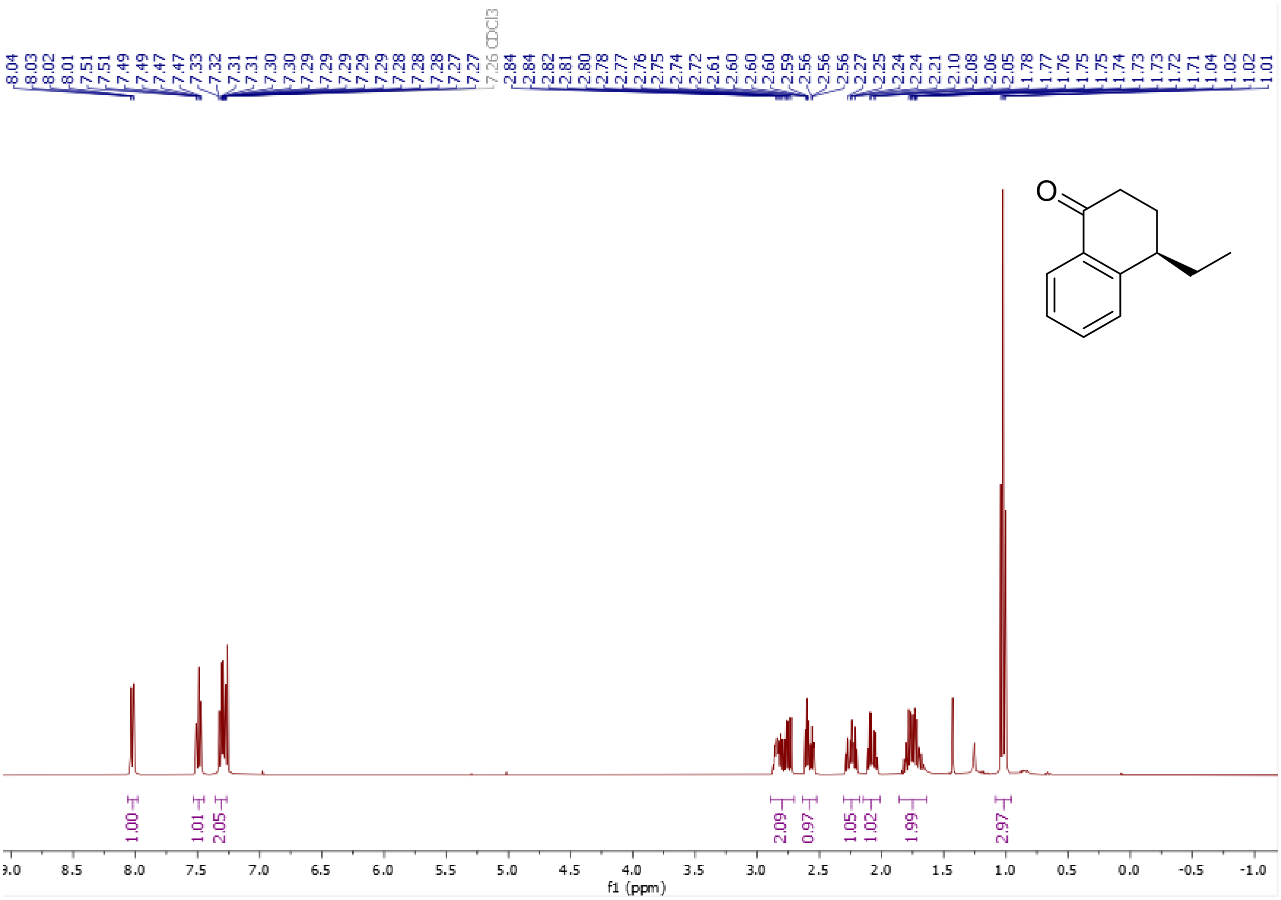

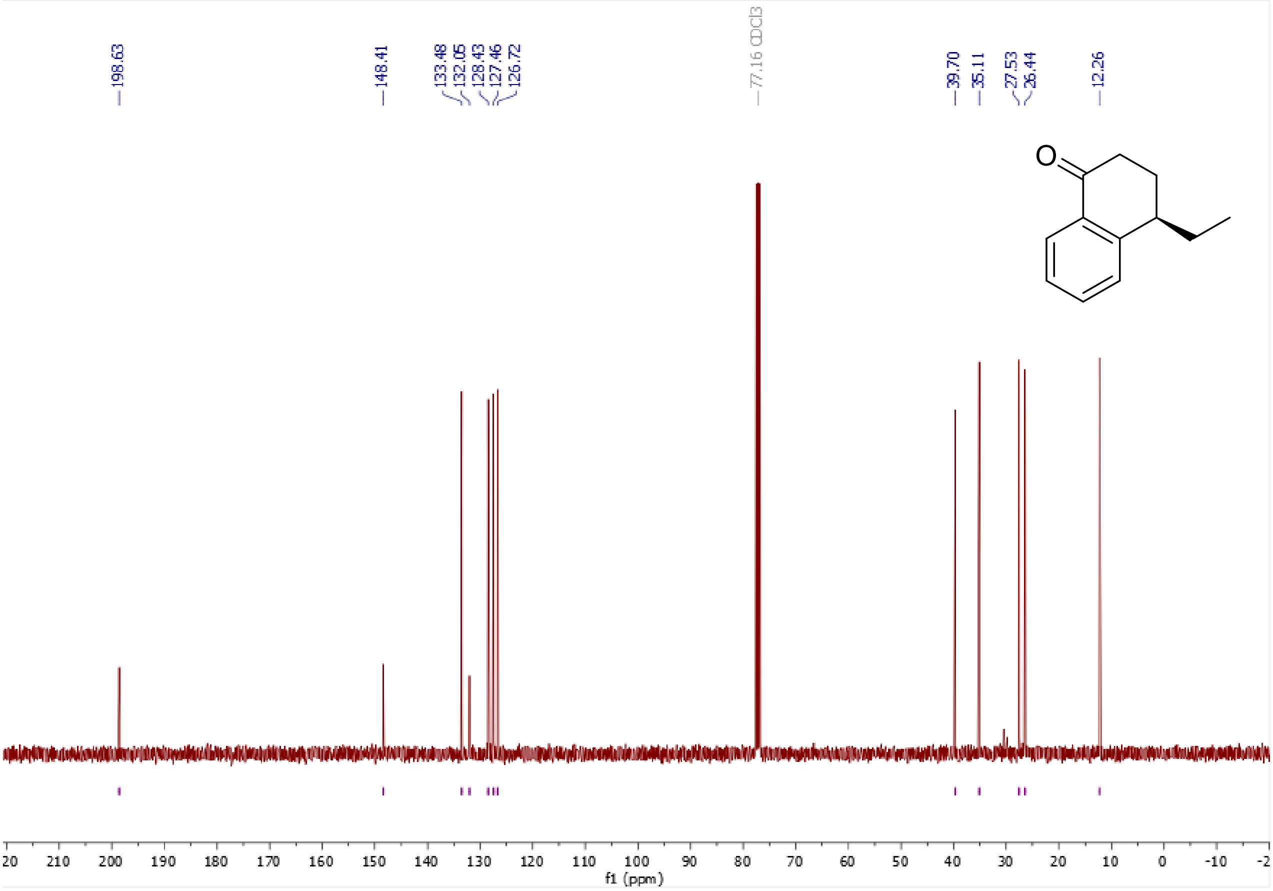

(top) ^{1}H NMR (400 MHz) and (bottom) ^{13}C NMR (101 MHz) spectra of **14**.

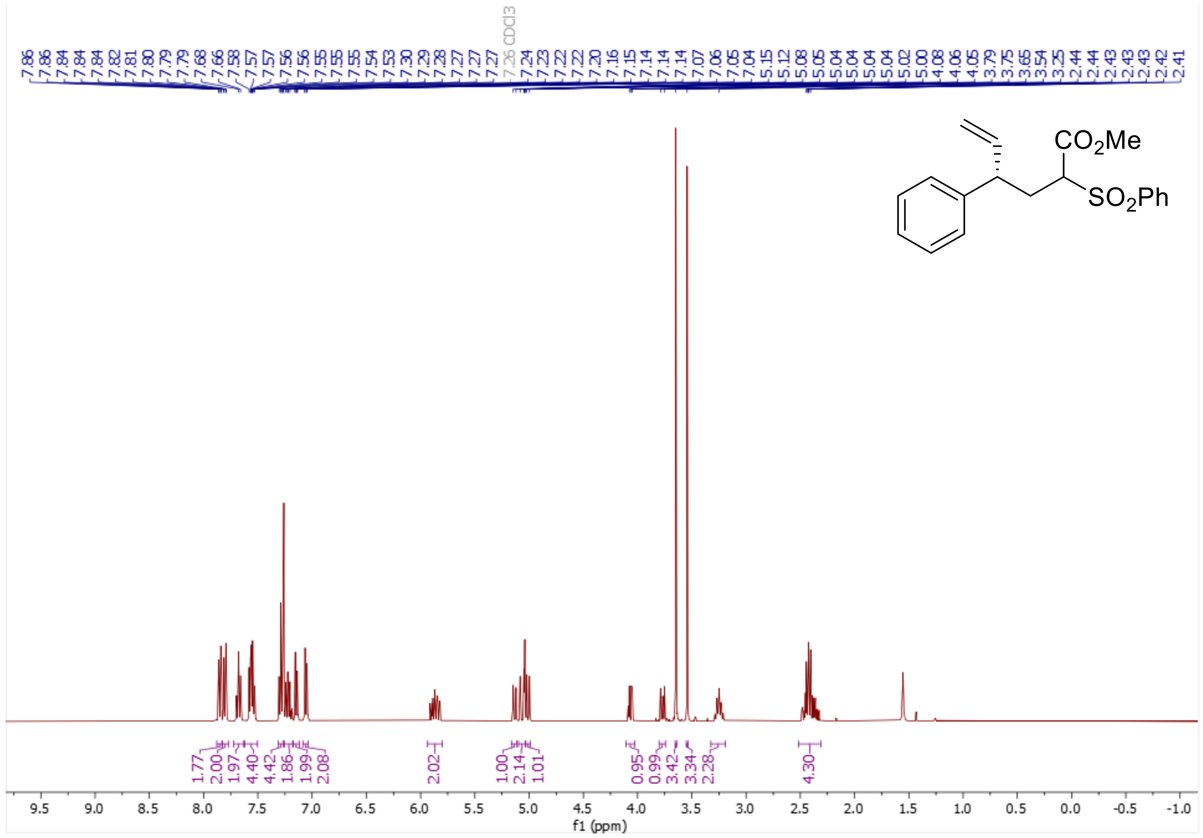

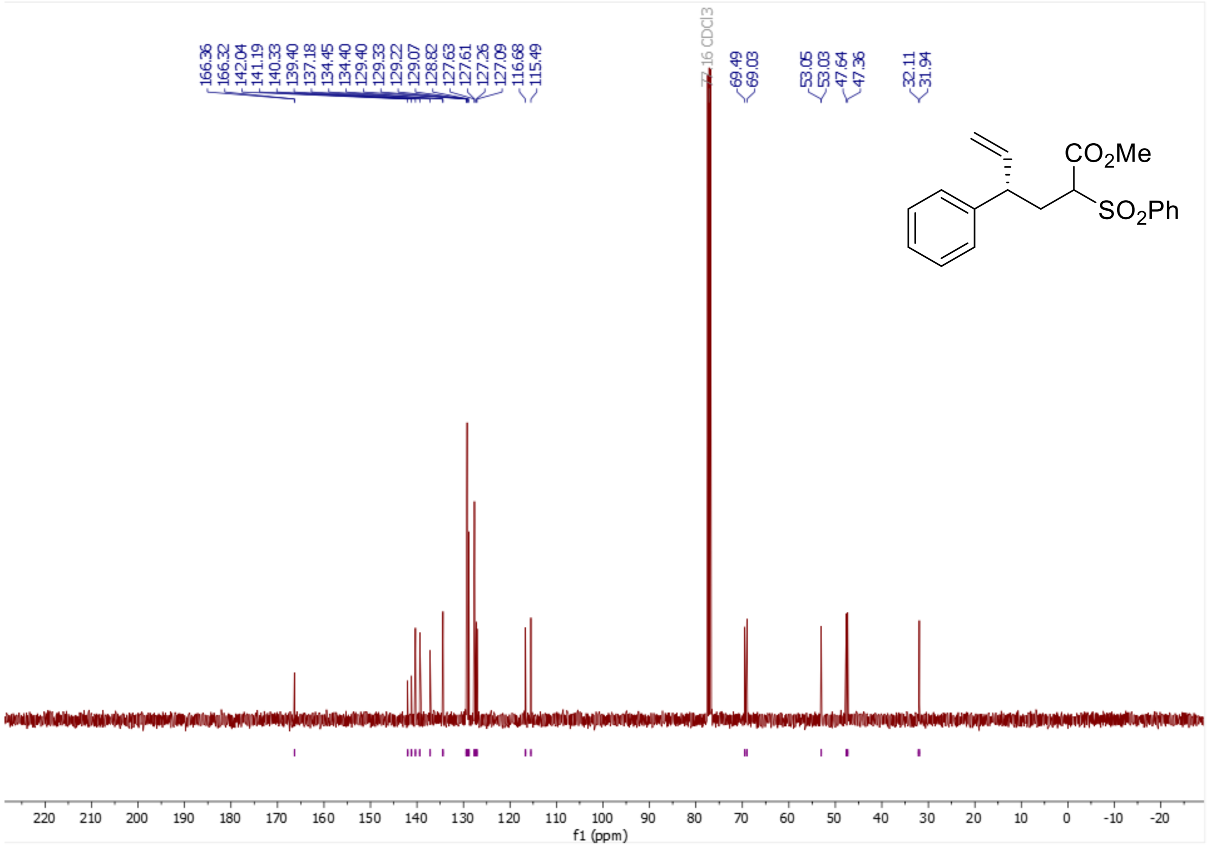

(top) ^{1}H NMR (400 MHz) and (bottom) ^{13}C NMR (101 MHz) spectra of **22**

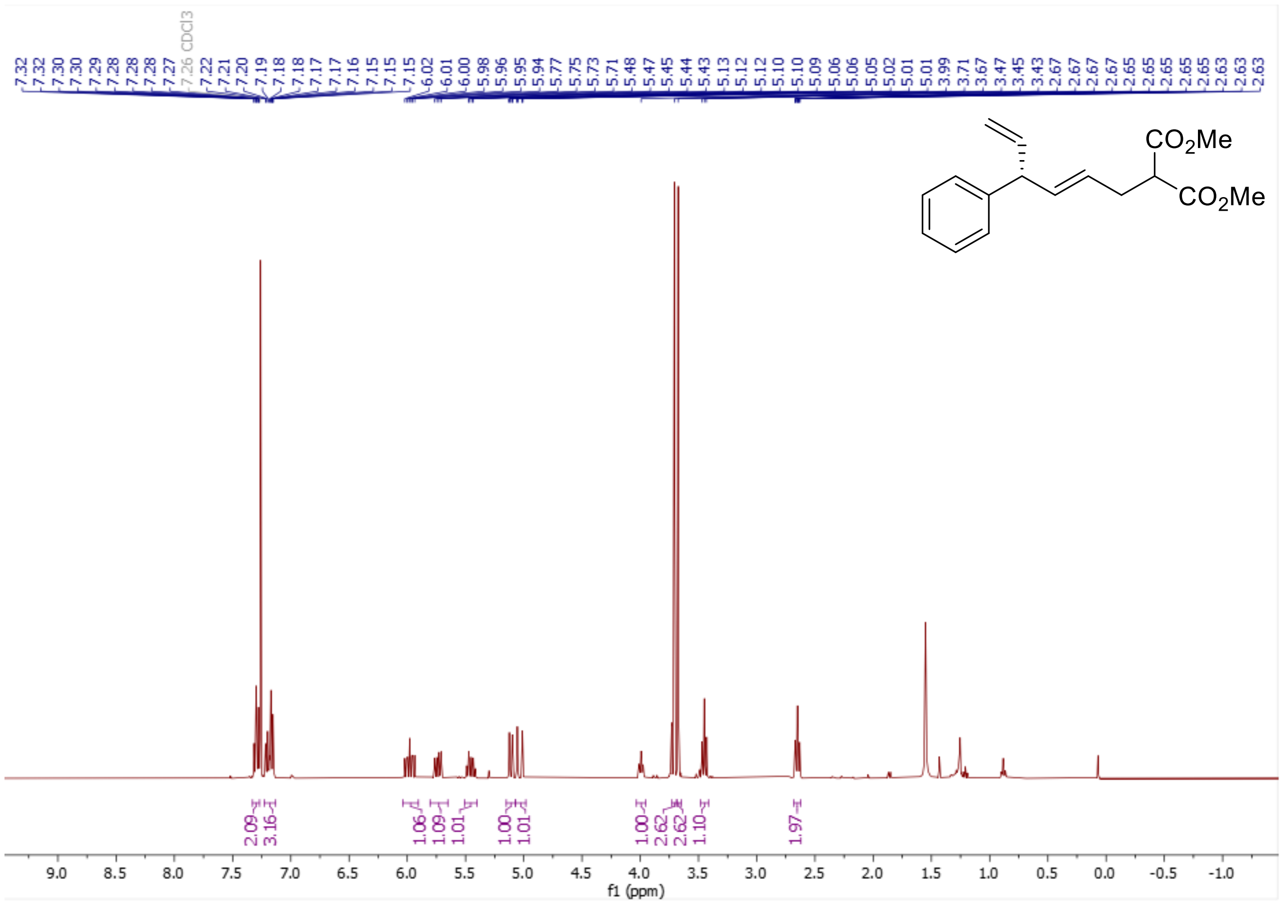

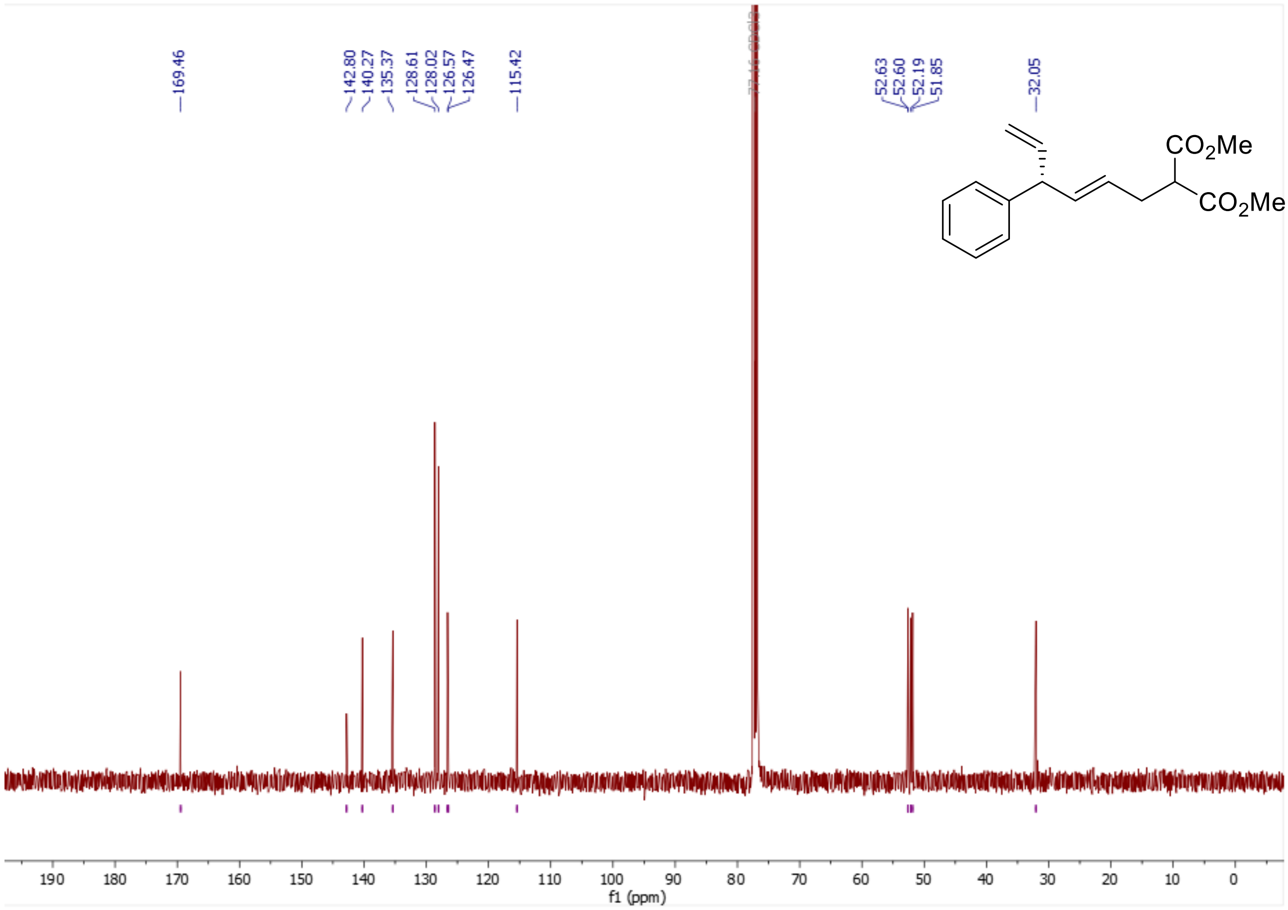

(top) ^{1}H NMR (400 MHz) and (bottom) ^{13}C NMR (101 MHz) spectra of **24**

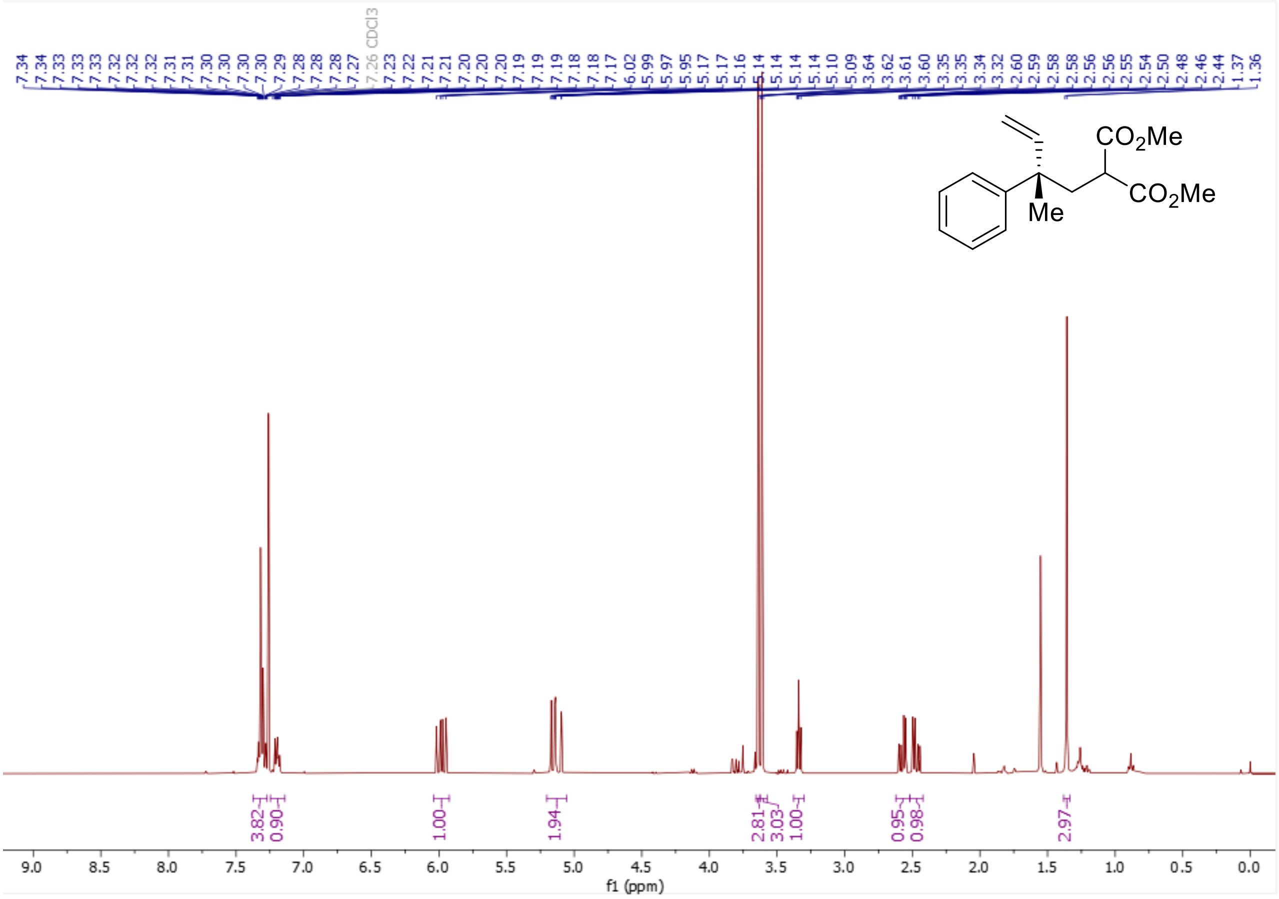

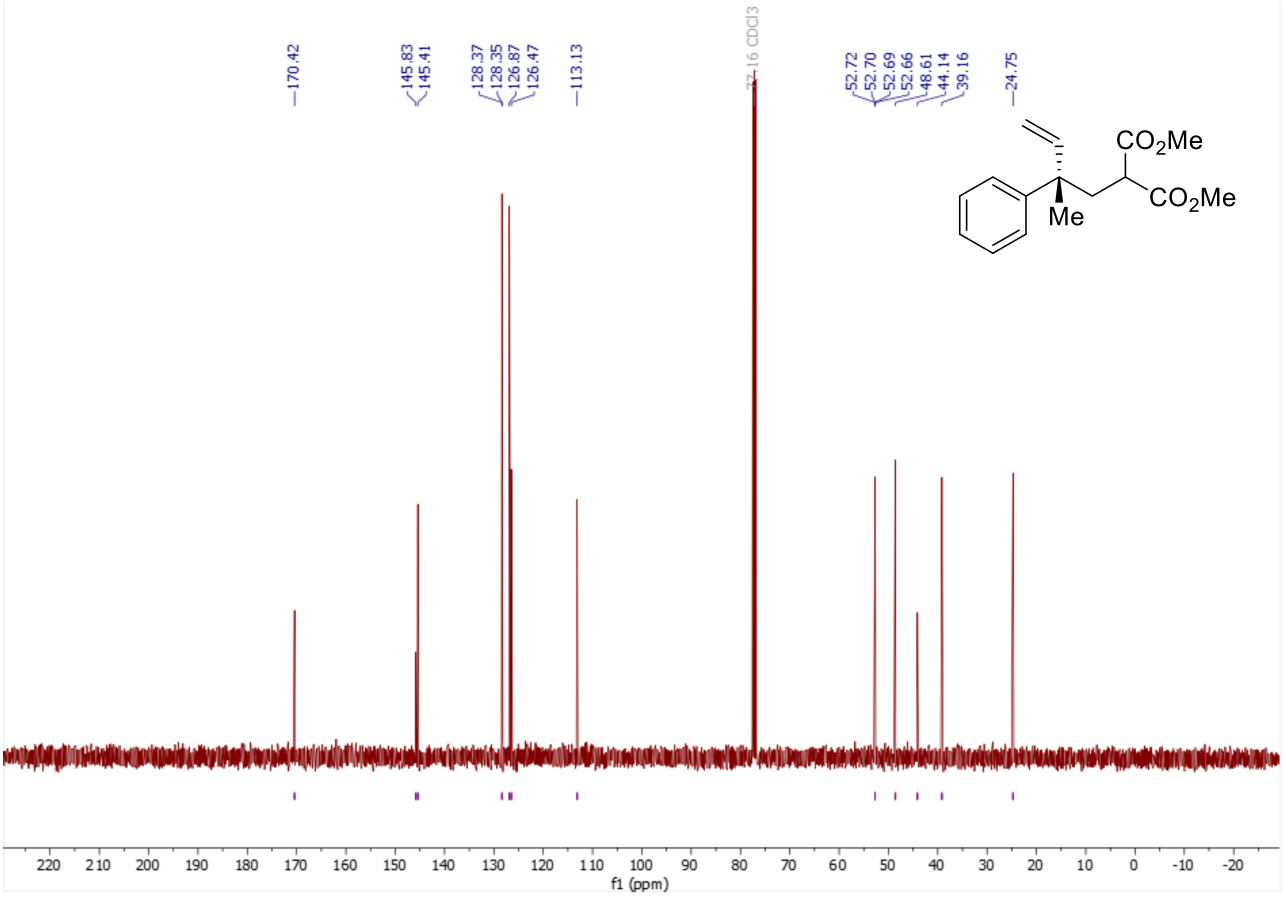

(top) ^{1}H NMR (400 MHz) and (bottom) ^{13}C NMR (101 MHz) spectra of **27**

10. SFC Traces

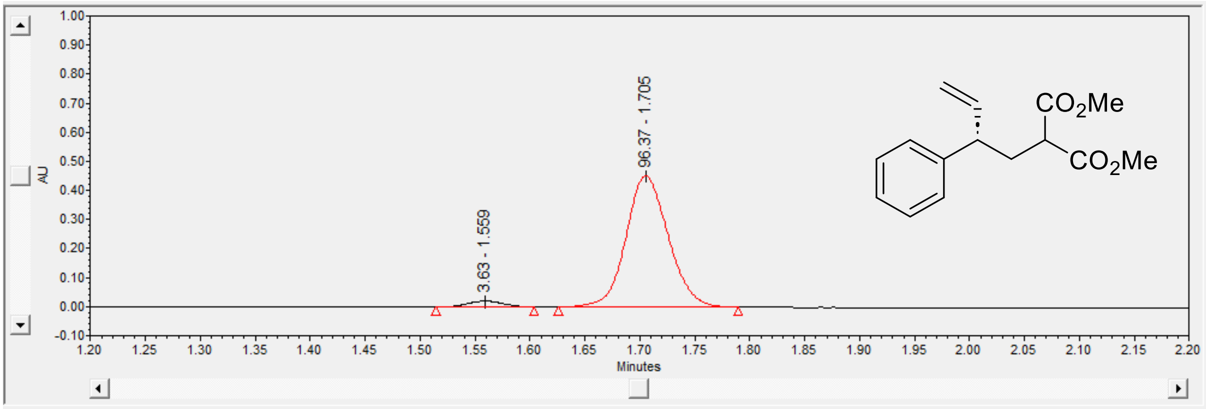

	Name	Retention Time (min)	Purity1 Angle	Purity1 Threshold	PDA/FLR Match1 Spect. Name	PDA/FLR Match1 Angle	PDA/FLR Match1 Threshold	PDA/FLR Match1 Lib. Name	Area (µV*sec)	% Area	Height (µV)	Int Type	Amount	Units	Peak Type	Peak Cod
1		1.559							45653	3.63	20537	bb			Unknown	
2		1.705							1213283	96.37	452136	bb			Unknown	

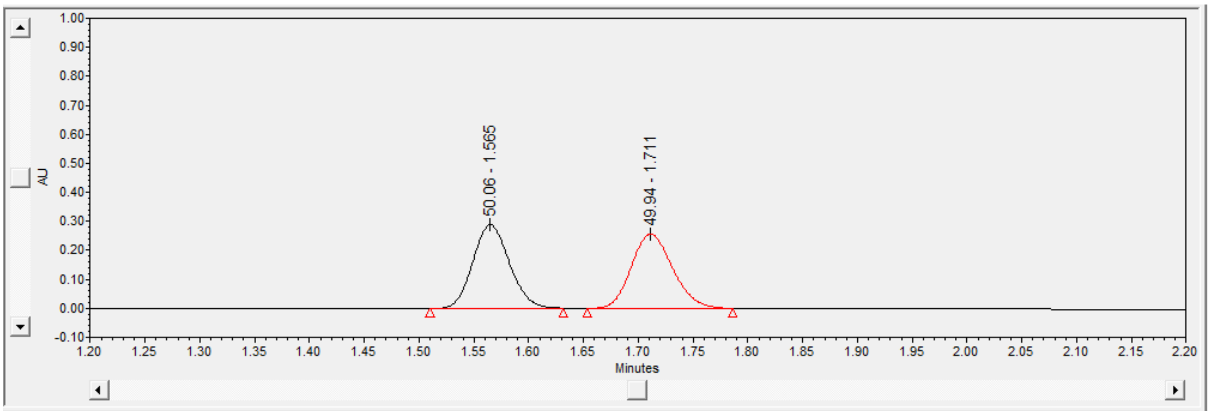

	Name	Retention Time (min)	Purity1 Angle	Purity1 Threshold	PDA/FLR Match1 Spect. Name	PDA/FLR Match1 Angle	PDA/FLR Match1 Threshold	PDA/FLR Match1 Lib. Name	Area (µV*sec)	% Area	Height (µV)	Int Type	Amount	Units	Peak Type	Peak Cod
1		1.565							671387	50.06	289347	bb			Unknown	
2		1.711							669661	49.94	256641	bb			Unknown	

SFC trace for (*S*)-**3a** and (±)-**3a**.

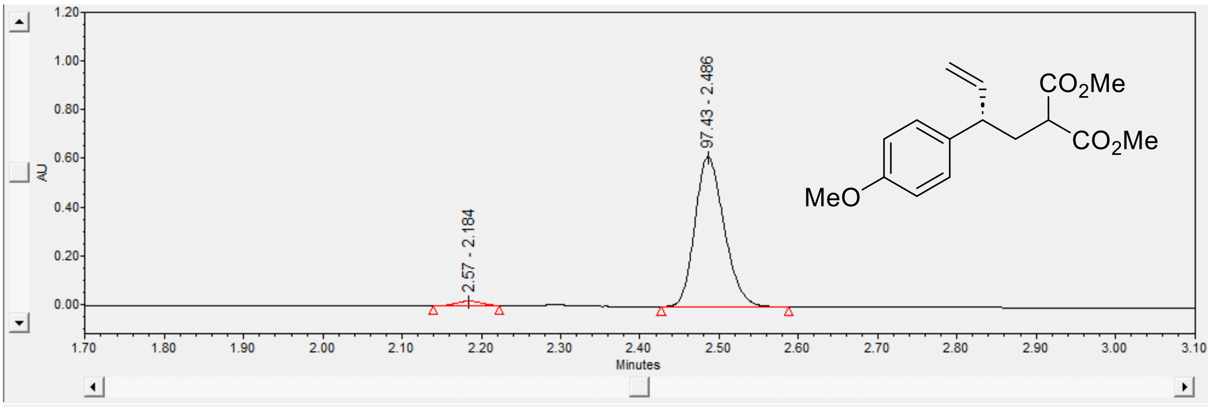

	Name	Retention Time (min)	Purity1 Angle	Purity1 Threshold	PDA/FLR Match1 Spect. Name	PDA/FLR Match1 Angle	PDA/FLR Match1 Threshold	PDA/FLR Match1 Lib. Name	Area (µV*sec)	% Area	Height (µV)	Int Type	Amount	Units	Peak Type	Peak Cod
1		2.184							41625	2.57	19210	bb			Unknown	
2		2.486							1576582	97.43	616240	bb			Unknown	

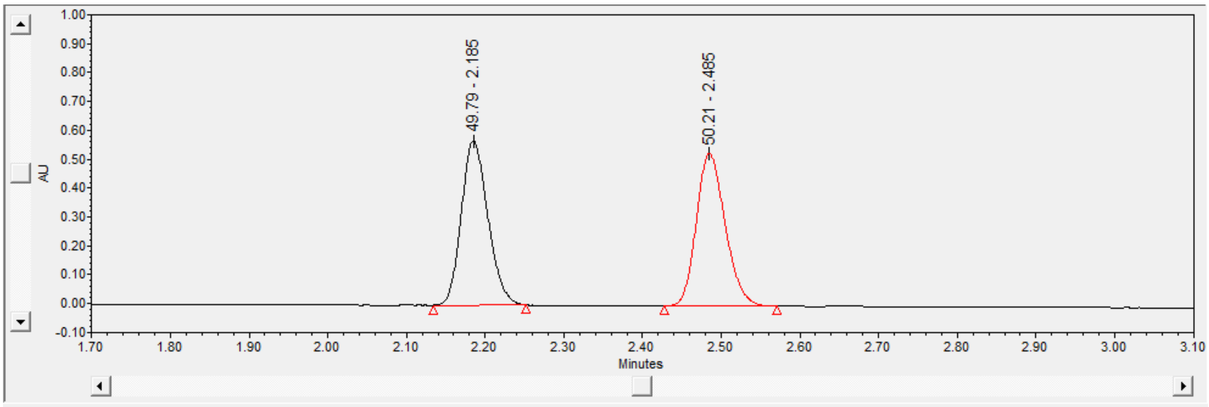

	Name	Retention Time (min)	Purity1 Angle	Purity1 Threshold	PDA/FLR Match1 Spect. Name	PDA/FLR Match1 Angle	PDA/FLR Match1 Threshold	PDA/FLR Match1 Lib. Name	Area (µV*sec)	% Area	Height (µV)	Int Type	Amount	Units	Peak Type	Peak C
1		2.185							1334912	49.79	568572	bb			Unknown	
2		2.485							1346420	50.21	528964	bb			Unknown	

SFC trace for (*S*)-**3b** and (±)-**3b**.

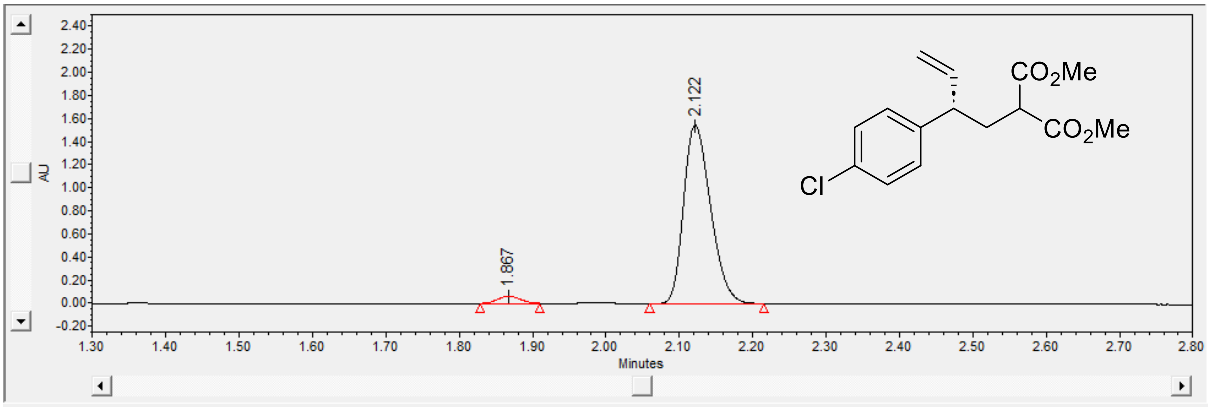

	Name	Retention Time (min)	Purity1 Angle	Purity1 Threshold	PDA/FLR Match1 Spect. Name	PDA/FLR Match1 Angle	PDA/FLR Match1 Threshold	PDA/FLR Match1 Lib. Name	Area (µV*sec)	% Area	Height (µV)	Int Type	Amount	Units	Peak Type	Peak
1		1.867							132114	3.24	62444	bb			Unknown	
2		2.122							3941671	96.76	1547403	bb			Unknown	

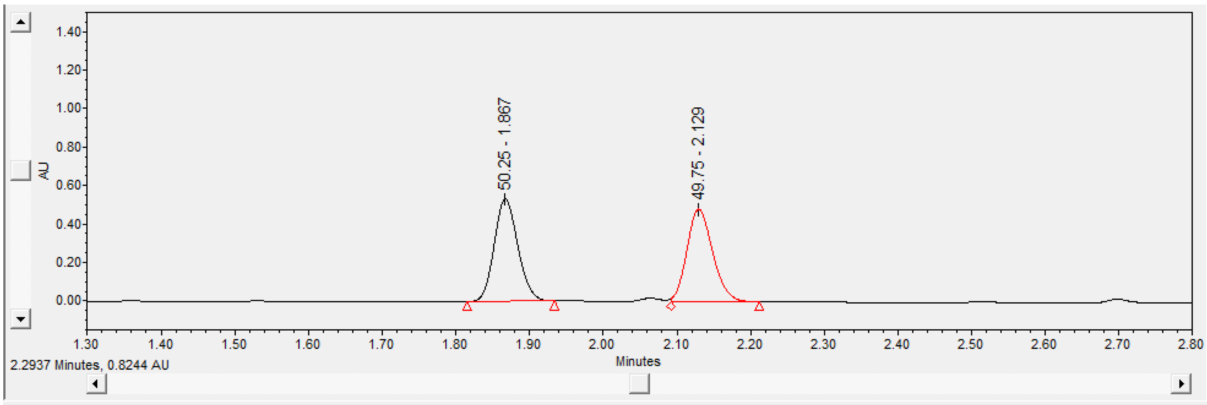

	Name	Retention Time (min)	Purity1 Angle	Purity1 Threshold	PDA/FLR Match1 Spect. Name	PDA/FLR Match1 Angle	PDA/FLR Match1 Threshold	PDA/FLR Match1 Lib. Name	Area (µV*sec)	% Area	Height (µV)	Int Type	Amount	Units	Peak Type	Peak (
1		1.867							1188985	50.25	533061	bb			Unknown	
2		2.129							1177136	49.75	482192	vb			Unknown	

SFC trace for (*S*)-**3c** and (±)-**3c**.

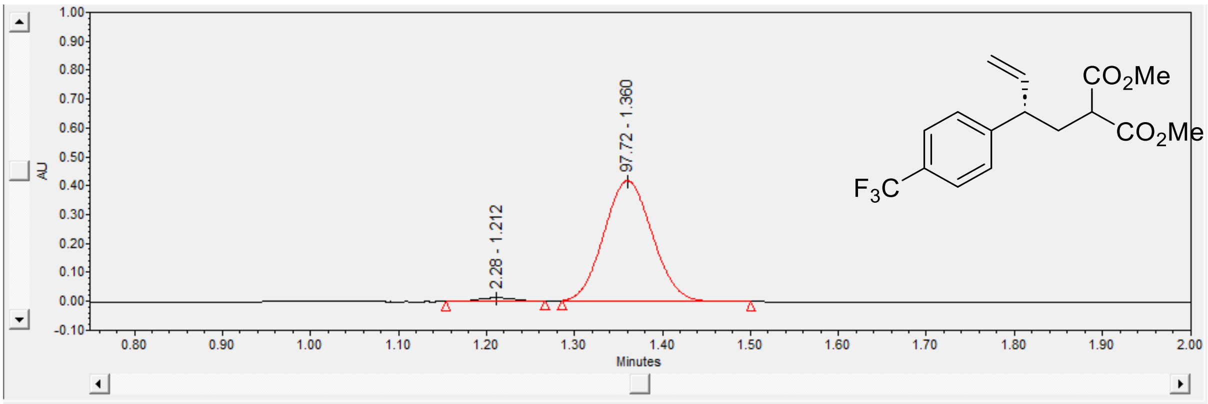

	Name	Retention Time (min)	Purity1 Angle	Purity1 Threshold	PDA/FLR Match1 Spect. Name	PDA/FLR Match1 Angle	PDA/FLR Match1 Threshold	PDA/FLR Match1 Lib. Name	Area (µV*sec)	% Area	Height (µV)	Int Type	Amount	Units	Peak Type	Peak C
1		1.212							36973	2.28	12791	bb			Unknown	
2		1.360							1586139	97.72	416382	bb			Unknown	

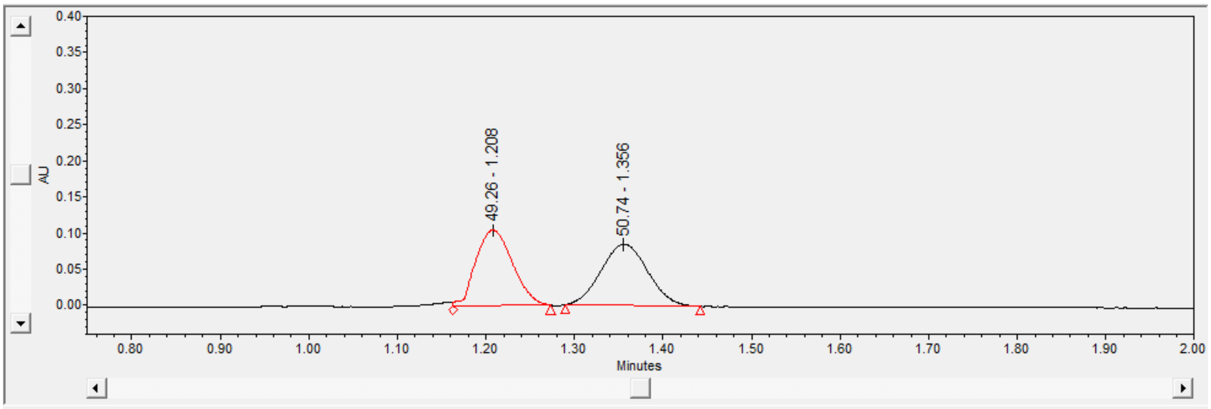

	Name	Retention Time (min)	Purity1 Angle	Purity1 Threshold	PDA/FLR Match1 Spect. Name	PDA/FLR Match1 Angle	PDA/FLR Match1 Threshold	PDA/FLR Match1 Lib. Name	Area (µV*sec)	% Area	Height (µV)	Int Type	Amount	Units	Peak Type	Peak (
1		1.208							301135	49.26	104734	vb			Unknown	
2		1.356							310216	50.74	84521	bb			Unknown	

SFC trace for (*S*)-**3d** and (±)-**3d**.

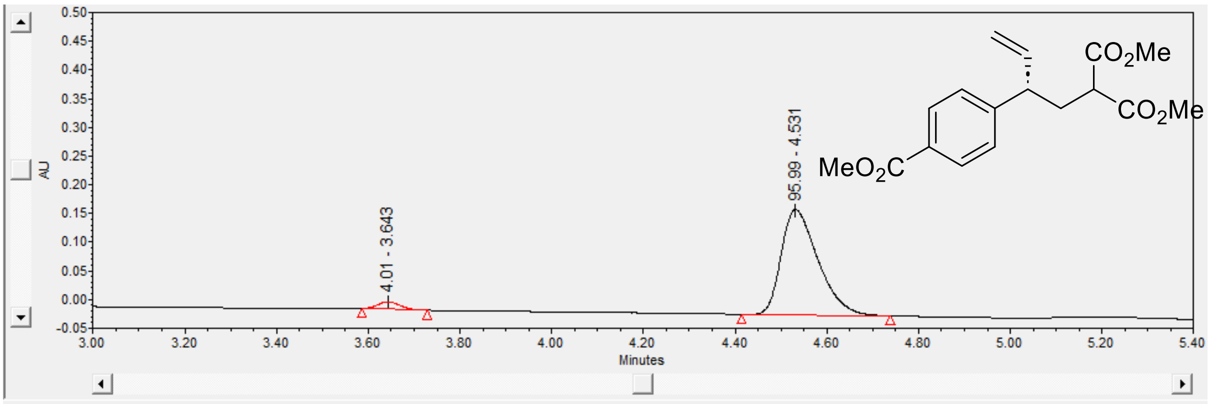

	Name	Retention Time (min)	Purity1 Angle	Purity1 Threshold	PDA/FLR Match1 Spect. Name	PDA/FLR Match1 Angle	PDA/FLR Match1 Threshold	PDA/FLR Match1 Lib. Name	Area (µV*sec)	% Area	Height (µV)	Int Type	Amount	Units	Peak Type	Peak
1		3.643							42705	4.01	12992	bb			Unknown	
2		4.531							1022476	95.99	183257	bb			Unknown	

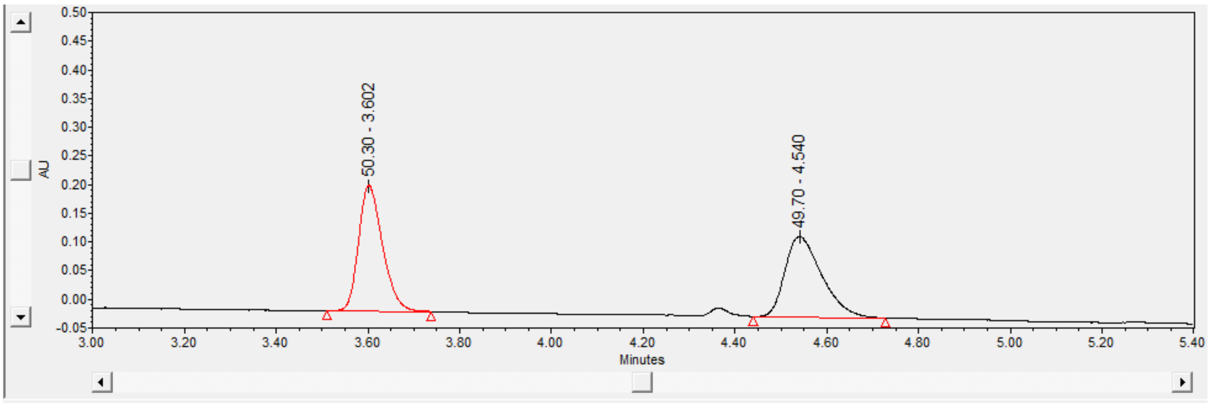

	Name	Retention Time (min)	Purity1 Angle	Purity1 Threshold	PDA/FLR Match1 Spect. Name	PDA/FLR Match1 Angle	PDA/FLR Match1 Threshold	PDA/FLR Match1 Lib. Name	Area (µV*sec)	% Area	Height (µV)	Int Type	Amount	Units	Peak Type	Peak
1		3.602							792842	50.30	218601	bb			Unknown	
2		4.540							783373	49.70	140446	bb			Unknown	

SFC trace for (*S*)-**3e** and (±)-**3e**.

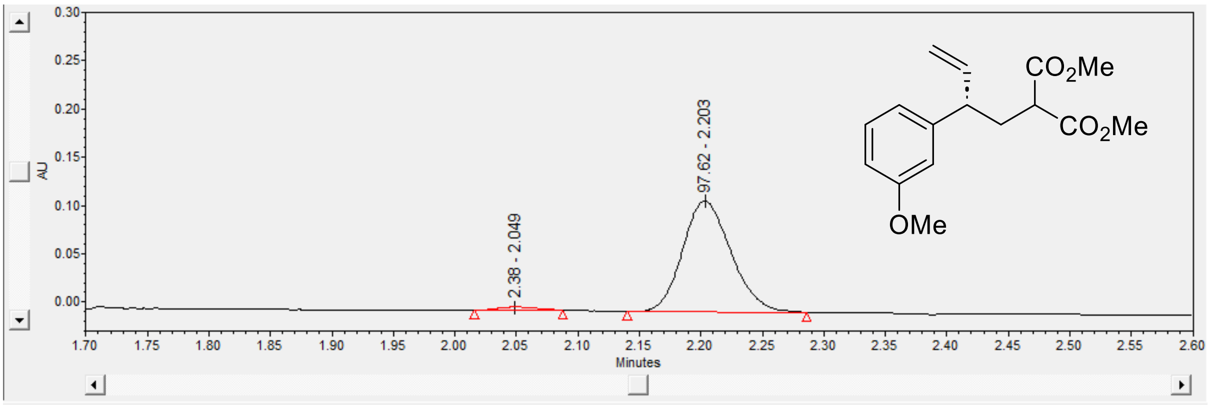

	Name	Retention Time (min)	Purity1 Angle	Purity1 Threshold	PDA/FLR Match1 Spect. Name	PDA/FLR Match1 Angle	PDA/FLR Match1 Threshold	PDA/FLR Match1 Lib. Name	Area (µV*sec)	% Area	Height (µV)	Int Type	Amount	Units	Peak Type	Peak C
1		2.049							7706	2.38	3572	bb			Unknown	
2		2.203							315861	97.62	114946	bb			Unknown	

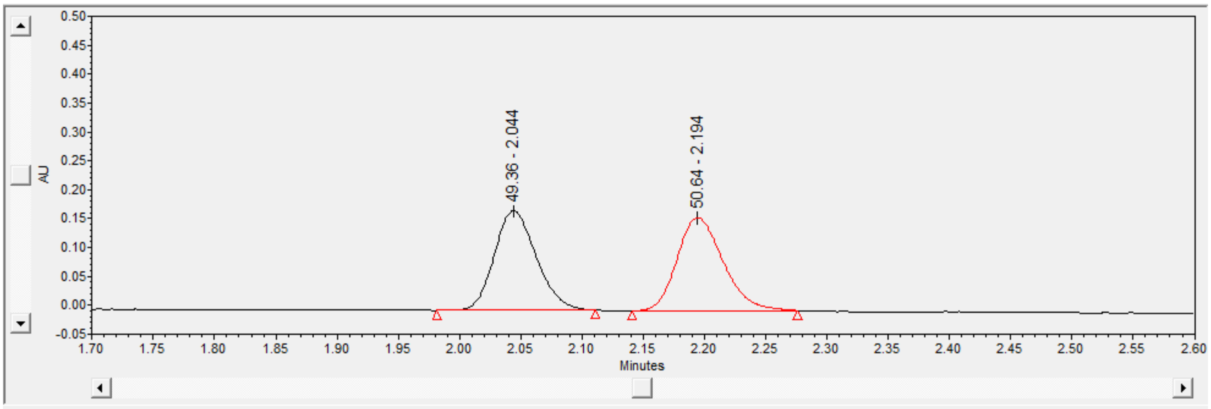

	Name	Retention Time (min)	Purity1 Angle	Purity1 Threshold	PDA/FLR Match1 Spect. Name	PDA/FLR Match1 Angle	PDA/FLR Match1 Threshold	PDA/FLR Match1 Lib. Name	Area (µV*sec)	% Area	Height (µV)	Int Type	Amount	Units	Peak Type	Peak C
1		2.044							409785	49.36	172318	bb			Unknown	
2		2.194							420479	50.64	161263	bb			Unknown	

SFC trace for (*S*)-**3f** and (±)-**3f**.

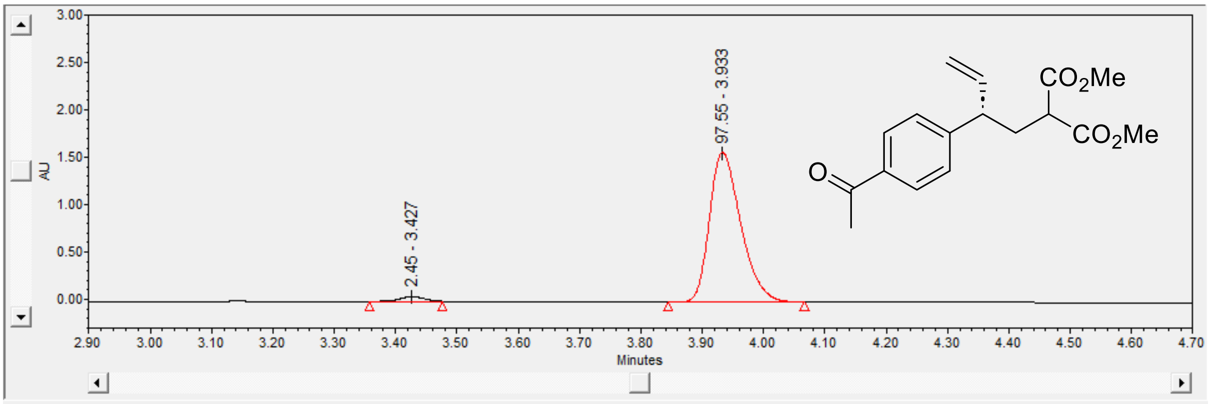

	Name	Retention Time (min)	Purity1 Angle	Purity1 Threshold	PDA/FLR Match1 Spect. Name	PDA/FLR Match1 Angle	PDA/FLR Match1 Threshold	PDA/FLR Match1 Lib. Name	Area (µV*sec)	% Area	Height (µV)	Int Type	Amount	Units	Peak Type	P
1		3.427							140767	2.45	52379	bb			Unknown	
2		3.933							5596648	97.55	1578959	bb			Unknown	

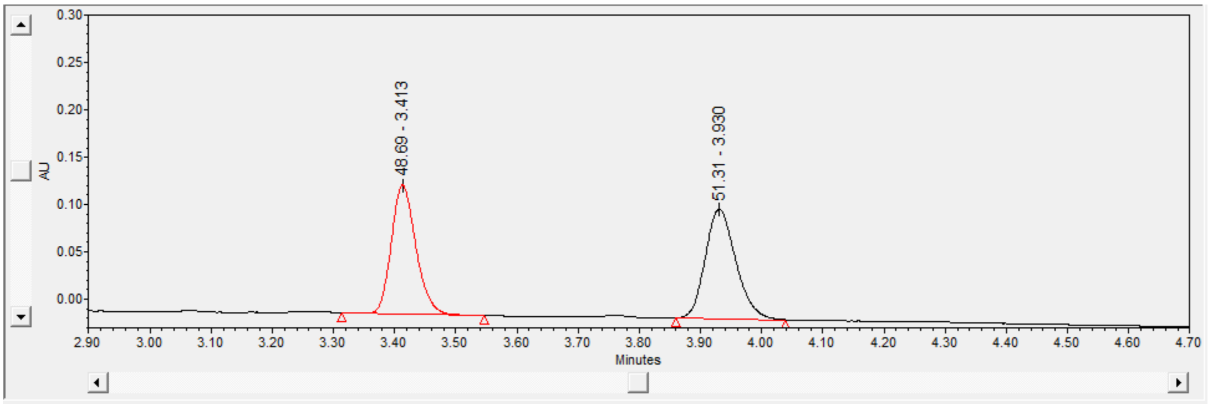

	Name	Retention Time (min)	Purity1 Angle	Purity1 Threshold	PDA/FLR Match1 Spect. Name	PDA/FLR Match1 Angle	PDA/FLR Match1 Threshold	PDA/FLR Match1 Lib. Name	Area (µV*sec)	% Area	Height (µV)	Int Type	Amount	Units	Peak Type	Pe
1		3.413							380284	48.69	136048	bb			Unknown	
2		3.930							400782	51.31	115642	bb			Unknown	

SFC trace for (*S*)-**3g** and (±)-**3g**.

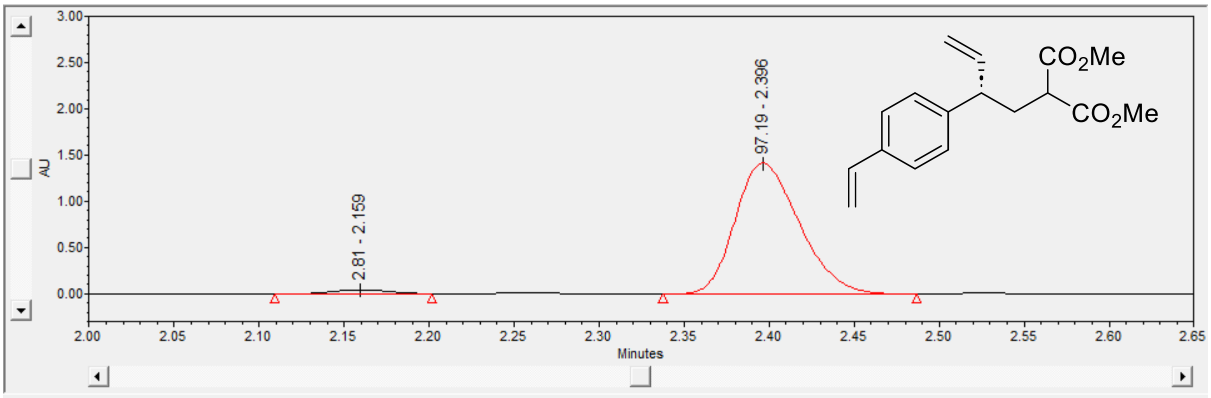

	Name	Retention Time (min)	Purity1 Angle	Purity1 Threshold	PDA/FLR Match1 Spect. Name	PDA/FLR Match1 Angle	PDA/FLR Match1 Threshold	PDA/FLR Match1 Lib. Name	Area (µV*sec)	% Area	Height (µV)	Int Type	Amount	Units	Peak Type	P
1		2.159							107919	2.81	46305	bb			Unknown	
2		2.396							3734914	97.19	1419175	bb			Unknown	

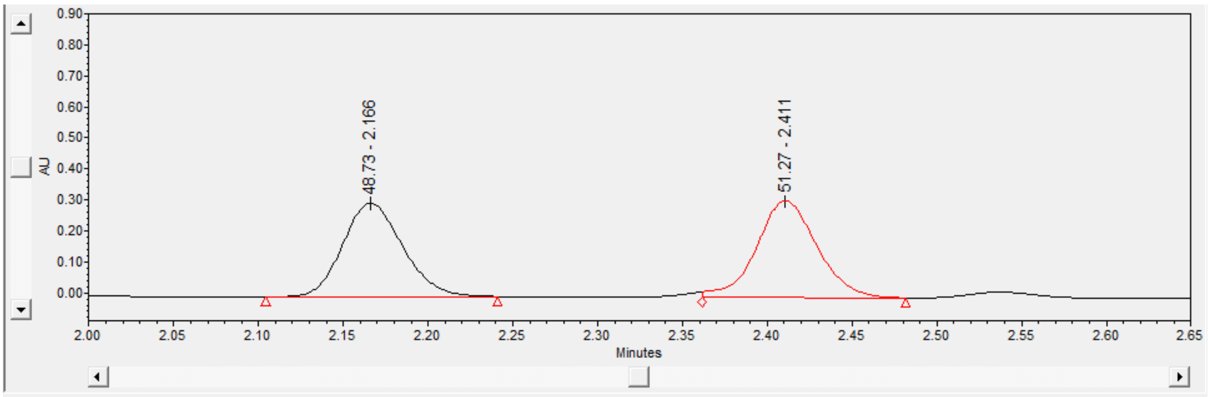

	Name	Retention Time (min)	Purity1 Angle	Purity1 Threshold	PDA/FLR Match1 Spect. Name	PDA/FLR Match1 Angle	PDA/FLR Match1 Threshold	PDA/FLR Match1 Lib. Name	Area (µV*sec)	% Area	Height (µV)	Int Type	Amount	Units	Peak Type	Pe
1		2.166							739001	48.73	301667	bb			Unknown	
2		2.411							777590	51.27	312615	vb			Unknown	

SFC trace for (*S*)-**3h** and (±)-**3h**.

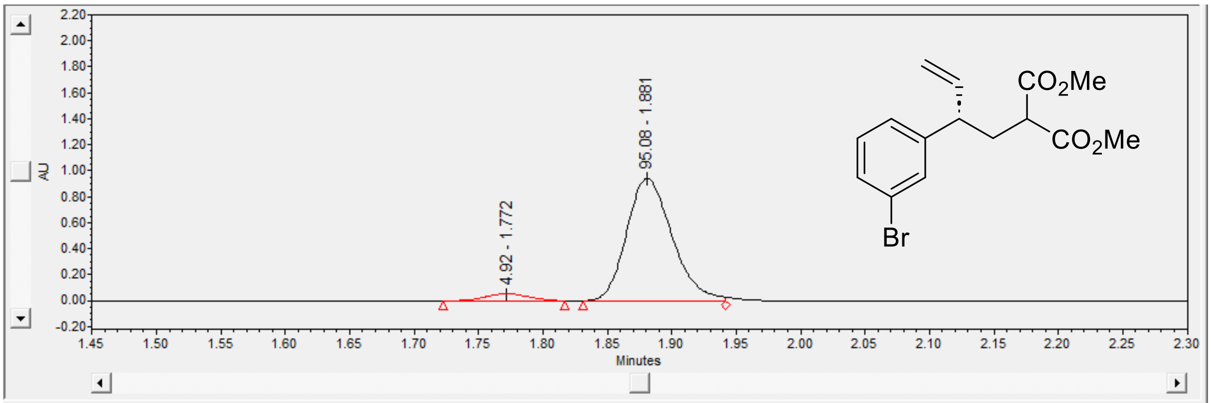

	Name	Retention Time (min)	Purity1 Angle	Purity1 Threshold	PDA/FLR Match1 Spect. Name	PDA/FLR Match1 Angle	PDA/FLR Match1 Threshold	PDA/FLR Match1 Lib. Name	Area (µV*sec)	% Area	Height (µV)	Int Type	Amount	Units	Peak Type	Peak
1		1.772							119765	4.92	53270	bb			Unknown	
2		1.881							2315071	95.08	945156	bv			Unknown	

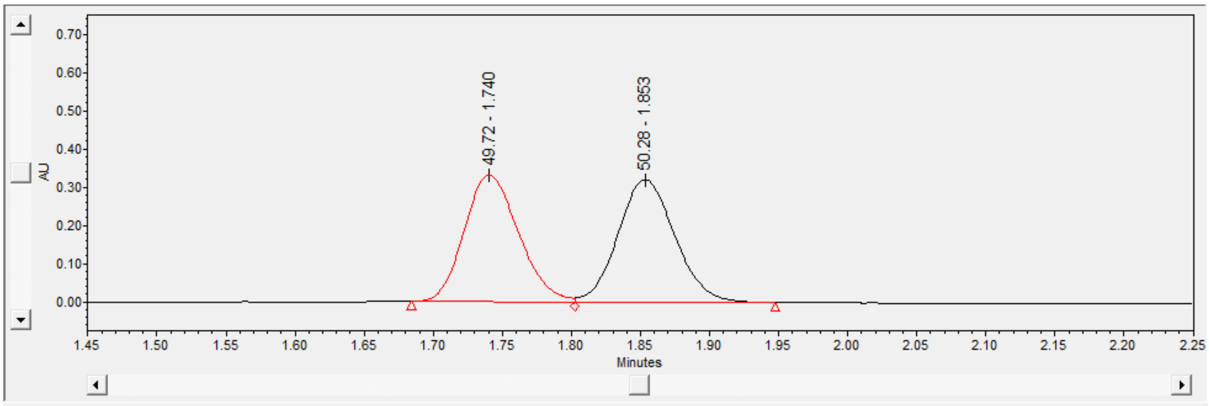

	Name	Retention Time (min)	Purity1 Angle	Purity1 Threshold	PDA/FLR Match1 Spect. Name	PDA/FLR Match1 Angle	PDA/FLR Match1 Threshold	PDA/FLR Match1 Lib. Name	Area (µV*sec)	% Area	Height (µV)	Int Type	Amount	Units	Peak Type	Peak
1		1.740							905241	49.72	331418	bv			Unknown	
2		1.853							915388	50.28	319984	vb			Unknown	

SFC trace for (*S*)-**3i** and (±)-**3i**.

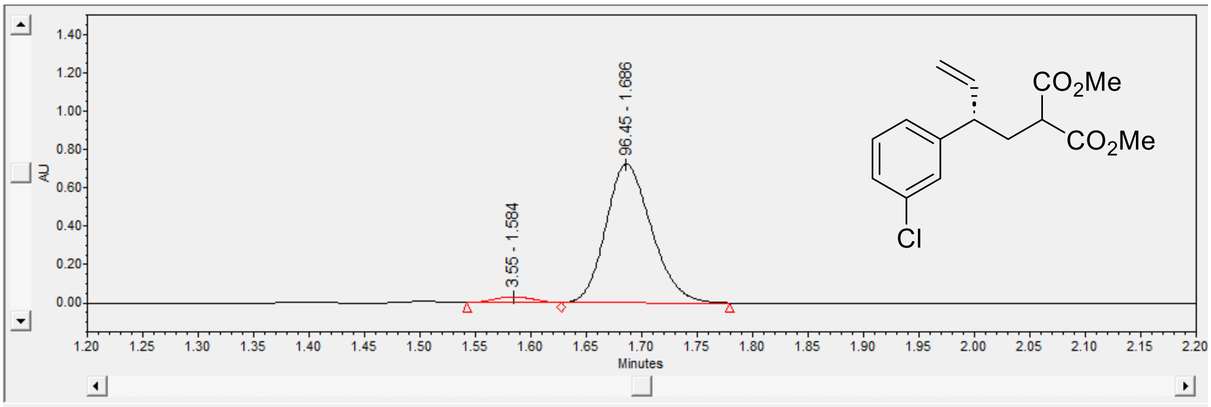

	Name	Retention Time (min)	Purity1 Angle	Purity1 Threshold	PDA/FLR Match1 Spect. Name	PDA/FLR Match1 Angle	PDA/FLR Match1 Threshold	PDA/FLR Match1 Lib. Name	Area (µV*sec)	% Area	Height (µV)	Int Type	Amount	Units	Peak Type	Peak
1		1.584							76588	3.55	31092	bv			Unknown	
2		1.686							2079923	96.45	722479	vb			Unknown	

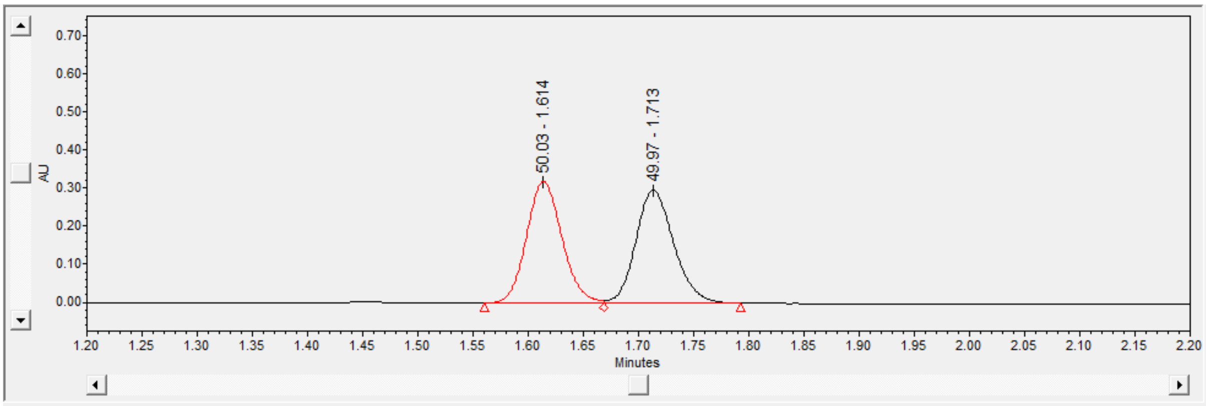

	Name	Retention Time (min)	Purity1 Angle	Purity1 Threshold	PDA/FLR Match1 Spect. Name	PDA/FLR Match1 Angle	PDA/FLR Match1 Threshold	PDA/FLR Match1 Lib. Name	Area (µV*sec)	% Area	Height (µV)	Int Type	Amount	Units	Peak Type	Peak
1		1.614							706740	50.03	317746	bv			Unknown	
2		1.713							706011	49.97	295404	vb			Unknown	

SFC trace for (*S*)-**3j** and (±)-**3j**.

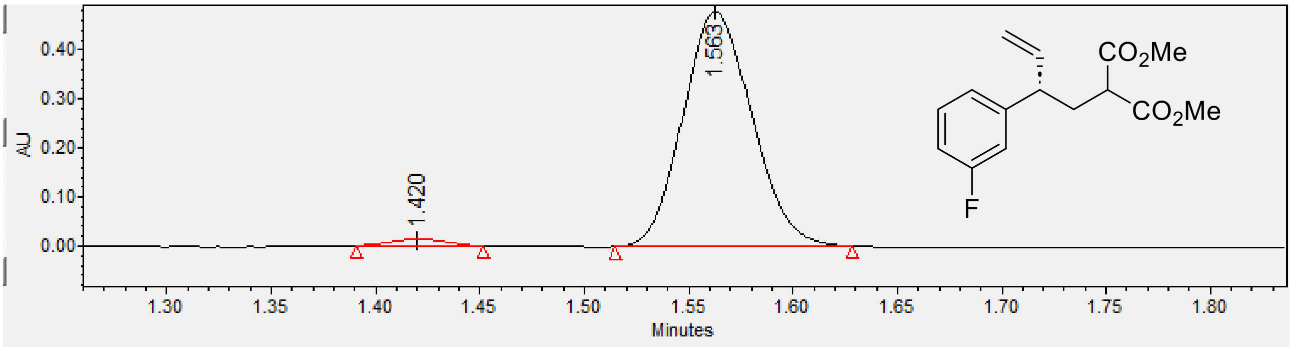

	Name	Retention Time (min)	Purity1 Angle	Purity1 Threshold	PDA/FLR Match1 Spect. Name	PDA/FLR Match1 Angle	PDA/FLR Match1 Threshold	PDA/FLR Match1 Lib. Name	Area (µV*sec)	% Area	Height (µV)	Int Type	Amount	Units	Peak Type	Pea
1		1.420							25783	2.26	13406	bb			Unknown	
2		1.563							1113459	97.74	480664	bb			Unknown	

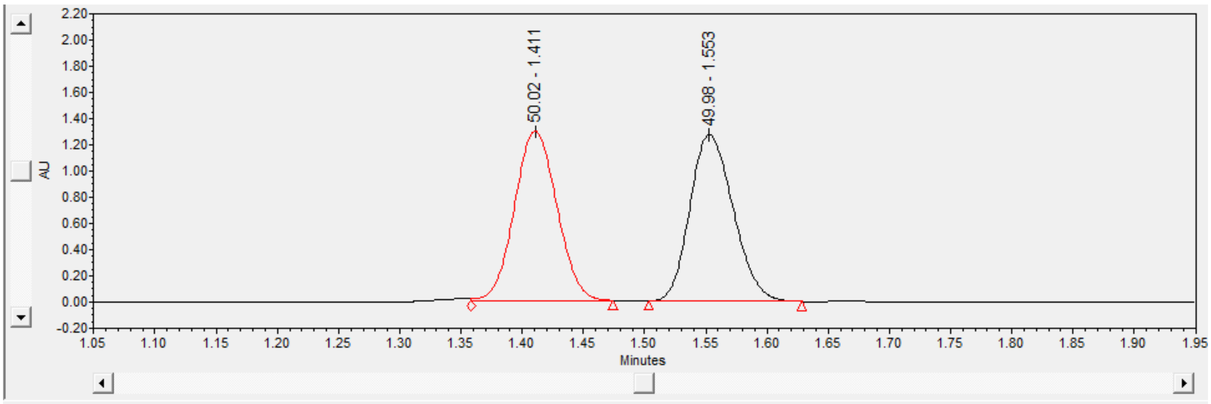

	Name	Retention Time (min)	Purity1 Angle	Purity1 Threshold	PDA/FLR Match1 Spect. Name	PDA/FLR Match1 Angle	PDA/FLR Match1 Threshold	PDA/FLR Match1 Lib. Name	Area (µV*sec)	% Area	Height (µV)	Int Type	Amount	Units	Peak Type	Pe
1		1.411							3136784	50.02	1296572	vb			Unknown	
2		1.553							3134343	49.98	1273089	bb			Unknown	

SFC trace for (*S*)-**3k** and (±)-**3k**.

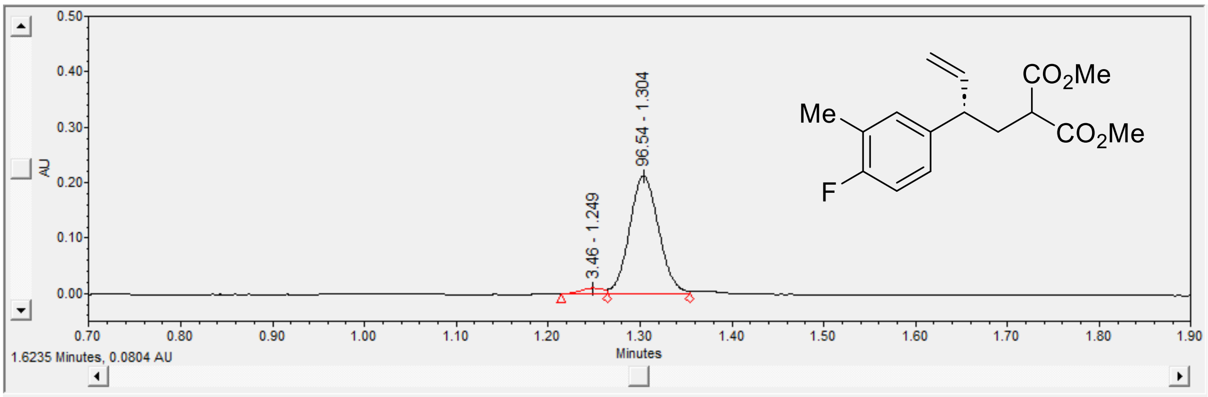

	Name	Retention Time (min)	Purity1 Angle	Purity1 Threshold	PDA/FLR Match1 Spect. Name	PDA/FLR Match1 Angle	PDA/FLR Match1 Threshold	PDA/FLR Match1 Lib. Name	Area (µV*sec)	% Area	Height (µV)	Int Type	Amount	Units	Peak Type	Pe
1		1.249							16920	3.46	10092	bv			Unknown	
2		1.304							472071	96.54	213225	vv			Unknown	

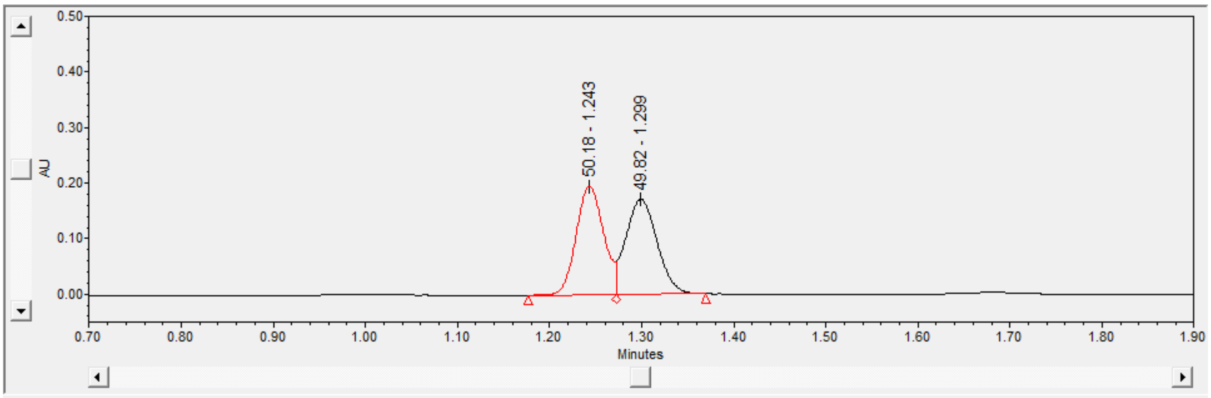

	Name	Retention Time (min)	Purity1 Angle	Purity1 Threshold	PDA/FLR Match1 Spect. Name	PDA/FLR Match1 Angle	PDA/FLR Match1 Threshold	PDA/FLR Match1 Lib. Name	Area (µV*sec)	% Area	Height (µV)	Int Type	Amount	Units	Peak Type	Pe
1		1.243							407060	50.18	195388	bv			Unknown	
2		1.299							404110	49.82	171476	vb			Unknown	

SFC trace for (*S*)-**3l** and (±)-**3l**.

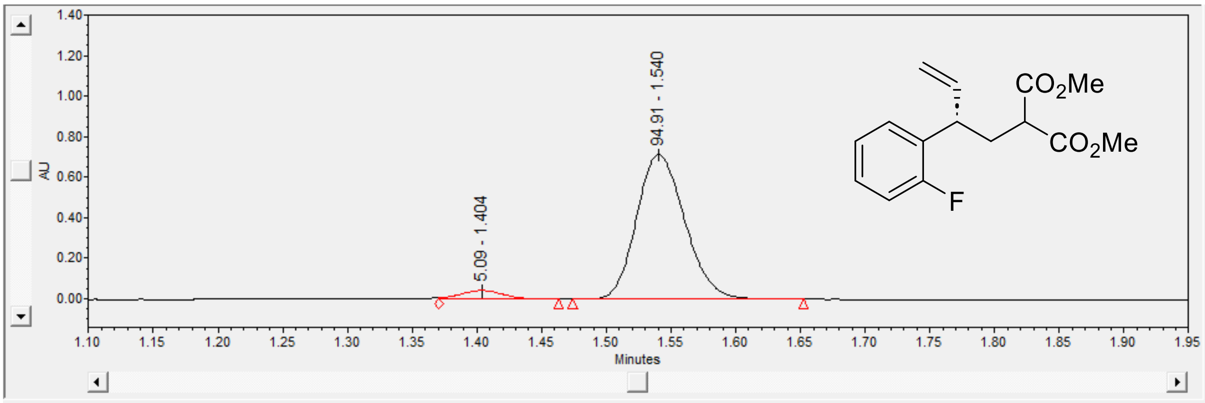

	Name	Retention Time (min)	Purity1 Angle	Purity1 Threshold	PDA/FLR Match1 Spect. Name	PDA/FLR Match1 Angle	PDA/FLR Match1 Threshold	PDA/FLR Match1 Lib. Name	Area (µV*sec)	% Area	Height (µV)	Int Type	Amount	Units	Peak Type	Pe
1		1.404							99486	5.09	42837	vb			Unknown	
2		1.540							1853555	94.91	715399	bb			Unknown	

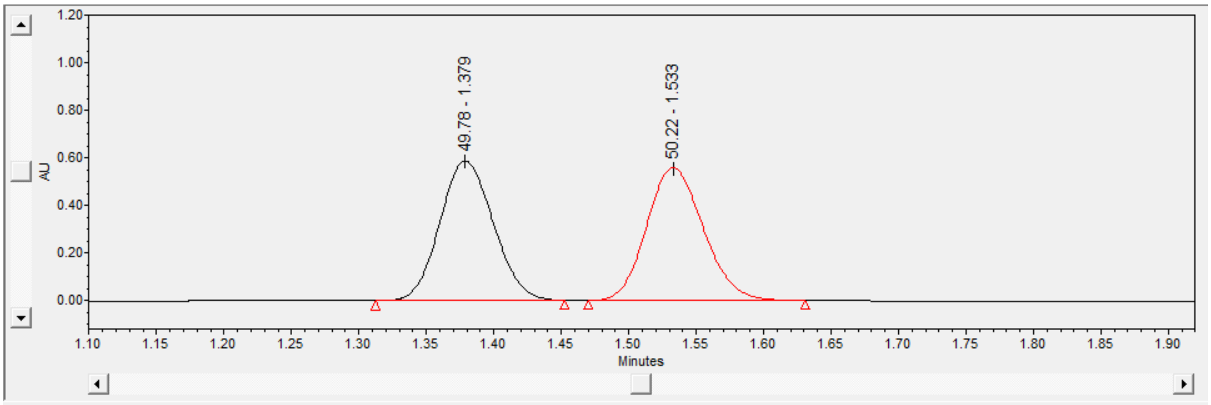

	Name	Retention Time (min)	Purity1 Angle	Purity1 Threshold	PDA/FLR Match1 Spect. Name	PDA/FLR Match1 Angle	PDA/FLR Match1 Threshold	PDA/FLR Match1 Lib. Name	Area (µV*sec)	% Area	Height (µV)	Int Type	Amount	Units	Peak Type	Pe
1		1.379							1621823	49.78	587844	bb			Unknown	
2		1.533							1636347	50.22	558323	bb			Unknown	

SFC trace for (*S*)-**3m** and (±)-**3m**.

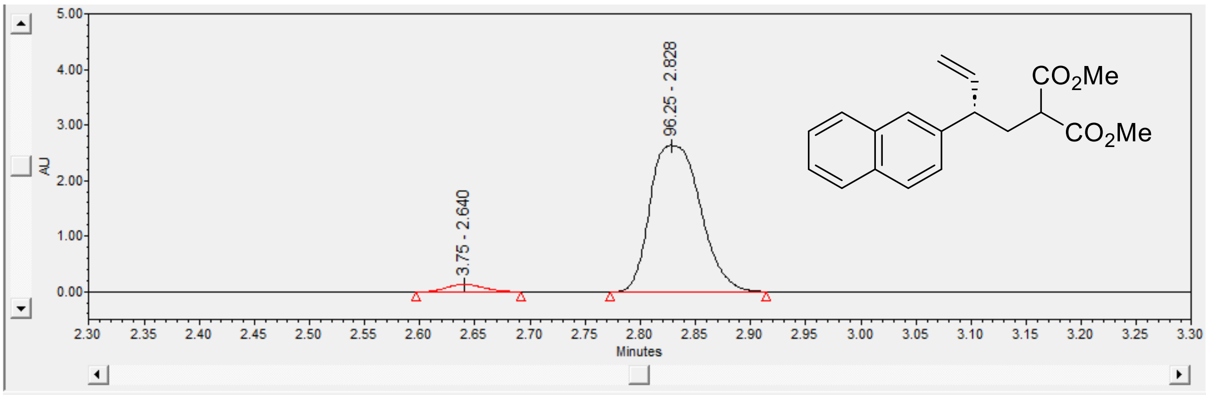

	Name	Retention Time (min)	Purity1 Angle	Purity1 Threshold	PDA/FLR Match1 Spect. Name	PDA/FLR Match1 Angle	PDA/FLR Match1 Threshold	PDA/FLR Match1 Lib. Name	Area (µV*sec)	% Area	Height (µV)	Int Type	Amount	Units	Peak Type	P
1		2.640							326845	3.75	138960	bb			Unknown	
2		2.828							8400158	96.25	2638813	bb			Unknown	

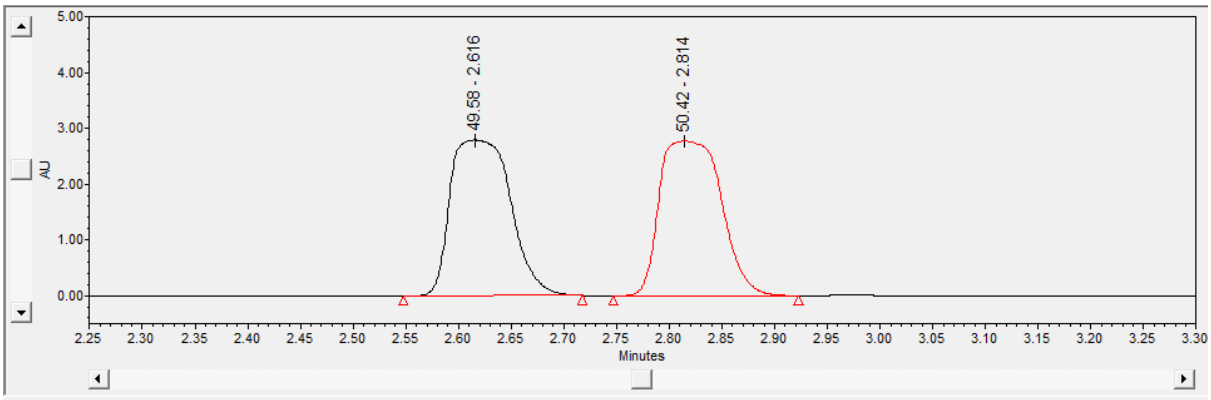

	Name	Retention Time (min)	Purity1 Angle	Purity1 Threshold	PDA/FLR Match1 Spect. Name	PDA/FLR Match1 Angle	PDA/FLR Match1 Threshold	PDA/FLR Match1 Lib. Name	Area (µV*sec)	% Area	Height (µV)	Int Type	Amount	Units	Peak Type
1		2.616							11102961	49.58	2794664	bb			Unknown
2		2.814							11293131	50.42	2779573	bb			Unknown

SFC trace for (*S*)-**3n** and (±)-**3n**.

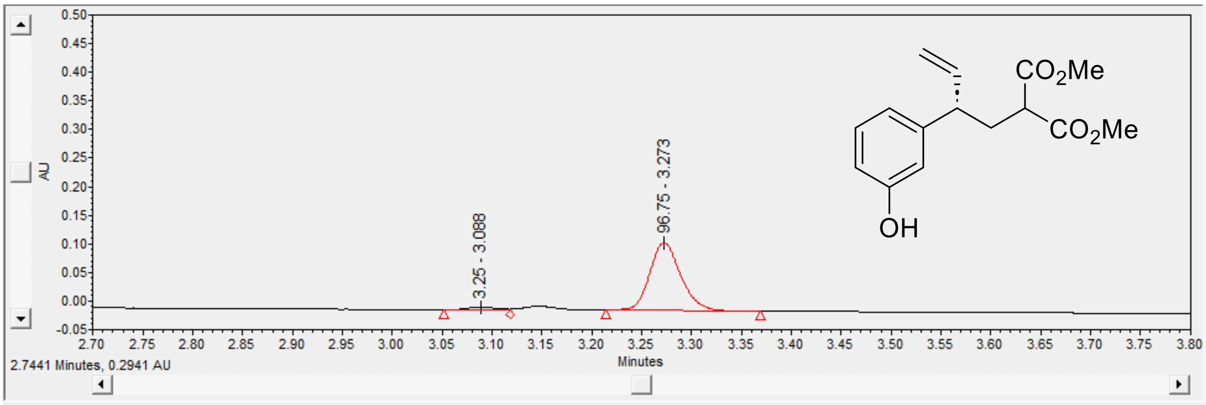

	Name	Retention Time (min)	Purity1 Angle	Purity1 Threshold	PDA/FLR Match1 Spect. Name	PDA/FLR Match1 Angle	PDA/FLR Match1 Threshold	PDA/FLR Match1 Lib. Name	Area (µV*sec)	% Area	Height (µV)	Int Type	Amount	Units	Peak Type	Pea
1		3.088							8545	3.25	4644	bv			Unknown	
2		3.273							254402	96.75	118411	bb			Unknown	

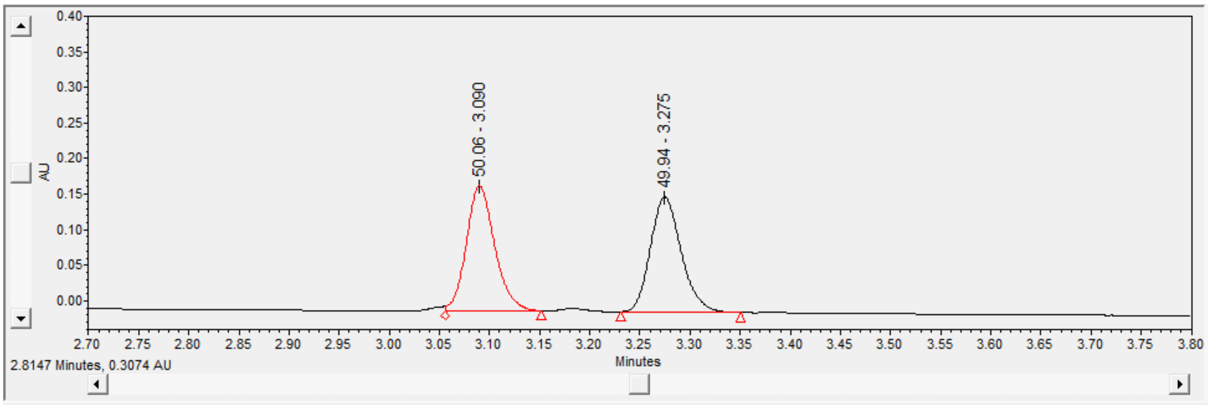

	Name	Retention Time (min)	Purity1 Angle	Purity1 Threshold	PDA/FLR Match1 Spect. Name	PDA/FLR Match1 Angle	PDA/FLR Match1 Threshold	PDA/FLR Match1 Lib. Name	Area (µV*sec)	% Area	Height (µV)	Int Type	Amount	Units	Peak Type	Pea
1		3.090							347071	50.06	175854	vb			Unknown	
2		3.275							346198	49.94	161904	bb			Unknown	

SFC trace for (*S*)-**3o** and (±)-**3o**.

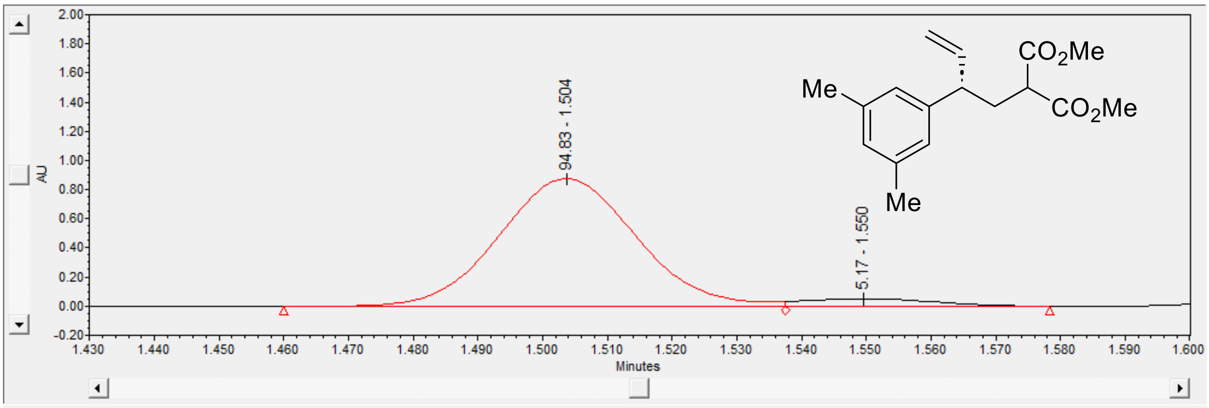

	Name	Retention Time (min)	Purity1 Angle	Purity1 Threshold	PDA/FLR Match1 Spect. Name	PDA/FLR Match1 Angle	PDA/FLR Match1 Threshold	PDA/FLR Match1 Lib. Name	Area (µV*sec)	% Area	Height (µV)	Int Type	Amount	Units	Peak Type	Peak Cod
1		1.504							1292610	94.83	877158	bv			Unknown	
2		1.550							70420	5.17	52406	vb			Unknown	

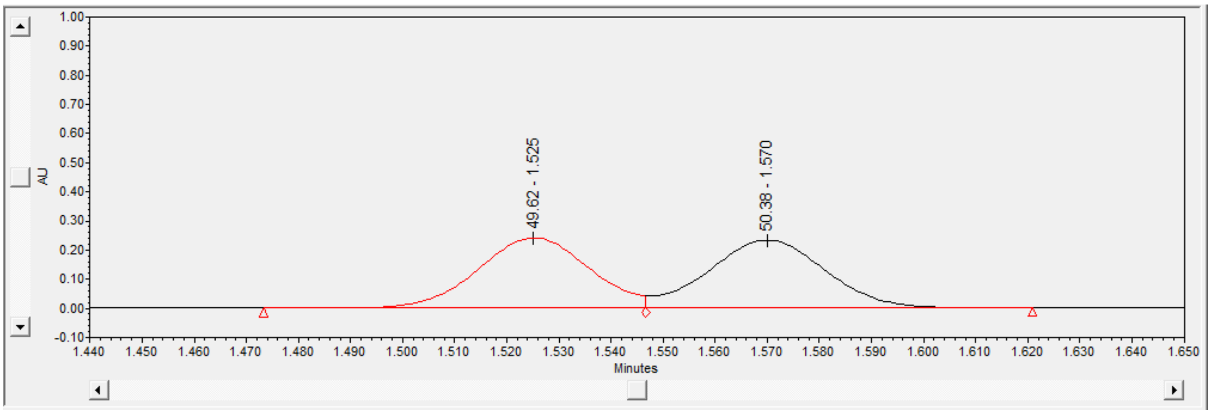

	Name	Retention Time (min)	Purity1 Angle	Purity1 Threshold	PDA/FLR Match1 Spect. Name	PDA/FLR Match1 Angle	PDA/FLR Match1 Threshold	PDA/FLR Match1 Lib. Name	Area (µV*sec)	% Area	Height (µV)	Int Type	Amount	Units	Peak Type	Peak Code
1		1.525							355759	49.62	240967	bv			Unknown	
2		1.570							361279	50.38	232664	vb			Unknown	

SFC trace for (*S*)-**3p** and (±)-**3p**.

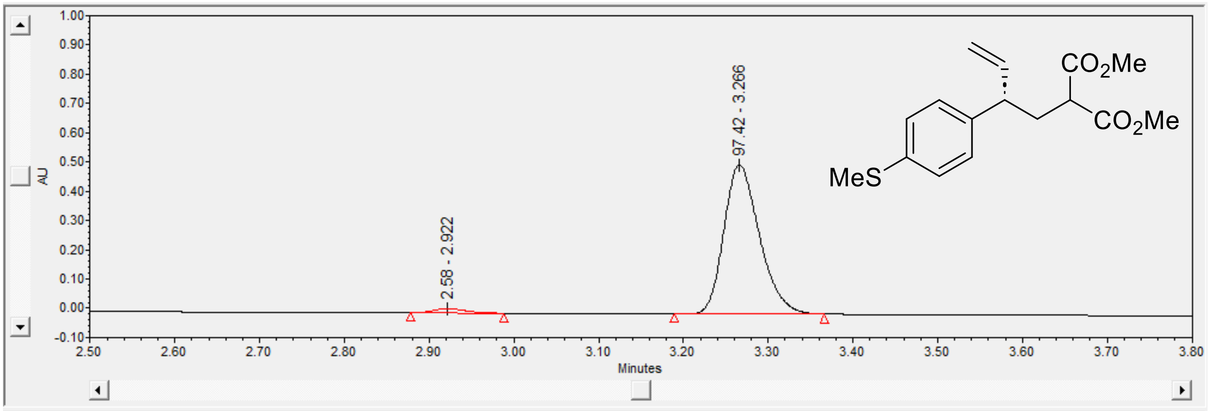

	Name	Retention Time (min)	Purity1 Angle	Purity1 Threshold	PDA/FLR Match1 Spect. Name	PDA/FLR Match1 Angle	PDA/FLR Match1 Threshold	PDA/FLR Match1 Lib. Name	Area (µV*sec)	% Area	Height (µV)	Int Type	Amount	Units	Peak Type	Peak Code
1		2.922							39433	2.58	15304	bb			Unknown	
2		3.266							1487813	97.42	510426	bb			Unknown	

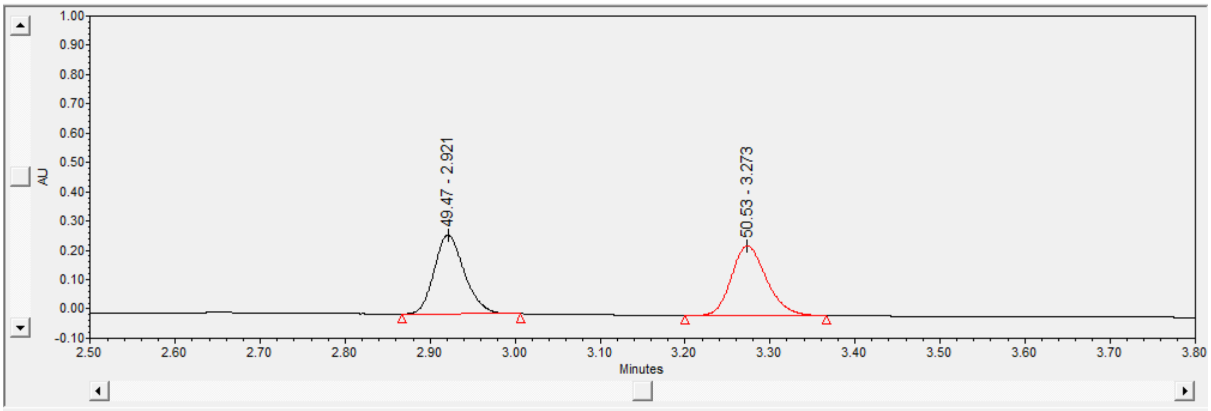

	Name	Retention Time (min)	Purity1 Angle	Purity1 Threshold	PDA/FLR Match1 Spect. Name	PDA/FLR Match1 Angle	PDA/FLR Match1 Threshold	PDA/FLR Match1 Lib. Name	Area (µV*sec)	% Area	Height (µV)	Int Type	Amount	Units	Peak Type	Peak Code
1		2.921							663805	49.47	269451	bb			Unknown	
2		3.273							677978	50.53	238026	bb			Unknown	

SFC trace for (*S*)-**3q** and (±)-**3q**.

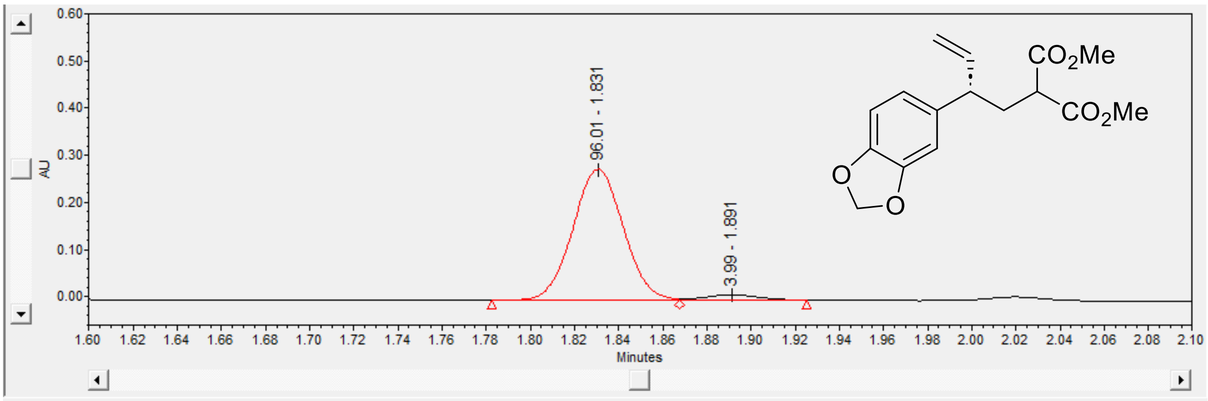

	Name	Retention Time (min)	Purity1 Angle	Purity1 Threshold	PDA/FLR Match1 Spect. Name	PDA/FLR Match1 Angle	PDA/FLR Match1 Threshold	PDA/FLR Match1 Lib. Name	Area (µV*sec)	% Area	Height (µV)	Int Type	Amount	Units	Peak Type	Pe
1		1.831							441202	96.01	277410	bv			Unknown	
2		1.891							18336	3.99	11158	vb			Unknown	

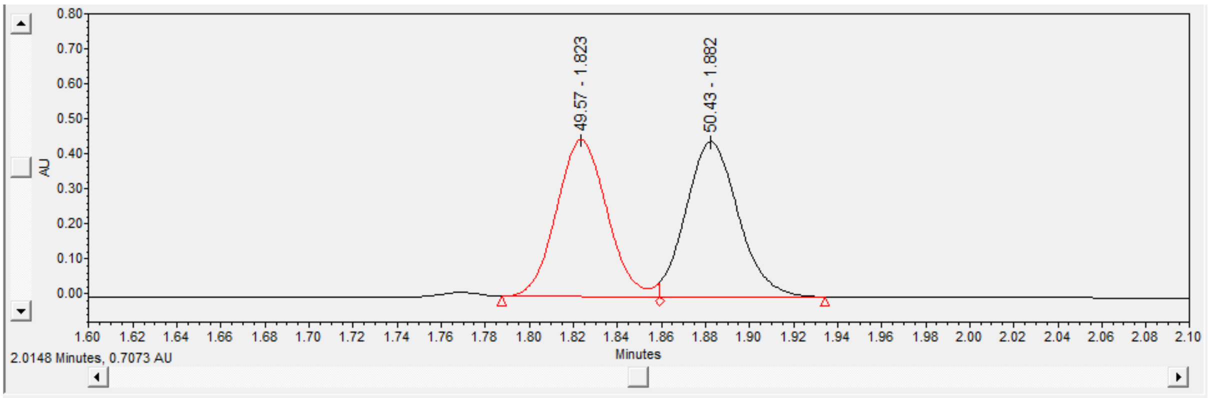

	Name	Retention Time (min)	Purity1 Angle	Purity1 Threshold	PDA/FLR Match1 Spect. Name	PDA/FLR Match1 Angle	PDA/FLR Match1 Threshold	PDA/FLR Match1 Lib. Name	Area (µV*sec)	% Area	Height (µV)	Int Type	Amount	Units	Peak Type	Pe
1		1.823							715389	49.57	447223	bv			Unknown	
2		1.882							727739	50.43	443029	vb			Unknown	

SFC trace for (*S*)-**3t** and (±)-**3t**.

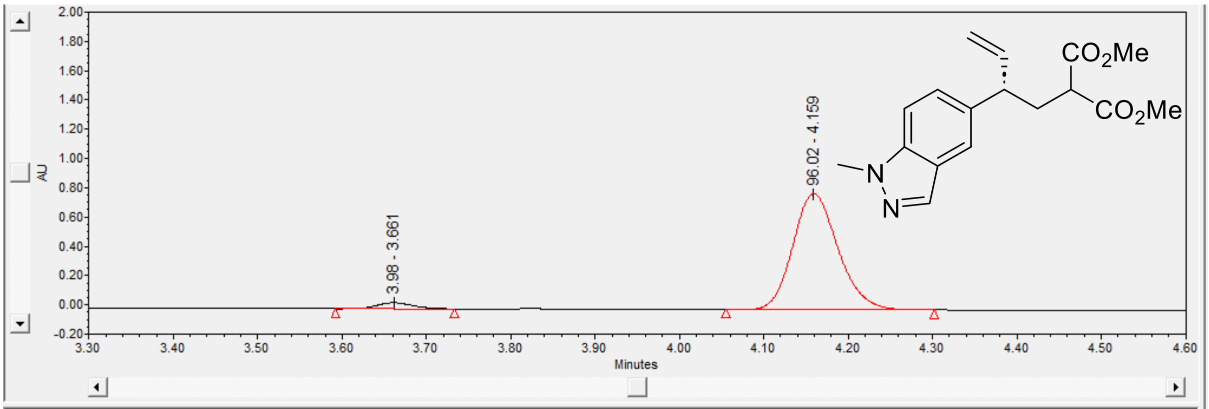

	Name	Retention Time (min)	Purity1 Angle	Purity1 Threshold	PDA/FLR Match1 Spect. Name	PDA/FLR Match1 Angle	PDA/FLR Match1 Threshold	PDA/FLR Match1 Lib. Name	Area (µV*sec)	% Area	Height (µV)	Int Type	Amount	Units	Peak Type	Peak Codes
1		3.661							122380	3.98	41340	bb			Unknown	
2		4.159							2949122	96.02	792780	bb			Unknown	

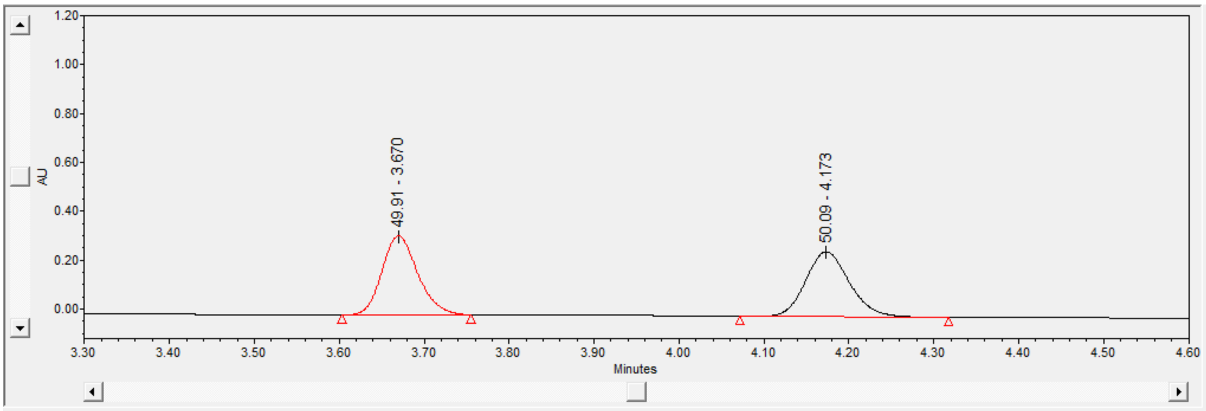

	Name	Retention Time (min)	Purity1 Angle	Purity1 Threshold	PDA/FLR Match1 Spect. Name	PDA/FLR Match1 Angle	PDA/FLR Match1 Threshold	PDA/FLR Match1 Lib. Name	Area (µV*sec)	% Area	Height (µV)	Int Type	Amount	Units	Peak Type	Peak Codes
1		3.670							945749	49.91	323022	bb			Unknown	
2		4.173							949159	50.09	265547	bb			Unknown	

SFC trace for (*S*)-**3u** and (±)-**3u**.

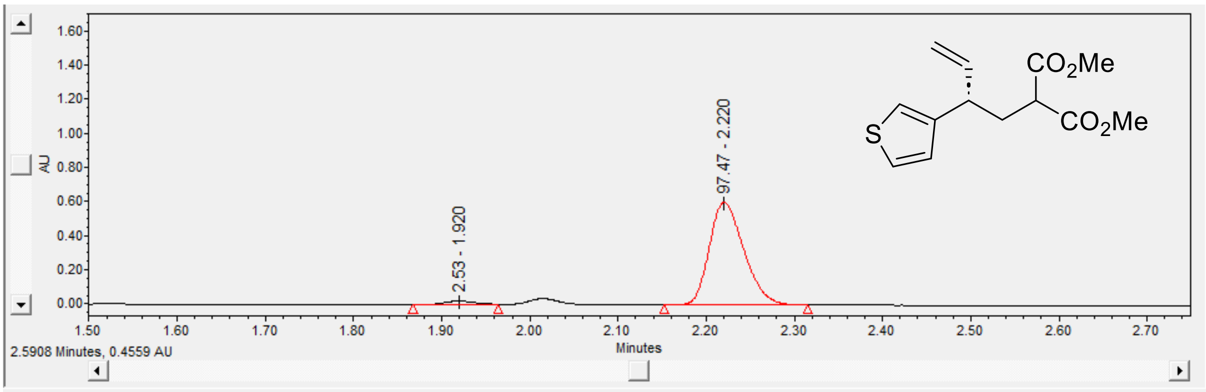

	Name	Retention Time (min)	Purity1 Angle	Purity1 Threshold	PDA/FLR Match1 Spect. Name	PDA/FLR Match1 Angle	PDA/FLR Match1 Threshold	PDA/FLR Match1 Lib. Name	Area (µV*sec)	% Area	Height (µV)	Int Type	Amount	Units	Peak Type	Pe
1		1.920							42392	2.53	19244	bb			Unknown	
2		2.220							1635276	97.47	600193	bb			Unknown	

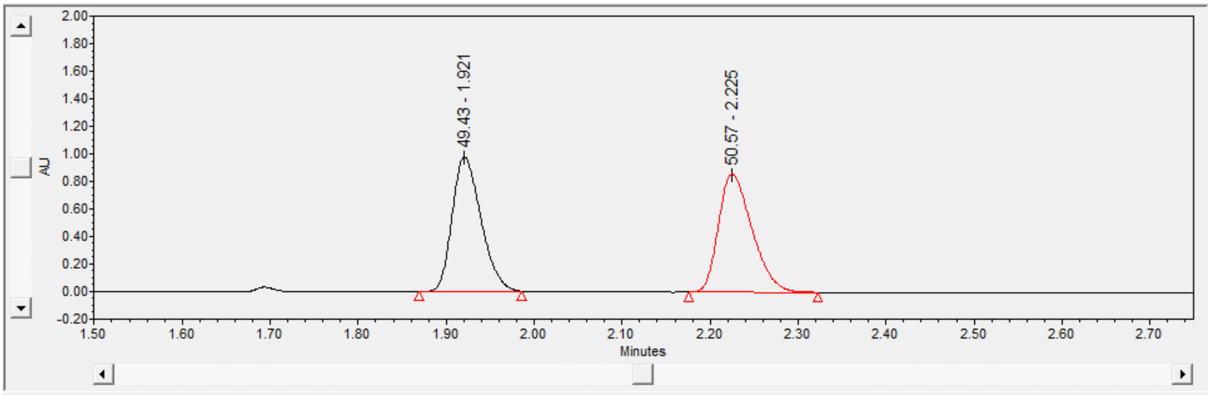

	Name	Retention Time (min)	Purity1 Angle	Purity1 Threshold	PDA/FLR Match1 Spect. Name	PDA/FLR Match1 Angle	PDA/FLR Match1 Threshold	PDA/FLR Match1 Lib. Name	Area (µV*sec)	% Area	Height (µV)	Int Type	Amount	Units	Peak Type	Pe
1		1.921							2223776	49.43	975300	bb			Unknown	
2		2.225							2274900	50.57	858106	bb			Unknown	

SFC trace for (*S*)-**3v** and (±)-**3v**.

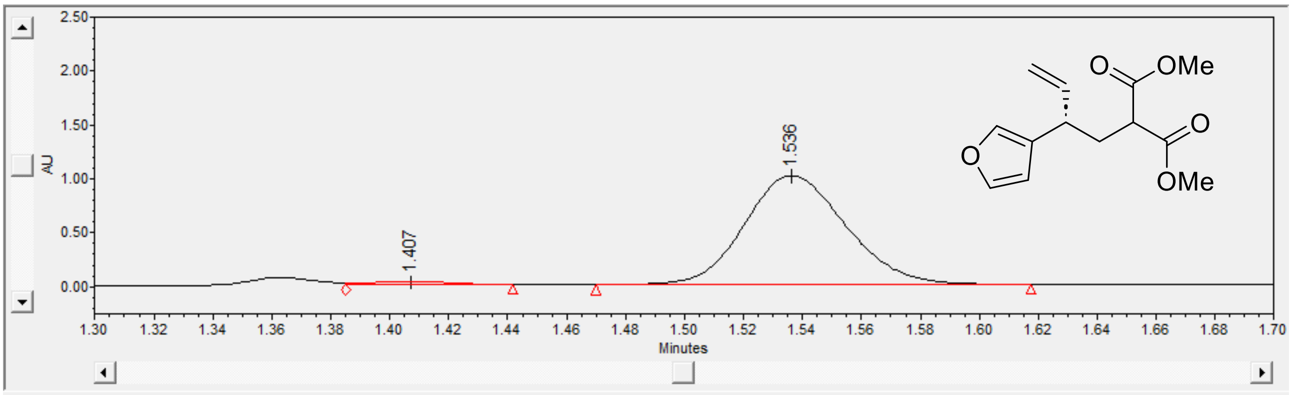

	Name	Retention Time (min)	Purity1 Angle	Purity1 Threshold	PDA/FLR Match1 Spect. Name	PDA/FLR Match1 Angle	PDA/FLR Match1 Threshold	PDA/FLR Match1 Lib. Name	Area (µV*sec)	% Area	Height (µV)	Int Type	Amount	Units	Peak Type	P
1		1.407							64108	2.58	30280	vb			Unknown	
2		1.536							2418082	97.42	1009893	bb			Unknown	

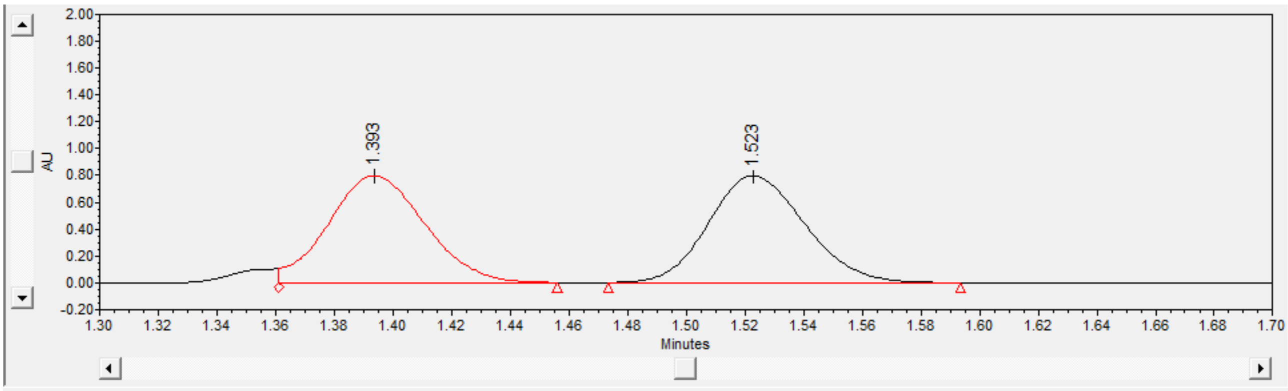

	Name	Retention Time (min)	Purity1 Angle	Purity1 Threshold	PDA/FLR Match1 Spect. Name	PDA/FLR Match1 Angle	PDA/FLR Match1 Threshold	PDA/FLR Match1 Lib. Name	Area (µV*sec)	% Area	Height (µV)	Int Type	Amount	Units	Peak Type	Pe
1		1.393							1816164	50.10	797392	vb			Unknown	
2		1.523							1809241	49.90	794575	bb			Unknown	

SFC trace for (*S*)-**3w** and (±)-**3w**.

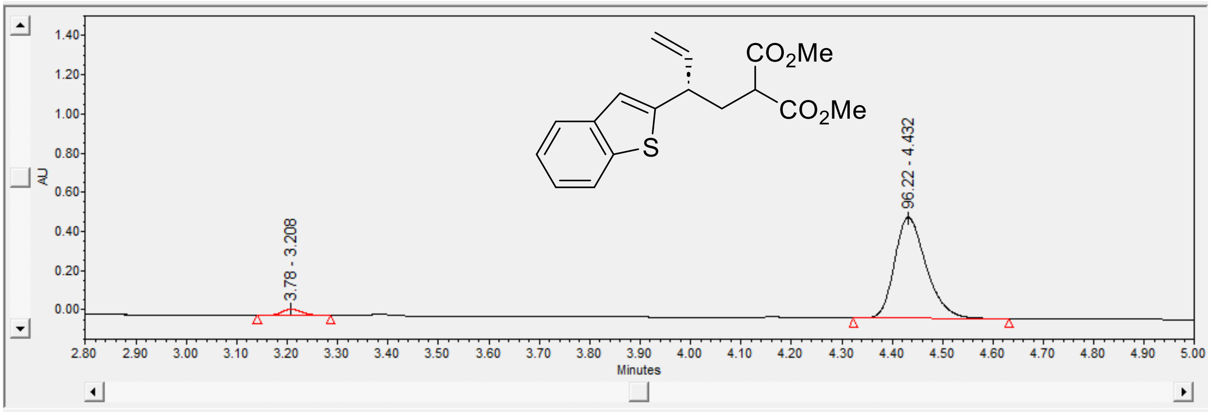

	Name	Retention Time (min)	Purity1 Angle	Purity1 Threshold	PDA/FLR Match1 Spect. Name	PDA/FLR Match1 Angle	PDA/FLR Match1 Threshold	PDA/FLR Match1 Lib. Name	Area (µV*sec)	% Area	Height (µV)	Int Type	Amount	Units	Peak Type	Peak Codes
1		3.208							88251	3.78	32348	bb			Unknown	
2		4.432							2243530	96.22	515154	bb			Unknown	

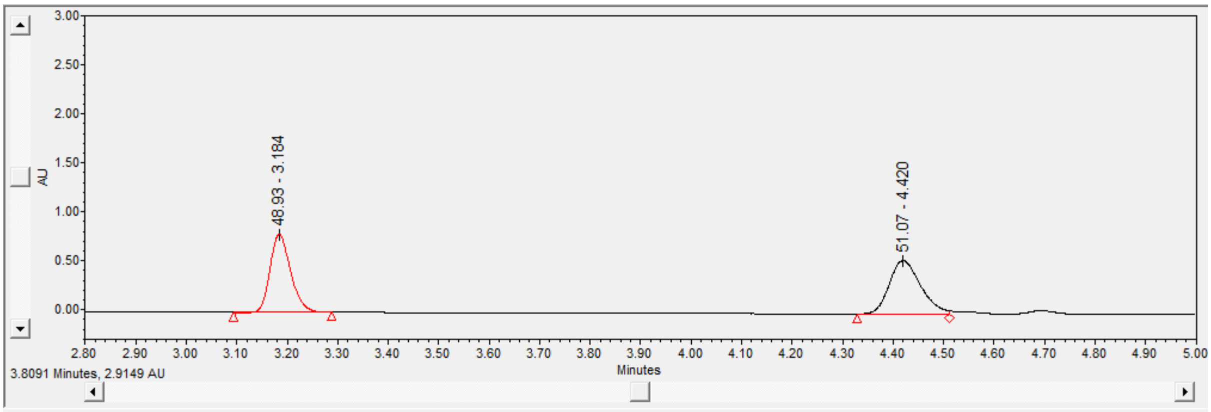

	Name	Retention Time (min)	Purity1 Angle	Purity1 Threshold	PDA/FLR Match1 Spect. Name	PDA/FLR Match1 Angle	PDA/FLR Match1 Threshold	PDA/FLR Match1 Lib. Name	Area (µV*sec)	% Area	Height (µV)	Int Type	Amount	Units	Peak Type	Peak Codes
1		3.184							2250154	48.93	796059	bb			Unknown	
2		4.420							2348831	51.07	544355	bv			Unknown	

SFC trace for (*S*)-**3x** and (±)-**3x**.

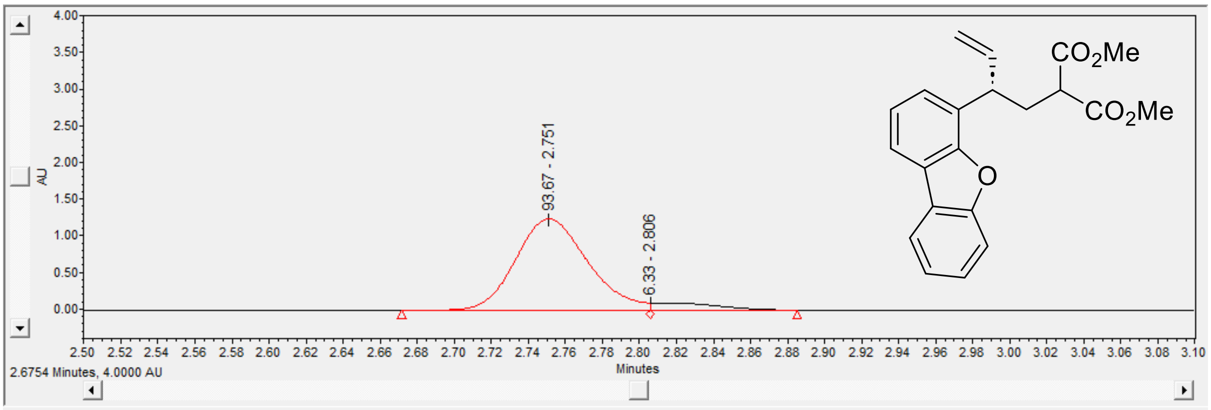

	Name	Retention Time (min)	Purity1 Angle	Purity1 Threshold	PDA/FLR Match1 Spect. Name	PDA/FLR Match1 Angle	PDA/FLR Match1 Threshold	PDA/FLR Match1 Lib. Name	Area (µV*sec)	% Area	Height (µV)	Int Type	Amount	Units	Peak Type	Peak Codes
1		2.751							3354373	93.67	1241064	bv			Unknown	
2		2.806							226655	6.33	94201	vb			Unknown	I06

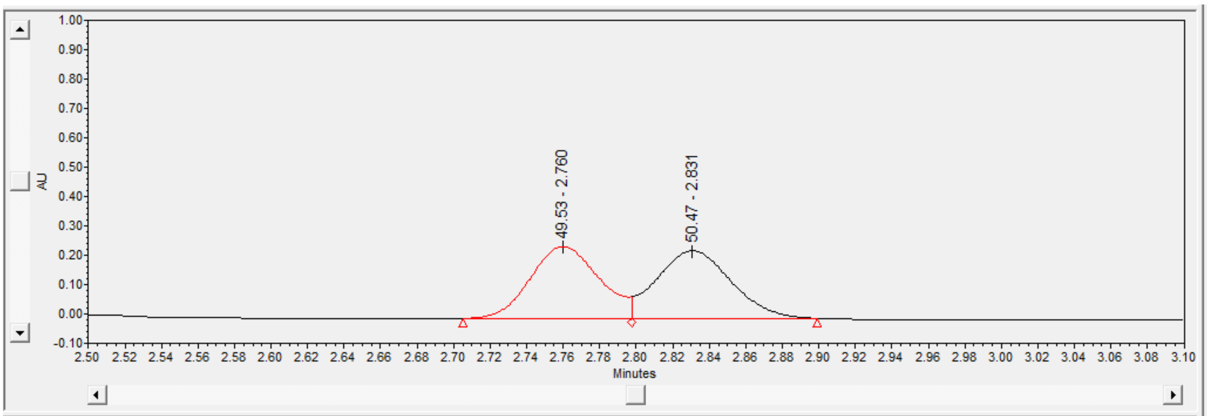

	Name	Retention Time (min)	Purity1 Angle	Purity1 Threshold	PDA/FLR Match1 Spect. Name	PDA/FLR Match1 Angle	PDA/FLR Match1 Threshold	PDA/FLR Match1 Lib. Name	Area (µV*sec)	% Area	Height (µV)	Int Type	Amount	Units	Peak Type	Peak Codes
1		2.760							640063	49.53	244196	bv			Unknown	
2		2.831							652238	50.47	231012	vb			Unknown	

SFC trace for (*S*)-**3y** and (±)-**3y**.

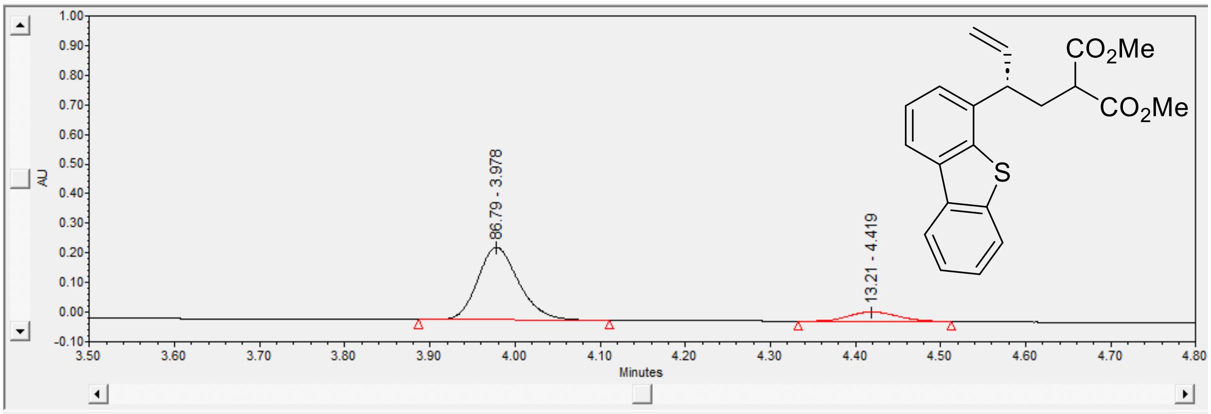

	Name	Retention Time (min)	Purity1 Angle	Purity1 Threshold	PDA/FLR Match1 Spect. Name	PDA/FLR Match1 Angle	PDA/FLR Match1 Threshold	PDA/FLR Match1 Lib. Name	Area (µV*sec)	% Area	Height (µV)	Int Type	Amount	Units	Peak Type	Peak Codes
1		3.978							821177	86.79	244621	bb			Unknown	
2		4.419							124971	13.21	31471	bb			Unknown	

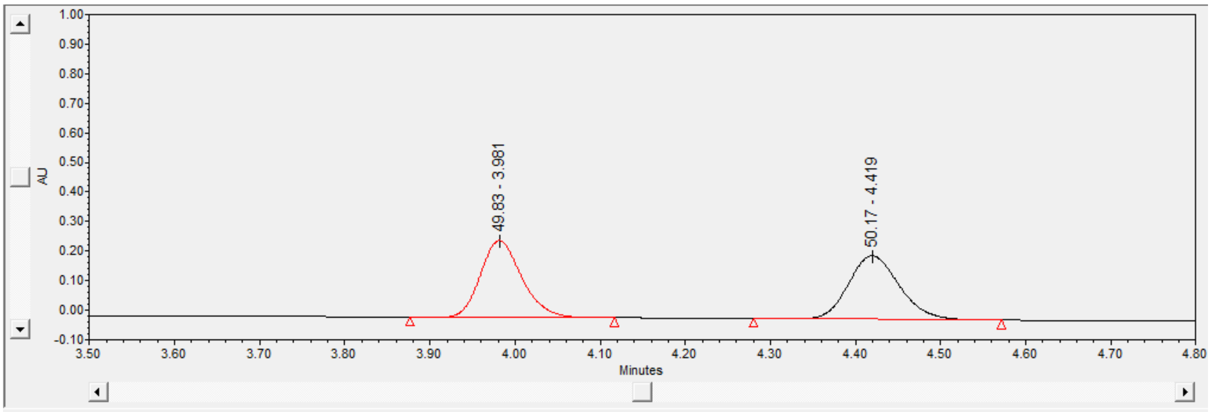

	Name	Retention Time (min)	Purity1 Angle	Purity1 Threshold	PDA/FLR Match1 Spect. Name	PDA/FLR Match1 Angle	PDA/FLR Match1 Threshold	PDA/FLR Match1 Lib. Name	Area (µV*sec)	% Area	Height (µV)	Int Type	Amount	Units	Peak Type	Peak Codes
1		3.981							881174	49.83	260612	bb			Unknown	
2		4.419							887027	50.17	214895	bb			Unknown	

SFC trace for (*S*)-**3z** and (±)-**3z**.

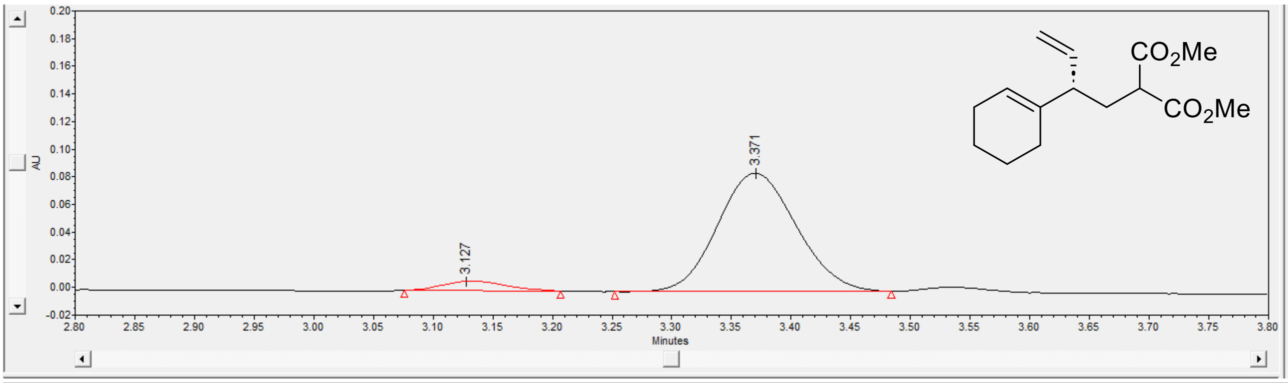

	Name	Retention Time (min)	Purity1 Angle	Purity1 Threshold	PDA/FLR Match1 Spect. Name	PDA/FLR Match1 Angle	PDA/FLR Match1 Threshold	PDA/FLR Match1 Lib. Name	Area (µV*sec)	% Area	Height (µV)	Int Type	Amount	Units	Peak Type	Peak Codes
1		3.127							25002	5.99	6808	bb			Unknown	
2		3.371							392048	94.01	85555	bb			Unknown	

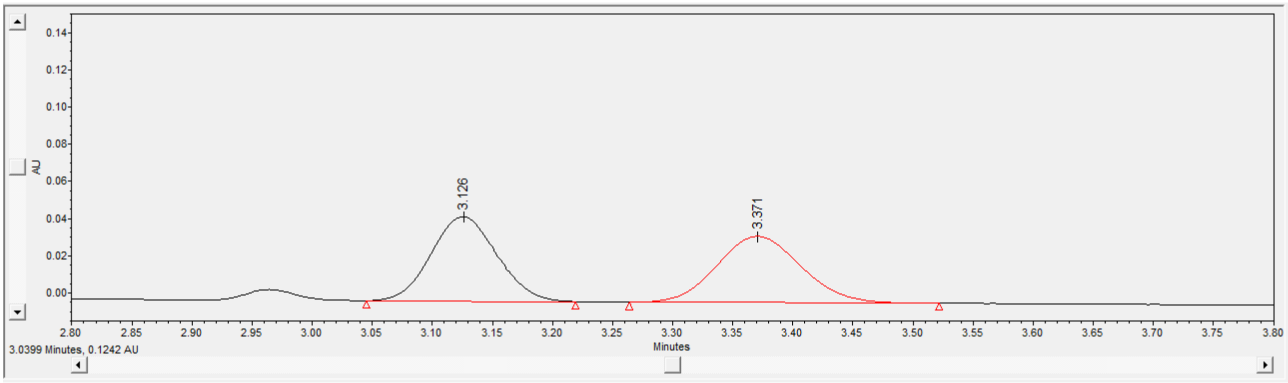

	Name	Retention Time (min)	Purity1 Angle	Purity1 Threshold	PDA/FLR Match1 Spect. Name	PDA/FLR Match1 Angle	PDA/FLR Match1 Threshold	PDA/FLR Match1 Lib. Name	Area (µV*sec)	% Area	Height (µV)	Int Type	Amount	Units	Peak Type	Peak Codes
1		3.126							172213	49.68	45395	bb			Unknown	
2		3.371							174454	50.32	35645	bb			Unknown	

SFC trace for (*S*)-**3aa** and (±)-**3aa**.

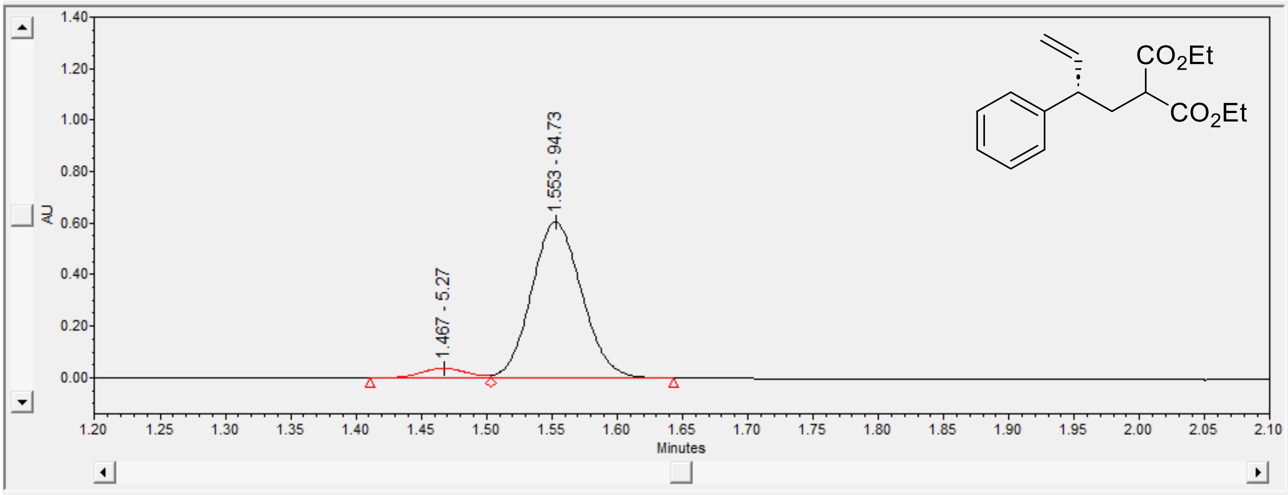

	Name	Retention Time (min)	Purity1 Angle	Purity1 Threshold	PDA/FLR Match1 Spect. Name	PDA/FLR Match1 Angle	PDA/FLR Match1 Threshold	PDA/FLR Match1 Lib. Name	Area (µV*sec)	% Area	Height (µV)	Int Type	Amount	Units	Peak Type	Pe
1		1.467							89977	5.27	37348	bv			Unknown	
2		1.553							1618433	94.73	606762	vb			Unknown	

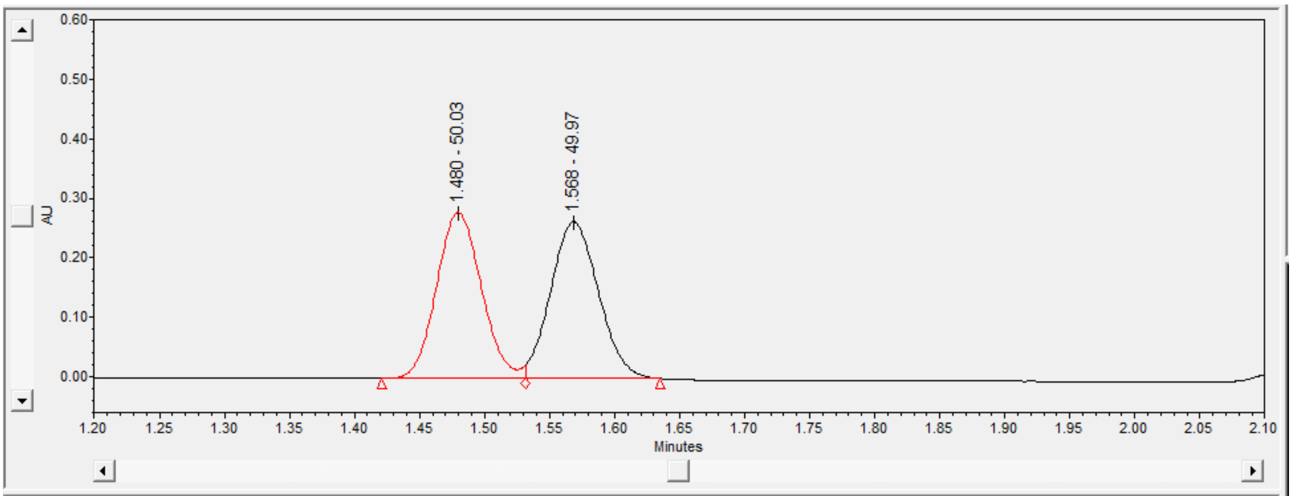

	Name	Retention Time (min)	Purity1 Angle	Purity1 Threshold	PDA/FLR Match1 Spect. Name	PDA/FLR Match1 Angle	PDA/FLR Match1 Threshold	PDA/FLR Match1 Lib. Name	Area (µV*sec)	% Area	Height (µV)	Int Type	Amount	Units	Peak Type	Pe
1		1.480							672181	50.03	279384	bv			Unknown	
2		1.568							671415	49.97	263592	vb			Unknown	

SFC trace for (*S*)-**4a** and (±)-**4a**.

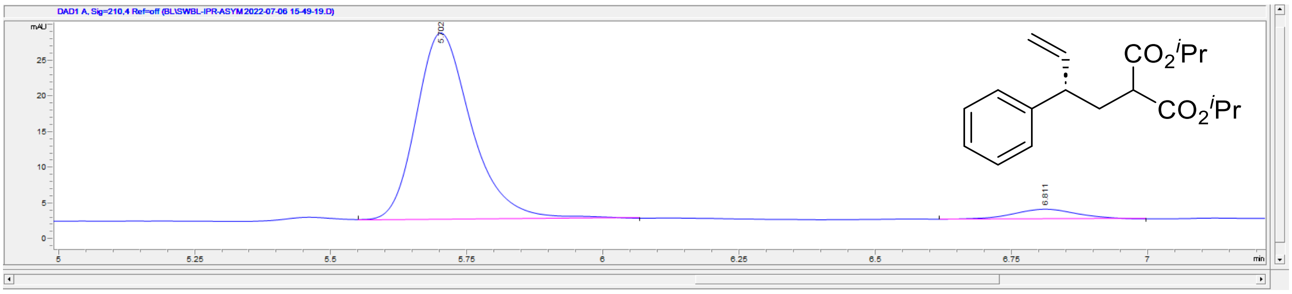

#	Time	Area	Height	Width	Area%	Symmetry
1	5.702	186.8	26.2	0.1071	94.289	0.741
2	6.811	11.3	1.4	0.1239	5.711	0.948

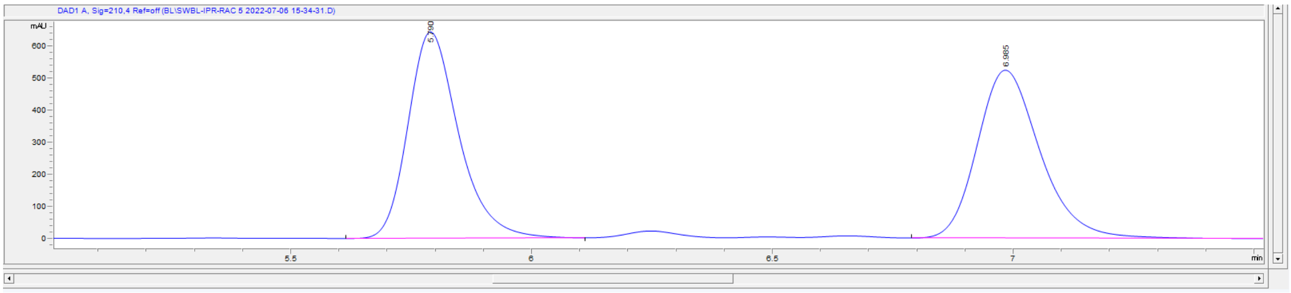

#	Time	Area	Height	Width	Area%	Symmetry
1	5.79	4736.2	644.1	0.1118	49.853	0.743
2	6.985	4764.2	523.7	0.1381	50.147	0.76

HPLC trace for (*S*)-**5a** and (±)-**5a**.

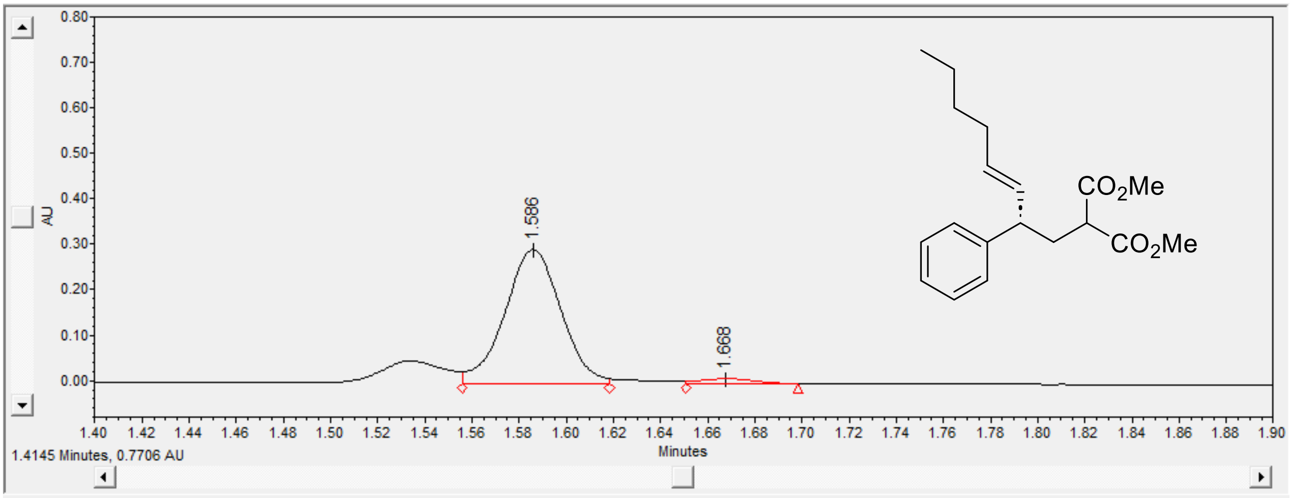

	Name	Retention Time (min)	Purity1 Angle	Purity1 Threshold	PDA/FLR Match1 Spect. Name	PDA/FLR Match1 Angle	PDA/FLR Match1 Threshold	PDA/FLR Match1 Lib. Name	Area (µV*sec)	% Area	Height (µV)	Int Type	Amount	Units	Peak Type	Pe
1		1.586							485856	96.73	294045	vv			Unknown	
2		1.668							16401	3.27	10374	vb			Unknown	

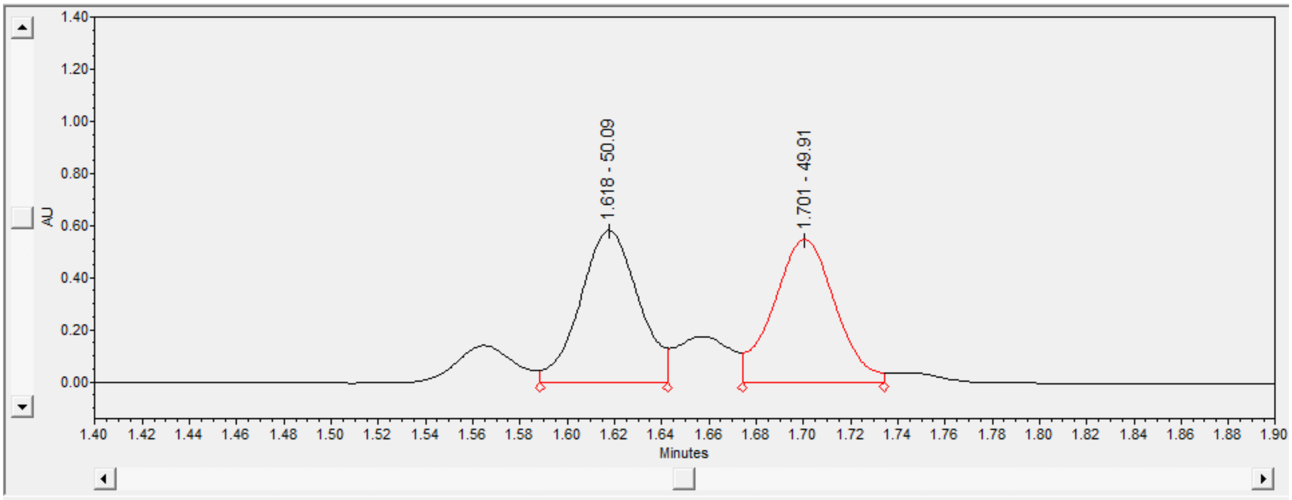

	Name	Retention Time (min)	Purity1 Angle	Purity1 Threshold	PDA/FLR Match1 Spect. Name	PDA/FLR Match1 Angle	PDA/FLR Match1 Threshold	PDA/FLR Match1 Lib. Name	Area (µV*sec)	% Area	Height (µV)	Int Type	Amount	Units	Peak Type	Pe
1		1.618							999288	50.09	584448	vv			Unknown	
2		1.701							995820	49.91	548523	vv			Unknown	

SFC trace for (*S*)-**7a** and (±)-**7a**.

SFC Trace for this compound contains both the (*E*) and (Z) alkenes; the enantiomeric excess reported is for the major (*E*)-Alkene product of the reaction. The (*Z*)-alkene peaks are too small to give accurate ee. In the first two traces below the (*E*)-alkene peaks are highlighted (asymmetric and then racemic); in the next two traces both the (*E*) and (*Z*) peaks are highlighted.

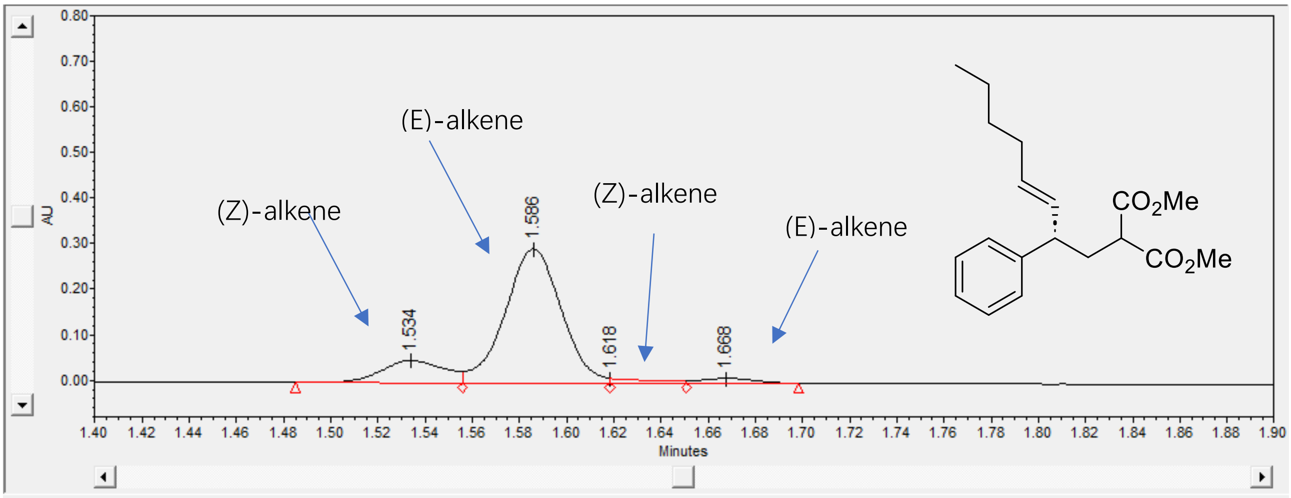

	Name	Retention Time (min)	Purity1 Angle	Purity1 Threshold	PDA/FLR Match1 Spect. Name	PDA/FLR Match1 Angle	PDA/FLR Match1 Threshold	PDA/FLR Match1 Lib. Name	Area (µV*sec)	% Area	Height (µV)	Int Type	Amount	Units	Peak Type	Pe
1		1.534							89816	14.89	49031	bv			Unknown	
2		1.586							485856	80.56	294045	vv			Unknown	
3		1.618							11054	1.83	9633	vv			Unknown	I0
4		1.668							16401	2.72	10374	vb			Unknown	

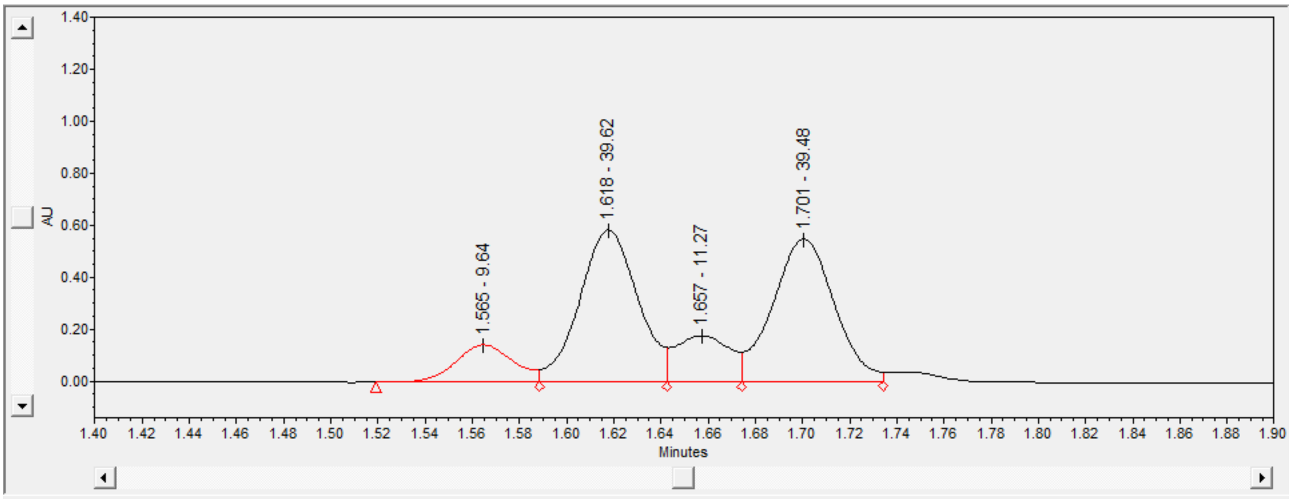

	Name	Retention Time (min)	Purity1 Angle	Purity1 Threshold	PDA/FLR Match1 Spect. Name	PDA/FLR Match1 Angle	PDA/FLR Match1 Threshold	PDA/FLR Match1 Lib. Name	Area (µV*sec)	% Area	Height (µV)	Int Type	Amount	Units	Peak Type	Pe
1		1.565							243179	9.64	143631	bv			Unknown	
2		1.618							999288	39.62	584448	vv			Unknown	
3		1.657							284165	11.27	177011	vv			Unknown	
4		1.701							995820	39.48	548523	vv			Unknown	

SFC trace for (*S*)-**7a** and (±)-**7a**.

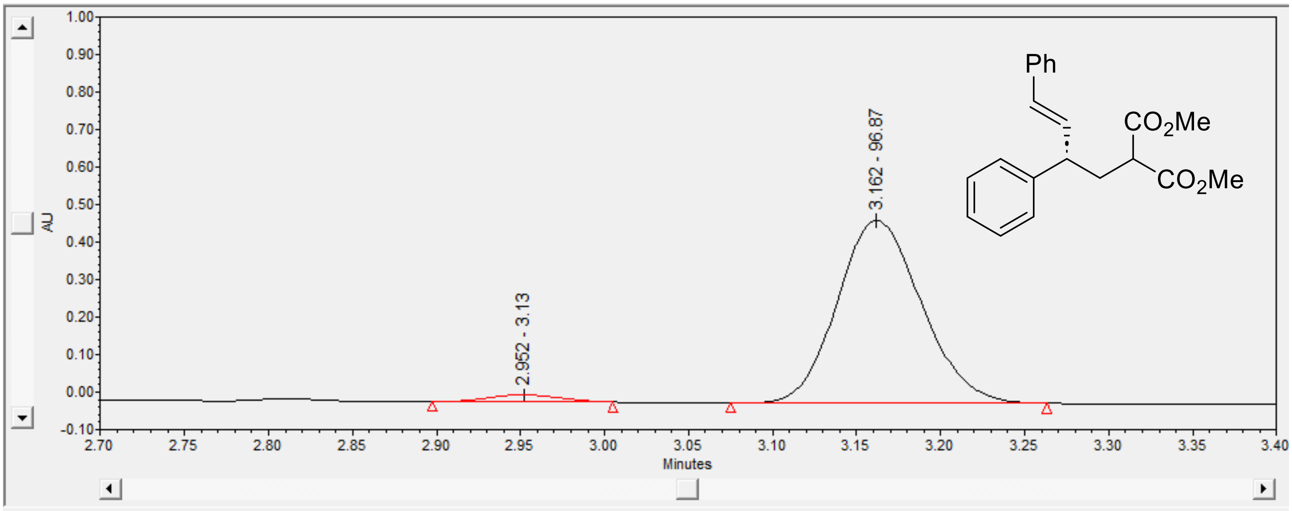

	Name	Retention Time (min)	Purity1 Angle	Purity1 Threshold	PDA/FLR Match1 Spect. Name	PDA/FLR Match1 Angle	PDA/FLR Match1 Threshold	PDA/FLR Match1 Lib. Name	Area (µV*sec)	% Area	Height (µV)	Int Type	Amount	Units	Peak Type	Pe
1		2.952							54774	3.13	18724	bb			Unknown	
2		3.162							1697131	96.87	488251	bb			Unknown	

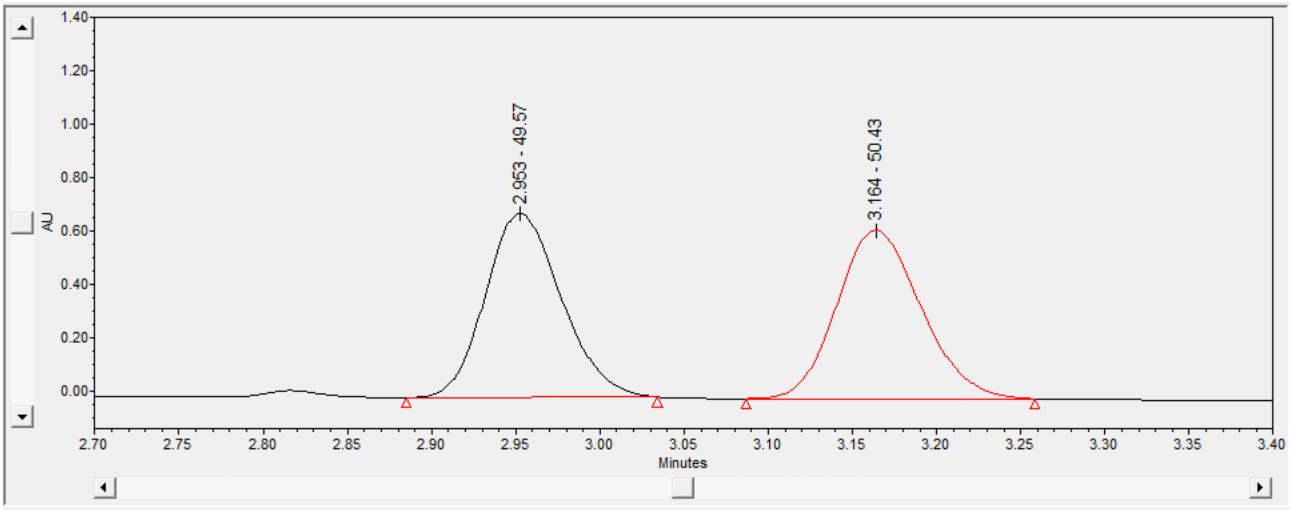

	Name	Retention Time (min)	Purity1 Angle	Purity1 Threshold	PDA/FLR Match1 Spect. Name	PDA/FLR Match1 Angle	PDA/FLR Match1 Threshold	PDA/FLR Match1 Lib. Name	Area (µV*sec)	% Area	Height (µV)	Int Type	Amount	Units	Peak Type	Pe
1		2.953							2161230	49.57	689515	bb			Unknown	
2		3.164							2198615	50.43	631652	bb			Unknown	

SFC trace for (*S*)-**7b** and (±)-**7b**.

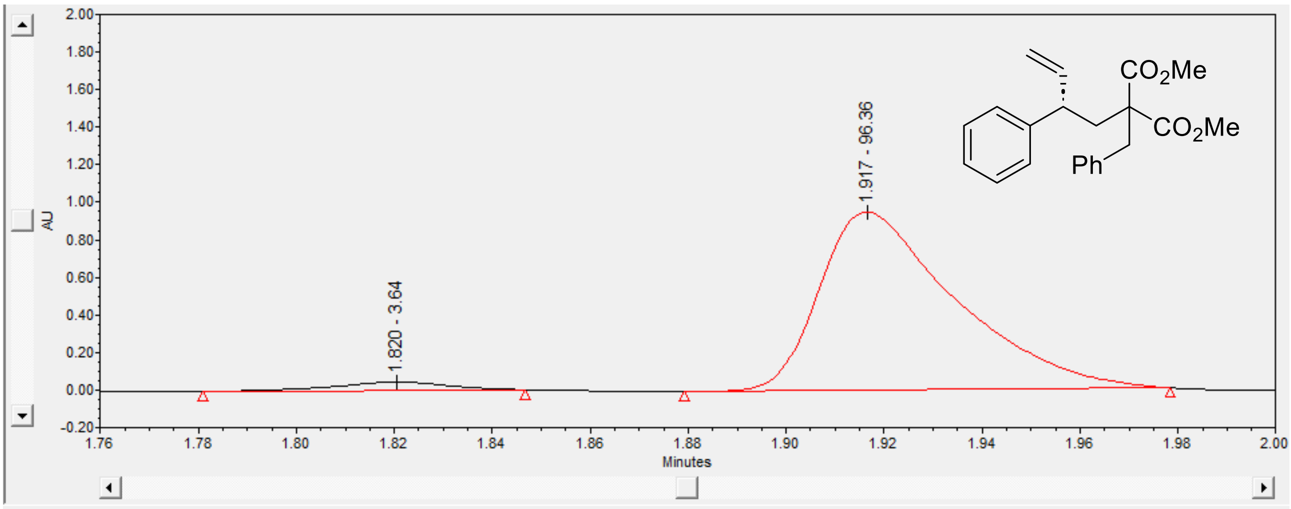

	Name	Retention Time (min)	Purity1 Angle	Purity1 Threshold	PDA/FLR Match1 Spect. Name	PDA/FLR Match1 Angle	PDA/FLR Match1 Threshold	PDA/FLR Match1 Lib. Name	Area (µV*sec)	% Area	Height (µV)	Int Type	Amount	Units	Peak Type	Pe
1		1.820							69251	3.64	44940	bb			Unknown	
2		1.917							1834908	96.36	947941	bb			Unknown	

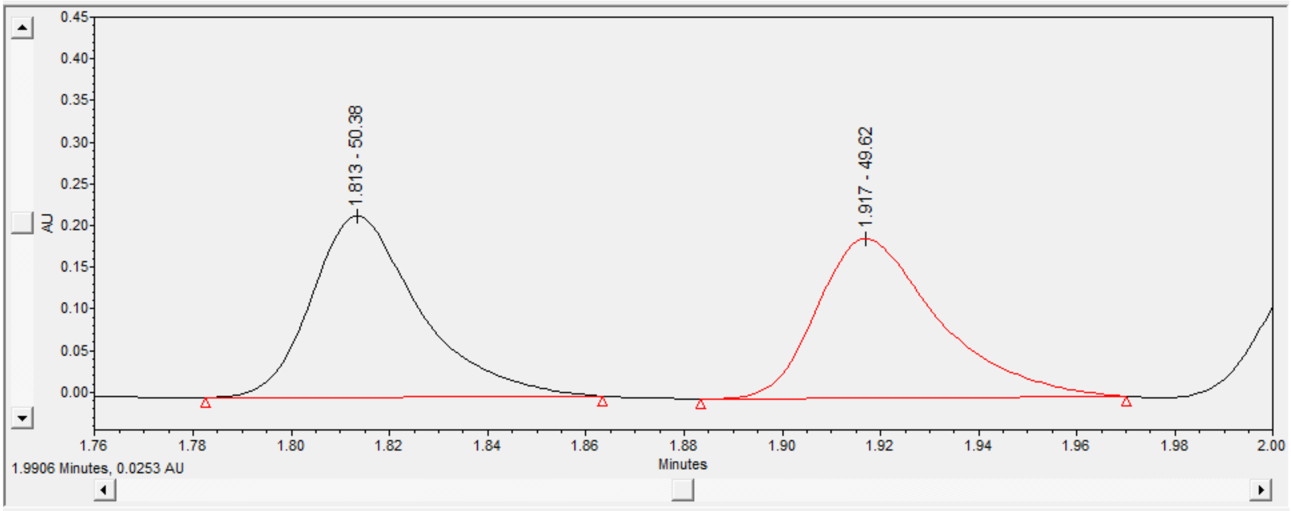

	Name	Retention Time (min)	Purity1 Angle	Purity1 Threshold	PDA/FLR Match1 Spect. Name	PDA/FLR Match1 Angle	PDA/FLR Match1 Threshold	PDA/FLR Match1 Lib. Name	Area (µV*sec)	% Area	Height (µV)	Int Type	Amount	Units	Peak Type	Pe
1		1.813							332989	50.38	217513	bb			Unknown	
2		1.917							327904	49.62	191539	bb			Unknown	

SFC trace for (*S*)-**8** and (±)-**8**.

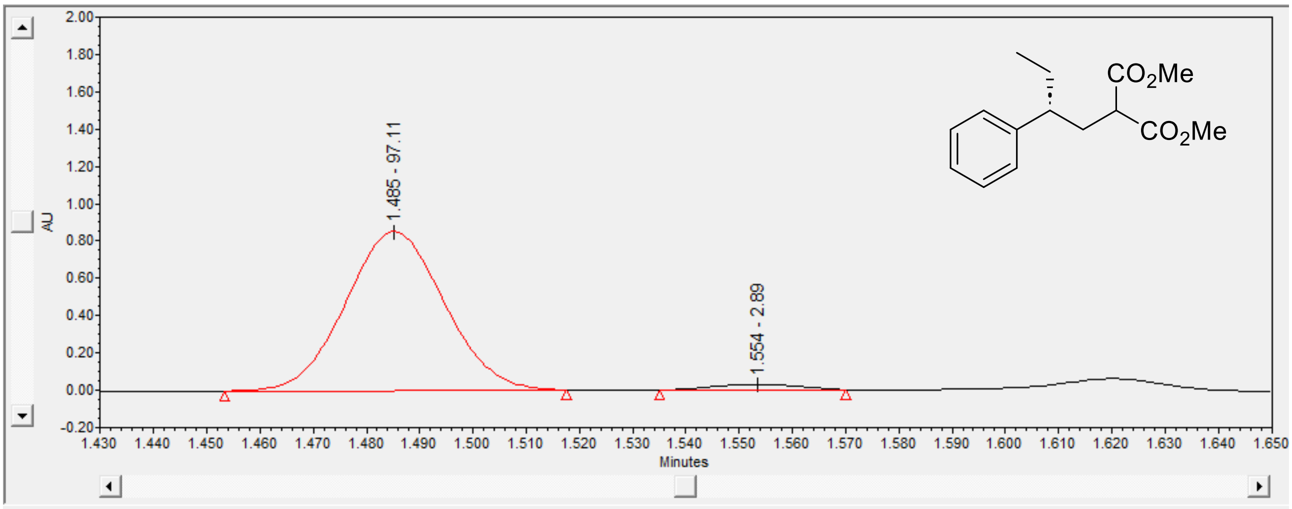

	Name	Retention Time (min)	Purity1 Angle	Purity1 Threshold	PDA/FLR Match1 Spect. Name	PDA/FLR Match1 Angle	PDA/FLR Match1 Threshold	PDA/FLR Match1 Lib. Name	Area (µV*sec)	% Area	Height (µV)	Int Type	Amount	Units	Peak Type	Pe
1		1.485							1096439	97.11	852098	bb			Unknown	
2		1.554							32586	2.89	28583	bb			Unknown	

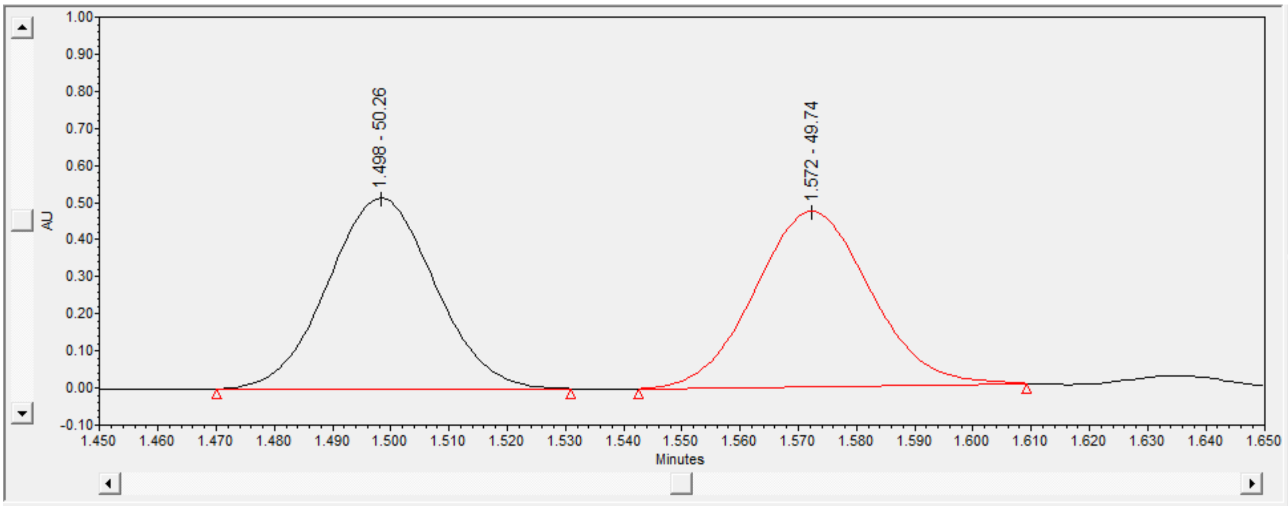

	Name	Retention Time (min)	Purity1 Angle	Purity1 Threshold	PDA/FLR Match1 Spect. Name	PDA/FLR Match1 Angle	PDA/FLR Match1 Threshold	PDA/FLR Match1 Lib. Name	Area (µV*sec)	% Area	Height (µV)	Int Type	Amount	Units	Peak Type	Pe
1		1.498							655988	50.26	514727	bb			Unknown	
2		1.572							649210	49.74	472003	bb			Unknown	

SFC trace for (*R*)-**9** and (±)-**9**.

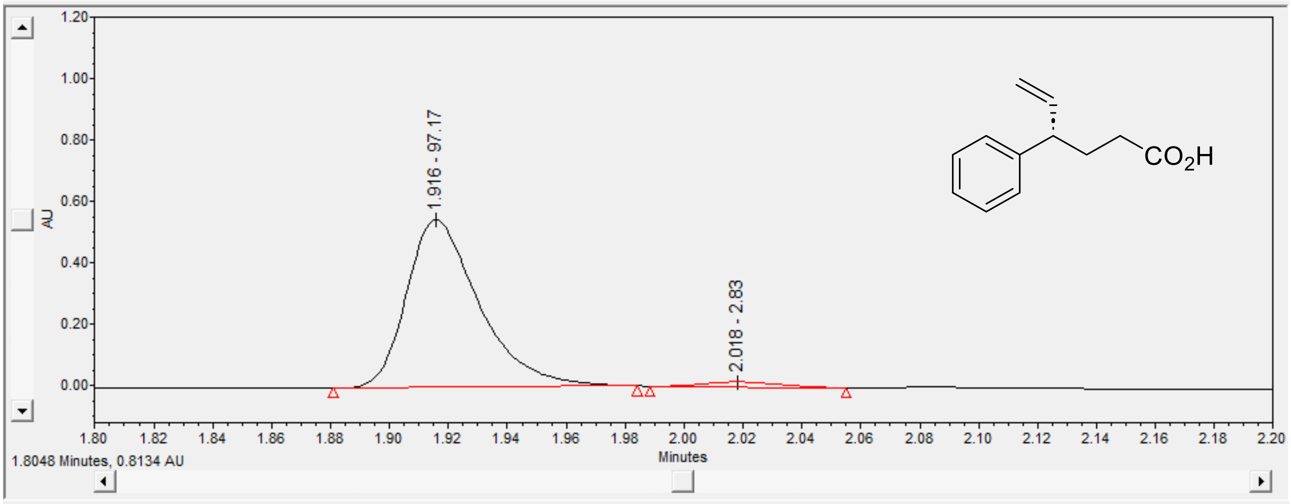

	Name	Retention Time (min)	Purity1 Angle	Purity1 Threshold	PDA/FLR Match1 Spect. Name	PDA/FLR Match1 Angle	PDA/FLR Match1 Threshold	PDA/FLR Match1 Lib. Name	Area (µV*sec)	% Area	Height (µV)	Int Type	Amount	Units	Peak Type	Pe
1		1.916							936498	97.17	546910	bb			Unknown	
2		2.018							27295	2.83	16111	bb			Unknown	

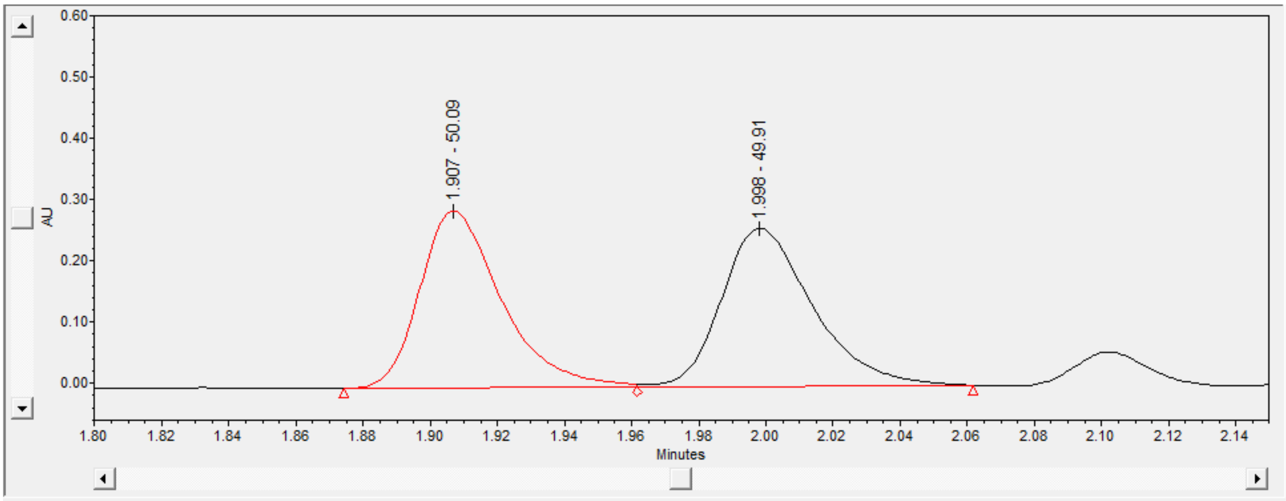

	Name	Retention Time (min)	Purity1 Angle	Purity1 Threshold	PDA/FLR Match1 Spect. Name	PDA/FLR Match1 Angle	PDA/FLR Match1 Threshold	PDA/FLR Match1 Lib. Name	Area (µV*sec)	% Area	Height (µV)	Int Type	Amount	Units	Peak Type	Pe
1		1.907							489246	50.09	289328	bv			Unknown	
2		1.998							487465	49.91	258093	vb			Unknown	

SFC trace for (*S*)-**10** and (±)-**10**.

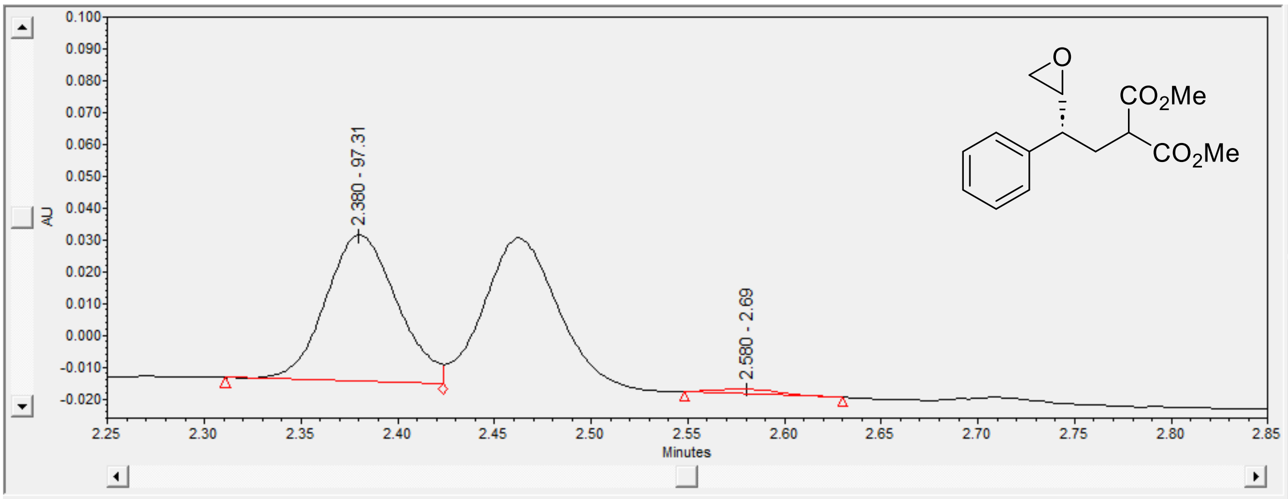

	Name	Retention Time (min)	Purity1 Angle	Purity1 Threshold	PDA/FLR Match1 Spect. Name	PDA/FLR Match1 Angle	PDA/FLR Match1 Threshold	PDA/FLR Match1 Lib. Name	Area (µV*sec)	% Area	Height (µV)	Int Type	Amount	Units	Peak Type	Pea
1		2.380							118449	97.31	46058	bv			Unknown	
2		2.580							3271	2.69	1561	bb			Unknown	

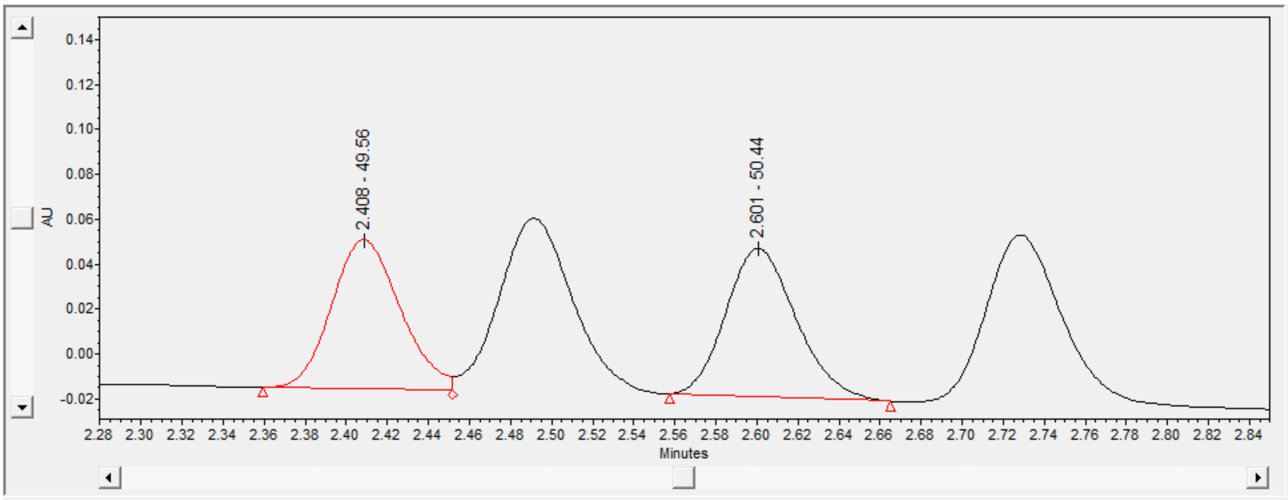

	Name	Retention Time (min)	Purity1 Angle	Purity1 Threshold	PDA/FLR Match1 Spect. Name	PDA/FLR Match1 Angle	PDA/FLR Match1 Threshold	PDA/FLR Match1 Lib. Name	Area (µV*sec)	% Area	Height (µV)	Int Type	Amount	Units	Peak Type	Pea
1		2.408							153962	49.56	66457	bv			Unknown	
2		2.601							156715	50.44	66025	bb			Unknown	

SFC trace for (*R*)-**12** and (±)-**12**.

SFC Trace for this compound contains both diastereomers (1:1 mixture).

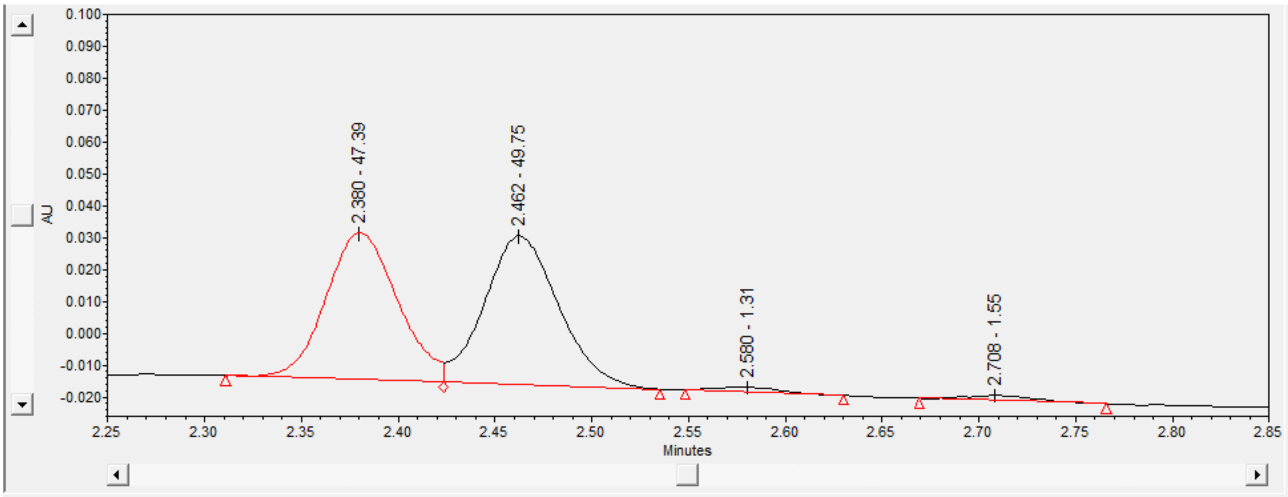

	Name	Retention Time (min)	Purity1 Angle	Purity1 Threshold	PDA/FLR Match1 Spect. Name	PDA/FLR Match1 Angle	PDA/FLR Match1 Threshold	PDA/FLR Match1 Lib. Name	Area (µV*sec)	% Area	Height (µV)	Int Type	Amount	Units	Peak Type	Pea
1		2.380							118449	47.39	46058	bv			Unknown	
2		2.462							124360	49.75	46716	vb			Unknown	
3		2.580							3271	1.31	1561	bb			Unknown	
4		2.708							3867	1.55	1660	bb			Unknown	

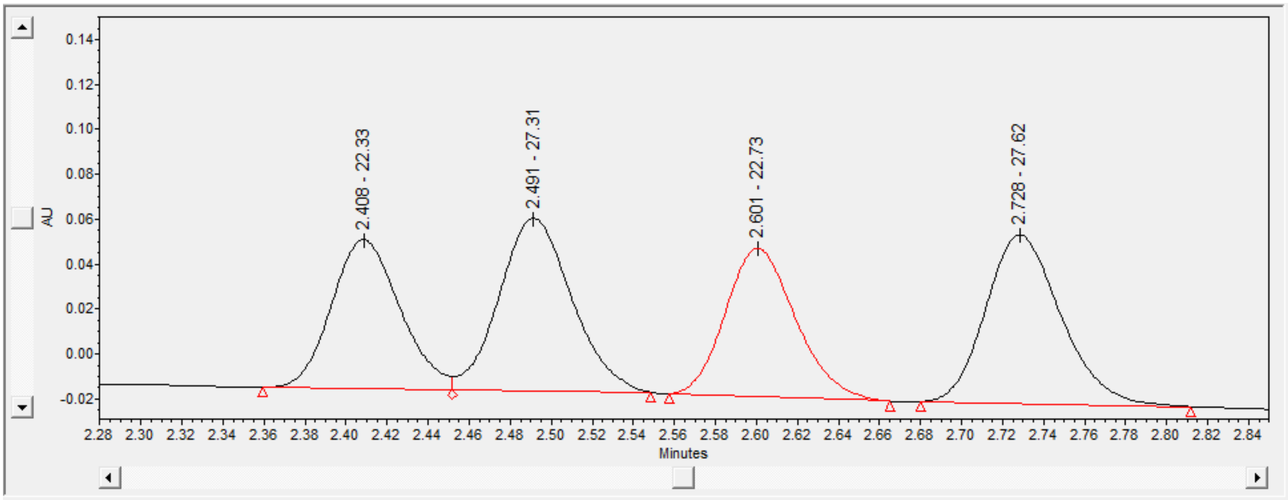

	Name	Retention Time (min)	Purity1 Angle	Purity1 Threshold	PDA/FLR Match1 Spect. Name	PDA/FLR Match1 Angle	PDA/FLR Match1 Threshold	PDA/FLR Match1 Lib. Name	Area (µV*sec)	% Area	Height (µV)	Int Type	Amount	Units	Peak Type	Pea
1		2.408							153962	22.33	66457	bv			Unknown	
2		2.491							188308	27.31	76759	vb			Unknown	
3		2.601							156715	22.73	66025	bb			Unknown	
4		2.728							190448	27.62	75050	bb			Unknown	

SFC trace for (*R*)-**12** and (±)-**12**.

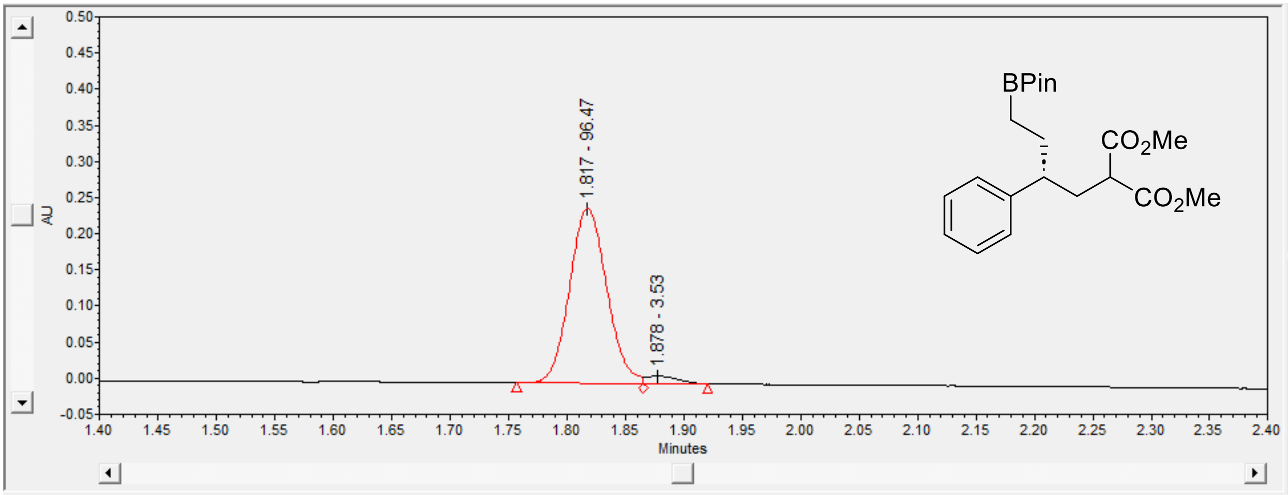

	Name	Retention Time (min)	Purity1 Angle	Purity1 Threshold	PDA/FLR Match1 Spect. Name	PDA/FLR Match1 Angle	PDA/FLR Match1 Threshold	PDA/FLR Match1 Lib. Name	Area (µV*sec)	% Area	Height (µV)	Int Type	Amount	Units	Peak Type	Pe
1		1.817							535332	96.47	242142	bv			Unknown	
2		1.878							19578	3.53	10302	vb			Unknown	

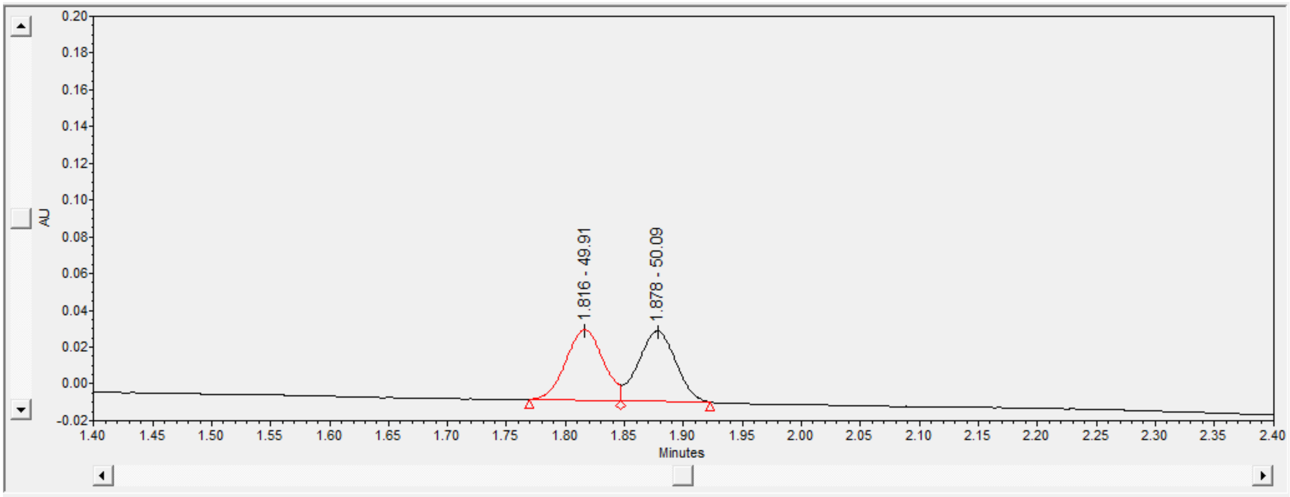

	Name	Retention Time (min)	Purity1 Angle	Purity1 Threshold	PDA/FLR Match1 Spect. Name	PDA/FLR Match1 Angle	PDA/FLR Match1 Threshold	PDA/FLR Match1 Lib. Name	Area (µV*sec)	% Area	Height (µV)	Int Type	Amount	Units	Peak Type	Peak Codes
1		1.816							83829	49.91	38281	bv			Unknown	
2		1.878							84145	50.09	38206	vb			Unknown	

SFC trace for (*R*)-**13** and (±)-**13**.

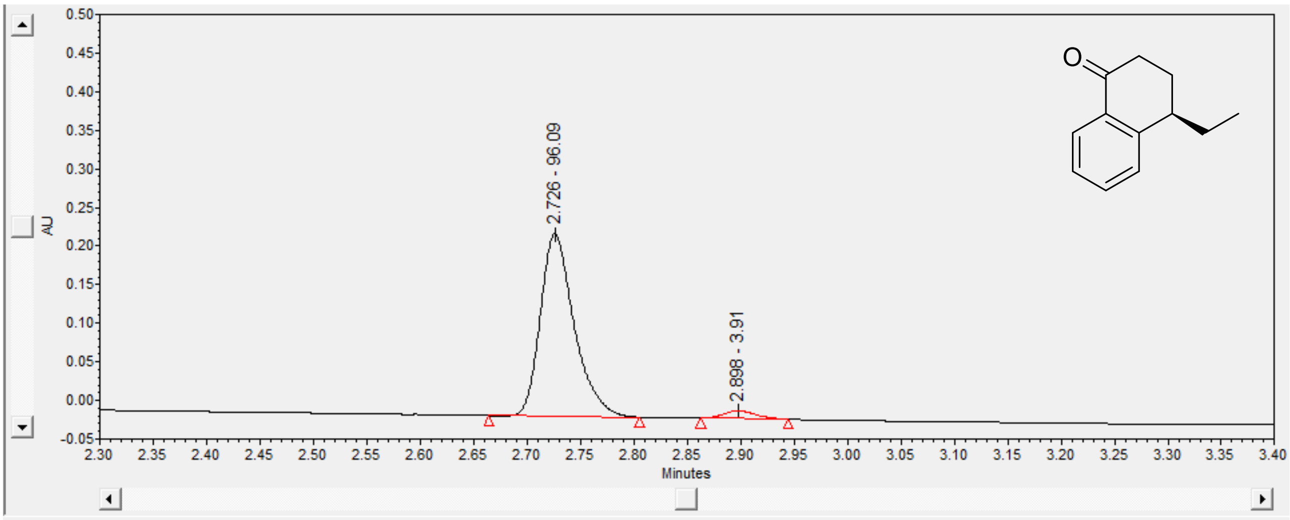

	Name	Retention Time (min)	Purity1 Angle	Purity1 Threshold	PDA/FLR Match1 Spect. Name	PDA/FLR Match1 Angle	PDA/FLR Match1 Threshold	PDA/FLR Match1 Lib. Name	Area (µV*sec)	% Area	Height (µV)	Int Type	Amount	Units	Peak Type	Pe
1		2.726							521219	96.09	236739	bb			Unknown	
2		2.898							21218	3.91	10253	bb			Unknown	

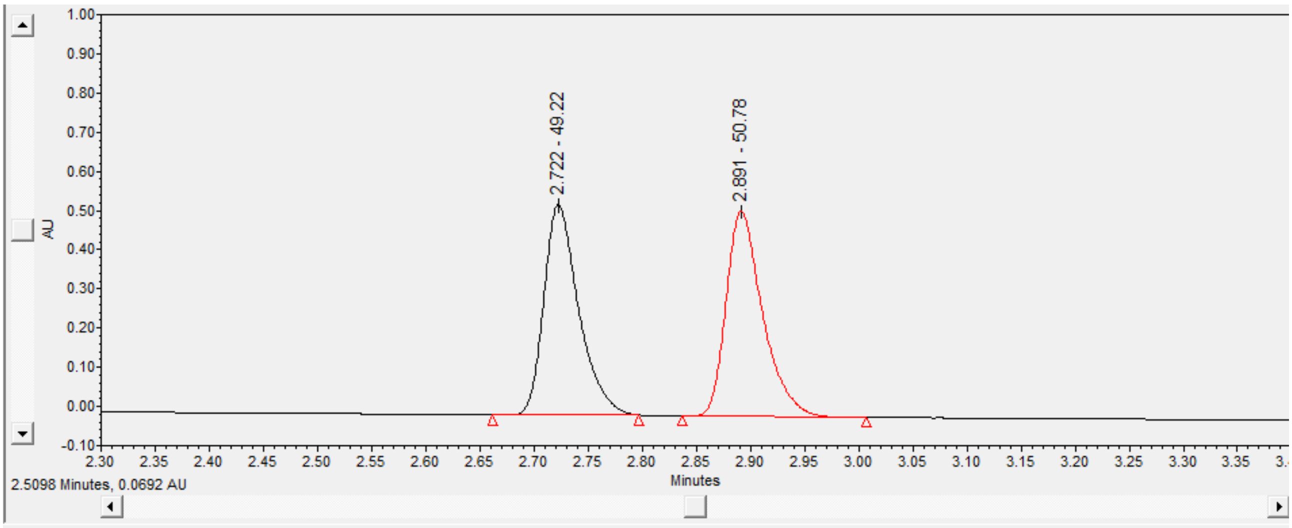

	Name	Retention Time (min)	Purity1 Angle	Purity1 Threshold	PDA/FLR Match1 Spect. Name	PDA/FLR Match1 Angle	PDA/FLR Match1 Threshold	PDA/FLR Match1 Lib. Name	Area (µV*sec)	% Area	Height (µV)	Int Type	Amount	Units	Peak Type
1		2.722							1199671	49.22	537467	bb			Unknown
2		2.891							1237909	50.78	524039	bb			Unknown

SFC trace for (*R*)-**14** and (±)-**14**.

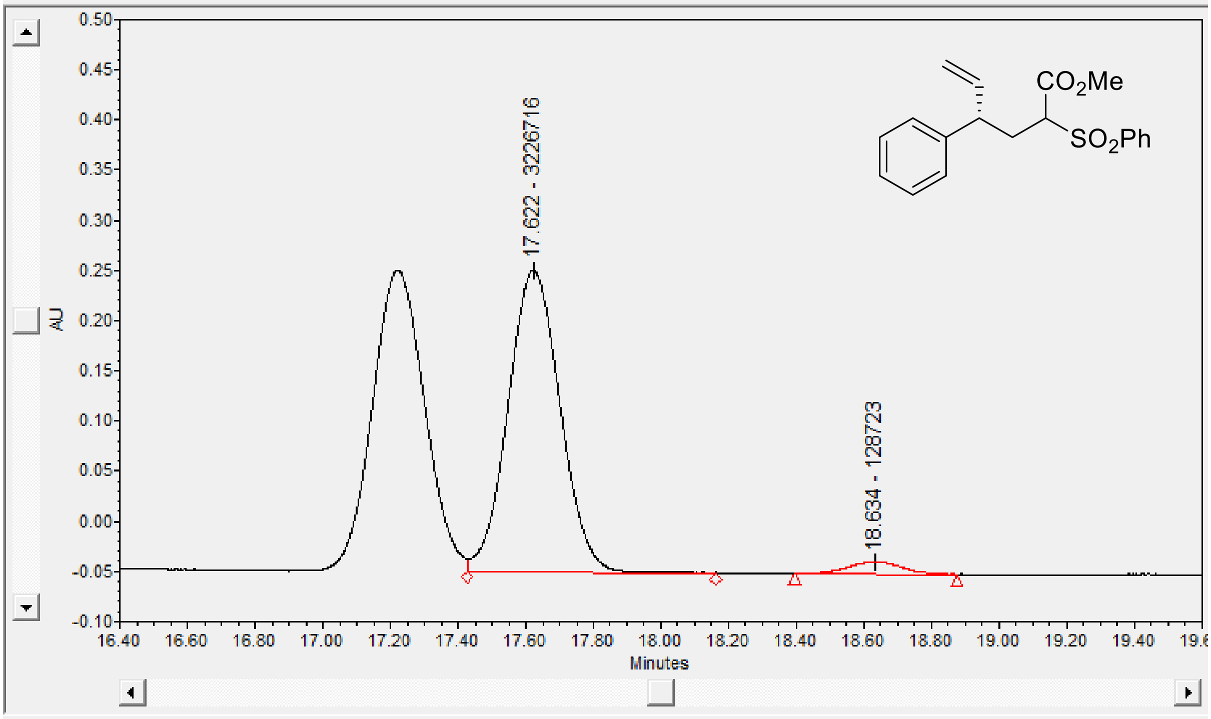

Name	Retention Time (min)	Purity1 Angle	Purity1 Threshold	PDA/FLR Match1 Spect. Name	PDA/FLR Match1 Angle	PDA/FLR Match1 Threshold	PDA/FLR Match1 Lib. Name	Area (µV*sec)	% Area	Height (µV)
1	17.622							3226716	96.16	300996
2	18.634							128723	3.84	11987

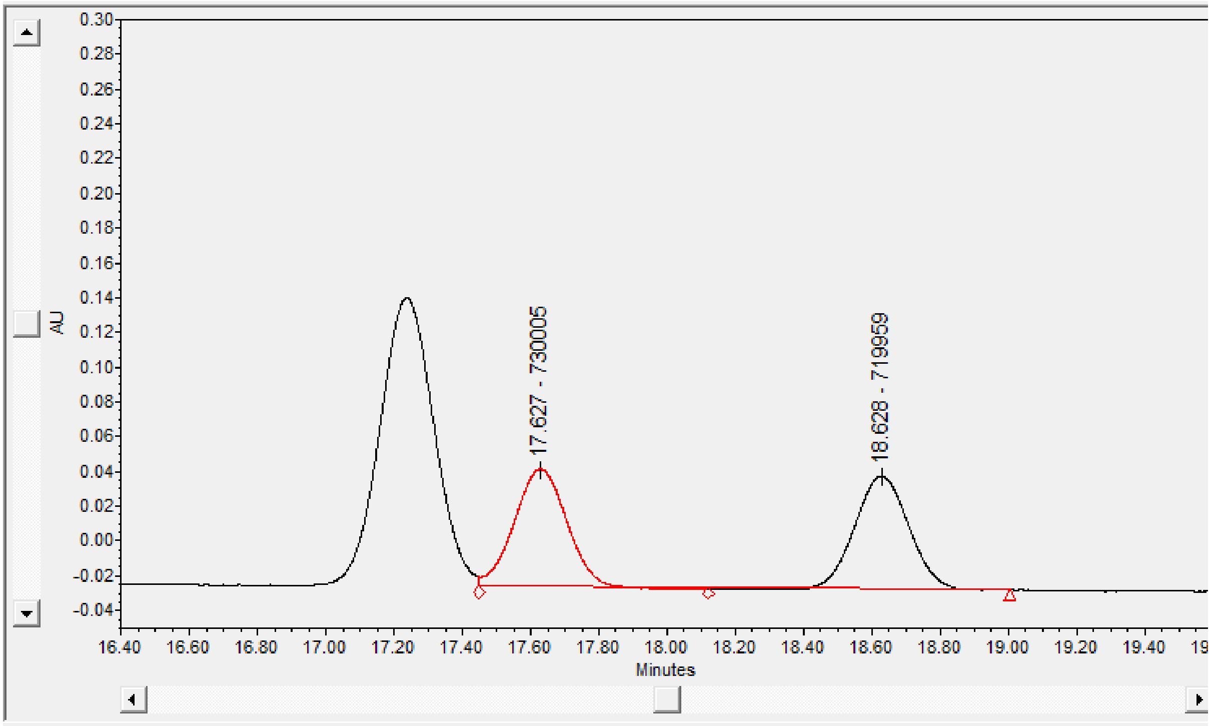

Name	Retention Time (min)	Purity1 Angle	Purity1 Threshold	PDA/FLR Match1 Spect. Name	PDA/FLR Match1 Angle	PDA/FLR Match1 Threshold	PDA/FLR Match1 Lib. Name	Area (µV*sec)	% Area	Height (µV)
1	17.627							730005	50.35	67571
2	18.628							719959	49.65	64632

SFC trace for (*S*)-**22a** and (±)-**22a**.

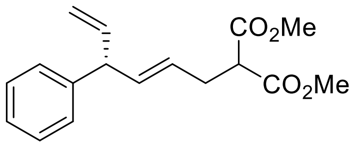

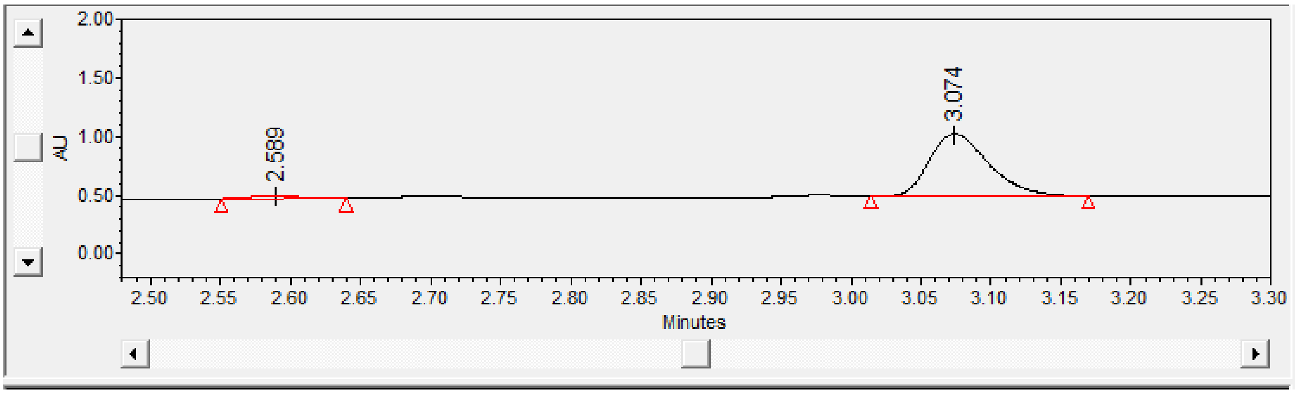

	Name	Retention Time (min)	Purity1 Angle	Purity1 Threshold	PDA/FLR Match1 Spect. Name	PDA/FLR Match1 Angle	PDA/FLR Match1 Threshold	PDA/FLR Match1 Lib. Name	Area (µV*sec)	% Area	Height (µV)
1		2.589							65333	4.00	26746
2		3.074							1567652	96.00	530087

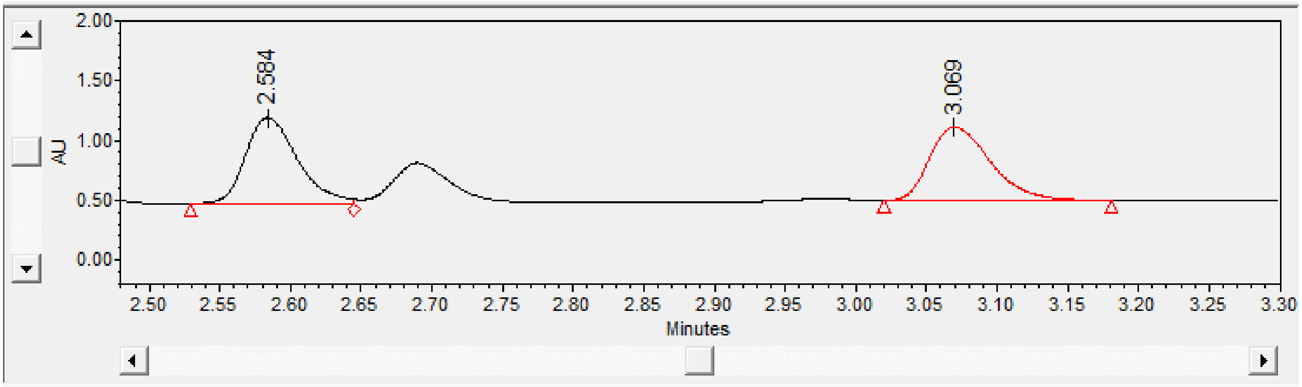

	Name	Retention Time (min)	Purity1 Angle	Purity1 Threshold	PDA/FLR Match1 Spect. Name	PDA/FLR Match1 Angle	PDA/FLR Match1 Threshold	PDA/FLR Match1 Lib. Name	Area (µV*sec)	% Area	Height (µV)
1		2.584							1831864	50.04	720411
2		3.069							1828665	49.96	616949

SFC trace for (*R*)-**24** and (±)-**24**.

11. References

(1) Dieskau, A. P.; Holzwarth, M. S.; Plietker, B. Fe-Catalyzed Allylic C–C-Bond Activation: Vinylcyclopropanes As Versatile A1,A3,D5-Synthons in Traceless Allylic Substitutions and [3 + 2]-Cycloadditions. *J. Am. Chem. Soc.* **2012**, *134*, 5048–5051. https://doi.org/10.1021/ja300294a.

(2) Yin, J.; Hyland, C. J. T. Ring-Opening of Vinylcyclopropane-1,1-Dicarboxylates by Boronic Acids under Ligandless Palladium Catalysis in Neat Water. *J. Org. Chem.* **2015**, *80*, 6529–6536. https://doi.org/10.1021/acs.joc.5b00672.

(3) Saha, S.; Debnath, B.; Talukdar, K.; Karjee, P.; Mandal, S.; Punniyamurthy, T. Cascade C–H Activation/Annulation of Sulfoxonium Ylides with Vinyl Cyclopropanes: Access to Cyclopropane-Fused α-Tetralones. *Org. Lett.* **2023**, *25*, 3352–3357. https://doi.org/10.1021/acs.orglett.3c00650.

(4) de Nanteuil, F.; Serrano, E.; Perrotta, D.; Waser, J. Dynamic Kinetic Asymmetric [3 + 2] Annulation Reactions of Aminocyclopropanes. *J. Am. Chem. Soc.* **2014**, *136*, 6239–6242. https://doi.org/10.1021/ja5024578.

(5) Ronzon, Q.; Zhang, W.; Charote, T.; Casaretto, N.; Frison, G.; Nay, B. Total Synthesis of (+)-Cinereain and (−)-Janoxepin through a Fragment Coupling/Retro-Claisen Rearrangement Cascade. *Angew. Chem. Int. Ed.* **2022**, *61*, e202212855. https://doi.org/https://doi.org/10.1002/anie.202212855.

(6) Bitai, J.; Nimmo, A. J.; Slawin, A. M. Z.; Smith, A. D. Cooperative Palladium/Isothiourea Catalyzed Enantioselective Formal (3+2) Cycloaddition of Vinylcyclopropanes and α,β-Unsaturated Esters. *Angew. Chem. Int. Ed.* **2022**, *61*, e202202621. https://doi.org/https://doi.org/10.1002/anie.202202621.

(7) Chandu, P.; Mallick, M.; Srinivasu, V.; Sureshkumar, D. Organophotocatalyzed Alkyl/Arylsulfonylation of Vinylcyclopropanes. *Chem. – A Eur. J.* **2024**, *30*, e202303187. https://doi.org/https://doi.org/10.1002/chem.202303187.

(8) Xiao, Y.-Q.; Li, M.-M.; Zhou, Z.-X.; Li, Y.-J.; Cao, M.-Y.; Liu, X.-P.; Lu, H.-H.; Rao, L.; Lu, L.-Q.; Beauchemin, A. M.; Xiao, W.-J. Taming Chiral Quaternary Stereocenters via Remote H-Bonding Stereoinduction in Palladium-Catalyzed (3+2) Cycloadditions. *Angew. Chem. Int. Ed.* **2023**, *62*, e202212444. https://doi.org/https://doi.org/10.1002/anie.202212444.

(9) Bowman, R. K.; Johnson, J. S. Nickel-Catalyzed Rearrangement of 1-Acyl-2-Vinylcyclopropanes. A Mild Synthesis of Substituted Dihydrofurans. *Org. Lett.* **2006**, *8*, 573–576. https://doi.org/10.1021/ol052700k.

(10) Vriesen, M. R.; Grover, H. K.; Kerr, M. A. Advancing the Reactivity of Dimethylcyclopropane-1,1-Dicarboxylates via Cross Metathesis. *Synlett* **2014**, *25* , 428–432.

(11) De Simone, F.; Saget, T.; Benfatti, F.; Almeida, S.; Waser, J. Formal Homo-Nazarov and Other Cyclization Reactions of Activated Cyclopropanes. *Chem. – A Eur. J.* **2011**, *17*, 14527–14538. https://doi.org/https://doi.org/10.1002/chem.201102583.

(12) Ma, Y.; Yun, Y. K.; Wondergem (nee Lukesh), J.; Sar, A.; Gone, J. R.; Lindeman, S.; Donaldson, W. A. Reactivity of (1-Methoxycarbonylpentadienyl)Iron(1+) Cations with Hydride, Methyl, and Nitrogen Nucleophiles. *Tetrahedron* **2017**, *73*, 4493–4500. https://doi.org/https://doi.org/10.1016/j.tet.2017.06.026.

(13) Kohara, K.; Trowbridge, A.; Smith, M. A.; Gaunt, M. J. Thiol-Mediated α-Amino Radical

Formation via Visible-Light-Activated Ion-Pair Charge-Transfer Complexes. *J. Am. Chem. Soc.* **2021**, *143*, 19268–19274. https://doi.org/10.1021/jacs.1c09445.

(14) Ren Jilai; Ma, Weiwei; Wang, Zhongwen, J. B. Tandem Diels–Alder [4+2] Cycloadditions and Intramolecular [3+2] Cross-Cycloadditions of Dienylcyclopropane 1,1-Diesters. *Synlett* **2014**, *25*, 2260–2264. https://doi.org/10.1055/s-0034-1378897.

(15) Sherry, B. D.; Fürstner, A. Iron-Catalyzed Addition of Grignard Reagents to Activated Vinyl Cyclopropanes. *Chem. Commun.* **2009**, No. 46, 7116–7118. https://doi.org/10.1039/B918818E.

(16) van Dijk, L.; Ardkhean, R.; Sidera, M.; Karabiyikoglu, S.; Sari, Ö.; Claridge, T. D. W.; Lloyd-Jones, G. C.; Paton, R. S.; Fletcher, S. P. Mechanistic Investigation of Rh(i)-Catalysed Asymmetric Suzuki–Miyaura Coupling with Racemic Allyl Halides. *Nat. Catal.* **2021**, *4*, 284–292. https://doi.org/10.1038/s41929-021-00589-y.

(17) Evans, R.; Deng, Z.; Rogerson, A. K.; McLachlan, A. S.; Richards, J. J.; Nilsson, M.; Morris, G. A. Quantitative Interpretation of Diffusion-Ordered NMR Spectra: Can We Rationalize Small Molecule Diffusion Coefficients? *Angew. Chem. Int. Ed.* **2013**, *52*, 3199–3202. https://doi.org/https://doi.org/10.1002/anie.201207403.

(18) Preetz, A.; Drexler, H.-J.; Schulz, S.; Heller, D. BINAP: Rhodium–Diolefin Complexes in Asymmetric Hydrogenation. *Tetrahedron: Asymmetry* **2010**, *21*, 1226–1231. https://doi.org/https://doi.org/10.1016/j.tetasy.2010.03.017.

(19) Luo, W.; Sun, Z.; Fernando, E. H. N.; Nesterov, V. N.; Cundari, T. R.; Wang, H. Asymmetric Ring-Opening of Donor–Acceptor Cyclopropanes with Primary Arylamines Catalyzed by a Chiral Heterobimetallic Catalyst. *ACS Catal.* **2019**, *9*, 8285–8293. https://doi.org/10.1021/acscatal.9b02523.

(20) Xiong, H.; Xu, H.; Liao, S.; Xie, Z.; Tang, Y. Copper-Catalyzed Highly Enantioselective Cyclopentannulation of Indoles with Donor–Acceptor Cyclopropanes. *J. Am. Chem. Soc.* **2013**, *135*, 7851–7854. https://doi.org/10.1021/ja4042127.

(21) Xia, Y.; Lin, L.; Chang, F.; Fu, X.; Liu, X.; Feng, X. Asymmetric Ring-Opening of Cyclopropyl Ketones with Thiol, Alcohol, and Carboxylic Acid Nucleophiles Catalyzed by a Chiral N,N′-Dioxide–Scandium(III) Complex. *Angew. Chem. Int. Ed.* **2015**, *54*, 13748–13752. https://doi.org/https://doi.org/10.1002/anie.201506909.

(22) Chen, T.; Gan, L.; Wang, R.; Deng, Y.; Peng, F.; Lautens, M.; Shao, Z. Rhodium(I)/Zn(OTf)2-Catalyzed Asymmetric Ring Opening/Cyclopropanation of Oxabenzonorbornadienes with Phosphorus Ylides. *Angew. Chem. Int. Ed.* **2019**, *58*, 15819–15823. https://doi.org/https://doi.org/10.1002/anie.201909596.

(23) Stojalnikova, V.; Webster, S. J.; Liu, K.; Fletcher, S. P. Chelation Enables Selectivity Control in Enantioconvergent Suzuki–Miyaura Cross-Couplings on Acyclic Allylic Systems. *Nat. Chem.* **2024**. https://doi.org/10.1038/s41557-023-01430-8.

(24) Zhao, J.; Shi, J.; Li, Y. Benzyne-Mediated Esterification Reaction. *Org. Lett.* **2021**, *23*, 7274–7278. https://doi.org/10.1021/acs.orglett.1c02702.
